# A combined transcriptome and proteome analysis extends the allergome of house dust mite *Dermatophagoides* species

**DOI:** 10.1371/journal.pone.0185830

**Published:** 2017-10-05

**Authors:** Véronique Bordas-Le Floch, Maxime Le Mignon, Laetitia Bussières, Karine Jain, Armelle Martelet, Véronique Baron-Bodo, Emmanuel Nony, Laurent Mascarell, Philippe Moingeon

**Affiliations:** Stallergenes Greer, Antony cedex, France; Centre National de la Recherche Scientifique, FRANCE

## Abstract

**Background:**

House dust mites (HDMs) such as *Dermatophagoides farinae* and *D*. *pteronyssinus* represent major causes of perennial allergy. HDM proteomes are currently poorly characterized, with information mostly restricted to allergens. As of today, 33 distinct allergen groups have been identified for these 2 mite species, with groups 1 and 2 established as major allergens. Given the multiplicity of IgE-reactive mite proteins, potential additional allergens have likely been overlooked.

**Objective:**

To perform a comprehensive characterization of the transcriptomes, proteomes and allergomes of *D*. *farinae* and *D*. *pteronyssinus* in order to identify novel allergens.

**Methods:**

Transcriptomes were analyzed by RNA sequencing and *de novo* assembly. Comprehensive mass spectrometry-based analyses proteomes were combined with two-dimensional IgE reactivity profiling.

**Results:**

Transcripts from *D*. *farinae* and *D*. *pteronyssinus* were assembled, translated into protein sequences and used to populate derived sequence databases in order to inform immunoproteomic analyses. A total of 527 and 157 proteins were identified by bottom-up MS analyses in aqueous extracts from purified HDM bodies and fecal pellets, respectively. Based on high sequence similarities (>71% identity), we also identified 2 partial and 11 complete putative sequences of currently undisclosed *D*. *pteronyssinus* counterparts of *D*. *farinae* registered allergens. Immunoprofiling on 2D-gels revealed the presence of unknown 23 kDa IgE reactive proteins in both species. Following expression of non-glycosylated recombinant forms of these molecules, we confirm that these new allergens react with serum IgEs from 42% (8/19) of HDM-allergic individuals.

**Conclusions:**

Using combined transcriptome and immunoproteome approaches, we provide a comprehensive characterization of *D*. *farinae* and *D*. *pteronyssinus* allergomes. We expanded the known allergen repertoire for *D*. *pteronyssinus* and identified two novel HDM allergens, now officially referred by the International Union of Immunological Societies (IUIS) Nomenclature Subcommittee as Der f 36 and Der p 36.

## Introduction

House dust mites (HDMs) represent the main cause of perennial respiratory allergies, affecting more than half a billion people worldwide [1, 2]. Specifically, the *Pyroglyphidae* species *Dermatophagoides farinae* and *D*. *pteronyssinus* are responsible for up to 90% of allergies to HDMs [3]. As of today, 33 distinct allergen groups have been identified in these *Dermatophagoides* species, including 30 and 19 allergens officially recorded by the International Union of Immunological Societies (IUIS) Allergen Nomenclature Subcommittee (www.allergen.org) for *D*. *farinae* and *D*. *pteronyssinus*, respectively [4–8]. Among those, groups 1 and 2 are established as the most clinically relevant allergens, with a prevalence of IgE sensitization of 60%-100% in mite allergic patients [9–12] and a high potency (with anti-group 1 and anti-group 2 IgEs collectively representing 50-77% of total IgEs) [3, 9–11, 13–18]. In addition, Der p 23 has been identified as a new important allergen, with serum IgEs detected in 46-74% HDM-allergic patients albeit with a much lower potency, suggesting that it could be clinically relevant for selected patients [10, 19, 20]. Recently, Der f 35 has been described as a novel Der p 2-like candidate major allergen, but its clinical significance remains to be documented [21]. Groups 4, 5, 7 and 21 are considered as mid-prevalence/potency allergens, whereas the other groups are regarded as of minor or unknown importance [3–5, 22].

As of today, the proteomes and transcriptomes of *Dermatophagoides* species are only partially characterized. A comprehensive analysis of protein content of *Dermatophagoides* extracts by mass spectrometry (MS) has been limited to cross-species identification due to the limited sequence information available for HDMs. To address in part this issue, Expressed Sequence Tags databases have previously been used to inform HDM proteome analysis [23–25]. Nonetheless, a more detailed molecular characterization is still needed as *Dermatophagoides* extracts are commonly used for diagnostic and therapeutic purposes.

Recently, extensive transcriptome analysis relying upon next generation sequencing technologies have been successfully used by us and others to facilitate protein identification [26] in allergen sources as diverse as the mite *Tyrophagus putrescentiae* [27] and pollens from either Johnson grass [28], Timothy grass [29] or short ragweed [30]. In the present study, we performed such a combined analysis of the transcriptomes, proteomes and allergomes from *D*. *farinae* and *D*. *pteronyssinus* using deep RNA sequencing in association with MS/MS analyses and IgE reactivity profiling. We report on the comprehensive molecular characterization of the two *Dermatophagoides* species. Our results complement the known allergen repertoire of *D*. *pteronyssinus* and provide evidence for novel HDM allergens for the two species, officially registered by IUIS as Der f 36 and Der p 36.

## Methods

### Plasma samples

Plasma samples were collected from 19 HDM-allergic patients (Table 1) at hospital Bichat-Claude Bernard (Paris, France) after approval of the study protocol (ref. #120147–30) by an ethical committee and regulatory authorities (CPP Ile de France 1, RCB N° 2011-A01508-33). Written informed consents were obtained from patients. These patients suffer from allergic rhinitis (with ARIA scores ranging from Intermittent to Persistent Moderate/Severe), with or without concomitant mild asthma (GINA score 1). IgE sensitization to *D*. *farinae* and *D*. *pteronyssinus* was established based on positive skin prick tests as well as specific IgE levels ≥5 kU/L measured by ImmunoCAP with mite extracts (Thermo Fisher Scientific, Villebon-sur-Yvette, France). Plasma samples were used to assess the IgE reactivity of recombinant Der f 36 and Der p 36 in western blots.

**Table 1:** Characteristics of HDM-allergic patients included in the study.

Gender	Age	Symptoms	ARIA	GINA	IgEs (kU/L)		IgE reactivity with rDer f 36 and rDer p 36
	(years)		score	score	*D*. *farinae*	*D*. *pteronyssinus*	
Female	25	AR+A	PMS	1	83.8	97.9	+
Female	22	AR	I	-	66.5	88.1	+
Female	21	AR+A	I	1	53.4	97.5	+
Male	28	AR+A	PM	1	48.3	59.7	+
Male	23	AR+A	PM	1	39	52.5	+
Female	39	AR+A	PM	1	19.3	28.8	+
Female	33	AR	I	-	19	15.2	+
Male	33	AR+A	PM	1	13.6	11.9	+
Male	23	AR+A	PMS	1	75.8	78.8	-
Female	44	AR	PM	-	36.3	39	-
Female	28	AR+A	I	1	33.2	21.6	-
Female	33	AR	PM	-	21.9	15.2	-
Male	22	AR+A	I	1	16.9	21.5	-
Female	28	AR	I	-	11.7	17.3	-
Female	19	AR	PMS	-	11.7	12.7	-
Male	23	AR+A	I	1	8.8	11.1	-
Male	22	AR+A	PMS	1	8.6	13.4	-
Female	35	AR+A	PM	1	7.7	12.4	-
Female	33	AR+A	I	1	5.8	8.1	-

HDM-allergic patients included in the study suffer from allergic rhinitis (AR) with or without concomitant asthma (A). Rhinitis and asthma symptoms are ranked according to ARIA (I: intermittent; PM: persistent mild; PMS: persistent moderate/severe) and GINA (1: mild) scores, respectively. Specific IgE titers were measured by ImmunoCAP with *D*. *farinae* or *D*. *pteronyssinus* extracts. The + and − symbols refer to positive and negative IgE reactivity of plasmas with recombinant Der f 36 and Der p 36 allergens in western blot experiments.

### Mites

*D*. *farinae* and *D*. *pteronyssinus* were grown for approximately 80 days. Whole culture media, containing mites at all development stages, were and harvested as described elsewhere [10].

### Analysis of *Dermatophagoides sp*. transcriptomes

Transcriptomes were analyzed by deep RNA sequencing. To this aim, *D*. *farinae* and *D*. *pteronyssinus* (Stallergenes Greer, Antony, France) bodies were purified by sequential sieving and flotation [10]. Total RNAs were then isolated using the RNeasy kit (Qiagen, Courtaboeuf, France). Sequencing libraries were constructed by Vertis Biotechnologies (Freising, Germany). Beckman Coulter Genomics (Grenoble, France) performed sequencing using 454 (Roche Diagnostics, Meylan, France) and Hiseq2000 (Illumina, San Diego, USA) sequencers followed by *de novo* assembly with the Trinity/Oases suite [31]. For subsequent annotation, likely coding regions were identified using the Transdecoder program implemented in Trinity [32], translated into protein sequences and annotated by BLAST analysis against registered Arthropods proteins. Protein sequences shorter than 79 amino acids were excluded.

### Analysis of *Dermatophagoides* sp. proteomes

Bodies and fecal particles were purified by sieving and flotation from *D*. *farinae* and *D*. *pteronyssinus* whole cultures (Stallergenes Greer), as reported elsewhere [10]. Briefly, proteins from aqueous extracts, obtained from one batch of bodies or fecal particles for each species, were denatured, then disulfide bonds were reduced and alkylated. Peptides obtained by trypsin digestion were subsequently separated by reverse-phase liquid chromatography (LC) using an Ultimate 3000 RS nano LC system (Thermo Fisher Scientific) and analyzed by tandem mass spectrometry (MS/MS) using an Impact HD mass spectrometer equipped with a CaptiveSpray source (Bruker Daltonics, Wissembourg, France) [10]. Peptide identification was performed using the PEAKS software version 8 (Bioinformatics Solutions Inc., Waterloo, Canada), using the species-specific transcriptome-derived databases supplemented with sequences of IUIS-registered allergens as reference datasets.

To further characterize the proteomes, HDM extracts, obtained from one whole culture for each mite species, were submitted to two-dimensional electrophoresis on 3–10 non linear pH range 12.5% DALT gels (GE Healthcare, Velizy-Villacoublay, France), as per the manufacturers’ instructions. After staining with Sypro Ruby, gel plugs were excised from 2D-gels using an EXquest spot cutter (Bio-Rad, Marnes-La–Coquette, France), then destained and dehydrated. After in-gel digestion of proteins with trypsin, resulting peptides were analyzed by LC-MS/MS using the aforementioned equipment and identified using the PEAKS software. Further details on MS analyses and protein identification are provided in the online repository.

### Functional categorization

Subsets of proteins detected by at least two peptides in MS analyses of body and feces extracts were classified in functional categories, using the KEGG (Kyoto Encyclopedia of Genes and Genomes) Automatic Annotation Server (KAAS) [33]. KEGG orthologies (KO) were assigned using the single-directional best hit (SBH) method and the gene data set for arthropods.

### Recombinant allergen expression in *Pichia pastoris*

Coding sequences of Der f 36 and Der p 36 were retrieved from transcriptome analysis. Codon-optimized DNA fragments encoding mature non-glycosylated variants (Der f 36 N50Q N121Q and Der p 36 N50Q N121Q) were cloned flush with the α-factor signal sequence into the pPICZαA vector (Life technologies, Saint-Aubin, France). *P*. *pastoris* X33 cells were transformed with the resulting plasmids by electroporation using a Gene Pulser Xcell apparatus, according to the manufacturer’s instructions (Bio-Rad). Recombinant Der f 36 and Der p 36 were produced by growing yeast transformants in Enpresso Y medium (Biosilta, St. Ives, United Kingdom), as per the manufacturer’s instructions. Culture supernatants were harvested by centrifugation and stored at -20°C until use. A mock strain, obtained by transformation with the empty vector, was used as a negative control.

## Results

### Transcriptome and proteome analyses of *D*. *farinae* and *D*. *pteronyssinus*

**Fig 1:**
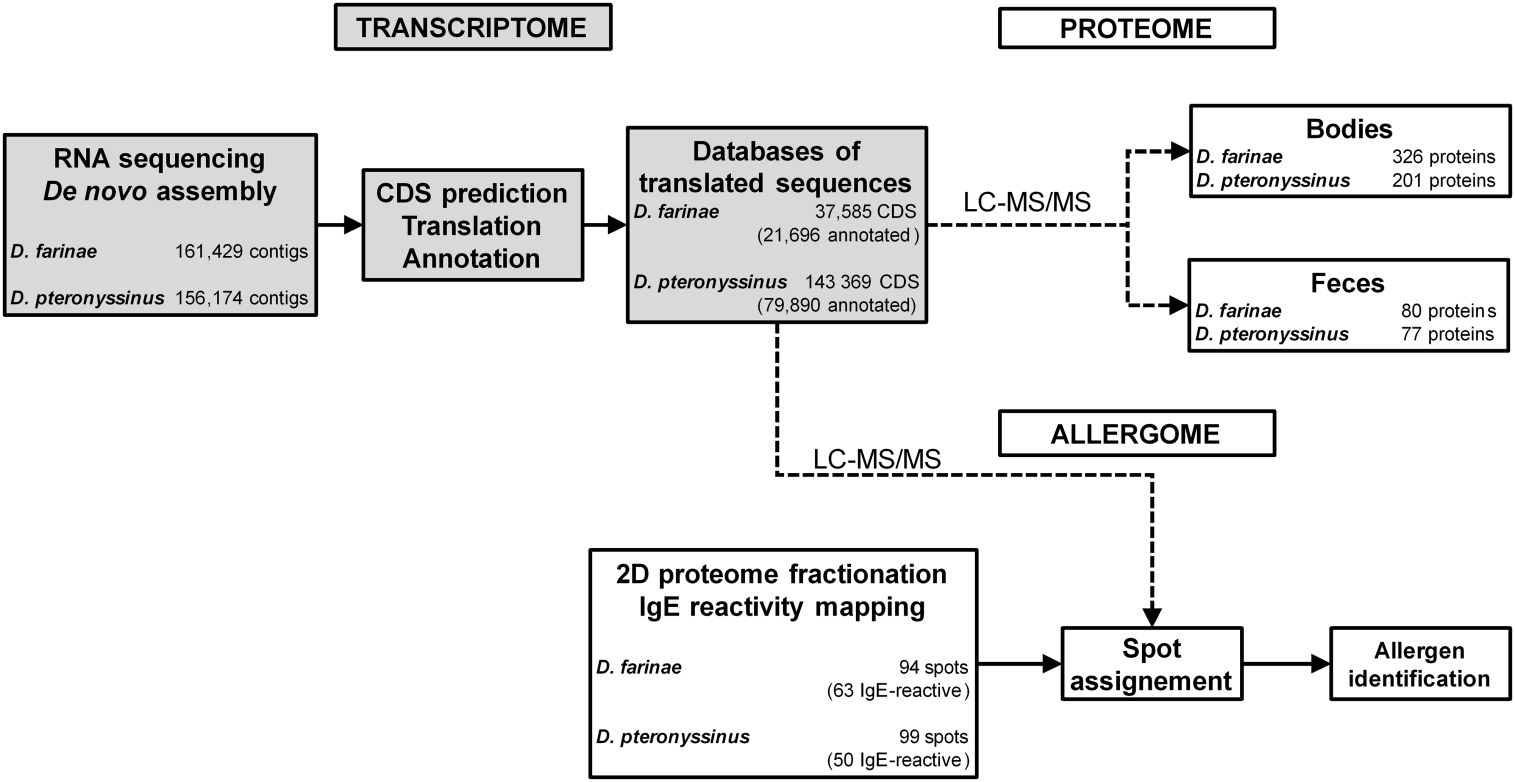
Experimental workflow of HDM transcriptome, proteome and allergome analyses. Messenger RNAs from *D*. *farinae* and *D*. *pteronyssinus* were sequenced using next generation sequencing. Following *de novo* assembly, translated sequence databases were derived after coding sequences (CDS) prediction and used as references to identify proteins in aqueous extracts from mite bodies and feces by LC-MS/MS analysis. In parallel, extracts from whole cultures were submitted to 2D-gel electrophoresis to establish reference 2D maps of species-specific proteomes and assign IgE reactivity.

We implemented a combined approach relying on deep RNA sequencing, MS/MS analyses and IgE reactivity mapping to characterize the transcriptomes, proteomes and allergomes of *D*. *farinae* and *D*. *pteronyssinus* (Fig 1). To this end, mRNAs were sequenced using 454 and Illumina sequencing technologies, then *de novo* assembled, to yield 161,429 and 156,174 contigs, respectively. After subsequent detection of likely coding regions, translation and annotation, *D*. *farinae* and *D*. *pteronyssinus*-specific protein sequence databases were populated with 37,585 and 143,369 entries (out of which 21,696 and 79,890 were annotated), respectively, for each of the mite species.

**Fig 2:**
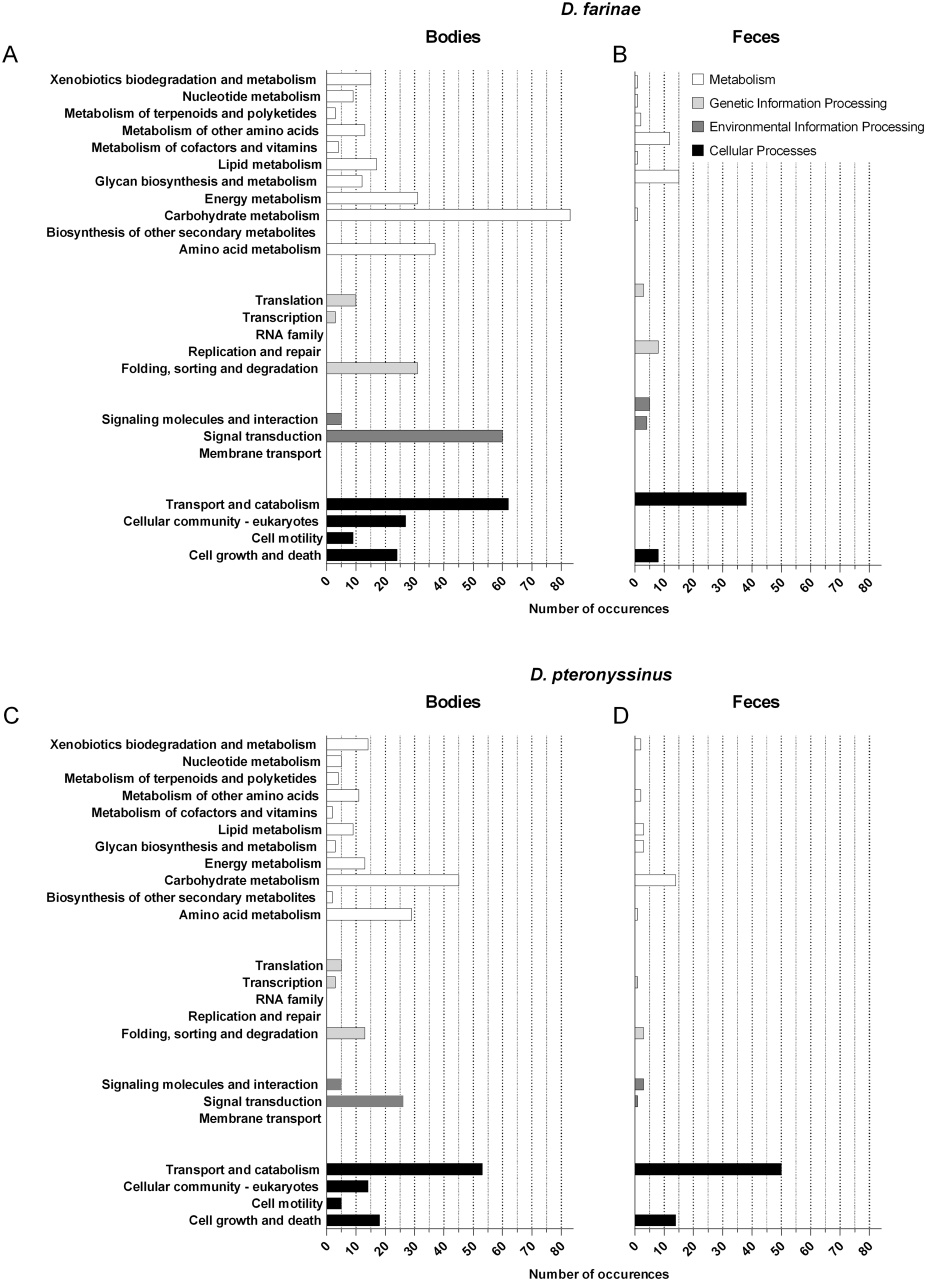
Functional analysis of proteomes from HDM bodies and feces. Proteins identified by LC-MS/MS in fractionated extracts from either body (left panel) or feces (right panel) were classified into functional categories using the KAAS server (details of protein identification are provided in supplementary S2 and S3 Tables). The histograms denote the numbers of occurrences of KEGG Orthology (KO) annotations for each of *D*. *farinae* (A, B) and *D*. *pteronyssinus* (C, D) species, assembled in functional categories.

We then characterized the protein content of each of the HDM extracts, using species-specific transcriptome-derived datasets as search databases for MS-based identification of peptides. To this end, aqueous protein extracts were generated from either bodies or fecal particles purified from whole cultures and subsequently analyzed by LC-MS/MS analysis after tryptic digestion. A total of 326 and 201 proteins were detected with a minimum of 2 supporting peptides, in extracts from purified *D*. *farinae* and *D*. *pteronyssinus* bodies, respectively. In fecal pellets from each of the two species, 80 and 77 proteins, respectively, were detected using the same criteria (supplementary S1 and S2 Tables). A functional annotation on these subsets of proteins was subsequently performed using the KEGG annotation server. Many of these proteins were categorized within well-known functional groups, related to either general metabolism, genetic information processing or cellular processes (Fig 2).

### Comprehensive characterization of *D*. *farinae* and *D*. *pteronyssinus* allergomes

We confirmed the presence in our transcriptome-derived databases of amino acid sequences corresponding to all HDM allergens currently registered by IUIS with supporting sequence data (*i*. *e*. Der f 1 to 4, Der f 6 to 8, Der f 10, Der f 11, Der f 13 to 16, Der f 18, Der f 20 to 22, Der f 24 to 35 as well as Der p 1 to 11, Der p 13 to 15, Der p 18, Der p 20, Der p 21, Der p 23, Der p 24). In addition, when mining the transcriptome-derived databases, we identified on the basis of high sequence similarities (≥71.2% in pairwise alignment) putative *D*. *pteronyssinus* counterparts of Der f 16, Der f 22, and Der f 25 to 35 (supplementary S3 Table). We also identified partial sequences of *D*. *farinae* counterparts of Der p 5, Der p 9 and Der p 23 (supplementary S3 Table). No sequence related to group 12 and 19 allergens associated with storage mites were detected. Interestingly, we also identified sequences related to families encompassing previously reported allergens in non-mite sources, such as enolases and phospholipases [34].

**Fig 3:**
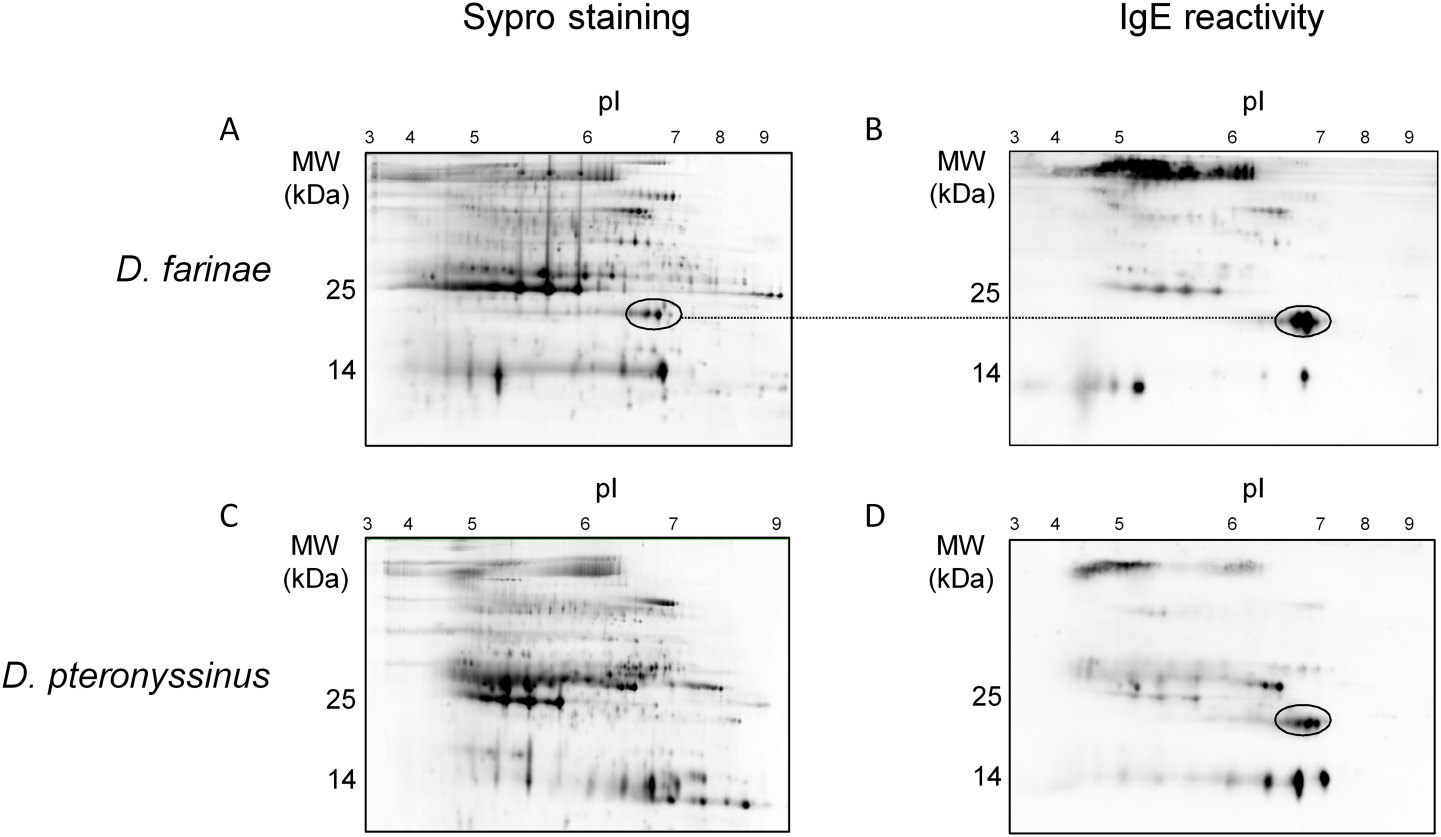
Proteome and IgE reactivity maps of *Dermatophagoides* species. Water-soluble HDM proteins were separated by 2D-gel electrophoresis and stained with Sypro Ruby or probed with a pool of serum IgEs from HDM-sensitized donors to establish 2D proteome (left panel) and IgE reactivity (right panel) maps for *D*. *farinae* (A, B) and *D*. *pteronyssinus* (C, D) species. Protein spots were picked and analyzed by LC-MS/MS after trypsin digestion, using the transcriptome-derived protein sequence collection supplemented with allergen sequences registered in the WHO/IUIS allergen database in order to facilitate identification. Spots corresponding to novel allergens (*i*. *e*. Der f 36 and Der p 36) are circled.

To further characterize HDM proteomes and allergomes, proteins from aqueous extracts of either *D*. *farinae* or *D*. *pteronyssinus* whole cultures were fractionated using high-resolution 2D-electrophoresis, prior to probing with a pool of sera from HDM-allergic donors (Fig 3). A total of 94 (out of which 63-IgE reactive) and 99 (out of which 50 IgE-reactive) gel plugs were recovered for *D*. *farinae* and *D*. *pteronyssinus* extracts, respectively, prior to LC-MS/MS analysis. Results of protein identification are provided in the online repository (supplementary S1 and S2 Figs, S4 and S5 Tables). Among the registered allergens 16 out of 30 (namely Der f 1 to 4, Der f 6, Der f 8, Der f 14, Der f 15, Der f 18, Der f 20, Der f 22, Der f 25, Der f 27, Der f 28, Der f 34 and Der f 35) and 9 out of 19 (namely Der p 1 to 4, Der p 6, Der p 8, Der p 9, Der p 15 and Der p 18) were detected in at least one spot for *D*. *farinae* and *D*. *pteronyssinus*, respectively. Whereas we did not detect the other known allergens under those experimental conditions, we confirmed that the latter are all present in the *D*. *farinae* or *D*. *pteronyssinus* extracts obtained from mite bodies and/or feces [10], with the exception of Der f 17 (whose sequence is not currently publically available), Der f 24, Der p 13 and Der p 24.

### Characterization of Der f 36 and Der p 36 as novel allergens

**Fig 4:**
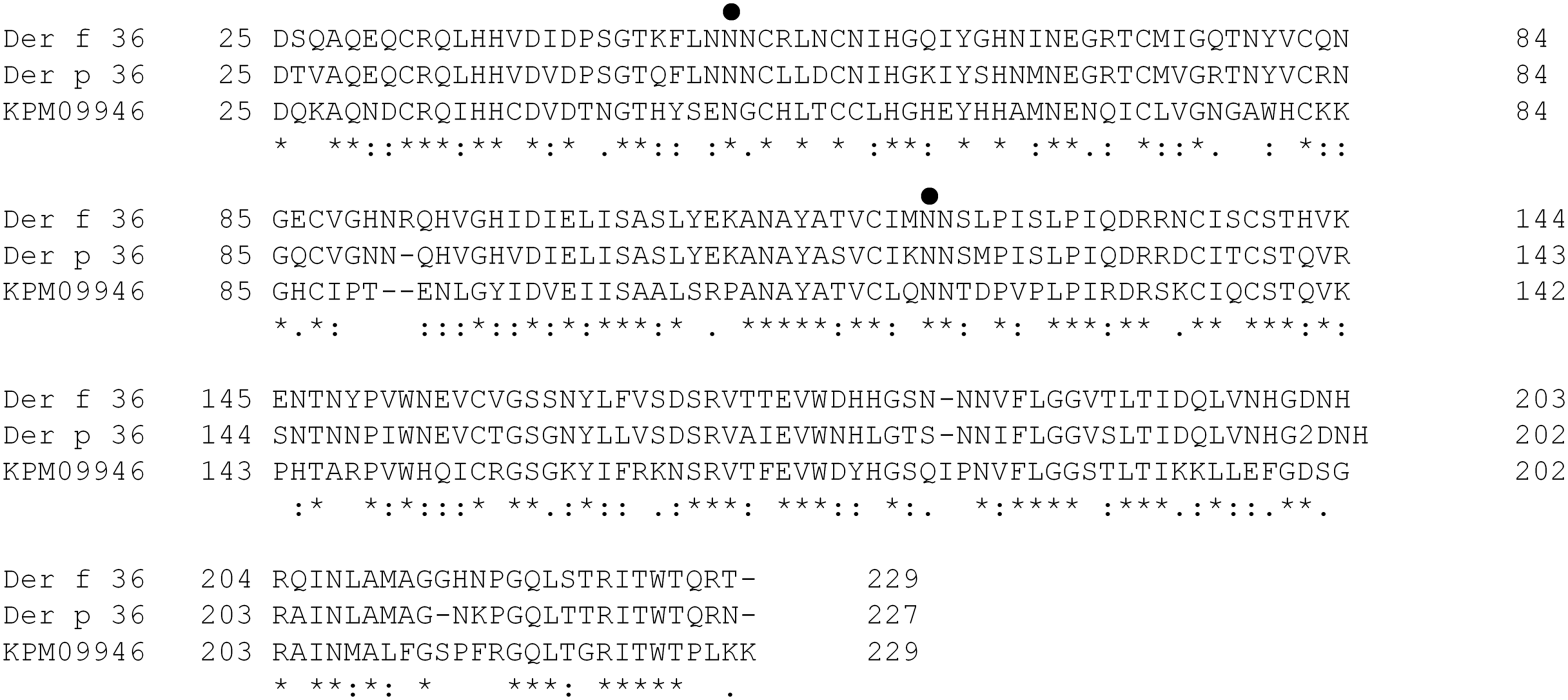
Primary sequence alignment of group 36 allergens. The amino acid sequences of Der f 36 and Der p 36 were aligned with the one of the putative KPM09946 protein *from S*. *scabei*. Putative N-glycosylation sites (●) in Der f 36 and Der p 36 are highlighted. The *,:,. symbols and empty space indicate identical, highly similar, similar or different amino acid, respectively. Residues are numbered relative to translation start.

Highly IgE-reactive alkaline spots were observed for both species on 2D-western blots, located within the 20–25 kDa / pI 6–7 range (Fig 3). The *D*. *farinae*-related protein spots (supplementary S1 Fig, spots #75–78) were picked after Sypro Ruby staining. MS/MS analysis revealed that these spots contain an undescribed protein displaying a 46.8% amino acid sequence identity in pairwise alignment with a putative protein from *Sarcoptes scabei* (KPM09946) [35] (Fig 4, supplementary S1 Table). Although the spots corresponding to a potential *D*. *pteronyssinus* counterpart were not detected on Sypro Ruby-stained 2D-gels, the presence of a highly similar molecule (79% amino acid identity) was nonetheless confirmed in the *D*. *pteronyssinus* extract by LC-MS/MS analysis (supplementary S1 Table). On the basis of the data presented below, these two molecules have been officially named and recorded as Der f 36 and Der p 36 in the IUIS allergen nomenclature. Further analyses confirmed that the two molecules are present in both body and feces extracts obtained from each of the two mite species (S1 and S2 Tables).

Nucleic coding sequences of Der f 36 and Der p 36 were retrieved from RNA sequencing data and shown to consist of 687 bp and 681 bp (Genbank KY465506 and KY465507 accession numbers) encoding 229- and 227- amino acid-long polypeptides, respectively. Importantly, the Der f 36 coding sequence was confirmed by alignment on the draft *D*. *farinae* genome [36], further indicating that this CDS is made of 3 exons. The inferred mature Der f 36 and Der p 36 polypeptides have both theoretical molecular masses of 22.7 kDa and isoelectric points of 6.6, in good agreement with our 2D-gel experimental data. Additionally, a N-terminal 24-amino acid signal sequence is predicted for both sequences.

**Fig 5:**
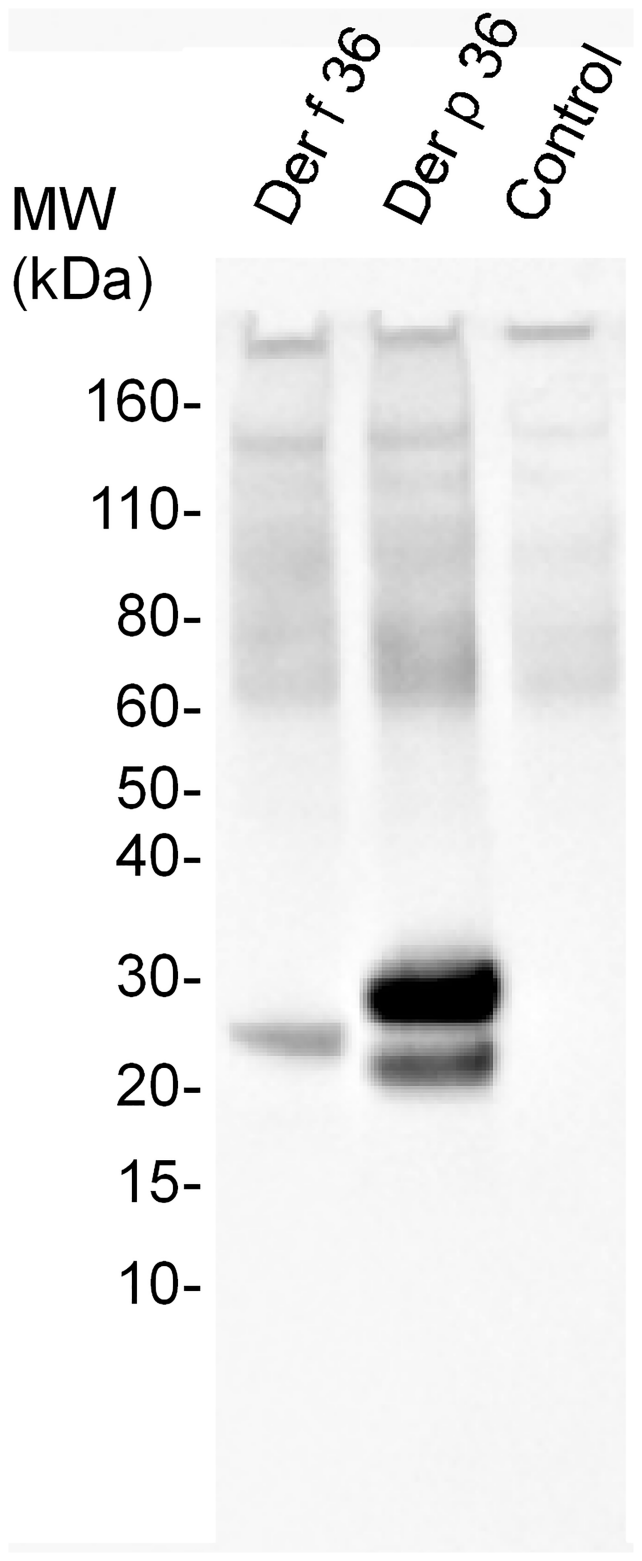
IgE reactivity of recombinant Der f 36 and Der p 36. Recombinant non-glycosylated Der f 36 and Der p 36 were produced in *P*. *pastoris*. IgE reactivity was assessed by western blot using a pool of sera from HDM-sensitized individuals. Culture supernatant from a mock strain was used as a negative control.

To confirm that Der f 36 and Der p 36 represent *bona fide* allergens, both mature molecules were produced as soluble secreted non-glycosylated variants in *P*. *pastoris*. Recombinant Der f 36 and Der p 36 molecules resolve as a single band and a doublet, respectively, following SDS-PAGE, likely due to differences in disulfide bridge distribution (data not shown). Importantly, both recombinant molecules were confirmed to react with IgEs from a pool of sera from HDM-sensitized individuals (Fig 5).

These recombinant non-glycosylated Der f 36 and Der p 36 allergens were also used to document the prevalence of IgE sensitization by western blot using individual plasma samples from 19 HDM-allergic patients exhibiting allergic rhinitis symptoms, with or without concomitant mild asthma. A similar IgE reactivity was observed for both molecules with 42% (8 out of 19 tested) of patients’ plasmas tested (Table 1).

## Discussion

*D*. *farinae* and *D*. *pteronyssinus* house dust mites are broadly distributed worldwide, and as such, they represent the main cause of respiratory allergies [3]. Frequent IgE sensitization to these two mite species can be in large part explained by the rich and complex pattern of allergens that they contain, encompassing as of today up to 33 different allergen groups officially recognized by IUIS. Whereas it is well established that groups 1 and 2 represent the main clinically relevant allergens, IgE reactivity profiles suggest that additional allergens remain to be identified [37]. Beyond the allergen content, a comprehensive molecular characterization of *D*. *farinae* and *D*. *pteronyssinus* extracts is also of major interest, since the latter are commonly used for diagnostic purposes but also as drug substances to treat HDM allergic patients by allergen immunotherapy [3, 38, 39]. Several studies conducted by us and others using MS-based analyses have shed some light on *Dermatophagoides* proteomes [23–25, 40, 41]. However, protein identification relying on sequence similarity searches using public databases has been consistently limited in those studies by the paucity of genomic or protein sequence data available.

In this context, we implemented herein a combined approach associating transcriptome sequencing, MS-based protein identification and IgE reactivity profiling in order to better document *Dermatophagoides* transcriptomes, proteomes and allergomes. We first generated a comprehensive inventory of transcripts by deep RNA sequencing, then inferred protein sequences to populate datasets encompassing 37,585 and 143,369 protein entries for *D*. *farinae* and *D*. *pteronyssinus*, respectively, in order to support MS-based protein identification. Approximately 75% of the transcripts assembled for *D*. *farinae* could be mapped to the recently published draft genome [36]. Failure to align the remaing transcripts could be explained *i*. *a*. by errors in *de novo* read assembly or by the absence of the corresponding regions in the draft genome. Furthermore, more than 55% of the inferred protein sequences were positively annotated by Blast analyses, thus confirming the accuracy of amino acid sequence prediction in those analyses. Collectively, more than 500 proteins have been identified in body or feces extracts from *D*. *farinae* and *D*. *pteronyssinus*, thus dramatically extending our knowledge of the proteomes of those two important mite species. A pathway analysis was performed on the latter dataset using KAAS server, revealing that many of the flagged proteins are related to general metabolism and cellular processes.

Focusing on allergens, our transcript databases comprise sequences corresponding to all known registered allergens with supporting sequencing data (*i*. *e*. Der f 1 to 4, Der f 6 to 8, Der f 10, Der f 11, Der f 13 to 16, Der f 18, Der f 20 to 22, Der f 24 to 35 and Der p 1 to 11, Der p 13 to 15, Der p 18, Der p 20, Der p 23, Der p 24). Among those, allergens from groups 5, 9 and 23 are officially registered in WHO/IUIS database only for *D*. *pteronyssinus*. We retrieved in our analyses partial *D*. *farinae* counterpart sequences which match those of Der f 5, Der f 9 and Der f 23 that have been deposited in Genbank. Likewise, groups 16, 22 and 25 to 35 are currently only documented in *D*. *farinae*. Importantly, we identified putative *D*. *pteronyssinus* counterparts to all these 13 allergens based on their high sequence similarity (≥71.2% identity) (supplementary S3 Table). Finally, as expected, we failed to detect in our analyses any sequence related to groups 12 and 19 allergens since the latter have previously been documented in storage mites and *Blomia tropicalis*, but are not reported in *Pyroglyphidae* mites [4, 8, 12, 36].

At a protein level, MS analyses after extract fractionation over 2D-gels led to the detection of Der f 1 to 4, Der f 6, Der f 8, Der f 14, Der f 15, Der f 18, Der f 20, Der f 22, Der f 25, Der f 27, Der f 28, Der f 34, Der f 35 as well as Der p 1 to 4, Der p 6, Der p 8, Der p 9, Der p 15, Der p 18 based on at least 2 specific peptides. In agreement with our previous study [10], these results confirm that most known allergens, including the recently registered Der f 4, Der f 34 and Der f 35 allergens, are present in our *Dermatophagoides* extracts. The presence of Der f 24, Der p 13 and Der p 24 could not be demonstrated using our analysis criteria. Minor discrepancies between in-gel and in-solution analyses in the present study (*e*. *g*. lack of detection of allergens from groups 7, 10 and 11) are likely explained by differences in sample preparation used for 2D-gel or MS analyses.

Our study also confirmed the presence in *Dermatophagoides* extracts of proteins with significant similarity with members of protein families known to encompass allergens, such as enolases or phospholipases, suggesting that additional allergens may be present in house dust mites [25, 34]. Consistent with this, we observed some IgE reactivity which could not be assigned to currently known mite allergens. Specifically, we identified α-glucosidases as high molecular mass candidate allergens eliciting IgEs in selected patients (supplementary S1 Fig, spots #9, #13–16; S2 Fig, spots #1–4, data not shown). The allergenicity of these molecules needs to be further investigated in dedicated studies.

Importantly, we also established that some 23 kDa proteins represent novel HDM group 36 mite allergens, present in both *D*. *farinae* and *D*. *pteronyssinus* species. Der f 36 and Der p 36 display a high amino acid sequence similarity (79% identity), and a 46% identity with the KPM0996 putative protein from the scabies mite *Sarcoptes scabei*. Whereas, the latter has a predicted C2 domain, it has no biochemical function ascribed as of today. Group 36 molecules do not display significant amino acid sequence similarity with known allergens, making Der f 36 and Der p 36 first-in-class allergens. Using recombinant molecules, we documented IgE reactivity towards group 36 molecules in 42% of HDM-allergic individuals, with no disconnect observed between Der f 36 and Der p 36, as expected given their high similarity. Although Der f 36 and Der p 36 harbor two putative N-glycosylation motifs, MS spectra failed to detect the presence of glycan moieties on the two natural molecules (data not shown). Importantly, non-glycosylated forms of recombinant Der f 36 and Der p 36 also bind IgE (Fig 5), confirming that cross-reactive carbohydrates are not responsible for the IgE reactivity of these *bona fide* allergens. The potency of group 36 allergens has not been assessed, however, given that (i) only 42% of HDM allergic patients exhibit IgE reactivity with such molecules, and (ii) 50–77% of IgE reactivity is commonly assigned to group 1 or group 2 allergens [3, 4, 9–11, 13–16], we conclude that Der f 36 and Der p 36 represent minor HDM allergens, even if they may be clinically relevant for selected mite-allergic patients.

In conclusion, our combined transcriptome and proteome analyses have yielded a comprehensive molecular characterization of *D*. *farinae* and *D*. *pteronyssinus* extracts, further expanding the known allergomes for these mite species to 34 groups of allergens.

## Supporting information

S1 Text Materials and methods. (DOCX)

S1 Fig 2D reference maps of the *D*. *farinae* proteome and allergome. (A) Proteins from a *D*. *farinae* whole culture extract were separated by 2D-gel electrophoresis and stained with Sypro Ruby. (B) IgE reactivity pattern using a pool of seric IgEs from HDM-sensitized individuals. IgE-reactive (red circles) and non IgE-reactive (green circles) spots were picked and analyzed by LC-MS/MS after trypsin digestion. Proteins were identified using the transcriptome derived sequence database supplemented with registered allergen sequences. Identification results are provided in supplementary S4 Table.
(TIF)

S2 Fig 2D reference maps of the *D*. *pteronyssinus* proteome and allergome. (A) Proteins from a *D*. *pteronyssinus* whole culture extract were separated by 2D-gel electrophoresis and stained with Sypro Ruby. (B) IgE reactivity pattern using a pool of seric IgEs from HDM-sensitized individuals. IgE-reactive (red circles) and non IgE-reactive (green circles) spots were picked and analyzed by LC-MS/MS after trypsin digestion. Proteins were identified using the transcriptome derived sequence database supplemented with registered allergen sequences. Identification results are provided in supplementary S5 Table.
(TIF)

S1 Table Overview of *D*. *farinae* body and fecal proteomes. Protein extracts from *D*. *farinae* fractionated bodies and fecal pellets were analyzed by LC-MS/MS. Protein identification was performed using the species-specific transcriptome derived protein database, supplemented with IUIS-registered allergen sequences, as reference dataset. Only entries identified by a minimum of 2 peptides sequenced were taken into account. Proteins are reported by the entry name with the total numbers of supporting mapping sequenced peptides (#peptides) and of uniquely mapping peptides (#unique) as well as, when available, the result of the annotation by blast analysis.
(PDF)

S2 Table Overview of *D*. *pteronyssinus* body and fecal proteomes. Protein extracts from *D*. *pteronyssinus* fractionated bodies and feces were analyzed by LC-MS/MS. Protein identification was performed using the species-specific transcriptome derived protein database, supplemented with IUIS-registered allergen sequences, as reference dataset. Only entries identified by a minimum of 2 peptides sequenced were taken into account. Proteins are reported by the entry name with the total numbers of supporting sequenced peptides (#peptides) and of uniquely mapping peptides (#unique) as well as, when available, the result of the annotation by blast analysis.
(PDF)

S3 Table Counterparts of registered allergens. Counterparts in either *D*. *farinae* or *D*. *pteronyssinus* of IUIS-recorded allergens were identified in transcriptome-derived databases using blast analyses.
(PDF)

S4 Table Analysis of *D*. *farinae* proteome after 2D electrophoresis. A protein extract from whole *D*. *farinae* culture was submitted to two-dimensional gel electrophoresis. After staining with Sypro Ruby, gel plugs from 94 protein spots (S1 Fig) were recovered, trypsin digested then analyzed by LC-MS/MS. Numbers refer to the IgE-reactive (red cell) and non IgE-reactive (green cell) spots analyzed. Protein identification was performed using the species-specific transcriptome derived protein database, supplemented with IUIS-registered allergen sequences, as reference dataset. Only entries identified by a minimum of 2 peptides sequenced were taken into account. Proteins are reported by the entry name with the total numbers of supporting sequenced peptides (#peptides) and of uniquely mapping peptides (#unique) as well as, when available, the result of the annotation by blast analysis.
(PDF)

S5 Table Analysis of *D*. *pteronyssinus* proteome after 2D electrophoresis. A protein extract from whole *D*. *pteronyssinus* culture was submitted to two-dimensional gel electrophoresis. After staining with Sypro Ruby, gel plugs from 99 protein spots (S2 Fig) were recovered, trypsin digested then analyzed by LC-MS/MS. Numbers refer to the IgE-reactive (red cell) and non IgE-reactive (green cell) spots analyzed. Protein identification was performed using the species-specific transcriptome derived protein database, supplemented with IUIS-registered allergen sequences, as reference dataset. Only entries identified by a minimum of 2 peptides sequenced were taken into account. Proteins are reported by the entry name with the total numbers of supporting sequenced peptides (#peptides) and of uniquely mapping peptides (#unique) as well as, when available, the result of the annotation by blast analysis.
(PDF)
