# Supplementary material for: A combined transcriptome and proteome analysis extends the allergome of house dust mite Dermatophagoides species
Source: PLoS One. 2017 Oct 5;12(10):e0185830. doi: 10.1371/journal.pone.0185830 (PMC5628879; doi:10.1371/journal.pone.0185830)
Supplement: S1 Text — (DOCX) [file pone.0185830.s001.docx]

**Supporting Information**

**Supplementary Material and Methods**

**In-solution mass spectrometry (MS) analyses**

Proteins (5 µg) were denatured with 0.5 % (w/v) RapiGest SF (Waters, Guyancourt, France) and disulfide bonds were reduced using dithiothreitol and alkylated by iodoacetamide. Trypsin digestion was performed at 37°C for 3 h using a trypsin:protein ratio of 1:25. Digested samples (0.5-1 µg of proteins) were loaded onto an Acclaim PepMap 100 C_18_ Trap column (Thermo Fisher Scientific, Saint-Quentin-en-Yvelines, France) and separated at 50°C using an Acclaim PepMap RSLC C_18_ column (2 µm, 100 Å, 75 µm x 50 cm, Thermo Fisher Scientific). Peptides were eluted at a 300 nL/min flow rate using a 200-min gradient of 0.1 % formic acid / 80% acetonitrile 0.1 % formic acid. The column eluent was introduced into an Impact HD QqToF mass spectrometer equipped with a CaptiveSpray source (Bruker Daltonics, Wissembourg, France). Analyses were performed in the positive ion mode using capillary and end plate offset voltages of -1300 V and -500 V, respectively. The drier temperature was set at 180°C. Data were acquired by the ToF analyzer from m/z 50 to 2000. MS/MS spectra were acquired in an intensity dependent mode at a rate of 2-16 Hz in the range of m/z 300‑1800 (charge state >2) and a MS spectra rate of 2 Hz. Mass calibration was performed both externally, using the ESI-L Low Concentration Tuning Mix (Agilent Technologies, Les Ulis, France), and internally, using the lock mass option in MS mode: m/z 1221.9906 (Chip cube high mass reference, Agilent Technologies).

Peptide identification was performed using the PEAKS software version 8 (Bioinformatics Solutions Inc., Waterloo, Canada) with the species-specific transcriptome-derived databases, supplemented with the sequences of IUIS‑registered allergens, as reference datasets (encompassing 37,640 and 143,430 entries for *D. farinae* and *D. pteronyssinus*, respectively). Database search was performed using the following parameters: peptide mass tolerance of 10 ppm (0.05 Da for fragment ions), trypsin enzyme, one non-specific cleavage at both ends of the peptide allowed, three missed cleavages permitted, carbamidomethylation of cysteine as a fixed modification, deamidation of glutamine/asparagine and oxidation of methionine as variable modifications, allowing a maximum of 5 variable post‑translational modifications per peptide. Protein identifications were confirmed with the application of a 0.1% peptide-spectrum matches false discovery rate (estimated with the decoy fusion method).

**In-gel MS analyses**

HDM extracts were submitted to two‑dimensional electrophoresis. Proteins were precipitated using TCA/acetone and resuspended in a 7 M urea, 2 M thiourea, 4% CHAPS and 30 mM Tris pH 8.8 buffer before 2D-gel electrophoresis using 3–10 non linear pH range 12.5% DALT gels (GE Healthcare, Velizy-Villacoublay, France), as per the manufacturers’ instructions. After staining with Sypro Ruby, gel pieces were excised using an EXquest spot cutter (Bio-Rad, Marnes-La–Coquette, France) then destained with a 50% acetonitrile / 50 mM ammonium bicarbonate solution and dehydrated using acetonitrile. Next, gel pieces were rehydrated with 5 µL of a 2 ng/µL trypsin solution prepared in 50 mM ammonium bicarbonate 0.01% (w/v) ProteaseMax (Promega, Lyon, France) digestion solution. After a 10 min incubation at room temperature, 40 µL of digestion solution were added. After a 1h45 incubation at 37°C, the enzymatic digestion was quenched by adding 10 µL of a 5% formic acid solution. Digests were analyzed by reversed-phase liquid chromatography using an Ultimate 3000 RS nano LC system (Thermo Fisher Scientific) coupled to a mass spectrometer. Digested samples were loaded onto an Acclaim PepMap 100 C_18_ Trap column (Thermo Scientific) and separated at 50°C at a flow rate of 450 nL/min using an Acclaim PepMap RSLC C_18_ column (2 µm, 100 Å, 75 µm x 15 cm, Thermo Scientific) using a 45 min gradient of 0.1% formic acid / 80 % acetonitrile 0.1 % formic acid. Eluted peptides were then introduced into an impact HD QqToF mass spectrometer with CaptiveSpray source (Brucker Daltonics). Acquisitions were performed in positive mode with capillary voltage set at - 1,400 V. MS spectra were acquired over the 110‑2,000 m/z range with a scan rate of 2 Hz. Acquisitions were performed using the Intensity Dependent Acquisition Speed (IDAS) mode with a MS-MS/MS cycle time fixed at 2 s and MS/MS acquisition speed varying from 2 to 8 Hz depending on precursor intensity. Peptide identification was performed using PEAKS software as described above but neither non-specific cleavage nor variable modification were allowed.
