# Supplementary figures and images for: A combined transcriptome and proteome analysis extends the allergome of house dust mite Dermatophagoides species
Source: PLoS One. 2017 Oct 5;12(10):e0185830. doi: 10.1371/journal.pone.0185830 (PMC5628879; doi:10.1371/journal.pone.0185830)

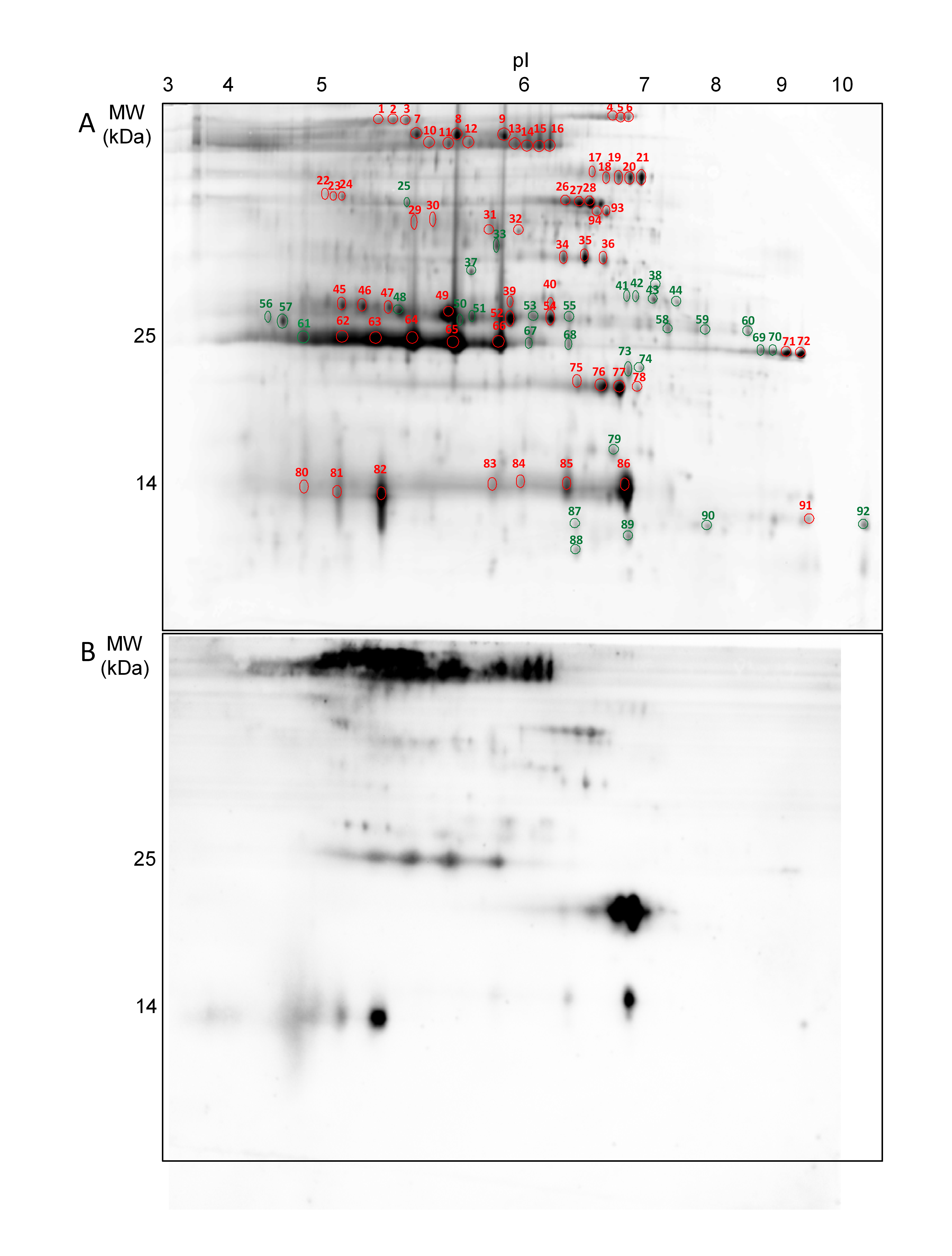

Supplement: S1 Fig — (A) Proteins from a D. farinae whole culture extract were separated by 2D-gel electrophoresis and stained with Sypro Ruby. (B) IgE reactivity pattern using a pool of seric IgEs from HDM-sensitized individuals. IgE-reactive (red circles) and non IgE-reactive (green circles) spots were picked and analyzed by LC-MS/MS after trypsin digestion. Proteins were identified using the transcriptome derived sequence database supplemented with registered allergen sequences. Identification results are provided in supplementary S4 Table. (TIF) [file pone.0185830.s002.tif]

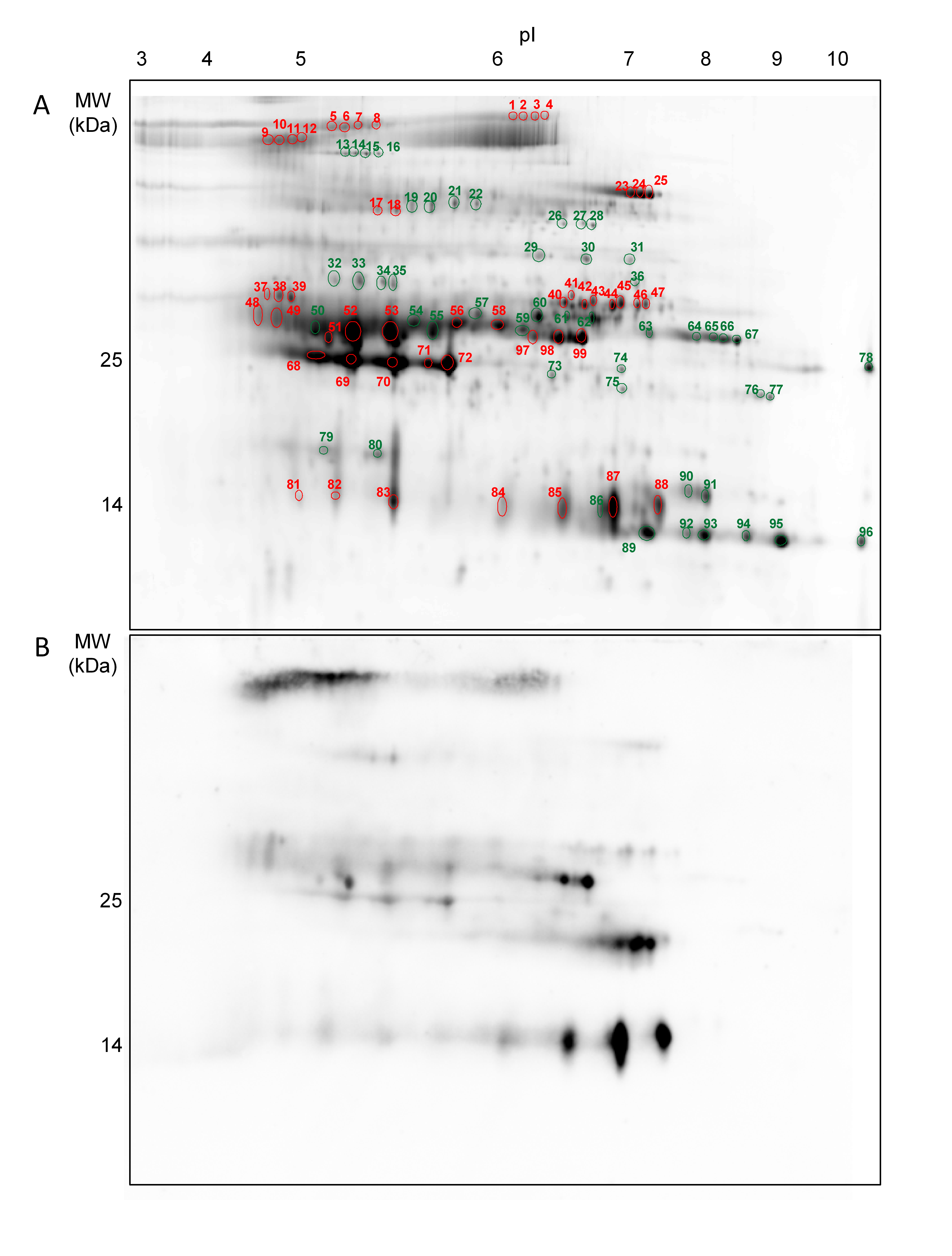

Supplement: S2 Fig — (A) Proteins from a D. pteronyssinus whole culture extract were separated by 2D-gel electrophoresis and stained with Sypro Ruby. (B) IgE reactivity pattern using a pool of seric IgEs from HDM-sensitized individuals. IgE-reactive (red circles) and non IgE-reactive (green circles) spots were picked and analyzed by LC-MS/MS after trypsin digestion. Proteins were identified using the transcriptome derived sequence database supplemented with registered allergen sequences. Identification results are provided in supplementary S5 Table. (TIF) [file pone.0185830.s003.tif]
