# Supplementary material for: A combined transcriptome and proteome analysis extends the allergome of house dust mite Dermatophagoides species
Source: PLoS One. 2017 Oct 5;12(10):e0185830. doi: 10.1371/journal.pone.0185830 (PMC5628879; doi:10.1371/journal.pone.0185830)
Supplement: S1 Table — Protein extracts from D. farinae fractionated bodies and fecal pellets were analyzed by LC-MS/MS. Protein identification was performed using the species-specific transcriptome derived protein database, supplemented with IUIS-registered allergen sequences, as reference dataset. Only entries identified by a minimum of 2 peptides sequenced were taken into account. Proteins are reported by the entry name with the total numbers of supporting mapping sequenced peptides (#peptides) and of uniquely mapping peptides (#unique) as well as, when available, the result of the annotation by blast analysis. (PDF) [file pone.0185830.s004.pdf]

Bodies/feces	Accession	#Peptides	#Unique	Description
Bodies	Der f 10.0101	49	49	Der f 10.0101
Bodies	Der f 11.0101	38	0	Der f 11.0101
Bodies	cds.comp43197_c0_seq1|m.10342	36	35	B2MVM3_SARSC | Actin
Bodies	cds.comp98587_c0_seq1|m.25206	31	30	Q2HXW9_BLAGE | Enolase
Bodies	Der f 20.0101	27	23	Der f 20.0101
Bodies	cds.comp111369_c0_seq1|m.35926	26	26	Q9U785_EURMA | High molecular weight allergen M-177 Flags: Precursor
Bodies	cds.comp70079_c0_seq1|m.16663	25	0	B2CQL3_9ACAR | Paramyosin
Bodies	cds.comp44977_c0_seq1|m.10822	22	22	I4DIT1_PAPXU | Similar to CG7031
Bodies	cds.comp96053_c0_seq1|m.22817	22	22	K7J833_NASVI | Malate dehydrogenase EC=1.1.1.37
Bodies	cds.comp26781_c0_seq1|m.6407	22	22	B7QE46_IXOSC | ATP synthase subunit beta EC=3.6.3.14
Bodies	cds.comp55665_c0_seq1|m.13512	21	21	A1KY40_BLOTA | Major abundant protein BTP1
Bodies	Der f 2.0103	20	2	Der f 2.0103
Bodies	Der f 2.0102	19	1	Der f 2.0102
Bodies	Der f 26.0101	19	19	Der f 26.0101
Bodies	cds.comp96183_c0_seq1|m.22947	18	18	D7URK4_SARSC | Myosin heavy chain Flags: Fragment
Bodies	cds.comp70080_c0_seq1|m.16667	17	4	Q6Y2F9_DERPT | HDM allergen
Bodies	cds.comp100593_c0_seq1|m.27383	17	17	F1CJ03_HOTJU | Putative muscular protein Flags: Fragment
Bodies	cds.comp98584_c0_seq1|m.25201	16	16	no hit
Bodies	Der f 1.0107	15	6	Der f 1.0107

Bodies/feces	Accession	#Peptides	#Unique	Description
Bodies	cds.comp103285_c0_seq1|m.29677	15	15	A1KXG7_DERFA | Der f 7 allergen
Bodies	Der f 15.0101	15	15	Der f 15.0101
Bodies	cds.comp127448_c0_seq1|m.44235	15	15	G3MT41_9ACAR | ATP synthase subunit alpha
Bodies	cds.comp94136_c0_seq1|m.21929	15	15	Q16FX9_AEDAE | 4-hydroxyphenylpyruvate dioxygenase
Bodies	cds.comp121486_c0_seq1|m.41772	14	14	A1KY40_BLOTA | Major abundant protein BTP1
Bodies	cds.comp7297_c0_seq1|m.1766	14	14	E2A599_CAMFO | Lysosomal alpha-glucosidase
Bodies	cds.comp127400_c0_seq1|m.44186	14	14	A1KY40_BLOTA | Major abundant protein BTP1
Bodies	cds.comp14640_c0_seq1|m.3489	14	14	E1ZW00_CAMFO | Myosin heavy chain muscle
Bodies	cds.comp128657_c0_seq1|m.44900	14	14	no hit
Bodies	cds.comp7296_c0_seq1|m.1765	13	13	E2BEF1_HARSA | Lysosomal alpha-glucosidase Flags: Fragment
Bodies	Der f 13.0101	13	13	Der f 13.0101
Bodies	cds.comp127978_c0_seq1|m.44503	13	13	B7QHI5_IXOSC | Putative uncharacterized protein
Bodies	Der f 20.0201	13	9	Der f 20.0201
Bodies	cds.comp111369_c0_seq1|m.35927	12	12	Q9U785_EURMA | High molecular weight allergen M-177 Flags: Precursor
Bodies	cds.comp78532_c0_seq1|m.18484	12	12	A1KXH8_DERFA | Der f Gal d 2 allergen
Bodies	cds.comp80154_c0_seq1|m.18914	12	12	Der f 35
Bodies	cds.comp39978_c0_seq1|m.9575	12	2	I4DIT1_PAPXU | Similar to CG7031
Bodies	cds.comp106293_c0_seq1|m.32453	12	12	B7QIG6_IXOSC | Secreted salivary gland peptide putative
Bodies	cds.comp143469_c0_seq1|m.49723	12	12	B0KZL1_TYRPU | Mite allergen Tyr p 7

Bodies/feces	Accession	#Peptides	#Unique	Description
Bodies	Der f 25.0201	12	12	Der f 25.0201
Bodies	cds.comp113999_c0_seq1|m.37857	12	12	B7PBS2_IXOSC | Structural constituent of cuticle putative
Bodies	cds.comp108058_c0_seq1|m.33824	12	12	F5HJZ1_ANOGA | AGAP000801-PB Flags: Fragment
Bodies	Der f 16.0101	12	7	Der f 16.0101
Bodies	cds.comp83879_c0_seq1|m.19673	12	12	R4G3J7_RHOPR | Putative gamma interferon inducible lysosomal thiol reductase gilt
Bodies	cds.comp117839_c0_seq1|m.39927	12	12	G6CYG8_DANPL | Phosphoribosylaminoimidazole carboxylase phosphoribosylaminoimidazole succinocarboxamide synthetase
Bodies	cds.comp114970_c0_seq1|m.38343	11	11	Der f 36
Bodies	Der f 7.0101	11	11	Der f 7.0101
Bodies	cds.comp88672_c0_seq1|m.20734	11	11	D2DGW3_TYRPU | Troponin C
Bodies	cds.comp5505_c0_seq1|m.1316	11	11	Q6QWP0_9ARAC | Glyceraldehyde-3-phosphate dehydrogenase EC=1.2.1.12 Flags: Fragment
Bodies	cds.comp96501_c0_seq1|m.23237	11	11	A8E4K0_9ACAR | Troponin T
Bodies	cds.comp34142_c0_seq1|m.8165	11	11	B7Q0R0_IXOSC | Phosphoglycerate mutase putative EC=5.4.2.1
Bodies	cds.comp23030_c0_seq1|m.5492	11	11	I1ZCC6_DERFA | Elongation factor 1-alpha Flags: Fragment
Bodies	cds.comp108273_c0_seq1|m.34021	11	11	Q0KKA6_HAELO | Leucine aminopeptidase
Bodies	cds.comp102386_c0_seq1|m.28694	11	11	A1KXH7_DERFA | Der f Alt a 10 allergen
Bodies	cds.comp96034_c0_seq1|m.22500	10	0	no hit
Bodies	cds.comp100545_c0_seq1|m.27337	10	10	Q1HQT8_AEDAE | AAEL007549-PA SubName: Full=Phosphatidylethanolamine-binding protein
Bodies	cds.comp140939_c0_seq1|m.49097	10	10	Q5TS83_ANOGA | AGAP008584-PA
Bodies	cds.comp103290_c0_seq1|m.29682	10	10	E2BBG1_HARSA | Filamin-C

Bodies/feces	Accession	#Peptides	#Unique	Description
Bodies	cds.comp57824_c0_seq1|m.14037	10	10	Q1M0Y1_BLOTA | Blo t allergen isoform 2
Bodies	Der f 4.0101	10	10	Der f 4.0101
Bodies	cds.comp105707_c0_seq1|m.31981	10	10	F4WQI8_ACREC | Actin-interacting protein 1
Bodies	cds.comp39311_c0_seq1|m.9404	10	10	F4W8Y5_ACREC | Lysosomal alpha-mannosidase
Bodies	cds.comp103780_c0_seq1|m.30108	10	10	no hit
Bodies	cds.comp128016_c0_seq1|m.44537	10	0	B7P3P1_IXOSC | Cathepsin B endopeptidase putative EC=3.4.22.1
Bodies	Der f 32.0101	10	10	Der f 32.0101
Bodies	cds.comp99320_c0_seq1|m.26025	9	0	A1KXH2_DERFA | Der f 1 allergen
Bodies	cds.comp114791_c0_seq1|m.38248	9	9	no hit
Bodies	cds.comp101423_c0_seq1|m.28025	9	2	A1KXH3_DERFA | Der f 3 allergen
Bodies	cds.comp89610_c0_seq1|m.20924	9	9	B7QNY0_IXOSC | Serpin-2 putative EC=2.7.11.1
Bodies	Der f 28.0101	9	6	Der f 28.0101
Bodies	cds.comp118120_c0_seq1|m.40093	9	9	L7UZ91_DERFA | Ferritin
Bodies	cds.comp96086_c0_seq1|m.22836	9	9	Q09JE3_ARGMO | Superoxide dismutase [Cu-Zn] EC=1.15.1.1
Bodies	cds.comp100364_c0_seq1|m.27163	9	9	Q8MRY3_DROME | SD13780p
Bodies	cds.comp101559_c0_seq1|m.28081	9	9	B7Q0D4_IXOSC | Fumarylacetoacetase putative EC=3.7.1.2
Bodies	Der f 31.0101	9	9	Der f 31.0101
Bodies	cds.comp113406_c0_seq1|m.37483	9	9	D2A5H8_TRICA | Phosphoglycerate kinase EC=2.7.2.3
Bodies	cds.comp104802_c0_seq1|m.31198	9	9	B7PXR5_IXOSC | Chaperonin complex component TCP-1 eta subunit putative

Bodies/feces	Accession	#Peptides	#Unique	Description
Bodies	cds.comp52788_c0_seq1|m.12723	9	9	L7MFY3_9ACAR | Putative thioredoxin peroxidase Flags: Fragment
Bodies	cds.comp43198_c0_seq1|m.10343	8	8	B2MVM3_SARSC | Actin
Bodies	cds.comp147426_c0_seq1|m.50581	8	8	Q8N0N0_DERPT | Group 14 allergen protein Flags: Fragment
Bodies	cds.comp37783_c0_seq1|m.9037	8	8	B7Q6Z1_IXOSC | Saposin putative EC=3.4.23.40
Bodies	cds.comp79186_c0_seq1|m.18649	8	8	H9KR11_APIME | Fructose-bisphosphate aldolase EC=4.1.2.13
Bodies	Der f 3.0101	8	1	Der f 3.0101
Bodies	Der f 22.0101	8	8	Der f 22.0101
Bodies	cds.comp103519_c0_seq1|m.29899	8	8	Q66RP5_TYRPU | Fatty acid-biding protein
Bodies	cds.comp33654_c0_seq1|m.8020	8	8	A1YW13_DERFA | Der f 1 allergen
Bodies	cds.comp99077_c0_seq1|m.25746	8	8	Q8MWR6_DERPT | 14.5 kDa bacteriolytic enzyme
Bodies	cds.comp310_c0_seq1|m.148	8	8	L7M5B4_9ACAR | Putative 3-hydroxyacyl-coa dehydrogenase
Bodies	cds.comp107551_c0_seq1|m.33365	8	8	L7M7S5_9ACAR | Putative dihydropteridine reductase dhpr/qdpr
Bodies	cds.comp14554_c0_seq1|m.3464	8	8	A0SHR2_AMBVA | Protein disulfide isomerase EC=5.3.4.1
Bodies	cds.comp80633_c0_seq1|m.19033	8	8	R4G8F0_RHOPR | Putative adenosine kinase
Bodies	cds.comp3629_c0_seq1|m.877	8	8	L7UZ91_DERFA | Ferritin
Bodies	cds.comp45957_c0_seq1|m.11079	8	8	B7QMV1_IXOSC | Elongation factor putative
Bodies	cds.comp58863_c0_seq1|m.14267	8	8	B7QC64_IXOSC | Glutathione S-transferase kappa putative EC=2.5.1.18
Bodies	cds.comp104026_c0_seq1|m.30379	8	8	no hit
Bodies	cds.comp128015_c0_seq1|m.44536	8	1	B7P3P1_IXOSC | Cathepsin B endopeptidase putative EC=3.4.22.1

Bodies/feces	Accession	#Peptides	#Unique	Description
Bodies	cds.comp99344_c0_seq1|m.26058	8	8	L7M2J0_9ACAR | Putative aminopeptidase of the m17 family
Bodies	Der f 8.0101	8	8	Der f 8.0101
Bodies	cds.comp51865_c0_seq1|m.12508	8	8	G6DFH3_DANPL | Tubulin beta 2C
Bodies	cds.comp86984_c0_seq1|m.20365	7	7	E9IA80_SOLIN | Fructose-bisphosphate aldolase EC=4.1.2.13 Flags: Fragment
Bodies	cds.comp39747_c0_seq1|m.9504	7	7	E7D199_LATHE | Glyceraldehyde-3-phosphate dehydrogenase EC=1.2.1.12 Flags: Fragment
Bodies	cds.comp15303_c0_seq1|m.3655	7	6	G3ACU4_GLOMR | Actin 5C/42A Flags: Fragment
Bodies	cds.comp117913_c0_seq1|m.39965	7	7	B7Q5N1_IXOSC | Cuticle protein putative
Bodies	cds.comp99614_c0_seq1|m.26339	7	7	A1KXC1_DERFA | DFP1
Bodies	cds.comp50051_c0_seq1|m.12079	7	7	B7QHI5_IXOSC | Putative uncharacterized protein
Bodies	cds.comp67650_c0_seq1|m.16137	7	7	I4DKD3_PAPXU | Malate dehydrogenase EC=1.1.1.37
Bodies	cds.comp99402_c0_seq1|m.26132	7	7	B7PXR5_IXOSC | Chaperonin complex component TCP-1 eta subunit putative
Bodies	cds.comp17779_c0_seq1|m.4228	7	7	A3EXM6_MACHI | Putative 60 kDa heat shock protein Flags: Fragment
Bodies	cds.comp19576_c0_seq1|m.4639	7	7	Q3Y596_MACRS | Superoxide dismutase EC=1.15.1.1
Bodies	cds.comp47365_c0_seq1|m.11413	7	7	B7P1Q2_IXOSC | Myosin heavy chain skeletal muscle or cardiac muscle putative EC=1.3.1.74
Bodies	cds.comp121851_c0_seq1|m.41954	7	7	B7P3M8_IXOSC | D-3-phosphoglycerate dehydrogenase putative EC=1.1.1.95 Flags: Fragment
Bodies	cds.comp102705_c0_seq1|m.28983	6	6	no hit
Bodies	cds.comp128012_c0_seq1|m.44522	6	4	B7P3P0_IXOSC | Cathepsin B endopeptidase putative EC=3.4.22.1
Bodies	cds.comp19387_c0_seq1|m.4597	6	6	A8B8I1_DERFA | Der f 5 allergen SubName: Full=Group 5 allergen
Bodies	cds.comp104985_c0_seq1|m.31342	6	6	no hit

Bodies/feces	Accession	#Peptides	#Unique	Description
Bodies	cds.comp96429_c0_seq1|m.23153	6	1	Q8MVU3_DERFA | Gelsolin-like allergen Der f 16
Bodies	cds.comp44282_c0_seq1|m.10627	6	6	no hit
Bodies	Der f 6.0101	6	3	Der f 6.0101
Bodies	cds.comp14359_c0_seq1|m.3415	6	6	J7FXP5_LATHE | Uncharacterized protein
Bodies	Der f 29.0101	6	4	Der f 29.0101
Bodies	cds.comp137944_c0_seq1|m.48198	6	6	B7PLR3_IXOSC | Filamin-C putative EC=3.1.3.48
Bodies	cds.comp104770_c0_seq1|m.31149	6	6	B5DS37_DROPS | GA27615
Bodies	cds.comp45557_c0_seq1|m.11005	6	6	no hit
Bodies	cds.comp52972_c0_seq1|m.12765	6	6	Q2XW13_RHIMP | Glutathione peroxidase
Bodies	cds.comp6133_c0_seq1|m.1459	6	6	L7MAZ4_9ACAR | Putative enoyl-coa hydratase
Bodies	cds.comp109979_c0_seq1|m.35071	6	6	Q95PA6_AEDAE | Putative short-chain dehydrogenase/reductase Mc1
Bodies	cds.comp103689_c0_seq1|m.30038	6	6	E9HAY5_DAPPU | Putative uncharacterized protein
Bodies	cds.comp113924_c0_seq1|m.37808	6	6	L7MGB9_9ACAR | Putative aicar transformylase/imp cyclohydrolase/methylglyoxal synthase Flags: Fragment
Bodies	cds.comp30058_c0_seq1|m.7182	5	5	C6SUZ2_DROME | AT15141p Flags: Fragment
Bodies	cds.comp42566_c0_seq1|m.10197	5	5	F4WWF5_ACREC | Calreticulin Flags: Fragment
Bodies	cds.comp102933_c0_seq1|m.29262	5	5	no hit
Bodies	cds.comp149823_c0_seq1|m.51085	5	3	M4LIT4_COTVE | Heat shock protein 70
Bodies	cds.comp99586_c0_seq1|m.26318	5	5	A1KY40_BLOTA | Major abundant protein BTP1
Bodies	cds.comp39309_c0_seq1|m.9403	5	5	E2ABS8_CAMFO | Lysosomal alpha-mannosidase

Bodies/feces	Accession	#Peptides	#Unique	Description
Bodies	cds.comp112368_c0_seq1|m.36667	5	2	Q155V8_DERFA | Der f 6 Flags: Fragment
Bodies	cds.comp18583_c0_seq1|m.4424	5	4	Q7PX08_ANOGA | AGAP001151-PA
Bodies	cds.comp149644_c0_seq1|m.51045	5	3	B7P8Q5_IXOSC | Hsp70 putative EC=1.3.1.74 Flags: Fragment
Bodies	cds.comp99596_c0_seq1|m.26322	5	5	R4G324_RHOPR | Putative succinyl-coa synthetase
Bodies	cds.comp124700_c0_seq1|m.43018	5	5	no hit
Bodies	cds.comp9364_c0_seq1|m.2253	5	5	E0VXD2_PEDHC | Homogentisate 1 2-dioxygenase putative EC=1.13.11.5
Bodies	cds.comp22865_c0_seq1|m.5451	5	5	Q3V6S1_TACTR | Cuticular protein
Bodies	cds.comp137687_c0_seq1|m.48124	5	5	A1KXC2_DERFA | DFP2
Bodies	cds.comp76522_c0_seq1|m.18022	5	5	G6CVS9_DANPL | Legumaturain
Bodies	cds.comp41289_c0_seq1|m.9905	5	5	Q4PN05_IXOSC | Putative salivary protein SubName: Full=Secreted salivary gland peptide putative Flags: Fragment
Bodies	cds.comp112615_c0_seq1|m.36876	5	5	G6CKF6_DANPL | Putative prolyl endopeptidase isoform 1
Bodies	cds.comp104760_c0_seq1|m.31142	5	5	E7D1V6_LATHE | Putative uncharacterized protein
Bodies	cds.comp42564_c0_seq1|m.10196	5	5	G9BIX2_PACLE | Calreticulin
Bodies	cds.comp134523_c0_seq1|m.47116	5	5	L7M765_9ACAR | Dihydrolipoyl dehydrogenase EC=1.8.1.4
Bodies	cds.comp101651_c0_seq1|m.28113	5	5	B7PFX8_IXOSC | Gamma-interferon inducible lysosomal thiol reductase putative
Bodies	cds.comp141315_c0_seq1|m.49177	5	5	B0W7T7_CULQU | N(4)-(Beta-N-acetylglucosaminyl)-L-asparaginase
Bodies	cds.comp189_c0_seq1|m.96	5	5	C0L8T6_9CRUS | Glucose-6-phosphate isomerase EC=5.3.1.9
Bodies	cds.comp119406_c0_seq1|m.40745	4	4	A1KXC2_DERFA | DFP2
Bodies	cds.comp108588_c0_seq1|m.34314	4	2	B3GW83_9CRUS | Glyceraldehyde-3-phosphate dehydrogenase EC=1.2.1.12

Bodies/feces	Accession	#Peptides	#Unique	Description
Bodies	cds.comp87855_c0_seq1|m.20561	4	4	G6CL91_DANPL | Erythrocyte carbonic anhydrase
Bodies	cds.comp140939_c0_seq1|m.49098	4	4	Q178W0_AEDAE | AAEL005752-PA
Bodies	Der f 28.0201	4	1	Der f 28.0201
Bodies	cds.comp38046_c0_seq1|m.9091	4	4	Q76FF7_DROYA | Histone H2B Flags: Fragment
Bodies	cds.comp115285_c0_seq1|m.38533	4	4	Q17DM4_AEDAE | AAEL004088-PC SubName: Full=AAEL004088-PD
Bodies	cds.comp55477_c0_seq1|m.13472	4	4	L7LYJ7_9ACAR | Putative ceramidase
Bodies	cds.comp25511_c0_seq1|m.6088	4	2	L0GD06_BOMMO | Glucose-regulated protein 78
Bodies	Der f 18.0101	4	4	Der f 18.0101
Bodies	cds.comp6656_c0_seq1|m.1592	4	4	L7MAK0_9ACAR | Putative acetyl-coa hydrolase
Bodies	cds.comp12568_c0_seq1|m.2984	4	4	B7QC45_IXOSC | Acetylcholinesterase putative EC=3.1.1.7 Flags: Fragment
Bodies	cds.comp107299_c0_seq1|m.33086	4	4	CYTL_TACTR | L-cystatin Flags: Precursor
Bodies	cds.comp21912_c0_seq1|m.5218	4	4	E1AC54_POLVA | Heat shock protein 60
Bodies	cds.comp55005_c0_seq1|m.13338	4	4	L7MGI2_9ACAR | Putative gmp synthase Flags: Fragment
Bodies	cds.comp128011_c0_seq1|m.44520	4	0	B0W0V3_CULQU | Cathepsin L
Bodies	cds.comp14638_c0_seq1|m.3488	4	4	B0W187_CULQU | Myosin-2 heavy chain
Bodies	cds.comp96259_c0_seq1|m.23054	4	4	L7M2A4_9ACAR | Putative alpha actinin
Bodies	cds.comp102395_c0_seq1|m.28705	4	4	L7M1L5_9ACAR | Citrate synthase
Bodies	cds.comp362_c0_seq1|m.164	4	4	Q2YFE3_DERPT | Glutathione transferase delta-like Dp7018E11
Bodies	cds.comp87378_c0_seq1|m.20461	4	4	A1KXH1_DERFA | Der f 13 allergen

Bodies/feces	Accession	#Peptides	#Unique	Description
Bodies	cds.comp116972_c0_seq1|m.39383	4	4	Q28XY6_DROPS | GA19673 isoform A SubName: Full=GA19673 isoform B
Bodies	cds.comp49033_c0_seq1|m.11823	4	4	B4JYL8_DROGR | GH14320
Bodies	cds.comp34734_c0_seq1|m.8337	4	4	E2A1T0_CAMFO | Cytosolic non-specific dipeptidase
Bodies	cds.comp36162_c0_seq1|m.8681	4	4	E9G305_DAPPU | Putative leukotriene A4 hydrolase EC=3.3.2.6
Bodies	cds.comp66639_c0_seq1|m.15883	4	4	Q9GPH2_BOMMO | Protein disulfide isomerase
Bodies	cds.comp38301_c0_seq1|m.9141	4	4	A1KXG9_BLOTA | Blo t 13 allergen
Bodies	cds.comp99761_c0_seq1|m.26510	4	4	I4DM34_PAPPL | Dusky
Bodies	cds.comp34132_c0_seq1|m.8162	4	4	no hit
Bodies	cds.comp128063_c0_seq1|m.44552	4	4	E2BBG1_HARSA | Filamin-C
Bodies	cds.comp28822_c0_seq1|m.6914	4	4	L7M7G4_9ACAR | Putative actin-binding cytoskeleton protein filamin
Bodies	cds.comp80632_c0_seq1|m.19031	4	4	Q1HQJ8_AEDAE | AAEL001102-PA SubName: Full=Adenosine kinase
Bodies	cds.comp1875_c0_seq1|m.497	4	4	A5JM38_ARTSF | SUMO-1-like protein
Bodies	cds.comp119707_c0_seq1|m.40876	4	4	R4G4X4_RHOPR | Putative acid sphingomyelinase and phm5 phosphate metabolism protein
Bodies	cds.comp9679_c0_seq1|m.2330	4	4	E0VQN7_PEDHC | Protein disulfide-isomerase A3 putative EC=2.4.1.119
Bodies	cds.comp50050_c0_seq1|m.12078	4	4	no hit
Bodies	cds.comp1606_c0_seq1|m.437	3	2	A2I458_MACHI | Putative 14-3-3 protein
Bodies	cds.comp122328_c0_seq1|m.42160	3	3	no hit
Bodies	cds.comp32800_c0_seq1|m.7826	3	3	no hit
Bodies	cds.comp108589_c0_seq1|m.34315	3	1	B3GW83_9CRUS | Glyceraldehyde-3-phosphate dehydrogenase EC=1.2.1.12

Bodies/feces	Accession	#Peptides	#Unique	Description
Bodies	Der f 21.0101	3	3	Der f 21.0101
Bodies	cds.comp30659_c0_seq1|m.7326	3	3	B7P8Q5_IXOSC | Hsp70 putative EC=1.3.1.74 Flags: Fragment
Bodies	cds.comp10377_c0_seq1|m.2471	3	3	B7Q5N0_IXOSC | Cuticle protein putative
Bodies	cds.comp13628_c0_seq1|m.3241	3	3	Q9XYC8_AEDAE | AAEL005798-PA SubName: Full=Vacuolar ATPase B subunit
Bodies	Der f 33.0101	3	2	Der f 33.0101
Bodies	cds.comp101702_c0_seq1|m.28157	3	3	B4MQW3_DROWI | GK21952
Bodies	cds.comp54893_c0_seq1|m.13297	3	3	F1CJ03_HOTJU | Putative muscular protein Flags: Fragment
Bodies	cds.comp105078_c0_seq1|m.31445	3	3	L7MFU7_9ACAR | Putative acetylcholinesterase/butyrylcholinesterase Flags: Fragment
Bodies	cds.comp130695_c0_seq1|m.45740	3	2	Q17KK5_AEDAE | AAEL001668-PA
Bodies	cds.comp14471_c0_seq1|m.3445	3	3	Q8MWR4_DERPT | Serine protease LM-1 Flags: Fragment
Bodies	cds.comp127681_c0_seq1|m.44372	3	3	A1KXC1_DERFA | DFP1
Bodies	cds.comp17781_c0_seq1|m.4231	3	3	B5DL96_DROPS | GA22684
Bodies	cds.comp127765_c0_seq1|m.44399	3	3	A1KY40_BLOTA | Major abundant protein BTP1
Bodies	cds.comp22137_c0_seq1|m.5272	3	3	I4DJK2_PAPXU | Similar to CG7781
Bodies	cds.comp85702_c0_seq1|m.20075	3	3	Q5TRG5_ANOGA | AGAP005728-PA
Bodies	cds.comp128009_c0_seq1|m.44514	3	0	B7P3P0_IXOSC | Cathepsin B endopeptidase putative EC=3.4.22.1
Bodies	cds.comp104675_c0_seq1|m.31078	3	3	B7PFJ2_IXOSC | Isocitrate dehydrogenase [NADP] EC=1.1.1.42
Bodies	cds.comp68921_c0_seq1|m.16406	3	3	no hit
Bodies	cds.comp42045_c0_seq1|m.10058	3	3	L7MAJ1_9ACAR | Putative fumarase

Bodies/feces	Accession	#Peptides	#Unique	Description
Bodies	cds.comp106880_c0_seq1|m.32794	3	3	B4LJF4_DROVI | GJ21531
Bodies	cds.comp22946_c0_seq1|m.5466	3	3	R4WR46_9HEMI | Glutamine synthetase 2
Bodies	cds.comp38065_c0_seq1|m.9096	3	3	G3MRB8_9ACAR | Adenosylhomocysteinase EC=3.3.1.1
Bodies	cds.comp112077_c0_seq1|m.36468	3	1	no hit
Bodies	cds.comp116431_c0_seq1|m.39099	3	3	L7LRY5_9ACAR | Putative enoyl-coa isomerase
Bodies	cds.comp135947_c0_seq1|m.47575	3	3	B4NND9_DROWI | GK22908
Bodies	cds.comp102931_c0_seq1|m.29253	3	3	no hit
Bodies	cds.comp68499_c0_seq1|m.16323	3	3	A5LHV9_HAELO | Protein disulfide isomerase-2
Bodies	cds.comp11075_c0_seq1|m.2643	3	3	H9IVV5_BOMMO | Ribose-phosphate pyrophosphokinase EC=2.7.6.1
Bodies	cds.comp108025_c0_seq1|m.33777	3	3	A1KYY1_9ACAR | Sui m 1 allergen
Bodies	cds.comp151186_c0_seq1|m.51392	3	3	L7M384_9ACAR | Putative biotinidase and vanin
Bodies	cds.comp57448_c0_seq1|m.13959	3	3	G6CKL7_DANPL | V-type proton ATPase subunit E
Bodies	cds.comp106225_c0_seq1|m.32397	3	1	B7PDZ5_IXOSC | Alpha-D-galactosidase putative EC=3.2.1.49
Bodies	cds.comp111232_c0_seq1|m.35846	3	3	no hit
Bodies	cds.comp25345_c0_seq1|m.6046	3	3	L7MAT5_9ACAR | Putative succinyl-coa synthetase beta subunit
Bodies	cds.comp140949_c0_seq1|m.49107	3	3	no hit
Bodies	cds.comp46831_c0_seq1|m.11287	3	3	B4XT43_9ARAC | Myosin II regulatory light chain
Bodies	cds.comp6380_c0_seq1|m.1517	3	3	R4G483_RHOPR | Putative radixin length
Bodies	cds.comp63089_c0_seq1|m.15074	3	3	B3TFG6_9ACAR | Esterase TCE1

Bodies/feces	Accession	#Peptides	#Unique	Description
Bodies	cds.comp78678_c0_seq1|m.18518	3	3	no hit
Bodies	cds.comp61962_c0_seq1|m.14848	3	3	CYT_CHIJI | Cystatin JZTX-75 Flags: Precursor
Bodies	cds.comp149778_c0_seq1|m.51069	3	3	A1KXC1_DERFA | DFP1
Bodies	cds.comp581_c0_seq1|m.236	3	3	Q7PQ97_ANOGA | AGAP004394-PA
Bodies	cds.comp53498_c0_seq1|m.12903	3	3	R4UV70_COPFO | Ribosomal protein P1-like protein
Bodies	cds.comp116462_c0_seq1|m.39120	3	3	B5DYT7_DROPS | GA26775
Bodies	cds.comp151211_c0_seq1|m.51394	3	3	no hit
Bodies	cds.comp107643_c0_seq1|m.33453	2	2	D3TS01_GLOMM | Nucleoside diphosphate kinase
Bodies	cds.comp117218_c0_seq1|m.39499	2	2	A5XB12_PENMO | ALG-2 interacting protein x
Bodies	cds.comp127406_c0_seq1|m.44189	2	2	F4WF28_ACREC | Haloacid dehalogenase-like hydrolase domain-containing protein 2
Bodies	cds.comp25024_c0_seq1|m.5969	2	2	B7QM86_IXOSC | Talin putative
Bodies	cds.comp2290_c0_seq1|m.594	2	2	no hit
Bodies	cds.comp75507_c0_seq1|m.17783	2	2	G6CJ88_DANPL | Pyruvate kinase EC=2.7.1.40
Bodies	cds.comp107744_c0_seq1|m.33523	2	2	D2A452_TRICA | Cytochrome b-c1 complex subunit 6 AltName: Full=Complex III subunit 6 AltName: Full=Mitochondrial hinge protein
Bodies	cds.comp77584_c0_seq1|m.18263	2	2	B3LWF9_DROAN | GF18656
Bodies	cds.comp30433_c0_seq1|m.7259	2	2	L7LXU0_9ACAR | Putative x globin
Bodies	cds.comp57443_c0_seq1|m.13958	2	2	D6WXI4_TRICA | 40S ribosomal protein SA
Bodies	cds.comp1846_c0_seq1|m.491	2	2	A1KYY2_9ACAR | Profilin
Bodies	cds.comp28277_c0_seq1|m.6791	2	2	L7M3U4_9ACAR | Putative macrophage migration inhibitory factor

Bodies/feces	Accession	#Peptides	#Unique	Description
Bodies	cds.comp76346_c0_seq1|m.17980	2	2	B7PDK2_IXOSC | Cuticular protein putative
Bodies	cds.comp49462_c0_seq1|m.11922	2	2	B4MNQ5_DROWI | GK19626
Bodies	cds.comp77177_c0_seq1|m.18163	2	2	B4J0R3_DROGR | GH17138
Bodies	cds.comp32709_c0_seq1|m.7805	2	2	B4NE95_DROWI | GK25335
Bodies	cds.comp55771_c0_seq1|m.13546	2	0	A1KXG2_DERFA | Peptidyl-prolyl cis-trans isomerase EC=5.2.1.8
Bodies	cds.comp115284_c0_seq1|m.38532	2	2	Q7PF06_ANOGA | AGAP011050-PA
Bodies	cds.comp106224_c0_seq1|m.32394	2	0	Q291S9_DROPS | GA20753 EC=3.2.1.-
Bodies	cds.comp150229_c0_seq1|m.51182	2	2	no hit
Bodies	cds.comp112078_c0_seq1|m.36473	2	0	no hit
Bodies	cds.comp128365_c0_seq1|m.44774	2	2	Q52NW5_9DIPT | Serine protease
Bodies	cds.comp34529_c0_seq1|m.8271	2	2	L7LWG4_9ACAR | Putative hu li tai shao
Bodies	cds.comp115994_c0_seq1|m.38953	2	2	A7UUE6_ANOGA | AGAP006584-PA
Bodies	cds.comp105193_c0_seq1|m.31579	2	2	D6X0G1_TRICA | Elongation factor Tu
Bodies	cds.comp8003_c0_seq1|m.1913	2	2	no hit
Bodies	cds.comp104556_c0_seq1|m.30964	2	2	B7PXP4_IXOSC | Hsp70 putative EC=1.3.1.74 Flags: Fragment
Bodies	cds.comp105191_c0_seq1|m.31572	2	2	L7M0B5_9ACAR | Putative beta-glucocerebrosidase
Bodies	cds.comp79176_c0_seq1|m.18648	2	2	R4G8W4_RHOPR | Putative actin filament-coating protein tropomyosin Flags: Fragment
Bodies	cds.comp123704_c0_seq1|m.42635	2	2	B4MSV2_DROWI | GK20039
Bodies	cds.comp96367_c0_seq1|m.23125	2	2	Q177R1_AEDAE | Proteasome subunit alpha type EC=3.4.25.1

Bodies/feces	Accession	#Peptides	#Unique	Description
Bodies	cds.comp99847_c0_seq1|m.26611	2	2	E0VS60_PEDHC | Alcohol dehydrogenase putative EC=1.1.1.100
Bodies	cds.comp96075_c0_seq1|m.22827	2	2	Q5TQ02_ANOGA | AGAP011457-PA Flags: Fragment
Bodies	cds.comp12516_c0_seq1|m.2968	2	2	B7QH76_IXOSC | Secreted protein putative
Bodies	cds.comp107755_c0_seq1|m.33525	2	2	B4JX76_DROGR | GH17897
Bodies	cds.comp101173_c0_seq1|m.27872	2	2	L7M7I7_9ACAR | Putative alkyl hydroperoxide reductase thiol specific antioxidant
Bodies	cds.comp11593_c0_seq1|m.2746	2	2	R4FNB7_RHOPR | Putative short-chain alcohol dehydrogenase/3-hydroxyacyl-coa dehydrogenase
Bodies	cds.comp112135_c0_seq1|m.36520	2	2	B7Q5M9_IXOSC | Cuticle protein putative
Bodies	cds.comp56907_c0_seq1|m.13817	2	2	R4I416_BACDO | Phosphoglucose isomerase
Bodies	cds.comp87133_c0_seq1|m.20405	2	2	B7P0M7_IXOSC | Aldehyde dehydrogenase putative EC=1.5.1.12 Flags: Fragment
Bodies	cds.comp128063_c0_seq1|m.44553	2	2	E2BBG1_HARSA | Filamin-C
Bodies	cds.comp13305_c0_seq1|m.3171	2	2	B3TFG6_9ACAR | Esterase TCE1
Bodies	cds.comp7534_c0_seq1|m.1813	2	2	no hit
Bodies	cds.comp4245_c0_seq1|m.1004	2	2	Q4GXQ8_9COLE | Ribosomal protein S15e
Bodies	cds.comp17156_c0_seq1|m.4102	2	2	Q2YFF0_SARSC | Glutathione transferase mu class Yv5004H11
Bodies	cds.comp21084_c0_seq1|m.5045	2	2	no hit
Bodies	cds.comp24266_c0_seq1|m.5821	2	2	no hit
Bodies	cds.comp122845_c0_seq1|m.42301	2	2	no hit
Bodies	cds.comp127085_c0_seq1|m.44018	2	2	A1KY40_BLOTA | Major abundant protein BTP1
Bodies	cds.comp14123_c0_seq1|m.3349	2	2	E0VQP6_PEDHC | Leukotriene A-4 hydrolase putative EC=3.3.2.6

Bodies/feces	Accession	#Peptides	#Unique	Description
Bodies	cds.comp17033_c0_seq1|m.4072	2	2	Q7QKK7_ANOGA | AGAP003261-PA
Bodies	cds.comp104812_c0_seq1|m.31219	2	2	no hit
Bodies	Der f 27.0101	2	2	Der f 27.0101
Bodies	cds.comp30657_c0_seq1|m.7324	2	2	I1ZE45_DERFA | Heat shock protein cognate 5 Flags: Fragment
Bodies	cds.comp21541_c0_seq1|m.5129	2	2	B7P8F8_IXOSC | cAMP-dependent protein kinase type I-beta regulatory subunit putative EC=2.7.10.2 Flags: Fragment
Bodies	cds.comp33701_c0_seq1|m.8037	2	2	B4YTT9_9ACAR | Heat shock protein 70-2
Bodies	cds.comp30656_c0_seq1|m.7323	2	2	I1ZE46_9ACAR | Heat shock protein cognate 5 Flags: Fragment
Bodies	cds.comp99177_c0_seq1|m.25924	2	2	L7M7J5_9ACAR | Putative 20s proteasome regulatory subunit beta type psmb1/pre7
Bodies	cds.comp19800_c0_seq1|m.4709	2	2	F0JA41_AMBVA | Na+/K+ ATPase beta subunit
Bodies	cds.comp59092_c0_seq1|m.14316	2	2	A9QQ40_LYCSI | 60s ribosomal protein L14
Bodies	cds.comp102099_c0_seq1|m.28494	2	2	no hit
Bodies	cds.comp59259_c0_seq1|m.14338	2	2	Q09JT7_ARGMO | Ca2+ calmodulin dependent protein kinase EF-Hand protein superfamily
Bodies	cds.comp96119_c0_seq1|m.22871	2	2	A9QQ87_LYCSI | 40S ribosomal protein S7
Bodies	cds.comp38513_c0_seq1|m.9200	2	2	K7IPQ8_NASVI | Proteasome subunit beta type EC=3.4.25.1
Bodies	cds.comp108516_c0_seq1|m.34242	2	2	E0VLF6_PEDHC | Spectrin beta chain putative EC=3.1.3.48
Bodies	cds.comp93681_c0_seq1|m.21819	2	2	B7QE79_IXOSC | Vesicle coat complex COPII subunit SFB3 putative
Bodies	cds.comp97982_c0_seq1|m.24536	2	2	E2BH78_HARSA | Phosphatidylinositol-specific phospholipase C X domain-containing protein 1
Bodies	cds.comp109890_c0_seq1|m.35037	2	2	B7QAN4_IXOSC | Sorbitol dehydrogenase putative EC=1.1.1.14
Bodies	cds.comp124493_c0_seq1|m.42940	2	2	G6DI61_DANPL | Phosphoglycerate kinase EC=2.7.2.3

Bodies/feces	Accession	#Peptides	#Unique	Description
Bodies	cds.comp139146_c0_seq1|m.48562	2	2	G0WRZ9_RHIMP | Papilin Flags: Fragment
Bodies	cds.comp56910_c0_seq1|m.13818	2	2	Q28Y79_DROPS | Glucose-6-phosphate isomerase EC=5.3.1.9
Bodies	cds.comp63052_c0_seq1|m.15063	2	2	E9HAK9_DAPPU | Lamin-like protein
Bodies	cds.comp3663_c0_seq1|m.888	2	2	L7M3W8_9ACAR | Putative tetraspanin-like protein
Bodies	cds.comp77670_c0_seq1|m.18275	2	2	Q17M31_AEDAE | AAEL001154-PA
Bodies	cds.comp39114_c0_seq1|m.9358	2	2	L7M493_9ACAR | Putative complement component 1 q subcomponent binding protein/mrna splicing factor sf2 subunit p32
Bodies	cds.comp57406_c0_seq1|m.13946	2	2	Q8IMI0_DROME | CG2246 isoform B EC=2.7.6.1 SubName: Full=CG2246 isoform G EC=2.7.6.1 SubName: Full=LD38861p SubName: Full=LP08261p
Bodies	cds.comp75006_c0_seq1|m.17679	2	1	B4GMN3_DROPE | GL12416
Bodies	cds.comp60010_c0_seq1|m.14481	2	2	D3TSB1_GLOMM | Isocitrate dehydrogenase alpha subunit
Bodies	cds.comp57442_c0_seq1|m.13956	2	2	E7D1C2_LATHE | Putative ribosomal protein SA Flags: Fragment
Bodies	cds.comp108798_c0_seq1|m.34424	2	2	G6CUI4_DANPL | Molting fluid carboxypeptidase A
Bodies	cds.comp50907_c0_seq1|m.12297	2	2	L7LXZ6_9ACAR | Beta-hexosaminidase EC=3.2.1.52
Bodies	cds.comp2620_c0_seq1|m.671	2	2	B7QCR3_IXOSC | Transmembrane protein putative
Bodies	cds.comp33705_c0_seq1|m.8040	2	2	Q1HR69_AEDAE | AAEL017349-PA SubName: Full=Heat shock cognate 70
Bodies	cds.comp121567_c0_seq1|m.41816	2	2	F5HL86_ANOGA | AGAP003513-PB
Bodies	cds.comp76298_c0_seq1|m.17965	2	2	C1IE32_9HEXA | Beta-1 3-D-glucanase EC=3.2.1.6 SubName: Full=Endo-beta-1 3-glucanase EC=3.2.1.39 Flags: Precursor
Bodies	cds.comp62940_c0_seq1|m.15039	2	2	L7MAC6_9ACAR | Putative glutamate/leucine/phenylalanine/valine dehydrogenase
Bodies	cds.comp103358_c0_seq1|m.29778	2	2	C4WUM8_ACYPI | ACYPI003308 protein SubName: Full=Uncharacterized protein
Bodies	cds.comp127448_c0_seq1|m.44236	2	2	Q2F5T3_BOMMO | ATP synthase subunit alpha

Bodies/feces	Accession	#Peptides	#Unique	Description
Bodies	cds.comp103865_c0_seq1|m.30178	2	2	A9QQA8_LYCSI | 40S ribosomal protein S5
Bodies	cds.comp138036_c0_seq1|m.48231	2	2	C1BTI7_9MAXI | Fructose-bisphosphate aldolase
Bodies	cds.comp72358_c0_seq1|m.17098	2	2	L7M0S7_9ACAR | Pyrroline-5-carboxylate reductase EC=1.5.1.2
Feces	Der f 1.0107	44	0	Der f 1.0107
Feces	Der f 1.0106	38	1	Der f 1.0106
Feces	Der f 1.0105	38	0	Der f 1.0105
Feces	Der f 15.0101	29	3	Der f 15.0101
Feces	cds.comp55138_c0_seq1|m.13375	27	1	Q9U6R7_DERFA | 98kDa HDM allergen SubName: Full=Group 15 allergen Der f 15
Feces	cds.comp101423_c0_seq1|m.28025	23	6	A1KXH3_DERFA | Der f 3 allergen
Feces	cds.comp114970_c0_seq1|m.38343	23	23	Der f 36
Feces	cds.comp7296_c0_seq1|m.1765	22	22	E2BEF1_HARSA | Lysosomal alpha-glucosidase Flags: Fragment
Feces	cds.comp100364_c0_seq1|m.27163	22	22	Q8MRY3_DROME | SD13780p
Feces	Der f 4.0101	22	22	Der f 4.0101
Feces	Der f 2.0103	21	4	Der f 2.0103
Feces	cds.comp80154_c0_seq1|m.18914	20	20	Der f 35
Feces	cds.comp7297_c0_seq1|m.1766	18	17	E2A599_CAMFO | Lysosomal alpha-glucosidase
Feces	Der f 2.0102	18	0	Der f 2.0102
Feces	Der f 3.0101	18	1	Der f 3.0101
Feces	cds.comp140939_c0_seq1|m.49097	13	13	Q5TS83_ANOGA | AGAP008584-PA

Bodies/feces	Accession	#Peptides	#Unique	Description
Feces	cds.comp128016_c0_seq1|m.44537	12	4	B7P3P1_IXOSC | Cathepsin B endopeptidase putative EC=3.4.22.1
Feces	cds.comp99077_c0_seq1|m.25746	12	12	Q8MWR6_DERPT | 14.5 kDa bacteriolytic enzyme
Feces	Der f 2.0116	12	0	Der f 2.0116
Feces	Der f 6.0101	12	7	Der f 6.0101
Feces	cds.comp128009_c0_seq1|m.44514	11	0	B7P3P0_IXOSC | Cathepsin B endopeptidase putative EC=3.4.22.1
Feces	cds.comp128012_c0_seq1|m.44522	11	3	B7P3P0_IXOSC | Cathepsin B endopeptidase putative EC=3.4.22.1
Feces	cds.comp104802_c0_seq1|m.31198	11	11	B7PXR5_IXOSC | Chaperonin complex component TCP-1 eta subunit putative
Feces	cds.comp83879_c0_seq1|m.19673	11	11	R4G3J7_RHOPR | Putative gamma interferon inducible lysosomal thiol reductase gilt
Feces	cds.comp39311_c0_seq1|m.9404	9	9	F4W8Y5_ACREC | Lysosomal alpha-mannosidase
Feces	cds.comp14471_c0_seq1|m.3445	9	9	Q8MWR4_DERPT | Serine protease LM-1 Flags: Fragment
Feces	cds.comp108273_c0_seq1|m.34021	9	9	Q0KKA6_HAELO | Leucine aminopeptidase
Feces	cds.comp101651_c0_seq1|m.28113	9	9	B7PFX8_IXOSC | Gamma-interferon inducible lysosomal thiol reductase putative
Feces	cds.comp128015_c0_seq1|m.44536	9	1	B7P3P1_IXOSC | Cathepsin B endopeptidase putative EC=3.4.22.1
Feces	cds.comp112367_c0_seq1|m.36666	9	4	Q155V8_DERFA | Der f 6 Flags: Fragment
Feces	cds.comp103780_c0_seq1|m.30108	7	7	no hit
Feces	cds.comp104556_c0_seq1|m.30964	7	7	B7PXP4_IXOSC | Hsp70 putative EC=1.3.1.74 Flags: Fragment
Feces	Der f 2.0109	7	0	Der f 2.0109
Feces	Der f 18.0101	7	7	Der f 18.0101
Feces	cds.comp131650_c0_seq1|m.46115	6	6	no hit

Bodies/feces	Accession	#Peptides	#Unique	Description
Feces	cds.comp39309_c0_seq1|m.9403	6	6	E2ABS8_CAMFO | Lysosomal alpha-mannosidase
Feces	cds.comp140939_c0_seq1|m.49098	6	6	Q178W0_AEDAE | AAEL005752-PA
Feces	cds.comp15838_c0_seq1|m.3755	6	6	B4R043_DROSI | GD21438
Feces	cds.comp151186_c0_seq1|m.51392	6	6	L7M384_9ACAR | Putative biotinidase and vanin
Feces	cds.comp98584_c0_seq1|m.25201	6	6	no hit
Feces	cds.comp33654_c0_seq1|m.8020	6	6	A1YW13_DERFA | Der f 1 allergen
Feces	Der f 22.0101	6	6	Der f 22.0101
Feces	cds.comp76522_c0_seq1|m.18022	5	5	G6CVS9_DANPL | Legumaturain
Feces	cds.comp108798_c0_seq1|m.34424	5	5	G6CUI4_DANPL | Molting fluid carboxypeptidase A
Feces	cds.comp25511_c0_seq1|m.6088	5	3	L0GD06_BOMMO | Glucose-regulated protein 78
Feces	cds.comp103306_c0_seq1|m.29699	4	4	B7P417_IXOSC | Peritrophic membrane chitin binding protein putative
Feces	cds.comp50907_c0_seq1|m.12297	4	4	L7LXZ6_9ACAR | Beta-hexosaminidase EC=3.2.1.52
Feces	cds.comp103285_c0_seq1|m.29677	4	4	A1KXG7_DERFA | Der f 7 allergen
Feces	cds.comp106225_c0_seq1|m.32397	4	2	B7PDZ5_IXOSC | Alpha-D-galactosidase putative EC=3.2.1.49
Feces	cds.comp151211_c0_seq1|m.51394	4	4	no hit
Feces	Der f 28.0101	4	2	Der f 28.0101
Feces	Der f 27.0101	4	4	Der f 27.0101
Feces	cds.comp149823_c0_seq1|m.51085	3	1	M4LIT4_COTVE | Heat shock protein 70
Feces	cds.comp103689_c0_seq1|m.30038	3	3	E9HAY5_DAPPU | Putative uncharacterized protein

Bodies/feces	Accession	#Peptides	#Unique	Description
Feces	cds.comp106224_c0_seq1|m.32394	3	1	Q291S9_DROPS | GA20753 EC=3.2.1.-
Feces	cds.comp104803_c0_seq1|m.31204	3	3	B7PXR5_IXOSC | Chaperonin complex component TCP-1 eta subunit putative
Feces	cds.comp122845_c0_seq1|m.42301	3	3	no hit
Feces	cds.comp97297_c0_seq1|m.24021	2	2	no hit
Feces	cds.comp40810_c0_seq1|m.9786	2	2	no hit
Feces	cds.comp119707_c0_seq1|m.40876	2	2	R4G4X4_RHOPR | Putative acid sphingomyelinase and phm5 phosphate metabolism protein
Feces	cds.comp73688_c0_seq1|m.17401	2	2	B7PXR0_IXOSC | Elongation factor Tu Flags: Fragment
Feces	cds.comp91035_c0_seq1|m.21227	2	2	B3NH03_DROER | GG13891
Feces	cds.comp102099_c0_seq1|m.28494	2	2	no hit
Feces	cds.comp105191_c0_seq1|m.31572	2	2	L7M0B5_9ACAR | Putative beta-glucocerebrosidase
Feces	cds.comp13305_c0_seq1|m.3171	2	2	B3TFG6_9ACAR | Esterase TCE1
Feces	cds.comp135557_c0_seq1|m.47461	2	2	B7QML9_IXOSC | Lipase putative EC=3.1.1.3 Flags: Fragment
Feces	cds.comp107299_c0_seq1|m.33086	2	2	CYTL_TACTR | L-cystatin Flags: Precursor
Feces	cds.comp111232_c0_seq1|m.35846	2	2	no hit
Feces	cds.comp113240_c0_seq1|m.37386	2	1	Q8ISH5_ARAVE | Chitinase
Feces	cds.comp7534_c0_seq1|m.1813	2	2	no hit
Feces	cds.comp115994_c0_seq1|m.38953	2	2	A7UUE6_ANOGA | AGAP006584-PA
Feces	cds.comp96190_c0_seq1|m.22957	2	2	no hit
Feces	cds.comp127515_c0_seq1|m.44304	2	1	E0V9V3_PEDHC | Alpha glucosidase putative EC=3.2.1.3

Bodies/feces	Accession	#Peptides	#Unique	Description
Feces	cds.comp106751_c0_seq1|m.32734	2	2	no hit
Feces	cds.comp96086_c0_seq1|m.22836	2	2	Q09JE3_ARGMO | Superoxide dismutase [Cu-Zn] EC=1.15.1.1
Feces	cds.comp8003_c0_seq1|m.1913	2	2	no hit
Feces	cds.comp119370_c0_seq1|m.40727	2	2	L7MIP8_9ACAR | Putative beta-lactamase Flags: Fragment
Feces	cds.comp119114_c0_seq1|m.40635	2	2	L7M2B8_9ACAR | Putative n-acylaminoacyl-peptide hydrolase
Feces	Der f 28.0201	2	0	Der f 28.0201
Feces	Der f 25.0201	2	2	Der f 25.0201
