# Supplementary material for: A combined transcriptome and proteome analysis extends the allergome of house dust mite Dermatophagoides species
Source: PLoS One. 2017 Oct 5;12(10):e0185830. doi: 10.1371/journal.pone.0185830 (PMC5628879; doi:10.1371/journal.pone.0185830)
Supplement: S2 Table — Protein extracts from D. pteronyssinus fractionated bodies and feces were analyzed by LC-MS/MS. Protein identification was performed using the species-specific transcriptome derived protein database, supplemented with IUIS-registered allergen sequences, as reference dataset. Only entries identified by a minimum of 2 peptides sequenced were taken into account. Proteins are reported by the entry name with the total numbers of supporting sequenced peptides (#peptides) and of uniquely mapping peptides (#unique) as well as, when available, the result of the annotation by blast analysis. (PDF) [file pone.0185830.s005.pdf]

Bodies/feces	Accession	#Peptides	#Unique	Description
bodies	cds.comp31381_c0_seq1|m.60541	38	5	G6DI19_DANPL | Putative cuticle protein CPH35
bodies	cds.comp48395_c0_seq1|m.90885	38	3	Q8N0N0_DERPT | Group 14 allergen protein Flags: Fragment
bodies	cds.comp31379_c0_seq1|m.60537	37	4	I4DIE5_PAPXU | Cuticular protein PxutCPG13
bodies	Der p 14.0101	35	0	Der p 14.0101
bodies	cds.comp116084_c0_seq1|m.195549	20	3	PEPT1_DERPT | Peptidase 1 EC=3.4.22.65 AltName: Full=Allergen Der p I AltName: Full=Major mite fecal allergen Der p 1 AltName: Allergen=Der p 1 Flags: Precursor
bodies	Der p 1.0115	19	0	Der p 1.0115
bodies	cds.comp35682_c0_seq1|m.69084	19	19	Q6Y2F9_DERPT | HDM allergen
bodies	Der p 1.0108	18	0	Der p 1.0108
bodies	Der p 4.0101	18	18	Der p 4.0101
bodies	cds.comp70824_c0_seq1|m.129938	17	12	Q71SP3_HELZE | Cytoplasmic actin A3a1 SubName: Full=Cytoplasmic actin A3b
bodies	cds.comp121128_c0_seq1|m.201850	16	16	A1KXC2_DERFA | DFP2
bodies	Der p 1.0111	16	0	Der p 1.0111
bodies	cds.comp102701_c0_seq1|m.178171	16	8	A1KXC3_DERFA | Arginine kinase
bodies	Der p 1.0112	15	0	Der p 1.0112
bodies	Der p 1.0103	15	0	Der p 1.0103
bodies	cds.comp25545_c0_seq1|m.49294	15	15	E9FZS8_DAPPU | Myosin heavy chain isoform 3
bodies	cds.comp8426_c0_seq1|m.15013	14	14	G6DI19_DANPL | Putative cuticle protein CPH35
bodies	Der p 1.0104	14	1	Der p 1.0104
bodies	cds.comp104308_c0_seq1|m.180148	14	14	B6E457_DERPT | Tropomyosin

Bodies/feces	Accession	#Peptides	#Unique	Description
bodies	cds.comp86986_c0_seq1|m.155830	13	13	no hit
bodies	cds.comp70793_c0_seq1|m.129873	13	8	B2MVM3_SARSC | Actin
bodies	cds.comp58727_c0_seq1|m.109493	13	13	G9C5D5_SCHGR | Fructose-bisphosphate aldolase EC=4.1.2.13
bodies	Der p 2.0106	12	0	Der p 2.0106
bodies	Der p 2.0107	12	0	Der p 2.0107
bodies	Der p 2.0105	12	0	Der p 2.0105
bodies	cds.comp23278_c0_seq1|m.44326	12	12	Q8MWR6_DERPT | 14.5 kDa bacteriolytic enzyme
bodies	cds.comp72134_c0_seq1|m.132054	12	12	DERP3_DERPT | Mite allergen Der p 3 EC=3.4.21.- AltName: Full=Allergen Der p III AltName: Allergen=Der p 3 Flags: Precursor
bodies	Der p 6.0101	12	12	Der p 6.0101
bodies	cds.comp117249_c0_seq1|m.197144	11	11	ALL2_PSOOV | Mite group 2 allergen Pso o 2 AltName: Full=Allergen Pso o A AltName: Allergen=Pso o 2 Flags: Precursor
bodies	Der p 2.0102	11	0	Der p 2.0102
bodies	Der p 2.0103	11	0	Der p 2.0103
bodies	Der p 2.0109	11	0	Der p 2.0109
bodies	Der p 2.0104	11	0	Der p 2.0104
bodies	Der p 20.0101	11	2	Der p 20.0101
bodies	cds.comp92984_c0_seq1|m.165212	11	11	R4V1A4_COPFO | Enolase
bodies	cds.comp8048_c0_seq1|m.14323	11	5	PEPT1_EURMA | Peptidase 1 EC=3.4.22.65 AltName: Full=Allergen Eur m I AltName: Full=Mite group 1 allergen Eur m 1 AltName: Allergen=Eur m 1 Flags: Precursor
bodies	Der p 2.0113	10	0	Der p 2.0113
bodies	Der p 2.0111	10	0	Der p 2.0111

Bodies/feces	Accession	#Peptides	#Unique	Description
bodies	Der p 2.0112	10	0	Der p 2.0112
bodies	cds.comp74726_c0_seq1|m.136742	10	10	A1KXG7_DERFA	Der f 7 allergen
bodies	cds.comp63345_c0_seq1|m.117493	10	10	I1ZCC6_DERFA	Elongation factor 1-alpha Flags: Fragment
bodies	cds.comp134821_c0_seq1|m.213456	10	2	Q7Z163_DERPT	Trypsin-like serine protease
bodies	cds.comp25516_c0_seq1|m.49092	9	6	B7P1Q2_IXOSC	Myosin heavy chain skeletal muscle or cardiac muscle putative EC=1.3.1.74
bodies	cds.comp54535_c0_seq1|m.102040	9	9	A1KXH8_DERFA	Der f Gal d 2 allergen
bodies	cds.comp17639_c0_seq1|m.32899	9	9	B7Q5N0_IXOSC	Cuticle protein putative
bodies	cds.comp89996_c0_seq1|m.160636	9	9	B7QE46_IXOSC	ATP synthase subunit beta EC=3.6.3.14
bodies	cds.comp57924_c0_seq1|m.107752	9	9	Q7PF06_ANOGA	AGAP011050-PA
bodies	cds.comp136842_c0_seq1|m.214781	9	9	L7UZA7_DERFA	Triosephosphate isomerase EC=5.3.1.1
bodies	cds.comp122662_c0_seq1|m.203436	8	2	A1KY40_BLOTA	Major abundant protein BTP1
bodies	cds.comp99064_c0_seq1|m.173024	8	8	A1KXC2_DERFA	DFP2
bodies	cds.comp79146_c0_seq1|m.143353	8	2	A1KXC2_DERFA	DFP2
bodies	cds.comp31214_c0_seq1|m.60174	8	5	A1KXC1_DERFA	DFP1
bodies	cds.comp109184_c0_seq1|m.186519	8	2	no hit
bodies	cds.comp15419_c0_seq1|m.28597	8	8	Q16XV8_AEDAE	AAEL008748-PA
bodies	Der p 9.0101	8	0	Der p 9.0101
bodies	cds.comp71098_c0_seq1|m.130257	8	7	B4YTT9_9ACAR	Heat shock protein 70-2
bodies	cds.comp31219_c0_seq1|m.60181	7	0	A1KXC1_DERFA	DFP1

Bodies/feces	Accession	#Peptides	#Unique	Description
bodies	cds.comp112920_c0_seq1|m.190979	7	7	Der p 36
bodies	cds.comp109183_c0_seq1|m.186518	7	1	no hit
bodies	cds.comp8053_c0_seq1|m.14331	7	2	PEPT1_EURMA | Peptidase 1 EC=3.4.22.65 AltName: Full=Allergen Eur m I AltName: Full=Mite group 1 allergen Eur m 1 AltName: Allergen=Eur m 1 Flags: Precursor
bodies	cds.comp35615_c0_seq1|m.68910	7	7	E1AC54_POLVA | Heat shock protein 60
bodies	cds.comp39348_c0_seq1|m.74983	7	7	Q09JE3_ARGMO | Superoxide dismutase [Cu-Zn] EC=1.15.1.1
bodies	cds.comp65555_c0_seq1|m.121353	7	7	B7QIG6_IXOSC | Secreted salivary gland peptide putative
bodies	cds.comp136643_c0_seq1|m.214661	7	7	E2C2J9_HARSA | Pancreatic triacylglycerol lipase
bodies	cds.comp110944_c0_seq1|m.188713	7	6	A1KXH7_DERFA | Der f Alt a 10 allergen
bodies	cds.comp86167_c0_seq1|m.154658	6	6	R4FNL2_RHOPR | Putative cathepsin b-like proteinase
bodies	cds.comp46754_c0_seq1|m.88321	6	5	B7Q8W6_IXOSC | Alkyl hydroperoxide reductase thiol specific antioxidant putative EC=1.11.1.15
bodies	cds.comp8060_c0_seq1|m.14342	6	2	PEPT1_EURMA | Peptidase 1 EC=3.4.22.65 AltName: Full=Allergen Eur m I AltName: Full=Mite group 1 allergen Eur m 1 AltName: Allergen=Eur m 1 Flags: Precursor
bodies	cds.comp2539_c0_seq1|m.3388	6	6	K0GG83_9HEMI | Na+ K+ ATPase alpha-subunit 1
bodies	cds.comp4304_c0_seq1|m.6632	6	6	E2A599_CAMFO | Lysosomal alpha-glucosidase
bodies	cds.comp103957_c0_seq1|m.179732	6	6	A1KXG2_DERFA | Peptidyl-prolyl cis-trans isomerase EC=5.2.1.8
bodies	cds.comp32379_c0_seq1|m.62353	6	6	K7N8M7_HAELO | Myosin alkali light chain protein
bodies	cds.comp94768_c0_seq1|m.167473	6	6	C1BS68_9MAXI | 3-oxoacyl-acyl-carrier-protein reductase
bodies	cds.comp130814_c0_seq1|m.210670	6	6	G3MND0_9ACAR | Aspartate aminotransferase EC=2.6.1.1
bodies	cds.comp122816_c0_seq1|m.203611	5	5	C1IE32_9HEXA | Beta-1 3-D-glucanase EC=3.2.1.6 SubName: Full=Endo-beta-1 3-glucanase EC=3.2.1.39 Flags: Precursor
bodies	cds.comp102709_c0_seq1|m.178202	5	0	B2ZSY4_DERPT | Der p 20 allergen

Bodies/feces	Accession	#Peptides	#Unique	Description
bodies	cds.comp62311_c0_seq1|m.115826	5	5	B7Q0R0_IXOSC | Phosphoglycerate mutase putative EC=5.4.2.1
bodies	cds.comp53542_c0_seq1|m.100389	5	5	G3MH40_9ACAR | Malate dehydrogenase EC=1.1.1.37 Flags: Fragment
bodies	cds.comp11084_c0_seq1|m.19766	5	5	F4W8Y5_ACREC | Lysosomal alpha-mannosidase
bodies	cds.comp9913_c0_seq1|m.17779	5	5	Q0KKA6_HAELO | Leucine aminopeptidase
bodies	cds.comp129706_c0_seq1|m.209819	5	5	A1KY40_BLOTA | Major abundant protein BTP1
bodies	cds.comp120513_c0_seq1|m.201013	5	5	L7MAA0_9ACAR | ATP synthase subunit alpha
bodies	cds.comp25750_c0_seq1|m.49621	5	5	Q1HQT8_AEDAE | AAEL007549-PA SubName: Full=Phosphatidylethanolamine-binding protein
bodies	cds.comp51043_c0_seq1|m.95888	5	5	PN16_PHONI | U24-ctenitoxin-Pn1a Short=U24-CNTX-Pn1a AltName: Full=Venom protein PN16C3
bodies	cds.comp67054_c0_seq1|m.123503	5	4	F4WTV7_ACREC | Heat shock 70 kDa protein cognate 4
bodies	cds.comp15332_c0_seq1|m.28429	5	5	Q2YFE4_DERPT | Glutathione transferase mu class Dp7002H05
bodies	cds.comp18129_c0_seq1|m.33891	5	5	K7J833_NASVI | Malate dehydrogenase EC=1.1.1.37
bodies	cds.comp39143_c0_seq1|m.74551	5	5	Q1M0Y2_BLOTA | Blo t allergen
bodies	cds.comp107938_c0_seq1|m.184963	5	4	A7XZK3_9ACAR | Aldehyde dehydrogenase
bodies	cds.comp28362_c0_seq1|m.54728	5	5	E2AB44_CAMFO | Cuticlin-1
bodies	cds.comp142310_c0_seq1|m.217545	4	4	Q8MRY3_DROME | SD13780p
bodies	cds.comp31224_c0_seq1|m.60185	4	0	A1KXC1_DERFA | DFP1
bodies	cds.comp25527_c0_seq1|m.49141	4	1	B7P1Q2_IXOSC | Myosin heavy chain skeletal muscle or cardiac muscle putative EC=1.3.1.74
bodies	cds.comp86199_c0_seq1|m.154685	4	4	no hit
bodies	cds.comp113441_c0_seq1|m.191645	4	4	F1CJ03_HOTJU | Putative muscular protein Flags: Fragment

Bodies/feces	Accession	#Peptides	#Unique	Description
bodies	cds.comp78002_c0_seq1|m.141751	4	4	A1KXC2_DERFA | DFP2
bodies	cds.comp99232_c0_seq1|m.173247	4	4	Q8MVU3_DERFA | Gelsolin-like allergen Der f 16
bodies	cds.comp123220_c0_seq1|m.204034	4	4	E2BBG1_HARSA | Filamin-C
bodies	cds.comp115052_c0_seq1|m.194257	4	4	E2BBG1_HARSA | Filamin-C
bodies	cds.comp82518_c0_seq1|m.148910	4	4	Q962I7_DERPT | Ferritin
bodies	cds.comp98676_c0_seq1|m.172570	4	4	D7URK4_SARSC | Myosin heavy chain Flags: Fragment
bodies	cds.comp71148_c0_seq1|m.130327	4	4	Q4JK71_DERPT | Group 18 allergen protein
bodies	cds.comp142811_c0_seq1|m.217782	4	4	A7UNZ5_DERFA | Der f 1 allergen
bodies	cds.comp37403_c0_seq1|m.71374	4	4	E2C8M6_HARSA | Elongation factor 2
bodies	cds.comp74079_c0_seq1|m.135782	4	1	G9JKY3_BACDO | Protein disulfide isomerase
bodies	cds.comp90815_c0_seq1|m.161796	4	4	L7M2J0_9ACAR | Putative aminopeptidase of the m17 family
bodies	Der p 8.0101	4	4	Der p 8.0101
bodies	cds.comp120601_c0_seq1|m.201099	4	4	Q17G61_AEDAE | AAEL003193-PA
bodies	cds.comp148823_c0_seq1|m.219720	4	4	no hit
bodies	cds.comp123766_c0_seq1|m.204555	3	3	A1KY40_BLOTA | Major abundant protein BTP1
bodies	cds.comp35892_c0_seq1|m.69332	3	3	B0G0U7_MONAT | Beta1-tubulin
bodies	cds.comp112920_c0_seq1|m.190978	3	3	B7PB45_IXOSC | Transferrin receptor putative EC=3.4.11.10
bodies	cds.comp100564_c0_seq1|m.175136	3	3	B7Q8U6_IXOSC | Adenosine kinase putative EC=2.7.1.20
bodies	cds.comp74081_c0_seq1|m.135792	3	0	G9JKY3_BACDO | Protein disulfide isomerase

Bodies/feces	Accession	#Peptides	#Unique	Description
bodies	cds.comp93400_c0_seq1|m.165795	3	3	A1KXG9_BLOTA | Blo t 13 allergen
bodies	cds.comp52875_c0_seq1|m.99307	3	3	A1KXC2_DERFA | DFP2
bodies	cds.comp66329_c0_seq1|m.122478	3	3	B7Q6Z1_IXOSC | Saposin putative EC=3.4.23.40
bodies	cds.comp19823_c0_seq1|m.36964	3	3	L7LXL4_9ACAR | Inosine-5'-monophosphate dehydrogenase Short=IMP dehydrogenase Short=IMPD Short=IMPDH EC=1.1.1.205
bodies	cds.comp42815_c0_seq1|m.80900	3	3	H9K697_APIME | Histone H4
bodies	cds.comp73673_c0_seq1|m.134961	3	3	G9BIX2_PACLE | Calreticulin
bodies	cds.comp135080_c0_seq1|m.213643	3	3	no hit
bodies	cds.comp71109_c0_seq1|m.130285	3	3	A1KXC1_DERFA | DFP1
bodies	cds.comp38721_c0_seq1|m.73534	3	3	B4XT43_9ARAC | Myosin II regulatory light chain
bodies	cds.comp27063_c0_seq1|m.52228	3	3	no hit
bodies	cds.comp131296_c0_seq1|m.211134	3	3	no hit
bodies	cds.comp8071_c0_seq1|m.14356	3	1	PEPT1_EURMA | Peptidase 1 EC=3.4.22.65 AltName: Full=Allergen Eur m I AltName: Full=Mite group 1 allergen Eur m 1 AltName: Allergen=Eur m 1 Flags: Precursor
bodies	cds.comp13825_c0_seq1|m.24963	3	3	O96938_CERCA | Acidic ribosomal protein
bodies	cds.comp18202_c0_seq1|m.33963	3	3	B7QHI5_IXOSC | Putative uncharacterized protein
bodies	cds.comp29245_c0_seq1|m.56272	3	3	R4WEH7_9HEMI | Ornithine decarboxylase Flags: Fragment
bodies	cds.comp41848_c0_seq1|m.79332	3	3	no hit
bodies	cds.comp24833_c0_seq1|m.47844	3	3	no hit
bodies	cds.comp26722_c0_seq1|m.51415	3	3	G6D1V5_DANPL | Putative lysosomal alpha-mannosidase
bodies	cds.comp2827_c0_seq1|m.3911	3	3	L7UZ91_DERFA | Ferritin

Bodies/feces	Accession	#Peptides	#Unique	Description
bodies	cds.comp30440_c0_seq1|m.58399	3	3	F0JA41_AMBVA | Na+/K+ ATPase beta subunit
bodies	cds.comp103704_c0_seq1|m.179484	2	2	B7Q5N1_IXOSC | Cuticle protein putative
bodies	cds.comp58673_c0_seq1|m.109408	2	2	A9QQ34_LYCSI | Cysteine and glycine-rich protein
bodies	cds.comp58860_c0_seq1|m.109626	2	2	A0SHR2_AMBVA | Protein disulfide isomerase EC=5.3.4.1
bodies	cds.comp23024_c0_seq1|m.43937	2	2	F1CJ03_HOTJU | Putative muscular protein Flags: Fragment
bodies	cds.comp103380_c0_seq1|m.179115	2	2	L7MGB9_9ACAR | Putative aicar transformylase/imp cyclohydrolase/methylglyoxal synthase Flags: Fragment
bodies	cds.comp141434_c0_seq1|m.217093	2	2	no hit
bodies	cds.comp61843_c0_seq1|m.114882	2	2	B7QAN4_IXOSC | Sorbitol dehydrogenase putative EC=1.1.1.14
bodies	cds.comp101947_c0_seq1|m.176976	2	2	F1CJ08_HOTJU | Hypothetical secreted protein
bodies	cds.comp125677_c0_seq1|m.206230	2	2	B7QH76_IXOSC | Secreted protein putative
bodies	cds.comp71061_c0_seq1|m.130213	2	1	B4YTU0_9ACAR | Heat shock protein 70-3
bodies	cds.comp123134_c0_seq1|m.203950	2	2	L7M5B4_9ACAR | Putative 3-hydroxyacyl-coa dehydrogenase
bodies	cds.comp21412_c0_seq1|m.40677	2	1	B4I114_DROSE | GM12332
bodies	cds.comp21407_c0_seq1|m.40576	2	0	no hit
bodies	cds.comp107883_c0_seq1|m.184877	2	2	no hit
bodies	cds.comp11937_c0_seq1|m.21539	2	2	B4MPF1_DROWI | GK21691
bodies	cds.comp89225_c0_seq1|m.159466	2	2	R4G5K4_RHOPR | Putative adenosylcysteinase
bodies	cds.comp101223_c0_seq1|m.176139	2	2	Q7QIX6_ANOGA | AGAP007120-PA
bodies	cds.comp83490_c0_seq1|m.150466	2	2	L7N6F8_DERPT | Dust mite allergen Flags: Precursor

Bodies/feces	Accession	#Peptides	#Unique	Description
bodies	cds.comp85485_c0_seq1|m.153656	2	1	E1ZZW6_CAMFO | Heat shock 70 kDa protein cognate 4
bodies	cds.comp123877_c0_seq1|m.204692	2	2	E2BIZ1_HARSA | 4-hydroxyphenylpyruvate dioxygenase
bodies	cds.comp125697_c0_seq1|m.206250	2	2	Q17PK8_AEDAE | AAEL000317-PA
bodies	cds.comp53352_c0_seq1|m.100121	2	2	B7P242_IXOSC | Glutathione S-transferase kappa putative
bodies	cds.comp124015_c0_seq1|m.204829	2	2	K7IPQ8_NASVI | Proteasome subunit beta type EC=3.4.25.1
bodies	cds.comp114623_c0_seq1|m.193681	2	2	A1KXH6_DERFA | Der f 8 allergen Flags: Fragment
bodies	cds.comp29495_c0_seq1|m.56671	2	2	L7M6D4_9ACAR | Putative failed axon connections fax protein/glutathione s-transferase-like protein
bodies	cds.comp52873_c0_seq1|m.99306	2	2	A1KXC2_DERFA | DFP2
bodies	cds.comp102913_c0_seq1|m.178487	2	2	Q8ISH5_ARAVE | Chitinase
bodies	cds.comp74116_c0_seq1|m.135879	2	2	Q2YFF0_SARSC | Glutathione transferase mu class Yv5004H11
bodies	cds.comp37378_c0_seq1|m.71331	2	2	L7M1Q0_9ACAR | Putative 14-3-3 protein zeta multifunctional 14-3-3 family chaperone
bodies	cds.comp44453_c0_seq1|m.83958	2	2	R4G324_RHOPR | Putative succinyl-coa synthetase
bodies	cds.comp92426_c0_seq1|m.164426	2	2	I1ZE47_DERPT | Heat shock protein cognate 5 Flags: Fragment
bodies	cds.comp93425_c0_seq1|m.165849	2	2	F4WQI8_ACREC | Actin-interacting protein 1
bodies	cds.comp116532_c0_seq1|m.196277	2	2	L7M765_9ACAR | Dihydrolipoyl dehydrogenase EC=1.8.1.4
bodies	cds.comp102752_c0_seq1|m.178281	2	2	F1DFM4_LIBPU | Projectin long variant Flags: Fragment
bodies	cds.comp78047_c0_seq1|m.141897	2	2	B4MGL9_DROVI | GJ16068
bodies	cds.comp39659_c0_seq1|m.75583	2	2	Q2XW13_RHIMP | Glutathione peroxidase
bodies	cds.comp49835_c0_seq1|m.93452	2	2	B7Q3I2_IXOSC | Citrate synthase Flags: Fragment

Bodies/feces	Accession	#Peptides	#Unique	Description
bodies	cds.comp102436_c0_seq1|m.177792	2	2	L7LZG6_9ACAR | Putative nuclear transport factor-2
bodies	cds.comp123547_c0_seq1|m.204321	2	2	A1KY40_BLOTA | Major abundant protein BTP1
bodies	cds.comp47522_c0_seq1|m.89360	2	2	A9QW25_CARMA | Glycosyl-phosphatidylinositol-linked carbonic anhydrase EC=4.2.1.1
bodies	cds.comp81665_c0_seq1|m.147665	2	2	no hit
bodies	cds.comp116353_c0_seq1|m.196018	2	2	no hit
bodies	cds.comp77279_c0_seq1|m.140672	2	2	A9QQC2_LYCSI | Cofilin
bodies	cds.comp27378_c0_seq1|m.52842	2	2	B5DS37_DROPS | GA27615
bodies	cds.comp99743_c0_seq1|m.173941	2	2	no hit
bodies	cds.comp116361_c0_seq1|m.196024	2	2	B3LWF9_DROAN | GF18656
bodies	cds.comp27523_c0_seq1|m.53169	2	2	B7Q5N2_IXOSC | Cuticle protein putative
bodies	cds.comp7308_c0_seq1|m.12724	2	2	F0J8F6_AMBVA | Metallopeptidase Flags: Fragment
bodies	cds.comp27661_c0_seq1|m.53356	2	2	E2BSG2_HARSA | Aconitate hydratase mitochondrial Flags: Fragment
bodies	cds.comp119852_c0_seq1|m.200270	2	2	no hit
bodies	cds.comp65192_c0_seq1|m.120732	2	2	TBA_LEPDS | Tubulin alpha chain AltName: Allergen=Lep d ?
bodies	cds.comp53422_c0_seq1|m.100213	2	2	B4PC03_DROYA | GE11380
bodies	cds.comp116835_c0_seq1|m.196699	2	1	L7M7I7_9ACAR | Putative alkyl hydroperoxide reductase thiol specific antioxidant
bodies	cds.comp125648_c0_seq1|m.206190	2	2	Q95P83_AMBAM | Gamma-interferon inducible lysosomal thiol reductase Flags: Fragment
bodies	cds.comp8010_c0_seq1|m.14279	2	2	PEPT1_EURMA | Peptidase 1 EC=3.4.22.65 AltName: Full=Allergen Eur m I AltName: Full=Mite group 1 allergen Eur m 1 AltName: Allergen=Eur m 1 Flags: Precursor
bodies	cds.comp140783_c0_seq1|m.216811	2	2	E1ZXQ0_CAMFO | Putative uncharacterized protein

Bodies/feces	Accession	#Peptides	#Unique	Description
bodies	cds.comp115395_c0_seq1|m.194741	2	2	A6N9Y8_ORNPR | 40S ribosomal protein S11
bodies	cds.comp70339_c0_seq1|m.129030	2	2	B7PYU7_IXOSC | Calcium-binding protein putative Flags: Fragment
bodies	cds.comp37354_c0_seq1|m.71307	2	2	G3MN10_9ACAR | Histone H2B
bodies	cds.comp33336_c0_seq1|m.64158	2	2	A8E4K0_9ACAR | Troponin T
bodies	cds.comp35805_c0_seq1|m.69173	2	2	D2SNU8_HELVI | Short-chain dehydrogenase
bodies	cds.comp110061_c0_seq1|m.187587	2	2	L7M4M3_9ACAR | Putative enoyl-coa isomerase
bodies	cds.comp148912_c0_seq1|m.219748	2	2	Q17EW2_AEDAE | AAEL003658-PA
bodies	cds.comp73546_c0_seq1|m.134459	2	2	Q9U1K7_DROME | CG3939 SubName: Full=EG:140G11.5 protein SubName: Full=RH07711p
bodies	cds.comp96326_c0_seq1|m.169658	2	2	L0AT90_COPFO | FK506-binding like protein
bodies	cds.comp25135_c0_seq1|m.48279	2	2	Q16ZT8_AEDAE | AAEL008073-PA
bodies	cds.comp93450_c0_seq1|m.165864	2	2	E0VHS4_PEDHC | 60S ribosomal protein L5 putative
feces	cds.comp116085_c0_seq1|m.195551	29	5	PEPT1_DERPT | Peptidase 1 EC=3.4.22.65 AltName: Full=Allergen Der p I AltName: Full=Major mite fecal allergen Der p 1 AltName: Allergen=Der p 1 Flags: Precursor
feces	Der p 1.0118	29	0	Der p 1.0118
feces	Der p 1.0102	28	0	Der p 1.0102
feces	Der p 1.0108	28	0	Der p 1.0108
feces	Der p 1.0124	27	0	Der p 1.0124
feces	Der p 1.0101	27	0	Der p 1.0101
feces	Der p 1.0103	26	1	Der p 1.0103
feces	Der p 1.0107	25	0	Der p 1.0107

Bodies/feces	Accession	#Peptides	#Unique	Description
feces	Der p 1.0111	23	0	Der p 1.0111
feces	cds.comp31388_c0_seq1|m.60552	22	22	G6DI19_DANPL | Putative cuticle protein CPH35
feces	Der p 1.0112	21	0	Der p 1.0112
feces	Der p 1.0104	21	1	Der p 1.0104
feces	cds.comp86986_c0_seq1|m.155830	21	21	no hit
feces	Der p 6.0101	18	18	Der p 6.0101
feces	cds.comp72134_c0_seq1|m.132054	18	18	DERP3_DERPT | Mite allergen Der p 3 EC=3.4.21.- AltName: Full=Allergen Der p III AltName: Allergen=Der p 3 Flags: Precursor
feces	Der p 2.0106	16	0	Der p 2.0106
feces	Der p 2.0113	16	0	Der p 2.0113
feces	Der p 2.0112	16	0	Der p 2.0112
feces	cds.comp4299_c0_seq1|m.6626	16	3	E2A599_CAMFO | Lysosomal alpha-glucosidase
feces	Der p 2.0107	15	0	Der p 2.0107
feces	Der p 2.0105	15	0	Der p 2.0105
feces	Der p 2.0102	15	0	Der p 2.0102
feces	Der p 2.0103	15	0	Der p 2.0103
feces	Der p 2.0109	15	0	Der p 2.0109
feces	Der p 2.0104	15	0	Der p 2.0104
feces	Der p 2.0111	15	0	Der p 2.0111
feces	cds.comp86167_c0_seq1|m.154658	15	3	R4FNL2_RHOPR | Putative cathepsin b-like proteinase

Bodies/feces	Accession	#Peptides	#Unique	Description
feces	Der p 4.0101	15	15	Der p 4.0101
feces	cds.comp117244_c0_seq1|m.197138	14	14	ALL2_PSOOV | Mite group 2 allergen Pso o 2 AltName: Full=Allergen Pso o A AltName: Allergen=Pso o 2 Flags: Precursor
feces	cds.comp112920_c0_seq1|m.190979	14	12	Der p 36
feces	cds.comp86168_c0_seq1|m.154662	13	0	R4FNL2_RHOPR | Putative cathepsin b-like proteinase
feces	cds.comp23278_c0_seq1|m.44326	13	13	Q8MWR6_DERPT | 14.5 kDa bacteriolytic enzyme
feces	cds.comp134821_c0_seq1|m.213456	13	13	Q7Z163_DERPT | Trypsin-like serine protease
feces	cds.comp86162_c0_seq1|m.154650	12	11	D6WGZ1_TRICA | Cathepsin B
feces	cds.comp136643_c0_seq1|m.214661	12	12	E2C2J9_HARSA | Pancreatic triacylglycerol lipase
feces	cds.comp57250_c0_seq1|m.106674	12	2	Q4JK69_DERPT | Group 15 allergen protein short isoform
feces	Der p 15.0102	12	2	Der p 15.0102
feces	cds.comp4288_c0_seq1|m.6615	12	0	F4WH36_ACREC | Lysosomal alpha-glucosidase
feces	cds.comp122816_c0_seq1|m.203611	11	11	C1IE32_9HEXA | Beta-1 3-D-glucanase EC=3.2.1.6 SubName: Full=Endo-beta-1 3-glucanase EC=3.2.1.39 Flags: Precursor
feces	Der p 15.0101	11	1	Der p 15.0101
feces	cds.comp71148_c0_seq1|m.130327	9	9	Q4JK71_DERPT | Group 18 allergen protein
feces	cds.comp83490_c0_seq1|m.150466	7	7	L7N6F8_DERPT | Dust mite allergen Flags: Precursor
feces	cds.comp4304_c0_seq1|m.6632	7	6	E2A599_CAMFO | Lysosomal alpha-glucosidase
feces	cds.comp8012_c0_seq1|m.14281	7	1	PEPT1_EURMA | Peptidase 1 EC=3.4.22.65 AltName: Full=Allergen Eur m I AltName: Full=Mite group 1 allergen Eur m 1 AltName: Allergen=Eur m 1 Flags: Precursor
feces	cds.comp11084_c0_seq1|m.19766	7	7	F4W8Y5_ACREC | Lysosomal alpha-mannosidase
feces	cds.comp26722_c0_seq1|m.51415	7	7	G6D1V5_DANPL | Putative lysosomal alpha-mannosidase

Bodies/feces	Accession	#Peptides	#Unique	Description
feces	cds.comp8057_c0_seq1|m.14337	6	0	PEPT1_EURMA | Peptidase 1 EC=3.4.22.65 AltName: Full=Allergen Eur m I AltName: Full=Mite group 1 allergen Eur m 1 AltName: Allergen=Eur m 1 Flags: Precursor
feces	cds.comp8071_c0_seq1|m.14356	6	2	PEPT1_EURMA | Peptidase 1 EC=3.4.22.65 AltName: Full=Allergen Eur m I AltName: Full=Mite group 1 allergen Eur m 1 AltName: Allergen=Eur m 1 Flags: Precursor
feces	cds.comp128133_c0_seq1|m.208437	5	5	C1IE32_9HEXA | Beta-1 3-D-glucanase EC=3.2.1.6 SubName: Full=Endo-beta-1 3-glucanase EC=3.2.1.39 Flags: Precursor
feces	cds.comp8014_c0_seq1|m.14284	5	3	PEPT1_EURMA | Peptidase 1 EC=3.4.22.65 AltName: Full=Allergen Eur m I AltName: Full=Mite group 1 allergen Eur m 1 AltName: Allergen=Eur m 1 Flags: Precursor
feces	cds.comp112920_c0_seq1|m.190978	5	5	B7PB45_IXOSC | Transferrin receptor putative EC=3.4.11.10
feces	cds.comp142310_c0_seq1|m.217545	4	4	Q8MRY3_DROME | SD13780p
feces	cds.comp129013_c0_seq1|m.209205	4	4	B4JYH8_DROGR | GH14296
feces	cds.comp27091_c0_seq1|m.52288	4	0	no hit
feces	cds.comp27090_c0_seq1|m.52286	4	0	no hit
feces	cds.comp67043_c0_seq1|m.123479	4	3	I3TBA6_CRYPU | Heat shock protein 70
feces	cds.comp62837_c0_seq1|m.116676	4	4	A0NBF1_ANOGA | AGAP007368-PA
feces	cds.comp11404_c0_seq1|m.20428	3	3	A5X5X4_DERFA | Allergen SubName: Full=Group 22 allergen Der f 22
feces	cds.comp4302_c0_seq1|m.6630	3	1	E2A599_CAMFO | Lysosomal alpha-glucosidase
feces	cds.comp11937_c0_seq1|m.21539	3	3	B4MPF1_DROWI | GK21691
feces	cds.comp141434_c0_seq1|m.217093	3	3	no hit
feces	cds.comp15337_c0_seq1|m.28440	3	3	Q2YFE4_DERPT | Glutathione transferase mu class Dp7002H05
feces	cds.comp112914_c0_seq1|m.190966	2	0	no hit
feces	cds.comp86983_c0_seq1|m.155817	2	2	no hit
feces	cds.comp41848_c0_seq1|m.79332	2	2	no hit

Bodies/feces	Accession	#Peptides	#Unique	Description
feces	cds.comp71097_c0_seq1|m.130255	2	1	B4YTT9_9ACAR | Heat shock protein 70-2
feces	cds.comp8015_c0_seq1|m.14285	2	0	PEPT1_EURMA | Peptidase 1 EC=3.4.22.65 AltName: Full=Allergen Eur m I AltName: Full=Mite group 1 allergen Eur m 1 AltName: Allergen=Eur m 1 Flags: Precursor
feces	cds.comp131296_c0_seq1|m.211134	2	2	no hit
feces	cds.comp114213_c0_seq1|m.193079	2	2	Q9U6R7_DERFA | 98kDa HDM allergen SubName: Full=Group 15 allergen Der f 15
feces	cds.comp58995_c0_seq1|m.109762	2	2	E2BUA9_HARSA | CD151 antigen
feces	cds.comp134947_c0_seq1|m.213562	2	2	E0VLI0_PEDHC | Purple acid phosphatase putative
feces	cds.comp9920_c0_seq1|m.17791	2	2	Q0KKA6_HAELO | Leucine aminopeptidase
feces	cds.comp17633_c0_seq1|m.32892	2	2	B7Q5N0_IXOSC | Cuticle protein putative
feces	cds.comp70394_c0_seq1|m.129110	2	2	A1KXH8_DERFA | Der f Gal d 2 allergen
feces	cds.comp125697_c0_seq1|m.206250	2	2	Q17PK8_AEDAE | AAEL000317-PA
feces	cds.comp99743_c0_seq1|m.173941	2	2	no hit
feces	cds.comp25833_c0_seq1|m.49774	2	2	no hit
