# Supplementary material for: A combined transcriptome and proteome analysis extends the allergome of house dust mite Dermatophagoides species
Source: PLoS One. 2017 Oct 5;12(10):e0185830. doi: 10.1371/journal.pone.0185830 (PMC5628879; doi:10.1371/journal.pone.0185830)
Supplement: S3 Table — Counterparts in either D. farinae or D. pteronyssinus of IUIS-recorded allergens were identified in transcriptome-derived databases using blast analyses. (PDF) [file pone.0185830.s006.pdf]

Organism	Name	Accession number	Protein family	Complete/Partial	% identity (pairwise)	Sequence
D. farinae	Der p 5-like	MF346040	Unknown	Partial	96.7% (Der p 5.0101)	VVCVSGEPKKHDYQNEFDFLLMQRIHEQMRKGEEALLHLQHQINTFEE NPTKEMKEQILGEMDTIIALIDGVRGVLNRLMKRTDLDIFERYNVEIALKS NEILERDLKKEEQRVKKIEV
D. farinae	Der p 9-like	MF346041	Collagenolytic serine protease	Partial	84.5% (Der p 9.0102) 96.9% (AIO08869)	MKFATIFVLIAIGTSVVIGDHSEEQAIRLPLPKAINERFPWMINEPITNGE RIVGGENASPGDAIYQIALLRKDSFTCGGSLISSRTVLTAAHCVFGDEA SPSYFKIRYNTLDRTNGPTIGVSKIYRHSLYSSTTIDYDVATLILSEPFTP SANADIISL
D. farinae	Der p 23-like	MF346042	Peritrophin-like protein	Partial	84% (Der p 23.0101)	EQPEDEFECPTRFGYFADPKDPCKFYICSNWEAIHKSCPGNTRWNEK ELTC
D. pteronyssinus	Der f 16-like	MF346043	Gelsolin/villin	Partial	89.8% (Der f 16.0101)	IWRVKQFELVPVPKEEYGKFYKGDCYIVICCTDTPTGGHSKMESKPILN GHGYCHIHYWIGSESTKDEAGVAAIKSVELDDFLGGYPVQHREIEEFES RQFSSYFKDGIIYLKGGYESGFTKMIDELKPTLLHVKGKKRPIVYECPAI SWKSMNDGDVFILIVPKFIFVWTGKHSNRMERTTAIRVANDLKSQLNRF KLSTVILEDGKEVEQTSGAEYDAFNKALPLDKQDTDLKKLPKGYDYAAC DIKFEKHERSFVTLYKCFEGTETIDISFVKNGPLTRKDLDTNDTFIVENG SEGLWVWVGKKATQKERQSAIKYAMELINKKKYPNNTPVTKVLEGDET ADFKSLFESWQMTEQEKITSARLFRVSRNGIFKLVANYEQDDLEEDNV MILDAIDKIYVWIGNQFKERIADEKHVDILAERFIQEDKSGRKFQPNQILK IKQGSEDATFKSYFPKWK
D. pteronyssinus	Der f 22-like	MF346044		Complete	71.8% (Der f 22.0101)	MFCFLKMNQHTACLALFCLIMMVAVQANDETNVQYKDCGHNEIKSLYL SGCNVHQKSCILHRHNKNQLRLGFVANENTGKTIKTRFICNLAGLEVG WPGIDGTDACQGHGLSCPLTKGQSYNYNLDFSLGDDVPLVNVTATVR LEDEQGGKLACARMHISLQA

Organism	Name	Accession number	Protein family	Complete/Partial	% identity (pairwise)	Sequence
D. pteronyssinus	Der f 25-like	MF346045	Triosephosphate isomerase	Complete	91.9%<br>(Der f 25.0201)	MGRKFFVGGNWKMNGSRATNDDLIKILTNTPLDPNTEVVVGVPSIYLA<br>DVRHKLSKSIGVAAQNCYKVAKGAFTGEISPAMIKDVGAEWVILGHSE<br>RRNVFGENDQLIGEKVEHALNEGLNVIACIGELFEEREAGKTTEVVFRQ<br>TQVISKHVKDWSKVVLAYEPVWAIGTGKTASPQQAQEVHQQLRQWFS<br>TNISPQIAETIRIIYGGSVTASNAKELASQADVDGFLVGGASLKPEFVQIV<br>NARQ
D. pteronyssinus	Der f 26-like	MF346046	Myosin alkali light chain	Complete	93.1%<br>(Der f 26.0101)	MADLRPPEVEKARLHFDIYDFEGQGKVDYHHLGDLLRSLDLRPTQEIV<br>AKNGWEKKKGQKYMTFEEFLPIYSQVKKDKDCGAYEDLVEGLKVYDK<br>AENGTMMEAELAHVLLSLGEKLDDSEVEEIIKTCAGGCDDEGFIKYETFI<br>KNVMAGPFPEEAGKS
D. pteronyssinus	Der f 27-like	MF346047	Serpin	Complete	71.2%<br>(Der f 27.0101)	MKIFITSFVLMMVMAVTYAAHVGSHSRKPASAEGFAKASNDFGFGLLK<br>QIVQHQSSSSSGSKNVLFSPYSVAVALSMVHQGSQGSTAEQFKRVLN<br>YDQVSQLNNGEHQAVANSVKQLREQMTKSEHNKLEYGNMLVVDHKM<br>PIKDEYRKTIEQYYDGQVMSVDFPKESKNIMEQINQYISNKTHGLIDRML<br>EQPPSSGTVLALINAVYFKGEWLKPFIPSNTVQGVFYDHQGHEYKNVE<br>YMNGEGPFGYAEIKQWNSDLIKMPYKGEDVAFYGILPRERNFDLSKIR<br>QSLNSSYIDEIVGQINDDQSSTVYFPKIELSTSYELPEILKSMGIQDVFTE<br>SADLSGISGNKSMKIDEAIHKAKLIVNEQGTEAAASTYIQIALMSSLENQ<br>KFSVLIIHFCILFVHQTTGQILFLGEIHNF

Organism	Name	Accession number	Protein family	Complete/Partial	% identity (pairwise)	Sequence
D. pteronyssinus	Der f 28-like	MF346048	Heat Shock Protein	Complete	95.9% (Der f 28.0201)	MPSKTSKAPAIGIDLGTTYSCVGVFQNGSVEIIANDQGNRTTPSYVAFTDTERLIGDAAKNQVAMNPANTIFDAKRLIGRRFDESSVKSDMKHWPFKVVSESGKPKIEVEFKGEQKRFWPEEVSAMVLTKMKETAEAYLGQKVTDAVITVPAYFNDSQRQATKDAGVIAGLNVLRIINEPTAAAIAYGLDKKGGGEKNVLIFDLGGGTFDVSVLTIDNGIFEVKSTAGDTHLGGEDFDNRLVNHFVQEFKRKFGKDIMSNKRALRRLRTACERAKRTLSSSTQTTIEIDSLHEGTDFYSTITRARFEELCSDLFRSTLEPVEKALRDAKLDKSKIDEIVLVGGSTRIPKIQKLLSDFFNGKELNKSINPDEAVAYGAAVQAAILTGDTSNNVKDLLLLDVAPLSLGIETAGGVMTTLIKRNTTIPTKQTQTFTTYADNQPAVTIQVYEGERAMTKDNNRLGTFDLTGIPPAPRGVPQIEVTFDVDANGILNVSAVDKSTGRQNKITITNDKGRLSKADIEKMVNEAEQYREEDERQRERISAKNQLEAYAFQLKSTMEEEAIKSKLSEEDRKTVLNKVEETLRWLDSNQLADKEEYEHRQKELESACRPIMTKIYQQQQQQHPGAPGSNGSCGSNAYPGYGGFNSNNDGPVVEEVN
D. pteronyssinus	Der f 29-like	MF346049	Peptidyl-prolyl cis-trans isomerase (cyclophilin)	Complete	93.3% (Der f 29.0101)	MALPRVYFDVAADNQPLGRIVMELRSDVVPKTAENFRCLCTGEKGFGFKSSSFHRIIPNFMIQGGDFTNHNGTGGKSIYGNKFADENFTLQHTGPGILSMANAGPNTNGSQFFLTTVKTSWLDGKHVVFGSVVDGMDIVKKIESYGSQSGKPSKKVTISDCGQL
D. pteronyssinus	Der f 30-like	MF346050	Ferritin	Complete	80.8% (Der f 30.0101)	MAANPESTTKTSRVRMNFHKECEAGINKQINLELYASYVYQQMAFHFNREDVALPGFEKFFHESSKEEREHAEKLMKLQNQRGGRIVLQDIPKPVQQDWSSGLEALKASLELEKTVNQSLLDLHDLATKHNDAQFADFIESNYLHEQVEAIKKLADYITNLERCGSVGLGEYLFDRHTLQ
D. pteronyssinus	Der f 31-like	MF346051	Cofilin	Complete	100% (Der f 31.0101)	MASGVTVATEAKTLYEEVKKDKKYRYIIYHIKDERVIEVETTGPRDATYSDFVAKLQDYKNECRYCVFDFPANIPVEGGGEKSNMSVDRLVLMTWCPESAKIKQKMLYSSSYDALKKALVGVYKYVQACDYEEASEEAIAEAFRKGGK

Organism	Name	Accession number	Protein family	Complete/Partial	% identity (pairwise)	Sequence
D. pteronyssinus	Der f 32-like	MF346052	Secreted inorganic pyrophosphatase	Partial	91.9% (Der f 32.0101)	MVVEIPRWTNEKMEIATAEPMTPIKQDVKKGALRYVKNVFPHKGYIWN YGAFPQTWENPNHIDQGTKAKGDNDPIDVIEIGSRIAKRGDVIPVKILGTI ALIDEGETDWKIITIDTRDELAGQMNNVDDVEKLLPGLLRATVEWFRIYK IPDGKPANKFAFNGEAKDREFAEKVVEETHQYWREMMENKAGEHQLD LKNITLGNSYTINDEQAKQFLETRPASNTVEPNPIADQVAIDKWHHVKLI
D. pteronyssinus	Der f 33-like	MF346053	Alpha-tubulin	Complete	93.3% (Der f 33.0101)	MRECISLHVGQAGVQIGNACWELYCLEHGIQPDGILSPIDSTTSTTESS LSSNDSFSTFFNETGSGHYVPRSIYVDLEPTVVDEVRTGEYRRLFHPE QLITGKEDAANNYARGHYTEGKALIEPVMQRIAKLAEQCSGLQGFLIFH SFGGGTGSGFSSLLMERLSVEYGKKSKLEFAIYPAPAISTAVVEPYNSIL TTHNTLEHSDCSFMVDNEAIYDICRRNLNIERPLYMNLNRMIGQIVSSIT ASLRFDGALNVDLTEFQTNLVPYPRIHFPLVSYAPIVSSEKAYHEQFSV AEITGTCFEPSNQMVKCNTRNGKFMACCLLYRGDVVPKEVNAAIAAIKA KRTIQFVDWCPTGFKIGINYRPPTVVPNGDQAKVQRAVCLLSNTTAIAE AWSRLNHKFDLMYSKRAFVHWYVGEGMEEGEFSEAREDLAALERDY EEVAAEYNADDDDDHDQDGEEF
D. pteronyssinus	Der f 34-like	MF346054	Imine deaminase	Complete	77.3% (Der f 34.0101)	MSPRRILSTPLAPMPIGPYSQAVQVGNQIYLSGQLGMNVRTGELLTESI QAEARQAFNNMKAVVESGGARMSDIVKLTIFIRDFNDFPTINDVMKEFF QSPFPARSTIGVASLPKNARIELECIAVIE
D. pteronyssinus	Der f 35-like	MF346055	MD-2−like	Complete	85.3% (Der f 35.0101)	MMKFLCIFALTFAVASAGKMKFIDCGHNEVKSLDVSGCEGDYCILHKGK TINLDMVCKSNQDSEHLKLVISADVNGIEIEVPGFDQDGCHYIQCPVHK GQQYDVKYSYTIPSVLPNIKANLTAKVTGDQGLLGCLKLQGEIAD
