# Supplementary material for: A combined transcriptome and proteome analysis extends the allergome of house dust mite Dermatophagoides species
Source: PLoS One. 2017 Oct 5;12(10):e0185830. doi: 10.1371/journal.pone.0185830 (PMC5628879; doi:10.1371/journal.pone.0185830)
Supplement: S4 Table — A protein extract from whole D. farinae culture was submitted to two-dimensional gel electrophoresis. After staining with Sypro Ruby, gel plugs from 94 protein spots (S1 Fig) were recovered, trypsin digested then analyzed by LC-MS/MS. Numbers refer to the IgE-reactive (red cell) and non IgE-reactive (green cell) spots analyzed. Protein identification was performed using the species-specific transcriptome derived protein database, supplemented with IUIS-registered allergen sequences, as reference dataset. Only entries identified by a minimum of 2 peptides sequenced were taken into account. Proteins are reported by the entry name with the total numbers of supporting sequenced peptides (#peptides) and of uniquely mapping peptides (#unique) as well as, when available, the result of the annotation by blast analysis. (PDF) [file pone.0185830.s007.pdf]

Spot number	Accession	#Peptides	#Unique	Description
1	Der f 15.0101	21	21	Der f 15.0101
1	cds.comp140939_c0_seq1|m.49098	7	7	Q178W0_AEDAE | AAEL005752-PA
1	cds.comp102032_c0_seq1|m.28404	7	7	no hit
1	cds.comp32307_c0_seq1|m.7704	6	6	F4W8Y5_ACREC | Lysosomal alpha-mannosidase
1	Der f 1.0104	4	4	Der f 1.0104
1	cds.comp7296_c0_seq1|m.1765	4	4	E2BEF1_HARSA | Lysosomal alpha-glucosidase Flags: Fragment
1	cds.comp32305_c0_seq1|m.7703	3	3	E2ABS8_CAMFO | Lysosomal alpha-mannosidase
1	cds.comp140939_c0_seq1|m.49097	3	3	Q5TS83_ANOGA | AGAP008584-PA
1	cds.comp113572_c0_seq1|m.37601	2	2	B7PDF5_IXOSC | Prolyl endopeptidase putative EC=3.4.21.26
1	cds.comp10022_c0_seq1|m.2407	2	2	E2A599_CAMFO | Lysosomal alpha-glucosidase
2	Der f 15.0101	20	20	Der f 15.0101
2	cds.comp140939_c0_seq1|m.49098	5	5	Q178W0_AEDAE | AAEL005752-PA
2	Der f 1.0104	4	4	Der f 1.0104
2	cds.comp32305_c0_seq1|m.7703	3	3	E2ABS8_CAMFO | Lysosomal alpha-mannosidase
2	cds.comp7296_c0_seq1|m.1765	3	3	E2BEF1_HARSA | Lysosomal alpha-glucosidase Flags: Fragment
2	cds.comp140939_c0_seq1|m.49097	3	3	Q5TS83_ANOGA | AGAP008584-PA
2	cds.comp113572_c0_seq1|m.37601	2	2	B7PDF5_IXOSC | Prolyl endopeptidase putative EC=3.4.21.26
2	cds.comp32307_c0_seq1|m.7704	2	2	F4W8Y5_ACREC | Lysosomal alpha-mannosidase
2	cds.comp10022_c0_seq1|m.2407	2	2	E2A599_CAMFO | Lysosomal alpha-glucosidase

Spot number	Accession	#Peptides	#Unique	Description
2	cds.comp102032_c0_seq1|m.28404	2	2	no hit
3	Der f 15.0101	25	25	Der f 15.0101
3	cds.comp140939_c0_seq1|m.49098	7	7	Q178W0_AEDAE | AAEL005752-PA
3	cds.comp32307_c0_seq1|m.7704	5	5	F4W8Y5_ACREC | Lysosomal alpha-mannosidase
3	cds.comp7296_c0_seq1|m.1765	5	5	E2BEF1_HARSA | Lysosomal alpha-glucosidase Flags: Fragment
3	cds.comp140939_c0_seq1|m.49097	5	5	Q5TS83_ANOGA | AGAP008584-PA
3	cds.comp10022_c0_seq1|m.2407	5	5	E2A599_CAMFO | Lysosomal alpha-glucosidase
3	Der f 1.0104	4	4	Der f 1.0104
3	cds.comp102032_c0_seq1|m.28404	3	3	no hit
3	cds.comp113572_c0_seq1|m.37601	2	2	B7PDF5_IXOSC | Prolyl endopeptidase putative EC=3.4.21.26
3	cds.comp32305_c0_seq1|m.7703	2	2	E2ABS8_CAMFO | Lysosomal alpha-mannosidase
3	cds.comp113569_c0_seq1|m.37594	2	2	E1U339_9HEMI | Prolyl-endylpeptidase Flags: Fragment
4	cds.comp140939_c0_seq1|m.49098	10	10	Q178W0_AEDAE | AAEL005752-PA
4	cds.comp32307_c0_seq1|m.7704	7	7	F4W8Y5_ACREC | Lysosomal alpha-mannosidase
4	cds.comp140939_c0_seq1|m.49097	7	7	Q5TS83_ANOGA | AGAP008584-PA
4	cds.comp32305_c0_seq1|m.7703	4	4	E2ABS8_CAMFO | Lysosomal alpha-mannosidase
4	cds.comp13305_c0_seq1|m.3171	2	2	B3TFG6_9ACAR | Esterase TCE1
5	cds.comp140939_c0_seq1|m.49098	10	10	Q178W0_AEDAE | AAEL005752-PA
5	cds.comp140939_c0_seq1|m.49097	7	7	Q5TS83_ANOGA | AGAP008584-PA

Spot number	Accession	#Peptides	#Unique	Description
5	cds.comp32307_c0_seq1|m.7704	6	6	F4W8Y5_ACREC | Lysosomal alpha-mannosidase
5	cds.comp32305_c0_seq1|m.7703	4	4	E2ABS8_CAMFO | Lysosomal alpha-mannosidase
5	cds.comp13305_c0_seq1|m.3171	2	2	B3TFG6_9ACAR | Esterase TCE1
6	cds.comp140939_c0_seq1|m.49098	10	10	Q178W0_AEDAE | AAEL005752-PA
6	cds.comp140939_c0_seq1|m.49097	7	7	Q5TS83_ANOGA | AGAP008584-PA
6	cds.comp32307_c0_seq1|m.7704	6	6	F4W8Y5_ACREC | Lysosomal alpha-mannosidase
6	cds.comp32305_c0_seq1|m.7703	5	5	E2ABS8_CAMFO | Lysosomal alpha-mannosidase
7	Der f 1.0104	7	7	Der f 1.0104
7	cds.comp10022_c0_seq1|m.2407	6	6	E2A599_CAMFO | Lysosomal alpha-glucosidase
7	cds.comp85702_c0_seq1|m.20075	5	5	Q5TRG5_ANOGA | AGAP005728-PA
7	cds.comp53900_c0_seq1|m.13006	4	1	Q9U6R7_DERFA | 98kDa HDM allergen SubName: Full=Group 15 allergen Der f 15
7	cds.comp7296_c0_seq1|m.1765	3	3	E2BEF1_HARSA | Lysosomal alpha-glucosidase Flags: Fragment
7	Der f 15.0101	3	0	Der f 15.0101
7	cds.comp113572_c0_seq1|m.37601	2	2	B7PDF5_IXOSC | Prolyl endopeptidase putative EC=3.4.21.26
7	cds.comp113569_c0_seq1|m.37594	2	2	E1U339_9HEMI | Prolyl-endylpeptidase Flags: Fragment
8	Der f 1.0104	8	8	Der f 1.0104
8	Der f 15.0101	5	5	Der f 15.0101
8	cds.comp10022_c0_seq1|m.2407	4	4	E2A599_CAMFO | Lysosomal alpha-glucosidase
8	cds.comp7296_c0_seq1|m.1765	3	3	E2BEF1_HARSA | Lysosomal alpha-glucosidase Flags: Fragment

Spot number	Accession	#Peptides	#Unique	Description
8	cds.comp113572_c0_seq1|m.37601	2	2	B7PDF5_IXOSC | Prolyl endopeptidase putative EC=3.4.21.26
8	cds.comp128012_c0_seq1|m.44522	1	1	B7P3P0_IXOSC | Cathepsin B endopeptidase putative EC=3.4.22.1
8	cds.comp112368_c0_seq1|m.36667	1	1	Q155V8_DERFA | Der f 6 Flags: Fragment
8	Der f 3.0101	1	1	Der f 3.0101
8	cds.comp77465_c0_seq1|m.18227	1	1	L7M6N0_9ACAR | Putative eukaryotic translation initiation factor
8	cds.comp111232_c0_seq1|m.35846	1	1	no hit
8	cds.comp88935_c0_seq1|m.20801	0	0	B4L9W0_DROMO | GI14170
8	cds.comp106051_c0_seq1|m.32245	0	0	L7M646_9ACAR | Putative aconitase/aconitase aconitase superfamily
8	cds.comp154024_c0_seq1|m.51879	0	0	B7PN16_IXOSC | Alpha-mannosidase putative EC=3.2.1.113
8	cds.comp113639_c0_seq1|m.37633	0	0	R4WE33_9HEMI | Mitochondrial ribosomal protein S2
8	cds.comp79845_c0_seq1|m.18841	0	0	B4N9S3_DROWI | GK11459
8	cds.comp75834_c0_seq1|m.17858	0	0	Q7Q9S3_ANOGA | AGAP005070-PA
8	cds.comp81310_c0_seq1|m.19166	0	0	L7M3H9_9ACAR | Putative long-chain acyl-coa synthetase amp-forming
8	cds.comp115925_c0_seq1|m.38916	0	0	Q16GJ6_AEDAE | AAEL014356-PA
8	cds.comp98716_c0_seq1|m.25337	0	0	Q963G2_RHYAM | Histone H1
9	cds.comp7296_c0_seq1|m.1765	10	10	E2BEF1_HARSA | Lysosomal alpha-glucosidase Flags: Fragment
9	Der f 1.0104	10	10	Der f 1.0104
9	cds.comp10022_c0_seq1|m.2407	9	9	E2A599_CAMFO | Lysosomal alpha-glucosidase
10	Der f 15.0101	21	3	Der f 15.0101

Spot number	Accession	#Peptides	#Unique	Description
10	cds.comp53900_c0_seq1|m.13006	19	1	Q9U6R7_DERFA | 98kDa HDM allergen SubName: Full=Group 15 allergen Der f 15
10	cds.comp111232_c0_seq1|m.35846	17	17	no hit
10	cds.comp7296_c0_seq1|m.1765	8	8	E2BEF1_HARSA | Lysosomal alpha-glucosidase Flags: Fragment
10	cds.comp10022_c0_seq1|m.2407	8	8	E2A599_CAMFO | Lysosomal alpha-glucosidase
10	Der f 1.0104	4	4	Der f 1.0104
10	cds.comp119114_c0_seq1|m.40635	4	4	L7M2B8_9ACAR | Putative n-acylaminoacyl-peptide hydrolase
10	cds.comp113572_c0_seq1|m.37601	3	3	B7PDF5_IXOSC | Prolyl endopeptidase putative EC=3.4.21.26
10	cds.comp113569_c0_seq1|m.37594	3	3	E1U339_9HEMI | Prolyl-endylpeptidase Flags: Fragment
10	cds.comp50907_c0_seq1|m.12297	3	3	L7LXZ6_9ACAR | Beta-hexosaminidase EC=3.2.1.52
10	cds.comp85702_c0_seq1|m.20075	2	2	Q5TRG5_ANOGA | AGAP005728-PA
11	Der f 15.0101	21	3	Der f 15.0101
11	cds.comp111232_c0_seq1|m.35846	20	20	no hit
11	cds.comp53900_c0_seq1|m.13006	19	1	Q9U6R7_DERFA | 98kDa HDM allergen SubName: Full=Group 15 allergen Der f 15
11	cds.comp10022_c0_seq1|m.2407	8	8	E2A599_CAMFO | Lysosomal alpha-glucosidase
11	cds.comp7296_c0_seq1|m.1765	7	7	E2BEF1_HARSA | Lysosomal alpha-glucosidase Flags: Fragment
11	Der f 1.0104	4	4	Der f 1.0104
11	cds.comp112615_c0_seq1|m.36876	4	4	G6CKF6_DANPL | Putative prolyl endopeptidase isoform 1
11	cds.comp50907_c0_seq1|m.12297	3	3	L7LXZ6_9ACAR | Beta-hexosaminidase EC=3.2.1.52
11	cds.comp119114_c0_seq1|m.40635	3	3	L7M2B8_9ACAR | Putative n-acylaminoacyl-peptide hydrolase

Spot number	Accession	#Peptides	#Unique	Description
11	cds.comp113572_c0_seq1|m.37601	2	2	B7PDF5_IXOSC | Prolyl endopeptidase putative EC=3.4.21.26
11	cds.comp113569_c0_seq1|m.37594	2	2	E1U339_9HEMI | Prolyl-endylpeptidase Flags: Fragment
11	Der f 3.0101	2	2	Der f 3.0101
11	cds.comp581_c0_seq1|m.236	2	2	Q7PQ97_ANOGA | AGAP004394-PA
12	Der f 15.0101	23	3	Der f 15.0101
12	cds.comp53900_c0_seq1|m.13006	21	1	Q9U6R7_DERFA | 98kDa HDM allergen SubName: Full=Group 15 allergen Der f 15
12	cds.comp111232_c0_seq1|m.35846	18	18	no hit
12	cds.comp10022_c0_seq1|m.2407	10	10	E2A599_CAMFO | Lysosomal alpha-glucosidase
12	cds.comp7296_c0_seq1|m.1765	9	9	E2BEF1_HARSA | Lysosomal alpha-glucosidase Flags: Fragment
12	Der f 1.0104	4	4	Der f 1.0104
12	cds.comp13305_c0_seq1|m.3171	2	2	B3TFG6_9ACAR | Esterase TCE1
12	cds.comp113572_c0_seq1|m.37601	2	2	B7PDF5_IXOSC | Prolyl endopeptidase putative EC=3.4.21.26
13	cds.comp7296_c0_seq1|m.1765	10	10	E2BEF1_HARSA | Lysosomal alpha-glucosidase Flags: Fragment
13	cds.comp10022_c0_seq1|m.2407	9	9	E2A599_CAMFO | Lysosomal alpha-glucosidase
13	cds.comp119114_c0_seq1|m.40635	6	6	L7M2B8_9ACAR | Putative n-acylaminoacyl-peptide hydrolase
13	cds.comp53871_c0_seq1|m.12995	5	5	B4K6G9_DROMO | GI10435
13	cds.comp99324_c0_seq1|m.26033	3	3	A1KXH2_DERFA | Der f 1 allergen
13	cds.comp128018_c0_seq1|m.44542	2	2	B7P3P0_IXOSC | Cathepsin B endopeptidase putative EC=3.4.22.1
13	cds.comp53900_c0_seq1|m.13006	2	2	Q9U6R7_DERFA | 98kDa HDM allergen SubName: Full=Group 15 allergen Der f 15

Spot number	Accession	#Peptides	#Unique	Description
13	cds.comp108775_c0_seq1|m.34394	2	2	D6WCN1_TRICA | Beta-galactosidase EC=3.2.1.23
13	cds.comp111232_c0_seq1|m.35846	2	2	no hit
14	cds.comp10022_c0_seq1|m.2407	11	11	E2A599_CAMFO | Lysosomal alpha-glucosidase
14	cds.comp7296_c0_seq1|m.1765	7	7	E2BEF1_HARSA | Lysosomal alpha-glucosidase Flags: Fragment
14	cds.comp119114_c0_seq1|m.40635	7	7	L7M2B8_9ACAR | Putative n-acylaminoacyl-peptide hydrolase
14	cds.comp99324_c0_seq1|m.26033	3	3	A1KXH2_DERFA | Der f 1 allergen
14	cds.comp108775_c0_seq1|m.34394	3	3	D6WCN1_TRICA | Beta-galactosidase EC=3.2.1.23
14	cds.comp53900_c0_seq1|m.13006	2	2	Q9U6R7_DERFA | 98kDa HDM allergen SubName: Full=Group 15 allergen Der f 15
15	cds.comp7296_c0_seq1|m.1765	11	11	E2BEF1_HARSA | Lysosomal alpha-glucosidase Flags: Fragment
15	cds.comp10022_c0_seq1|m.2407	11	11	E2A599_CAMFO | Lysosomal alpha-glucosidase
15	cds.comp108775_c0_seq1|m.34394	3	3	D6WCN1_TRICA | Beta-galactosidase EC=3.2.1.23
15	cds.comp99324_c0_seq1|m.26033	2	2	A1KXH2_DERFA | Der f 1 allergen
15	cds.comp140939_c0_seq1|m.49098	2	2	Q178W0_AEDAE | AAEL005752-PA
15	cds.comp129544_c0_seq1|m.45265	2	2	Q9Y196_EURMA | Alpha-amylase Flags: Precursor
15	Der f 15.0101	2	2	Der f 15.0101
15	cds.comp119114_c0_seq1|m.40635	2	2	L7M2B8_9ACAR | Putative n-acylaminoacyl-peptide hydrolase
15	cds.comp53871_c0_seq1|m.12995	2	2	B4K6G9_DROMO | GI10435
16	cds.comp7296_c0_seq1|m.1765	12	12	E2BEF1_HARSA | Lysosomal alpha-glucosidase Flags: Fragment
16	cds.comp10022_c0_seq1|m.2407	10	10	E2A599_CAMFO | Lysosomal alpha-glucosidase

Spot number	Accession	#Peptides	#Unique	Description
16	cds.comp119114_c0_seq1|m.40635	4	4	L7M2B8_9ACAR | Putative n-acylaminoacyl-peptide hydrolase
16	cds.comp108775_c0_seq1|m.34394	3	3	D6WCN1_TRICA | Beta-galactosidase EC=3.2.1.23
16	Der f 4.0101	3	3	Der f 4.0101
16	cds.comp99324_c0_seq1|m.26033	2	2	A1KXH2_DERFA | Der f 1 allergen
16	cds.comp140939_c0_seq1|m.49098	2	2	Q178W0_AEDAE | AAEL005752-PA
17	cds.comp140939_c0_seq1|m.49098	10	10	Q178W0_AEDAE | AAEL005752-PA
17	cds.comp140939_c0_seq1|m.49097	7	7	Q5TS83_ANOGA | AGAP008584-PA
17	cds.comp108775_c0_seq1|m.34394	7	7	D6WCN1_TRICA | Beta-galactosidase EC=3.2.1.23
17	Der f 4.0101	4	4	Der f 4.0101
17	cds.comp110393_c0_seq1|m.35248	3	3	D6WCN1_TRICA | Beta-galactosidase EC=3.2.1.23
17	cds.comp13305_c0_seq1|m.3171	2	2	B3TFG6_9ACAR | Esterase TCE1
18	cds.comp140939_c0_seq1|m.49098	10	10	Q178W0_AEDAE | AAEL005752-PA
18	cds.comp140939_c0_seq1|m.49097	8	8	Q5TS83_ANOGA | AGAP008584-PA
18	cds.comp108775_c0_seq1|m.34394	6	6	D6WCN1_TRICA | Beta-galactosidase EC=3.2.1.23
18	cds.comp110393_c0_seq1|m.35248	3	3	D6WCN1_TRICA | Beta-galactosidase EC=3.2.1.23
18	cds.comp105191_c0_seq1|m.31572	3	3	L7M0B5_9ACAR | Putative beta-glucocerebrosidase
18	cds.comp13305_c0_seq1|m.3171	2	2	B3TFG6_9ACAR | Esterase TCE1
18	cds.comp97082_c0_seq1|m.23801	2	2	B7PNW7_IXOSC | Beta-glucocerebrosidase putative EC=3.2.1.45
18	cds.comp132682_c0_seq1|m.46457	2	2	E2ARG4_CAMFO | Beta-hexosaminidase EC=3.2.1.52

Spot number	Accession	#Peptides	#Unique	Description
19	cds.comp140939_c0_seq1|m.49098	9	9	Q178W0_AEDAE | AAEL005752-PA
19	cds.comp140939_c0_seq1|m.49097	7	7	Q5TS83_ANOGA | AGAP008584-PA
19	cds.comp13305_c0_seq1|m.3171	2	2	B3TFG6_9ACAR | Esterase TCE1
19	cds.comp132682_c0_seq1|m.46457	2	2	E2ARG4_CAMFO | Beta-hexosaminidase EC=3.2.1.52
19	cds.comp108775_c0_seq1|m.34394	2	2	D6WCN1_TRICA | Beta-galactosidase EC=3.2.1.23
20	cds.comp140939_c0_seq1|m.49098	8	8	Q178W0_AEDAE | AAEL005752-PA
20	cds.comp140939_c0_seq1|m.49097	6	6	Q5TS83_ANOGA | AGAP008584-PA
21	cds.comp140939_c0_seq1|m.49097	12	12	Q5TS83_ANOGA | AGAP008584-PA
21	cds.comp140939_c0_seq1|m.49098	11	11	Q178W0_AEDAE | AAEL005752-PA
21	cds.comp13305_c0_seq1|m.3171	2	2	B3TFG6_9ACAR | Esterase TCE1
22	cds.comp104804_c0_seq1|m.31206	28	27	B7PXR5_IXOSC | Chaperonin complex component TCP-1 eta subunit putative
22	cds.comp103307_c0_seq1|m.29701	13	13	B7P417_IXOSC | Peritrophic membrane chitin binding protein putative
22	cds.comp104803_c0_seq1|m.31204	8	8	B7PXR5_IXOSC | Chaperonin complex component TCP-1 eta subunit putative
22	cds.comp133675_c0_seq1|m.46787	5	5	F4WJH9_ACREC | Plasma glutamate carboxypeptidase
22	Der f 4.0101	5	5	Der f 4.0101
22	cds.comp21911_c0_seq1|m.5216	4	4	B5DL96_DROPS | GA22684
22	cds.comp112077_c0_seq1|m.36468	4	2	no hit
22	cds.comp112078_c0_seq1|m.36473	3	1	no hit
22	cds.comp99324_c0_seq1|m.26033	3	3	A1KXH2_DERFA | Der f 1 allergen

Spot number	Accession	#Peptides	#Unique	Description
22	cds.comp53900_c0_seq1|m.13006	3	3	Q9U6R7_DERFA | 98kDa HDM allergen SubName: Full=Group 15 allergen Der f 15
22	cds.comp44319_c0_seq1|m.10636	3	3	B7Q0R0_IXOSC | Phosphoglycerate mutase putative EC=5.4.2.1
22	cds.comp25511_c0_seq1|m.6088	2	2	L0GD06_BOMMO | Glucose-regulated protein 78
22	cds.comp13305_c0_seq1|m.3171	2	2	B3TFG6_9ACAR | Esterase TCE1
22	cds.comp21913_c0_seq1|m.5219	2	2	A3EXM6_MACHI | Putative 60 kDa heat shock protein Flags: Fragment
22	cds.comp120708_c0_seq1|m.41345	2	2	L7M898_9ACAR | Putative glycerol metabolic process
22	cds.comp17780_c0_seq1|m.4230	2	2	E1AC54_POLVA | Heat shock protein 60
22	cds.comp14554_c0_seq1|m.3464	2	2	A0SHR2_AMBVA | Protein disulfide isomerase EC=5.3.4.1
22	cds.comp9678_c0_seq1|m.2329	2	2	A0SHR2_AMBVA | Protein disulfide isomerase EC=5.3.4.1
22	cds.comp119114_c0_seq1|m.40635	2	2	L7M2B8_9ACAR | Putative n-acylaminoacyl-peptide hydrolase
23	cds.comp104802_c0_seq1|m.31198	29	29	B7PXR5_IXOSC | Chaperonin complex component TCP-1 eta subunit putative
23	cds.comp103307_c0_seq1|m.29701	12	12	B7P417_IXOSC | Peritrophic membrane chitin binding protein putative
23	cds.comp104804_c0_seq1|m.31207	11	11	B7PXR5_IXOSC | Chaperonin complex component TCP-1 eta subunit putative
23	cds.comp133675_c0_seq1|m.46787	6	6	F4WJH9_ACREC | Plasma glutamate carboxypeptidase
23	Der f 4.0101	6	6	Der f 4.0101
23	cds.comp21911_c0_seq1|m.5216	4	4	B5DL96_DROPS | GA22684
23	cds.comp112077_c0_seq1|m.36468	4	2	no hit
23	cds.comp53900_c0_seq1|m.13006	4	4	Q9U6R7_DERFA | 98kDa HDM allergen SubName: Full=Group 15 allergen Der f 15
23	Der f 15.0101	4	4	Der f 15.0101

Spot number	Accession	#Peptides	#Unique	Description
23	cds.comp112078_c0_seq1|m.36473	3	1	no hit
23	cds.comp99324_c0_seq1|m.26033	3	3	A1KXH2_DERFA | Der f 1 allergen
23	cds.comp13305_c0_seq1|m.3171	2	2	B3TFG6_9ACAR | Esterase TCE1
23	cds.comp44320_c0_seq1|m.10637	2	2	B7Q0R0_IXOSC | Phosphoglycerate mutase putative EC=5.4.2.1
23	cds.comp17779_c0_seq1|m.4228	2	2	A3EXM6_MACHI | Putative 60 kDa heat shock protein Flags: Fragment
23	cds.comp125335_c0_seq1|m.43231	2	2	L7M225_9ACAR | Putative lysosomal pro-x carboxypeptidase-like protein
23	cds.comp17780_c0_seq1|m.4230	2	2	E1AC54_POLVA | Heat shock protein 60
23	cds.comp44319_c0_seq1|m.10636	2	2	B7Q0R0_IXOSC | Phosphoglycerate mutase putative EC=5.4.2.1
23	cds.comp119114_c0_seq1|m.40635	2	2	L7M2B8_9ACAR | Putative n-acylaminoacyl-peptide hydrolase
24	cds.comp104802_c0_seq1|m.31198	27	26	B7PXR5_IXOSC | Chaperonin complex component TCP-1 eta subunit putative
24	cds.comp103307_c0_seq1|m.29701	14	14	B7P417_IXOSC | Peritrophic membrane chitin binding protein putative
24	cds.comp104804_c0_seq1|m.31207	9	9	B7PXR5_IXOSC | Chaperonin complex component TCP-1 eta subunit putative
24	cds.comp21911_c0_seq1|m.5216	8	8	B5DL96_DROPS | GA22684
24	Der f 4.0101	8	8	Der f 4.0101
24	cds.comp133675_c0_seq1|m.46787	6	6	F4WJH9_ACREC | Plasma glutamate carboxypeptidase
24	cds.comp53900_c0_seq1|m.13006	5	5	Q9U6R7_DERFA | 98kDa HDM allergen SubName: Full=Group 15 allergen Der f 15
24	cds.comp17779_c0_seq1|m.4228	4	4	A3EXM6_MACHI | Putative 60 kDa heat shock protein Flags: Fragment
24	Der f 1.0104	4	4	Der f 1.0104
24	cds.comp112074_c0_seq1|m.36460	3	3	no hit

Spot number	Accession	#Peptides	#Unique	Description
24	cds.comp17780_c0_seq1|m.4230	3	3	E1AC54_POLVA | Heat shock protein 60
24	cds.comp14554_c0_seq1|m.3464	3	3	A0SHR2_AMBVA | Protein disulfide isomerase EC=5.3.4.1
24	cds.comp44319_c0_seq1|m.10636	3	3	B7Q0R0_IXOSC | Phosphoglycerate mutase putative EC=5.4.2.1
24	cds.comp13305_c0_seq1|m.3171	2	2	B3TFG6_9ACAR | Esterase TCE1
24	cds.comp44320_c0_seq1|m.10637	2	2	B7Q0R0_IXOSC | Phosphoglycerate mutase putative EC=5.4.2.1
24	cds.comp151186_c0_seq1|m.51392	2	2	L7M384_9ACAR | Putative biotinidase and vanin
24	cds.comp125335_c0_seq1|m.43231	2	2	L7M225_9ACAR | Putative lysosomal pro-x carboxypeptidase-like protein
24	cds.comp112077_c0_seq1|m.36468	2	2	no hit
24	cds.comp105191_c0_seq1|m.31572	2	2	L7M0B5_9ACAR | Putative beta-glucocerebrosidase
24	Der f 3.0101	2	2	Der f 3.0101
25	Der f 4.0101	9	9	Der f 4.0101
25	cds.comp133675_c0_seq1|m.46787	8	8	F4WJH9_ACREC | Plasma glutamate carboxypeptidase
25	cds.comp103307_c0_seq1|m.29701	7	7	B7P417_IXOSC | Peritrophic membrane chitin binding protein putative
25	Der f 1.0104	5	5	Der f 1.0104
25	cds.comp105191_c0_seq1|m.31572	4	4	L7M0B5_9ACAR | Putative beta-glucocerebrosidase
25	cds.comp104802_c0_seq1|m.31198	4	4	B7PXR5_IXOSC | Chaperonin complex component TCP-1 eta subunit putative
25	cds.comp44319_c0_seq1|m.10636	4	4	B7Q0R0_IXOSC | Phosphoglycerate mutase putative EC=5.4.2.1
25	cds.comp112074_c0_seq1|m.36460	3	3	no hit
25	cds.comp112077_c0_seq1|m.36468	3	2	no hit

Spot number	Accession	#Peptides	#Unique	Description
25	cds.comp10023_c0_seq1|m.2409	3	3	E2BEF1_HARSA | Lysosomal alpha-glucosidase Flags: Fragment
25	Der f 18.0101	3	3	Der f 18.0101
25	cds.comp25511_c0_seq1|m.6088	2	2	L0GD06_BOMMO | Glucose-regulated protein 78
25	cds.comp13305_c0_seq1|m.3171	2	2	B3TFG6_9ACAR | Esterase TCE1
25	cds.comp97082_c0_seq1|m.23801	2	2	B7PNW7_IXOSC | Beta-glucocerebrosidase putative EC=3.2.1.45
25	cds.comp132682_c0_seq1|m.46457	2	2	E2ARG4_CAMFO | Beta-hexosaminidase EC=3.2.1.52
25	cds.comp112078_c0_seq1|m.36473	2	1	no hit
25	cds.comp151186_c0_seq1|m.51392	2	2	L7M384_9ACAR | Putative biotinidase and vanin
25	cds.comp120707_c0_seq1|m.41344	2	2	B7PNT8_IXOSC | Glycerophosphoryl diester phosphodiesterase putative
25	cds.comp131586_c0_seq1|m.46087	2	2	B7PB45_IXOSC | Transferrin receptor putative EC=3.4.11.10
25	cds.comp144500_c0_seq1|m.49975	2	2	E0W0P0_PEDHC | Plasma alpha-L-fucosidase putative EC=3.2.1.51
25	cds.comp151211_c0_seq1|m.51394	2	2	no hit
25	Der f 3.0101	2	2	Der f 3.0101
25	cds.comp106224_c0_seq1|m.32394	2	2	Q291S9_DROPS | GA20753 EC=3.2.1.-
25	cds.comp119114_c0_seq1|m.40635	2	2	L7M2B8_9ACAR | Putative n-acylaminoacyl-peptide hydrolase
26	Der f 4.0101	17	17	Der f 4.0101
26	cds.comp119114_c0_seq1|m.40635	15	15	L7M2B8_9ACAR | Putative n-acylaminoacyl-peptide hydrolase
26	cds.comp105191_c0_seq1|m.31572	9	9	L7M0B5_9ACAR | Putative beta-glucocerebrosidase
26	cds.comp144500_c0_seq1|m.49975	7	7	E0W0P0_PEDHC | Plasma alpha-L-fucosidase putative EC=3.2.1.51

Spot number	Accession	#Peptides	#Unique	Description
26	cds.comp56907_c0_seq1|m.13817	7	7	R4I416_BACDO | Phosphoglucose isomerase
26	cds.comp810_c0_seq1|m.281	5	5	L7MAN3_9ACAR | UDP-glucose 6-dehydrogenase EC=1.1.1.22
26	cds.comp77177_c0_seq1|m.18163	4	4	B4J0R3_DROGR | GH17138
26	cds.comp97082_c0_seq1|m.23801	4	4	B7PNW7_IXOSC | Beta-glucocerebrosidase putative EC=3.2.1.45
26	cds.comp32305_c0_seq1|m.7703	3	3	E2ABS8_CAMFO | Lysosomal alpha-mannosidase
26	cds.comp56906_c0_seq1|m.13816	3	3	C0L8T6_9CRUS | Glucose-6-phosphate isomerase EC=5.3.1.9
26	cds.comp87133_c0_seq1|m.20405	3	3	B7P0M7_IXOSC | Aldehyde dehydrogenase putative EC=1.5.1.12 Flags: Fragment
26	cds.comp134523_c0_seq1|m.47116	3	3	L7M765_9ACAR | Dihydrolipoyl dehydrogenase EC=1.8.1.4
26	cds.comp99826_c0_seq1|m.26591	3	3	L7MAH9_9ACAR | Succinyl-CoA:3-ketoacid-coenzyme A transferase EC=2.8.3.5
26	cds.comp13305_c0_seq1|m.3171	2	2	B3TFG6_9ACAR | Esterase TCE1
26	cds.comp151650_c0_seq1|m.51478	2	2	E7D172_LATHE | Putative purple acid phosphatase Flags: Fragment
26	cds.comp77176_c0_seq1|m.18162	2	2	L7M6V9_9ACAR | Putative aldehyde dehydrogenase
26	cds.comp99324_c0_seq1|m.26033	2	2	A1KXH2_DERFA | Der f 1 allergen
26	cds.comp120707_c0_seq1|m.41344	2	2	B7PNT8_IXOSC | Glycerophosphoryl diester phosphodiesterase putative
26	cds.comp114264_c0_seq1|m.37987	2	2	C1BQY6_9MAXI | Arylsulfatase A
27	Der f 4.0101	18	18	Der f 4.0101
27	cds.comp134523_c0_seq1|m.47116	9	9	L7M765_9ACAR | Dihydrolipoyl dehydrogenase EC=1.8.1.4
27	cds.comp119114_c0_seq1|m.40635	9	9	L7M2B8_9ACAR | Putative n-acylaminoacyl-peptide hydrolase
27	cds.comp144500_c0_seq1|m.49975	6	6	E0W0P0_PEDHC | Plasma alpha-L-fucosidase putative EC=3.2.1.51

Spot number	Accession	#Peptides	#Unique	Description
27	cds.comp105191_c0_seq1|m.31572	6	6	L7M0B5_9ACAR | Putative beta-glucocerebrosidase
27	cds.comp13305_c0_seq1|m.3171	3	3	B3TFG6_9ACAR | Esterase TCE1
27	cds.comp97082_c0_seq1|m.23801	3	3	B7PNW7_IXOSC | Beta-glucocerebrosidase putative EC=3.2.1.45
27	cds.comp56907_c0_seq1|m.13817	2	2	R4I416_BACDO | Phosphoglucose isomerase
27	cds.comp77175_c0_seq1|m.18161	2	2	B7P0M7_IXOSC | Aldehyde dehydrogenase putative EC=1.5.1.12 Flags: Fragment
28	Der f 4.0101	13	13	Der f 4.0101
28	cds.comp105191_c0_seq1|m.31572	5	5	L7M0B5_9ACAR | Putative beta-glucocerebrosidase
28	cds.comp97082_c0_seq1|m.23801	2	2	B7PNW7_IXOSC | Beta-glucocerebrosidase putative EC=3.2.1.45
29	cds.comp108274_c0_seq1|m.34025	11	11	Q0KKA6_HAELO | Leucine aminopeptidase
29	cds.comp103307_c0_seq1|m.29701	8	8	B7P417_IXOSC | Peritrophic membrane chitin binding protein putative
29	Der f 1.0104	7	7	Der f 1.0104
29	cds.comp125335_c0_seq1|m.43231	6	6	L7M225_9ACAR | Putative lysosomal pro-x carboxypeptidase-like protein
29	cds.comp144500_c0_seq1|m.49975	5	5	E0W0P0_PEDHC | Plasma alpha-L-fucosidase putative EC=3.2.1.51
29	cds.comp105191_c0_seq1|m.31572	5	5	L7M0B5_9ACAR | Putative beta-glucocerebrosidase
29	cds.comp119707_c0_seq1|m.40876	5	5	R4G4X4_RHOPR | Putative acid sphingomyelinase and phm5 phosphate metabolism protein
29	cds.comp98592_c0_seq1|m.25211	5	5	Q2HXW9_BLAGE | Enolase
29	Der f 4.0101	4	4	Der f 4.0101
29	cds.comp151186_c0_seq1|m.51392	3	3	L7M384_9ACAR | Putative biotinidase and vanin
29	cds.comp106225_c0_seq1|m.32397	3	2	B7PDZ5_IXOSC | Alpha-D-galactosidase putative EC=3.2.1.49

Spot number	Accession	#Peptides	#Unique	Description
29	cds.comp106224_c0_seq1|m.32394	3	2	Q291S9_DROPS | GA20753 EC=3.2.1.-
29	Der f 18.0101	3	3	Der f 18.0101
29	cds.comp101730_c0_seq1|m.28173	3	3	Q86R84_DERFA | 60 kDa allergen Der f 18p
29	cds.comp13305_c0_seq1|m.3171	2	2	B3TFG6_9ACAR | Esterase TCE1
29	cds.comp97082_c0_seq1|m.23801	2	2	B7PNW7_IXOSC | Beta-glucocerebrosidase putative EC=3.2.1.45
29	cds.comp132682_c0_seq1|m.46457	2	2	E2ARG4_CAMFO | Beta-hexosaminidase EC=3.2.1.52
29	cds.comp23945_c0_seq1|m.5737	2	2	no hit
29	cds.comp130821_c0_seq1|m.45783	2	2	no hit
29	cds.comp120707_c0_seq1|m.41344	2	2	B7PNT8_IXOSC | Glycerophosphoryl diester phosphodiesterase putative
29	Der f 3.0101	2	2	Der f 3.0101
29	cds.comp36162_c0_seq1|m.8681	2	2	E9G305_DAPPU | Putative leukotriene A4 hydrolase EC=3.3.2.6
29	cds.comp103559_c0_seq1|m.29931	2	2	L7M0C6_9ACAR | Putative beta-glucocerebrosidase
29	cds.comp99345_c0_seq1|m.26060	2	2	L7M2J0_9ACAR | Putative aminopeptidase of the m17 family
30	cds.comp108274_c0_seq1|m.34025	10	10	Q0KKA6_HAELO | Leucine aminopeptidase
30	cds.comp125335_c0_seq1|m.43231	7	7	L7M225_9ACAR | Putative lysosomal pro-x carboxypeptidase-like protein
30	cds.comp103307_c0_seq1|m.29701	7	7	B7P417_IXOSC | Peritrophic membrane chitin binding protein putative
30	cds.comp144500_c0_seq1|m.49975	6	6	E0W0P0_PEDHC | Plasma alpha-L-fucosidase putative EC=3.2.1.51
30	cds.comp98592_c0_seq1|m.25211	6	6	Q2HXW9_BLAGE | Enolase
30	cds.comp119707_c0_seq1|m.40876	5	5	R4G4X4_RHOPR | Putative acid sphingomyelinase and phm5 phosphate metabolism protein

Spot number	Accession	#Peptides	#Unique	Description
30	Der f 18.0101	4	4	Der f 18.0101
30	cds.comp106225_c0_seq1|m.32398	3	3	B7PDZ5_IXOSC | Alpha-D-galactosidase putative EC=3.2.1.49
30	cds.comp23945_c0_seq1|m.5737	3	3	no hit
30	cds.comp99320_c0_seq1|m.26025	3	3	A1KXH2_DERFA | Der f 1 allergen
30	cds.comp105191_c0_seq1|m.31572	3	3	L7M0B5_9ACAR | Putative beta-glucocerebrosidase
30	cds.comp106224_c0_seq1|m.32394	3	2	Q291S9_DROPS | GA20753 EC=3.2.1.-
30	Der f 4.0101	3	3	Der f 4.0101
30	cds.comp13305_c0_seq1|m.3171	2	2	B3TFG6_9ACAR | Esterase TCE1
30	cds.comp97082_c0_seq1|m.23801	2	2	B7PNW7_IXOSC | Beta-glucocerebrosidase putative EC=3.2.1.45
30	cds.comp132682_c0_seq1|m.46457	2	2	E2ARG4_CAMFO | Beta-hexosaminidase EC=3.2.1.52
30	cds.comp106225_c0_seq1|m.32397	2	1	B7PDZ5_IXOSC | Alpha-D-galactosidase putative EC=3.2.1.49
30	cds.comp36162_c0_seq1|m.8681	2	2	E9G305_DAPPU | Putative leukotriene A4 hydrolase EC=3.3.2.6
30	cds.comp129544_c0_seq1|m.45265	2	2	Q9Y196_EURMA | Alpha-amylase Flags: Precursor
30	cds.comp140939_c0_seq1|m.49097	2	2	Q5TS83_ANOGA | AGAP008584-PA
30	cds.comp137372_c0_seq1|m.48033	2	2	B7PI01_IXOSC | Serine carboxypeptidase putative EC=3.4.16.5 Flags: Fragment
30	cds.comp103559_c0_seq1|m.29931	2	2	L7M0C6_9ACAR | Putative beta-glucocerebrosidase
31	cds.comp98592_c0_seq1|m.25211	20	20	Q2HXW9_BLAGE | Enolase
31	cds.comp108273_c0_seq1|m.34021	15	15	Q0KKA6_HAELO | Leucine aminopeptidase
31	cds.comp144500_c0_seq1|m.49975	7	7	E0W0P0_PEDHC | Plasma alpha-L-fucosidase putative EC=3.2.1.51

Spot number	Accession	#Peptides	#Unique	Description
31	cds.comp119707_c0_seq1|m.40876	6	6	R4G4X4_RHOPR | Putative acid sphingomyelinase and phm5 phosphate metabolism protein
31	Der f 18.0101	5	5	Der f 18.0101
31	cds.comp106225_c0_seq1|m.32398	4	4	B7PDZ5_IXOSC | Alpha-D-galactosidase putative EC=3.2.1.49
31	cds.comp106224_c0_seq1|m.32394	4	3	Q291S9_DROPS | GA20753 EC=3.2.1.-
31	cds.comp99344_c0_seq1|m.26058	4	4	L7M2J0_9ACAR | Putative aminopeptidase of the m17 family
31	cds.comp99324_c0_seq1|m.26033	3	3	A1KXH2_DERFA | Der f 1 allergen
31	cds.comp36060_c0_seq1|m.8657	3	3	R4WCQ6_9HEMI | Proteinase
31	cds.comp132682_c0_seq1|m.46457	2	2	E2ARG4_CAMFO | Beta-hexosaminidase EC=3.2.1.52
31	cds.comp119370_c0_seq1|m.40727	2	2	L7MIP8_9ACAR | Putative beta-lactamase Flags: Fragment
31	cds.comp106225_c0_seq1|m.32397	2	1	B7PDZ5_IXOSC | Alpha-D-galactosidase putative EC=3.2.1.49
31	cds.comp137372_c0_seq1|m.48033	2	2	B7PI01_IXOSC | Serine carboxypeptidase putative EC=3.4.16.5 Flags: Fragment
31	cds.comp140939_c0_seq1|m.49097	2	2	Q5TS83_ANOGA | AGAP008584-PA
31	cds.comp103559_c0_seq1|m.29931	2	1	L7M0C6_9ACAR | Putative beta-glucocerebrosidase
32	cds.comp98592_c0_seq1|m.25211	15	15	Q2HXW9_BLAGE | Enolase
32	cds.comp144500_c0_seq1|m.49975	7	7	E0W0P0_PEDHC | Plasma alpha-L-fucosidase putative EC=3.2.1.51
32	cds.comp108274_c0_seq1|m.34025	6	6	Q0KKA6_HAELO | Leucine aminopeptidase
32	cds.comp99345_c0_seq1|m.26060	4	4	L7M2J0_9ACAR | Putative aminopeptidase of the m17 family
32	cds.comp99324_c0_seq1|m.26033	2	2	A1KXH2_DERFA | Der f 1 allergen
32	Der f 18.0101	2	2	Der f 18.0101

Spot number	Accession	#Peptides	#Unique	Description
32	cds.comp128094_c0_seq1|m.44567	2	2	L7M118_9ACAR | Putative purple acid phosphatase
33	cds.comp106225_c0_seq1|m.32397	8	8	B7PDZ5_IXOSC | Alpha-D-galactosidase putative EC=3.2.1.49
33	cds.comp32307_c0_seq1|m.7704	6	6	F4W8Y5_ACREC | Lysosomal alpha-mannosidase
33	Der f 1.0104	6	6	Der f 1.0104
33	cds.comp39309_c0_seq1|m.9403	5	5	E2ABS8_CAMFO | Lysosomal alpha-mannosidase
33	Der f 2.0108	4	4	Der f 2.0108
33	cds.comp113240_c0_seq1|m.37386	4	4	Q8ISH5_ARAVE | Chitinase
33	cds.comp98592_c0_seq1|m.25211	4	4	Q2HXW9_BLAGE | Enolase
33	cds.comp144500_c0_seq1|m.49975	3	3	E0W0P0_PEDHC | Plasma alpha-L-fucosidase putative EC=3.2.1.51
33	cds.comp119707_c0_seq1|m.40876	3	3	R4G4X4_RHOPR | Putative acid sphingomyelinase and phm5 phosphate metabolism protein
33	cds.comp137372_c0_seq1|m.48033	3	3	B7PI01_IXOSC | Serine carboxypeptidase putative EC=3.4.16.5 Flags: Fragment
33	cds.comp99344_c0_seq1|m.26058	3	3	L7M2J0_9ACAR | Putative aminopeptidase of the m17 family
33	Der f 18.0101	3	3	Der f 18.0101
33	cds.comp149823_c0_seq1|m.51085	2	2	M4LIT4_COTVE | Heat shock protein 70
34	cds.comp32307_c0_seq1|m.7704	8	8	F4W8Y5_ACREC | Lysosomal alpha-mannosidase
34	cds.comp32305_c0_seq1|m.7703	7	7	E2ABS8_CAMFO | Lysosomal alpha-mannosidase
34	cds.comp149823_c0_seq1|m.51085	6	4	M4LIT4_COTVE | Heat shock protein 70
34	cds.comp95676_c0_seq1|m.22346	6	6	Q16FX9_AEDAE | 4-hydroxyphenylpyruvate dioxygenase
34	Der f 28.0101	6	3	Der f 28.0101

Spot number	Accession	#Peptides	#Unique	Description
34	Der f 28.0201	5	2	Der f 28.0201
34	cds.comp10022_c0_seq1|m.2407	2	2	E2A599_CAMFO | Lysosomal alpha-glucosidase
35	cds.comp32307_c0_seq1|m.7704	8	8	F4W8Y5_ACREC | Lysosomal alpha-mannosidase
35	cds.comp32305_c0_seq1|m.7703	8	8	E2ABS8_CAMFO | Lysosomal alpha-mannosidase
35	cds.comp95676_c0_seq1|m.22346	7	7	Q16FX9_AEDAE | 4-hydroxyphenylpyruvate dioxygenase
35	cds.comp149823_c0_seq1|m.51085	3	2	M4LIT4_COTVE | Heat shock protein 70
35	cds.comp45467_c0_seq1|m.10986	2	2	no hit
35	Der f 28.0101	2	1	Der f 28.0101
36	cds.comp32305_c0_seq1|m.7703	6	6	E2ABS8_CAMFO | Lysosomal alpha-mannosidase
36	cds.comp149823_c0_seq1|m.51085	6	4	M4LIT4_COTVE | Heat shock protein 70
36	cds.comp95676_c0_seq1|m.22346	6	6	Q16FX9_AEDAE | 4-hydroxyphenylpyruvate dioxygenase
36	cds.comp32307_c0_seq1|m.7704	5	5	F4W8Y5_ACREC | Lysosomal alpha-mannosidase
36	Der f 28.0101	5	2	Der f 28.0101
36	cds.comp30659_c0_seq1|m.7326	3	3	B7P8Q5_IXOSC | Hsp70 putative EC=1.3.1.74 Flags: Fragment
36	Der f 28.0201	3	0	Der f 28.0201
37	cds.comp79187_c0_seq1|m.18650	5	5	E9IA80_SOLIN | Fructose-bisphosphate aldolase EC=4.1.2.13 Flags: Fragment
37	Der f 1.0104	5	5	Der f 1.0104
37	Der f 2.0108	4	4	Der f 2.0108
37	cds.comp113240_c0_seq1|m.37386	4	4	Q8ISH5_ARAVE | Chitinase

Spot number	Accession	#Peptides	#Unique	Description
37	Der f 18.0101	4	4	Der f 18.0101
37	cds.comp151211_c0_seq1|m.51394	3	3	no hit
37	cds.comp135033_c0_seq1|m.47262	2	2	B4J0B5_DROGR | GH15860
37	cds.comp86983_c0_seq1|m.20364	2	2	H9KR11_APIME | Fructose-bisphosphate aldolase EC=4.1.2.13
37	Der f 3.0101	2	2	Der f 3.0101
37	cds.comp119707_c0_seq1|m.40876	2	2	R4G4X4_RHOPR | Putative acid sphingomyelinase and phm5 phosphate metabolism protein
38	cds.comp45467_c0_seq1|m.10986	16	16	no hit
38	cds.comp310_c0_seq1|m.148	6	6	L7M5B4_9ACAR | Putative 3-hydroxyacyl-coa dehydrogenase
38	cds.comp79187_c0_seq1|m.18650	5	5	E9IA80_SOLIN | Fructose-bisphosphate aldolase EC=4.1.2.13 Flags: Fragment
38	cds.comp5505_c0_seq1|m.1316	3	3	Q6QWP0_9ARAC | Glyceraldehyde-3-phosphate dehydrogenase EC=1.2.1.12 Flags: Fragment
38	Der f 20.0201	3	3	Der f 20.0201
38	cds.comp39747_c0_seq1|m.9504	2	2	E7D199_LATHE | Glyceraldehyde-3-phosphate dehydrogenase EC=1.2.1.12 Flags: Fragment
38	cds.comp18558_c0_seq1|m.4417	2	2	Q8MWR4_DERPT | Serine protease LM-1 Flags: Fragment
38	cds.comp86983_c0_seq1|m.20364	2	2	H9KR11_APIME | Fructose-bisphosphate aldolase EC=4.1.2.13
38	cds.comp128015_c0_seq1|m.44536	2	2	B7P3P1_IXOSC | Cathepsin B endopeptidase putative EC=3.4.22.1
39	cds.comp128013_c0_seq1|m.44526	7	3	B7P3P0_IXOSC | Cathepsin B endopeptidase putative EC=3.4.22.1
39	Der f 3.0101	7	7	Der f 3.0101
39	cds.comp128018_c0_seq1|m.44542	6	0	B7P3P0_IXOSC | Cathepsin B endopeptidase putative EC=3.4.22.1
39	cds.comp310_c0_seq1|m.148	5	5	L7M5B4_9ACAR | Putative 3-hydroxyacyl-coa dehydrogenase

Spot number	Accession	#Peptides	#Unique	Description
39	cds.comp128016_c0_seq1|m.44537	5	1	B7P3P1_IXOSC | Cathepsin B endopeptidase putative EC=3.4.22.1
39	cds.comp90677_c0_seq1|m.21142	5	5	A7UI22_AMBAM | Lospin 7
39	cds.comp128015_c0_seq1|m.44536	5	1	B7P3P1_IXOSC | Cathepsin B endopeptidase putative EC=3.4.22.1
39	cds.comp5505_c0_seq1|m.1316	4	4	Q6QWP0_9ARAC | Glyceraldehyde-3-phosphate dehydrogenase EC=1.2.1.12 Flags: Fragment
39	Der f 1.0104	4	4	Der f 1.0104
39	cds.comp39747_c0_seq1|m.9504	3	3	E7D199_LATHE | Glyceraldehyde-3-phosphate dehydrogenase EC=1.2.1.12 Flags: Fragment
39	cds.comp151211_c0_seq1|m.51394	3	3	no hit
39	cds.comp52908_c0_seq1|m.12748	3	3	A1YW13_DERFA | Der f 1 allergen
39	cds.comp49378_c0_seq1|m.11900	3	3	B7QMV1_IXOSC | Elongation factor putative
39	cds.comp13305_c0_seq1|m.3171	2	2	B3TFG6_9ACAR | Esterase TCE1
39	cds.comp18558_c0_seq1|m.4417	2	2	Q8MWR4_DERPT | Serine protease LM-1 Flags: Fragment
39	cds.comp79187_c0_seq1|m.18650	2	2	E9IA80_SOLIN | Fructose-bisphosphate aldolase EC=4.1.2.13 Flags: Fragment
39	cds.comp10022_c0_seq1|m.2407	2	2	E2A599_CAMFO | Lysosomal alpha-glucosidase
40	cds.comp128013_c0_seq1|m.44526	6	2	B7P3P0_IXOSC | Cathepsin B endopeptidase putative EC=3.4.22.1
40	cds.comp128018_c0_seq1|m.44542	6	0	B7P3P0_IXOSC | Cathepsin B endopeptidase putative EC=3.4.22.1
40	cds.comp128015_c0_seq1|m.44536	5	1	B7P3P1_IXOSC | Cathepsin B endopeptidase putative EC=3.4.22.1
40	cds.comp115285_c0_seq1|m.38533	4	4	Q17DM4_AEDAE | AAEL004088-PC SubName: Full=AAEL004088-PD
40	cds.comp128016_c0_seq1|m.44537	4	0	B7P3P1_IXOSC | Cathepsin B endopeptidase putative EC=3.4.22.1
40	Der f 3.0101	4	4	Der f 3.0101

Spot number	Accession	#Peptides	#Unique	Description
40	cds.comp49378_c0_seq1|m.11900	4	4	B7QMV1_IXOSC | Elongation factor putative
40	cds.comp5505_c0_seq1|m.1316	3	3	Q6QWP0_9ARAC | Glyceraldehyde-3-phosphate dehydrogenase EC=1.2.1.12 Flags: Fragment
40	cds.comp310_c0_seq1|m.148	3	3	L7M5B4_9ACAR | Putative 3-hydroxyacyl-coa dehydrogenase
40	cds.comp39747_c0_seq1|m.9504	2	2	E7D199_LATHE | Glyceraldehyde-3-phosphate dehydrogenase EC=1.2.1.12 Flags: Fragment
40	cds.comp32305_c0_seq1|m.7703	2	2	E2ABS8_CAMFO | Lysosomal alpha-mannosidase
40	cds.comp99324_c0_seq1|m.26033	2	2	A1KXH2_DERFA | Der f 1 allergen
40	cds.comp52908_c0_seq1|m.12748	2	2	A1YW13_DERFA | Der f 1 allergen
41	cds.comp310_c0_seq1|m.148	13	13	L7M5B4_9ACAR | Putative 3-hydroxyacyl-coa dehydrogenase
41	cds.comp5505_c0_seq1|m.1316	7	7	Q6QWP0_9ARAC | Glyceraldehyde-3-phosphate dehydrogenase EC=1.2.1.12 Flags: Fragment
41	cds.comp10022_c0_seq1|m.2407	7	7	E2A599_CAMFO | Lysosomal alpha-glucosidase
41	Der f 2.0108	6	6	Der f 2.0108
41	cds.comp39747_c0_seq1|m.9504	5	5	E7D199_LATHE | Glyceraldehyde-3-phosphate dehydrogenase EC=1.2.1.12 Flags: Fragment
41	cds.comp128015_c0_seq1|m.44536	5	4	B7P3P1_IXOSC | Cathepsin B endopeptidase putative EC=3.4.22.1
41	cds.comp128013_c0_seq1|m.44526	4	3	B7P3P0_IXOSC | Cathepsin B endopeptidase putative EC=3.4.22.1
41	cds.comp128012_c0_seq1|m.44522	4	3	B7P3P0_IXOSC | Cathepsin B endopeptidase putative EC=3.4.22.1
41	cds.comp45467_c0_seq1|m.10986	4	4	no hit
41	Der f 20.0201	4	4	Der f 20.0201
41	cds.comp121851_c0_seq1|m.41954	3	3	B7P3M8_IXOSC | D-3-phosphoglycerate dehydrogenase putative EC=1.1.1.95 Flags: Fragment
41	cds.comp128018_c0_seq1|m.44542	2	0	B7P3P0_IXOSC | Cathepsin B endopeptidase putative EC=3.4.22.1

Spot number	Accession	#Peptides	#Unique	Description
41	cds.comp18558_c0_seq1|m.4417	2	2	Q8MWR4_DERPT | Serine protease LM-1 Flags: Fragment
41	cds.comp99324_c0_seq1|m.26033	2	2	A1KXH2_DERFA | Der f 1 allergen
41	cds.comp79187_c0_seq1|m.18650	2	2	E9IA80_SOLIN | Fructose-bisphosphate aldolase EC=4.1.2.13 Flags: Fragment
41	Der f 3.0101	2	2	Der f 3.0101
42	cds.comp310_c0_seq1|m.148	13	13	L7M5B4_9ACAR | Putative 3-hydroxyacyl-coa dehydrogenase
42	cds.comp5505_c0_seq1|m.1316	8	8	Q6QWP0_9ARAC | Glyceraldehyde-3-phosphate dehydrogenase EC=1.2.1.12 Flags: Fragment
42	cds.comp39747_c0_seq1|m.9504	5	5	E7D199_LATHE | Glyceraldehyde-3-phosphate dehydrogenase EC=1.2.1.12 Flags: Fragment
42	cds.comp128015_c0_seq1|m.44536	5	5	B7P3P1_IXOSC | Cathepsin B endopeptidase putative EC=3.4.22.1
42	cds.comp45467_c0_seq1|m.10986	4	4	no hit
42	cds.comp128013_c0_seq1|m.44526	3	3	B7P3P0_IXOSC | Cathepsin B endopeptidase putative EC=3.4.22.1
42	cds.comp121851_c0_seq1|m.41954	3	3	B7P3M8_IXOSC | D-3-phosphoglycerate dehydrogenase putative EC=1.1.1.95 Flags: Fragment
42	cds.comp18558_c0_seq1|m.4417	2	2	Q8MWR4_DERPT | Serine protease LM-1 Flags: Fragment
42	cds.comp10022_c0_seq1|m.2407	2	2	E2A599_CAMFO | Lysosomal alpha-glucosidase
43	cds.comp310_c0_seq1|m.148	14	14	L7M5B4_9ACAR | Putative 3-hydroxyacyl-coa dehydrogenase
43	cds.comp5505_c0_seq1|m.1316	9	9	Q6QWP0_9ARAC | Glyceraldehyde-3-phosphate dehydrogenase EC=1.2.1.12 Flags: Fragment
43	cds.comp45467_c0_seq1|m.10986	8	8	no hit
43	cds.comp5506_c0_seq1|m.1317	5	5	E7D199_LATHE | Glyceraldehyde-3-phosphate dehydrogenase EC=1.2.1.12 Flags: Fragment
43	cds.comp128015_c0_seq1|m.44536	5	5	B7P3P1_IXOSC | Cathepsin B endopeptidase putative EC=3.4.22.1
43	cds.comp83879_c0_seq1|m.19673	4	4	R4G3J7_RHOPR | Putative gamma interferon inducible lysosomal thiol reductase gilt

Spot number	Accession	#Peptides	#Unique	Description
43	Der f 20.0201	4	4	Der f 20.0201
43	cds.comp128013_c0_seq1|m.44526	3	3	B7P3P0_IXOSC | Cathepsin B endopeptidase putative EC=3.4.22.1
43	cds.comp10022_c0_seq1|m.2407	3	3	E2A599_CAMFO | Lysosomal alpha-glucosidase
43	cds.comp121851_c0_seq1|m.41954	3	3	B7P3M8_IXOSC | D-3-phosphoglycerate dehydrogenase putative EC=1.1.1.95 Flags: Fragment
43	cds.comp18558_c0_seq1|m.4417	2	2	Q8MWR4_DERPT | Serine protease LM-1 Flags: Fragment
43	cds.comp99324_c0_seq1|m.26033	2	2	A1KXH2_DERFA | Der f 1 allergen
43	cds.comp79187_c0_seq1|m.18650	2	2	E9IA80_SOLIN | Fructose-bisphosphate aldolase EC=4.1.2.13 Flags: Fragment
43	cds.comp96191_c0_seq1|m.22959	2	2	no hit
44	cds.comp310_c0_seq1|m.148	15	15	L7M5B4_9ACAR | Putative 3-hydroxyacyl-coa dehydrogenase
44	cds.comp5505_c0_seq1|m.1316	7	7	Q6QWP0_9ARAC | Glyceraldehyde-3-phosphate dehydrogenase EC=1.2.1.12 Flags: Fragment
44	cds.comp39747_c0_seq1|m.9504	5	5	E7D199_LATHE | Glyceraldehyde-3-phosphate dehydrogenase EC=1.2.1.12 Flags: Fragment
44	cds.comp128015_c0_seq1|m.44536	4	4	B7P3P1_IXOSC | Cathepsin B endopeptidase putative EC=3.4.22.1
44	cds.comp95676_c0_seq1|m.22346	4	4	Q16FX9_AEDAE | 4-hydroxyphenylpyruvate dioxygenase
44	cds.comp128013_c0_seq1|m.44526	3	3	B7P3P0_IXOSC | Cathepsin B endopeptidase putative EC=3.4.22.1
44	cds.comp18558_c0_seq1|m.4417	2	2	Q8MWR4_DERPT | Serine protease LM-1 Flags: Fragment
44	cds.comp115285_c0_seq1|m.38533	2	2	Q17DM4_AEDAE | AAEL004088-PC SubName: Full=AAEL004088-PD
45	cds.comp108798_c0_seq1|m.34424	12	12	G6CUI4_DANPL | Molting fluid carboxypeptidase A
45	Der f 3.0101	10	10	Der f 3.0101
45	cds.comp128015_c0_seq1|m.44536	7	5	B7P3P1_IXOSC | Cathepsin B endopeptidase putative EC=3.4.22.1

Spot number	Accession	#Peptides	#Unique	Description
45	Der f 1.0104	6	6	Der f 1.0104
45	cds.comp15303_c0_seq1|m.3655	5	4	G3ACU4_GLOMR | Actin 5C/42A Flags: Fragment
45	cds.comp128013_c0_seq1|m.44526	5	3	B7P3P0_IXOSC | Cathepsin B endopeptidase putative EC=3.4.22.1
45	Der f 6.0101	5	4	Der f 6.0101
45	cds.comp20812_c0_seq1|m.4957	4	3	B2MVM3_SARSC | Actin
45	cds.comp128018_c0_seq1|m.44542	4	0	B7P3P0_IXOSC | Cathepsin B endopeptidase putative EC=3.4.22.1
45	cds.comp100364_c0_seq1|m.27163	4	4	Q8MRY3_DROME | SD13780p
45	cds.comp99833_c0_seq1|m.26596	4	4	A7UNU0_9ACAR | Ale o 1 allergen
45	cds.comp112368_c0_seq1|m.36667	2	1	Q155V8_DERFA | Der f 6 Flags: Fragment
45	cds.comp151211_c0_seq1|m.51394	2	2	no hit
45	cds.comp52908_c0_seq1|m.12748	2	2	A1YW13_DERFA | Der f 1 allergen
45	Der f 27.0101	2	2	Der f 27.0101
46	cds.comp108798_c0_seq1|m.34424	11	11	G6CUI4_DANPL | Molting fluid carboxypeptidase A
46	Der f 3.0101	9	9	Der f 3.0101
46	cds.comp128015_c0_seq1|m.44536	6	1	B7P3P1_IXOSC | Cathepsin B endopeptidase putative EC=3.4.22.1
46	cds.comp128013_c0_seq1|m.44526	5	3	B7P3P0_IXOSC | Cathepsin B endopeptidase putative EC=3.4.22.1
46	cds.comp128016_c0_seq1|m.44537	5	0	B7P3P1_IXOSC | Cathepsin B endopeptidase putative EC=3.4.22.1
46	cds.comp102387_c0_seq1|m.28696	5	5	A1KXH7_DERFA | Der f Alt a 10 allergen
46	cds.comp128018_c0_seq1|m.44542	4	0	B7P3P0_IXOSC | Cathepsin B endopeptidase putative EC=3.4.22.1

Spot number	Accession	#Peptides	#Unique	Description
46	cds.comp100364_c0_seq1|m.27163	4	4	Q8MRY3_DROME | SD13780p
46	Der f 1.0104	4	4	Der f 1.0104
46	Der f 6.0101	4	4	Der f 6.0101
46	cds.comp15303_c0_seq1|m.3655	3	3	G3ACU4_GLOMR | Actin 5C/42A Flags: Fragment
46	cds.comp151211_c0_seq1|m.51394	2	2	no hit
46	cds.comp52908_c0_seq1|m.12748	2	2	A1YW13_DERFA | Der f 1 allergen
47	cds.comp108798_c0_seq1|m.34424	10	10	G6CUI4_DANPL | Molting fluid carboxypeptidase A
47	cds.comp102387_c0_seq1|m.28696	10	10	A1KXH7_DERFA | Der f Alt a 10 allergen
47	Der f 3.0101	9	9	Der f 3.0101
47	cds.comp128015_c0_seq1|m.44536	7	5	B7P3P1_IXOSC | Cathepsin B endopeptidase putative EC=3.4.22.1
47	cds.comp128013_c0_seq1|m.44526	6	3	B7P3P0_IXOSC | Cathepsin B endopeptidase putative EC=3.4.22.1
47	cds.comp128018_c0_seq1|m.44542	5	0	B7P3P0_IXOSC | Cathepsin B endopeptidase putative EC=3.4.22.1
47	Der f 1.0104	4	4	Der f 1.0104
47	Der f 6.0101	4	4	Der f 6.0101
47	cds.comp90677_c0_seq1|m.21142	4	4	A7UI22_AMBAM | Lospin 7
47	cds.comp99833_c0_seq1|m.26596	3	3	A7UNU0_9ACAR | Ale o 1 allergen
47	cds.comp151211_c0_seq1|m.51394	3	3	no hit
47	cds.comp100364_c0_seq1|m.27163	3	3	Q8MRY3_DROME | SD13780p
47	cds.comp52908_c0_seq1|m.12748	3	3	A1YW13_DERFA | Der f 1 allergen

Spot number	Accession	#Peptides	#Unique	Description
47	cds.comp103358_c0_seq1|m.29778	2	2	C4WUM8_ACYPI | ACYPI003308 protein SubName: Full=Uncharacterized protein
47	Der f 2.0106	2	2	Der f 2.0106
47	cds.comp101581_c0_seq1|m.28089	2	2	no hit
47	cds.comp25200_c0_seq1|m.6015	2	2	E9GFR6_DAPPU | Alpha-carbonic anhydrase EC=4.2.1.1
47	cds.comp115285_c0_seq1|m.38533	2	2	Q17DM4_AEDAE | AAEL004088-PC SubName: Full=AAEL004088-PD
47	cds.comp49378_c0_seq1|m.11900	2	2	B7QMV1_IXOSC | Elongation factor putative
47	cds.comp113240_c0_seq1|m.37386	2	2	Q8ISH5_ARAVE | Chitinase
48	Der f 3.0101	13	13	Der f 3.0101
48	cds.comp128013_c0_seq1|m.44526	7	3	B7P3P0_IXOSC | Cathepsin B endopeptidase putative EC=3.4.22.1
48	Der f 1.0104	7	7	Der f 1.0104
48	cds.comp108798_c0_seq1|m.34424	7	7	G6CUI4_DANPL | Molting fluid carboxypeptidase A
48	cds.comp128018_c0_seq1|m.44542	6	0	B7P3P0_IXOSC | Cathepsin B endopeptidase putative EC=3.4.22.1
48	Der f 6.0101	6	4	Der f 6.0101
48	cds.comp128015_c0_seq1|m.44536	6	4	B7P3P1_IXOSC | Cathepsin B endopeptidase putative EC=3.4.22.1
48	cds.comp113240_c0_seq1|m.37386	6	6	Q8ISH5_ARAVE | Chitinase
48	cds.comp52908_c0_seq1|m.12748	4	4	A1YW13_DERFA | Der f 1 allergen
48	cds.comp115285_c0_seq1|m.38533	3	3	Q17DM4_AEDAE | AAEL004088-PC SubName: Full=AAEL004088-PD
48	cds.comp112367_c0_seq1|m.36666	3	1	Q155V8_DERFA | Der f 6 Flags: Fragment
48	cds.comp13305_c0_seq1|m.3171	2	2	B3TFG6_9ACAR | Esterase TCE1

Spot number	Accession	#Peptides	#Unique	Description
48	cds.comp25200_c0_seq1|m.6015	2	2	E9GFR6_DAPPU | Alpha-carbonic anhydrase EC=4.2.1.1
48	cds.comp45957_c0_seq1|m.11079	2	2	B7QMV1_IXOSC | Elongation factor putative
49	cds.comp101423_c0_seq1|m.28025	20	5	A1KXH3_DERFA | Der f 3 allergen
49	Der f 3.0101	16	1	Der f 3.0101
49	cds.comp128016_c0_seq1|m.44537	7	1	B7P3P1_IXOSC | Cathepsin B endopeptidase putative EC=3.4.22.1
49	cds.comp128015_c0_seq1|m.44536	7	1	B7P3P1_IXOSC | Cathepsin B endopeptidase putative EC=3.4.22.1
49	Der f 1.0104	5	5	Der f 1.0104
49	cds.comp128013_c0_seq1|m.44526	4	2	B7P3P0_IXOSC | Cathepsin B endopeptidase putative EC=3.4.22.1
49	cds.comp128018_c0_seq1|m.44542	4	0	B7P3P0_IXOSC | Cathepsin B endopeptidase putative EC=3.4.22.1
49	cds.comp52908_c0_seq1|m.12748	2	2	A1YW13_DERFA | Der f 1 allergen
50	Der f 6.0101	9	5	Der f 6.0101
50	Der f 3.0101	9	9	Der f 3.0101
50	cds.comp113240_c0_seq1|m.37386	9	9	Q8ISH5_ARAVE | Chitinase
50	Der f 1.0104	8	8	Der f 1.0104
50	cds.comp128013_c0_seq1|m.44526	7	3	B7P3P0_IXOSC | Cathepsin B endopeptidase putative EC=3.4.22.1
50	cds.comp128015_c0_seq1|m.44536	7	5	B7P3P1_IXOSC | Cathepsin B endopeptidase putative EC=3.4.22.1
50	cds.comp128018_c0_seq1|m.44542	6	0	B7P3P0_IXOSC | Cathepsin B endopeptidase putative EC=3.4.22.1
50	cds.comp112368_c0_seq1|m.36667	5	1	Q155V8_DERFA | Der f 6 Flags: Fragment
50	cds.comp52908_c0_seq1|m.12748	5	5	A1YW13_DERFA | Der f 1 allergen

Spot number	Accession	#Peptides	#Unique	Description
50	Der f 18.0101	3	3	Der f 18.0101
50	cds.comp13305_c0_seq1|m.3171	2	2	B3TFG6_9ACAR | Esterase TCE1
50	cds.comp25200_c0_seq1|m.6015	2	2	E9GFR6_DAPPU | Alpha-carbonic anhydrase EC=4.2.1.1
50	cds.comp18558_c0_seq1|m.4417	2	2	Q8MWR4_DERPT | Serine protease LM-1 Flags: Fragment
50	cds.comp116462_c0_seq1|m.39120	2	2	B5DYT7_DROPS | GA26775
51	Der f 3.0101	8	8	Der f 3.0101
51	cds.comp128013_c0_seq1|m.44526	7	3	B7P3P0_IXOSC | Cathepsin B endopeptidase putative EC=3.4.22.1
51	cds.comp128018_c0_seq1|m.44542	6	0	B7P3P0_IXOSC | Cathepsin B endopeptidase putative EC=3.4.22.1
51	cds.comp128016_c0_seq1|m.44537	6	1	B7P3P1_IXOSC | Cathepsin B endopeptidase putative EC=3.4.22.1
51	cds.comp128015_c0_seq1|m.44536	6	1	B7P3P1_IXOSC | Cathepsin B endopeptidase putative EC=3.4.22.1
51	cds.comp113240_c0_seq1|m.37386	6	6	Q8ISH5_ARAVE | Chitinase
51	Der f 1.0104	5	5	Der f 1.0104
51	cds.comp52908_c0_seq1|m.12748	4	4	A1YW13_DERFA | Der f 1 allergen
51	Der f 6.0101	3	3	Der f 6.0101
51	cds.comp13305_c0_seq1|m.3171	2	2	B3TFG6_9ACAR | Esterase TCE1
51	cds.comp25200_c0_seq1|m.6015	2	2	E9GFR6_DAPPU | Alpha-carbonic anhydrase EC=4.2.1.1
51	cds.comp151211_c0_seq1|m.51394	2	2	no hit
52	Der f 3.0101	9	9	Der f 3.0101
52	cds.comp128013_c0_seq1|m.44526	7	3	B7P3P0_IXOSC | Cathepsin B endopeptidase putative EC=3.4.22.1

Spot number	Accession	#Peptides	#Unique	Description
52	cds.comp128015_c0_seq1|m.44536	7	1	B7P3P1_IXOSC | Cathepsin B endopeptidase putative EC=3.4.22.1
52	cds.comp128018_c0_seq1|m.44542	6	0	B7P3P0_IXOSC | Cathepsin B endopeptidase putative EC=3.4.22.1
52	cds.comp128016_c0_seq1|m.44537	6	0	B7P3P1_IXOSC | Cathepsin B endopeptidase putative EC=3.4.22.1
52	Der f 1.0104	4	4	Der f 1.0104
52	Der f 6.0101	2	2	Der f 6.0101
53	cds.comp105708_c0_seq1|m.31983	7	7	F4WQI8_ACREC | Actin-interacting protein 1
53	Der f 3.0101	7	7	Der f 3.0101
53	cds.comp128015_c0_seq1|m.44536	7	5	B7P3P1_IXOSC | Cathepsin B endopeptidase putative EC=3.4.22.1
53	cds.comp52908_c0_seq1|m.12748	6	6	A1YW13_DERFA | Der f 1 allergen
53	cds.comp128013_c0_seq1|m.44526	5	3	B7P3P0_IXOSC | Cathepsin B endopeptidase putative EC=3.4.22.1
53	cds.comp128018_c0_seq1|m.44542	4	0	B7P3P0_IXOSC | Cathepsin B endopeptidase putative EC=3.4.22.1
53	Der f 1.0104	4	4	Der f 1.0104
53	cds.comp90677_c0_seq1|m.21142	3	3	A7UI22_AMBAM | Lospin 7
53	Der f 18.0101	3	3	Der f 18.0101
53	cds.comp13305_c0_seq1|m.3171	2	2	B3TFG6_9ACAR | Esterase TCE1
53	cds.comp18558_c0_seq1|m.4417	2	2	Q8MWR4_DERPT | Serine protease LM-1 Flags: Fragment
53	cds.comp115285_c0_seq1|m.38533	2	2	Q17DM4_AEDAE | AAEL004088-PC SubName: Full=AAEL004088-PD
53	cds.comp83879_c0_seq1|m.19673	2	2	R4G3J7_RHOPR | Putative gamma interferon inducible lysosomal thiol reductase gilt
53	cds.comp17339_c0_seq1|m.4137	2	2	B7PQV0_IXOSC | Alpha-aspartyl dipeptidase putative EC=3.4.13.21

Spot number	Accession	#Peptides	#Unique	Description
53	cds.comp115085_c0_seq1|m.38400	2	2	B7P3P0_IXOSC | Cathepsin B endopeptidase putative EC=3.4.22.1
53	Der f 4.0101	2	2	Der f 4.0101
54	cds.comp128013_c0_seq1|m.44526	7	3	B7P3P0_IXOSC | Cathepsin B endopeptidase putative EC=3.4.22.1
54	cds.comp128016_c0_seq1|m.44537	7	1	B7P3P1_IXOSC | Cathepsin B endopeptidase putative EC=3.4.22.1
54	cds.comp128015_c0_seq1|m.44536	7	1	B7P3P1_IXOSC | Cathepsin B endopeptidase putative EC=3.4.22.1
54	cds.comp128018_c0_seq1|m.44542	6	0	B7P3P0_IXOSC | Cathepsin B endopeptidase putative EC=3.4.22.1
54	Der f 3.0101	5	5	Der f 3.0101
54	Der f 1.0104	3	3	Der f 1.0104
55	cds.comp105708_c0_seq1|m.31983	12	12	F4WQI8_ACREC | Actin-interacting protein 1
55	Der f 3.0101	9	9	Der f 3.0101
55	cds.comp52908_c0_seq1|m.12748	8	8	A1YW13_DERFA | Der f 1 allergen
55	cds.comp90677_c0_seq1|m.21142	8	8	A7UI22_AMBAM | Lospin 7
55	cds.comp128015_c0_seq1|m.44536	7	5	B7P3P1_IXOSC | Cathepsin B endopeptidase putative EC=3.4.22.1
55	cds.comp128013_c0_seq1|m.44526	5	3	B7P3P0_IXOSC | Cathepsin B endopeptidase putative EC=3.4.22.1
55	cds.comp128018_c0_seq1|m.44542	4	0	B7P3P0_IXOSC | Cathepsin B endopeptidase putative EC=3.4.22.1
55	Der f 2.0106	3	3	Der f 2.0106
55	cds.comp99324_c0_seq1|m.26033	2	2	A1KXH2_DERFA | Der f 1 allergen
56	cds.comp14401_c0_seq1|m.3427	6	6	Q7PX08_ANOGA | AGAP001151-PA
56	Der f 27.0101	6	6	Der f 27.0101

Spot number	Accession	#Peptides	#Unique	Description
56	cds.comp128016_c0_seq1|m.44537	4	0	B7P3P1_IXOSC | Cathepsin B endopeptidase putative EC=3.4.22.1
56	Der f 1.0104	4	4	Der f 1.0104
56	cds.comp128015_c0_seq1|m.44536	4	0	B7P3P1_IXOSC | Cathepsin B endopeptidase putative EC=3.4.22.1
56	Der f 3.0101	4	4	Der f 3.0101
56	cds.comp128018_c0_seq1|m.44542	2	1	B7P3P0_IXOSC | Cathepsin B endopeptidase putative EC=3.4.22.1
56	cds.comp100364_c0_seq1|m.27163	2	2	Q8MRY3_DROME | SD13780p
56	cds.comp99833_c0_seq1|m.26596	2	2	A7UNU0_9ACAR | Ale o 1 allergen
56	cds.comp14554_c0_seq1|m.3464	2	2	A0SHR2_AMBVA | Protein disulfide isomerase EC=5.3.4.1
57	cds.comp128015_c0_seq1|m.44536	6	5	B7P3P1_IXOSC | Cathepsin B endopeptidase putative EC=3.4.22.1
57	Der f 1.0104	5	5	Der f 1.0104
57	Der f 3.0101	5	5	Der f 3.0101
57	Der f 27.0101	4	4	Der f 27.0101
57	cds.comp99833_c0_seq1|m.26596	3	3	A7UNU0_9ACAR | Ale o 1 allergen
57	cds.comp100364_c0_seq1|m.27163	3	3	Q8MRY3_DROME | SD13780p
57	cds.comp14554_c0_seq1|m.3464	3	3	A0SHR2_AMBVA | Protein disulfide isomerase EC=5.3.4.1
57	cds.comp128018_c0_seq1|m.44542	2	1	B7P3P0_IXOSC | Cathepsin B endopeptidase putative EC=3.4.22.1
57	cds.comp78532_c0_seq1|m.18484	2	2	A1KXH8_DERFA | Der f Gal d 2 allergen
57	cds.comp108798_c0_seq1|m.34424	2	2	G6CUI4_DANPL | Molting fluid carboxypeptidase A
57	Der f 6.0101	2	2	Der f 6.0101

Spot number	Accession	#Peptides	#Unique	Description
57	cds.comp98590_c0_seq1|m.25209	2	2	Q2HXW9_BLAGE | Enolase
57	cds.comp103307_c0_seq1|m.29701	2	2	B7P417_IXOSC | Peritrophic membrane chitin binding protein putative
58	cds.comp128015_c0_seq1|m.44536	4	4	B7P3P1_IXOSC | Cathepsin B endopeptidase putative EC=3.4.22.1
58	cds.comp18558_c0_seq1|m.4417	3	3	Q8MWR4_DERPT | Serine protease LM-1 Flags: Fragment
58	cds.comp39309_c0_seq1|m.9403	2	2	E2ABS8_CAMFO | Lysosomal alpha-mannosidase
58	Der f 1.0104	2	2	Der f 1.0104
59	cds.comp128015_c0_seq1|m.44536	5	5	B7P3P1_IXOSC | Cathepsin B endopeptidase putative EC=3.4.22.1
59	cds.comp18558_c0_seq1|m.4417	4	4	Q8MWR4_DERPT | Serine protease LM-1 Flags: Fragment
59	cds.comp106751_c0_seq1|m.32734	4	4	no hit
59	Der f 1.0104	3	3	Der f 1.0104
59	cds.comp310_c0_seq1|m.148	2	2	L7M5B4_9ACAR | Putative 3-hydroxyacyl-coa dehydrogenase
60	cds.comp128015_c0_seq1|m.44536	6	6	B7P3P1_IXOSC | Cathepsin B endopeptidase putative EC=3.4.22.1
60	cds.comp106751_c0_seq1|m.32734	5	5	no hit
60	cds.comp18558_c0_seq1|m.4417	4	4	Q8MWR4_DERPT | Serine protease LM-1 Flags: Fragment
60	cds.comp128013_c0_seq1|m.44526	2	2	B7P3P0_IXOSC | Cathepsin B endopeptidase putative EC=3.4.22.1
60	cds.comp99324_c0_seq1|m.26033	2	2	A1KXH2_DERFA | Der f 1 allergen
61	Der f 1.0104	10	10	Der f 1.0104
61	Der f 27.0101	4	4	Der f 27.0101
61	cds.comp111232_c0_seq1|m.35846	4	4	no hit

Spot number	Accession	#Peptides	#Unique	Description
61	cds.comp18558_c0_seq1|m.4417	3	3	Q8MWR4_DERPT | Serine protease LM-1 Flags: Fragment
61	cds.comp104653_c0_seq1|m.31049	3	3	no hit
61	Der f 3.0101	3	3	Der f 3.0101
61	cds.comp13305_c0_seq1|m.3171	2	2	B3TFG6_9ACAR | Esterase TCE1
61	cds.comp128018_c0_seq1|m.44542	2	2	B7P3P0_IXOSC | Cathepsin B endopeptidase putative EC=3.4.22.1
61	cds.comp80154_c0_seq1|m.18914	2	2	Der f 35
61	cds.comp78532_c0_seq1|m.18484	2	2	A1KXH8_DERFA | Der f Gal d 2 allergen
61	Der f 6.0101	2	1	Der f 6.0101
61	cds.comp70311_c0_seq1|m.16736	2	2	E9GAM5_DAPPU | 6-phosphogluconate dehydrogenase decarboxylating EC=1.1.1.44
61	cds.comp100364_c0_seq1|m.27163	2	2	Q8MRY3_DROME | SD13780p
61	cds.comp112368_c0_seq1|m.36667	2	1	Q155V8_DERFA | Der f 6 Flags: Fragment
62	Der f 1.0104	8	8	Der f 1.0104
62	cds.comp104654_c0_seq1|m.31050	5	5	no hit
62	Der f 6.0101	4	2	Der f 6.0101
62	cds.comp70311_c0_seq1|m.16736	3	3	E9GAM5_DAPPU | 6-phosphogluconate dehydrogenase decarboxylating EC=1.1.1.44
62	cds.comp112368_c0_seq1|m.36667	3	1	Q155V8_DERFA | Der f 6 Flags: Fragment
62	cds.comp18558_c0_seq1|m.4417	2	2	Q8MWR4_DERPT | Serine protease LM-1 Flags: Fragment
62	cds.comp111232_c0_seq1|m.35846	2	2	no hit
63	Der f 1.0104	8	8	Der f 1.0104

Spot number	Accession	#Peptides	#Unique	Description
63	Der f 6.0101	4	2	Der f 6.0101
63	cds.comp112368_c0_seq1|m.36667	3	1	Q155V8_DERFA | Der f 6 Flags: Fragment
63	cds.comp18558_c0_seq1|m.4417	2	2	Q8MWR4_DERPT | Serine protease LM-1 Flags: Fragment
63	Der f 3.0101	2	2	Der f 3.0101
64	Der f 1.0104	13	13	Der f 1.0104
64	Der f 6.0101	5	2	Der f 6.0101
64	cds.comp112368_c0_seq1|m.36667	5	2	Q155V8_DERFA | Der f 6 Flags: Fragment
64	cds.comp18558_c0_seq1|m.4417	3	3	Q8MWR4_DERPT | Serine protease LM-1 Flags: Fragment
64	cds.comp14554_c0_seq1|m.3464	2	2	A0SHR2_AMBVA | Protein disulfide isomerase EC=5.3.4.1
64	cds.comp52908_c0_seq1|m.12748	2	2	A1YW13_DERFA | Der f 1 allergen
65	Der f 1.0104	12	12	Der f 1.0104
65	Der f 6.0101	10	4	Der f 6.0101
65	cds.comp112368_c0_seq1|m.36667	8	2	Q155V8_DERFA | Der f 6 Flags: Fragment
65	cds.comp18558_c0_seq1|m.4417	3	3	Q8MWR4_DERPT | Serine protease LM-1 Flags: Fragment
65	cds.comp13305_c0_seq1|m.3171	2	2	B3TFG6_9ACAR | Esterase TCE1
65	cds.comp52908_c0_seq1|m.12748	2	2	A1YW13_DERFA | Der f 1 allergen
65	cds.comp128018_c0_seq1|m.44542	1	1	B7P3P0_IXOSC | Cathepsin B endopeptidase putative EC=3.4.22.1
65	cds.comp77465_c0_seq1|m.18227	1	1	L7M6N0_9ACAR | Putative eukaryotic translation initiation factor
66	Der f 1.0104	13	13	Der f 1.0104

Spot number	Accession	#Peptides	#Unique	Description
66	cds.comp13305_c0_seq1|m.3171	3	3	B3TFG6_9ACAR | Esterase TCE1
66	cds.comp18558_c0_seq1|m.4417	3	3	Q8MWR4_DERPT | Serine protease LM-1 Flags: Fragment
66	cds.comp52908_c0_seq1|m.12748	3	3	A1YW13_DERFA | Der f 1 allergen
67	cds.comp108058_c0_seq1|m.33824	7	7	F5HJZ1_ANOGA | AGAP000801-PB Flags: Fragment
67	Der f 1.0104	7	7	Der f 1.0104
67	cds.comp18558_c0_seq1|m.4417	6	6	Q8MWR4_DERPT | Serine protease LM-1 Flags: Fragment
67	cds.comp128015_c0_seq1|m.44536	4	3	B7P3P1_IXOSC | Cathepsin B endopeptidase putative EC=3.4.22.1
67	cds.comp52908_c0_seq1|m.12748	3	3	A1YW13_DERFA | Der f 1 allergen
67	cds.comp13305_c0_seq1|m.3171	2	2	B3TFG6_9ACAR | Esterase TCE1
67	cds.comp56449_c0_seq1|m.13708	2	2	E2A5F2_CAMFO | Purine nucleoside phosphorylase
67	cds.comp128018_c0_seq1|m.44542	2	1	B7P3P0_IXOSC | Cathepsin B endopeptidase putative EC=3.4.22.1
67	cds.comp98258_c0_seq1|m.24905	2	2	L7UZA7_DERFA | Triosephosphate isomerase EC=5.3.1.1
67	Der f 6.0101	2	2	Der f 6.0101
67	cds.comp114969_c0_seq1|m.38342	2	2	Der f 36
67	cds.comp12936_c0_seq1|m.3079	2	2	L7M8L1_9ACAR | Putative gdp-l-fucose synthetase
68	cds.comp18558_c0_seq1|m.4417	5	5	Q8MWR4_DERPT | Serine protease LM-1 Flags: Fragment
68	cds.comp12936_c0_seq1|m.3079	5	5	L7M8L1_9ACAR | Putative gdp-l-fucose synthetase
68	cds.comp108058_c0_seq1|m.33824	4	4	F5HJZ1_ANOGA | AGAP000801-PB Flags: Fragment
68	cds.comp108059_c0_seq1|m.33825	4	4	F5HJZ1_ANOGA | AGAP000801-PB Flags: Fragment

Spot number	Accession	#Peptides	#Unique	Description
68	Der_f_1.0104	4	4	Der_f_1.0104
68	Der_f_1.0102	4	4	Der_f_1.0102
68	cds.comp98258_c0_seq1|m.24905	3	3	L7UZA7_DERFA | Triosephosphate isomerase EC=5.3.1.1
68	Der_f_6.0101	3	2	Der_f_6.0101
68	cds.comp52908_c0_seq1|m.12748	3	3	A1YW13_DERFA | Der f 1 allergen
68	cds.comp101558_c0_seq1|m.28079	3	3	B7Q0D4_IXOSC | Fumarylacetoacetase putative EC=3.7.1.2
68	cds.comp13305_c0_seq1|m.3171	2	2	B3TFG6_9ACAR | Esterase TCE1
68	cds.comp101628_c0_seq1|m.28106	2	2	no hit
68	cds.comp106751_c0_seq1|m.32734	2	2	no hit
68	cds.comp128016_c0_seq1|m.44537	2	2	B7P3P1_IXOSC | Cathepsin B endopeptidase putative EC=3.4.22.1
68	cds.comp112368_c0_seq1|m.36667	2	1	Q155V8_DERFA | Der f 6 Flags: Fragment
69	cds.comp106751_c0_seq1|m.32734	10	10	no hit
69	cds.comp18558_c0_seq1|m.4417	8	8	Q8MWR4_DERPT | Serine protease LM-1 Flags: Fragment
69	Der f 1.0104	3	3	Der f 1.0104
70	cds.comp106751_c0_seq1|m.32734	10	10	no hit
70	cds.comp18558_c0_seq1|m.4417	9	9	Q8MWR4_DERPT | Serine protease LM-1 Flags: Fragment
70	Der f 1.0104	3	3	Der f 1.0104
70	cds.comp5505_c0_seq1|m.1316	2	2	Q6QWP0_9ARAC | Glyceraldehyde-3-phosphate dehydrogenase EC=1.2.1.12 Flags: Fragment
70	cds.comp79187_c0_seq1|m.18650	2	2	E9IA80_SOLIN | Fructose-bisphosphate aldolase EC=4.1.2.13 Flags: Fragment

Spot number	Accession	#Peptides	#Unique	Description
71	cds.comp18558_c0_seq1|m.4417	8	8	Q8MWR4_DERPT | Serine protease LM-1 Flags: Fragment
71	Der f 1.0104	3	3	Der f 1.0104
72	cds.comp18558_c0_seq1|m.4417	10	10	Q8MWR4_DERPT | Serine protease LM-1 Flags: Fragment
72	Der f 1.0104	3	3	Der f 1.0104
73	Der f 2.0108	11	11	Der f 2.0108
73	Der f 25.0201	7	7	Der f 25.0201
73	cds.comp56403_c0_seq1|m.13691	6	6	Q16UH5_AEDAE | Proteasome subunit alpha type EC=3.4.25.1
73	cds.comp94542_c0_seq1|m.22071	6	6	F4WNF6_ACREC | Proteasome subunit alpha type EC=3.4.25.1 Flags: Fragment
73	cds.comp114973_c0_seq1|m.38346	5	5	Der f 36
73	cds.comp108218_c0_seq1|m.33973	4	4	G3MMG5_9ACAR | Proteasome subunit alpha type EC=3.4.25.1
73	cds.comp83879_c0_seq1|m.19673	3	3	R4G3J7_RHOPR | Putative gamma interferon inducible lysosomal thiol reductase gilt
73	cds.comp106751_c0_seq1|m.32734	3	3	no hit
73	cds.comp13305_c0_seq1|m.3171	2	2	B3TFG6_9ACAR | Esterase TCE1
73	cds.comp99324_c0_seq1|m.26033	2	2	A1KXH2_DERFA | Der f 1 allergen
73	cds.comp120707_c0_seq1|m.41344	2	2	B7PNT8_IXOSC | Glycerophosphoryl diester phosphodiesterase putative
73	cds.comp104007_c0_seq1|m.30355	2	2	L7M9E3_9ACAR | Putative beta adaptin
74	cds.comp98258_c0_seq1|m.24905	19	19	L7UZA7_DERFA | Triosephosphate isomerase EC=5.3.1.1
74	cds.comp126426_c0_seq1|m.43748	11	11	G3MFV0_9ACAR | Proteasome subunit alpha type EC=3.4.25.1 Flags: Fragment
74	cds.comp56403_c0_seq1|m.13691	9	9	Q16UH5_AEDAE | Proteasome subunit alpha type EC=3.4.25.1

Spot number	Accession	#Peptides	#Unique	Description
74	cds.comp83879_c0_seq1|m.19673	7	7	R4G3J7_RHOPR | Putative gamma interferon inducible lysosomal thiol reductase gilt
74	cds.comp106751_c0_seq1|m.32734	7	7	no hit
74	cds.comp109979_c0_seq1|m.35071	5	5	Q95PA6_AEDAE | Putative short-chain dehydrogenase/reductase Mc1
74	cds.comp108218_c0_seq1|m.33973	5	5	G3MMG5_9ACAR | Proteasome subunit alpha type EC=3.4.25.1
74	cds.comp94136_c0_seq1|m.21929	5	5	Q16FX9_AEDAE | 4-hydroxyphenylpyruvate dioxygenase
74	cds.comp88173_c0_seq1|m.20618	3	3	G3MLT2_9ACAR | Proteasome subunit alpha type EC=3.4.25.1
74	Der f 2.0109	3	3	Der f 2.0109
74	cds.comp104008_c0_seq1|m.30356	3	3	L7M9E3_9ACAR | Putative beta adaptin
74	cds.comp17339_c0_seq1|m.4137	3	3	B7PQV0_IXOSC | Alpha-aspartyl dipeptidase putative EC=3.4.13.21
74	cds.comp128015_c0_seq1|m.44536	3	3	B7P3P1_IXOSC | Cathepsin B endopeptidase putative EC=3.4.22.1
74	cds.comp100083_c0_seq1|m.26923	2	2	B7PM02_IXOSC | Proteasome subunit beta type EC=3.4.25.1
74	cds.comp99324_c0_seq1|m.26033	2	2	A1KXH2_DERFA | Der f 1 allergen
74	cds.comp114969_c0_seq1|m.38342	2	2	Der f 36
74	cds.comp67650_c0_seq1|m.16137	2	2	I4DKD3_PAPXU | Malate dehydrogenase EC=1.1.1.37
74	cds.comp97982_c0_seq1|m.24536	2	2	E2BH78_HARSA | Phosphatidylinositol-specific phospholipase C X domain-containing protein 1
74	cds.comp96258_c0_seq1|m.23052	2	2	L7M2A4_9ACAR | Putative alpha actinin
74	cds.comp34198_c0_seq1|m.8186	2	2	B7PEY0_IXOSC | AP-2 complex subunit alpha-1 putative
75	cds.comp112483_c0_seq1|m.36741	7	7	no hit
75	cds.comp114969_c0_seq1|m.38342	6	6	Der f 36

Spot number	Accession	#Peptides	#Unique	Description
75	cds.comp83879_c0_seq1|m.19673	5	5	R4G3J7_RHOPR | Putative gamma interferon inducible lysosomal thiol reductase gilt
75	cds.comp13305_c0_seq1|m.3171	3	3	B3TFG6_9ACAR | Esterase TCE1
75	cds.comp19576_c0_seq1|m.4639	3	3	Q3Y596_MACRS | Superoxide dismutase EC=1.15.1.1
75	Der f 2.0107	2	2	Der f 2.0107
75	cds.comp99324_c0_seq1|m.26033	2	2	A1KXH2_DERFA | Der f 1 allergen
75	cds.comp79187_c0_seq1|m.18650	2	2	E9IA80_SOLIN | Fructose-bisphosphate aldolase EC=4.1.2.13 Flags: Fragment
75	Der f 8.0101	2	2	Der f 8.0101
75	cds.comp86748_c0_seq1|m.20317	2	2	G6CVS9_DANPL | Legumaturain
75	cds.comp54005_c0_seq1|m.13051	2	2	B7QC64_IXOSC | Glutathione S-transferase kappa putative EC=2.5.1.18
76	cds.comp114973_c0_seq1|m.38346	8	8	Der f 36
76	cds.comp54005_c0_seq1|m.13051	4	4	B7QC64_IXOSC | Glutathione S-transferase kappa putative EC=2.5.1.18
76	cds.comp83879_c0_seq1|m.19673	3	3	R4G3J7_RHOPR | Putative gamma interferon inducible lysosomal thiol reductase gilt
76	Der f 1.0104	3	3	Der f 1.0104
76	cds.comp13305_c0_seq1|m.3171	2	2	B3TFG6_9ACAR | Esterase TCE1
76	cds.comp79187_c0_seq1|m.18650	2	2	E9IA80_SOLIN | Fructose-bisphosphate aldolase EC=4.1.2.13 Flags: Fragment
76	cds.comp104357_c0_seq1|m.30743	2	2	A2I427_MACHI | Putative hydroxysteroid (17-beta) dehydrogenase 8
76	cds.comp19576_c0_seq1|m.4639	2	2	Q3Y596_MACRS | Superoxide dismutase EC=1.15.1.1
76	Der f 8.0101	2	2	Der f 8.0101
76	cds.comp112483_c0_seq1|m.36741	2	2	no hit

Spot number	Accession	#Peptides	#Unique	Description
76	cds.comp90677_c0_seq1|m.21142	2	2	A7UI22_AMBAM | Lospin 7
77	cds.comp114973_c0_seq1|m.38346	10	10	Der f 36
77	Der f 1.0104	3	3	Der f 1.0104
77	cds.comp19576_c0_seq1|m.4639	2	2	Q3Y596_MACRS | Superoxide dismutase EC=1.15.1.1
77	Der f 8.0101	2	2	Der f 8.0101
78	cds.comp83879_c0_seq1|m.19673	6	6	R4G3J7_RHOPR | Putative gamma interferon inducible lysosomal thiol reductase gilt
78	cds.comp114969_c0_seq1|m.38342	6	6	Der f 36
78	cds.comp54005_c0_seq1|m.13051	6	6	B7QC64_IXOSC | Glutathione S-transferase kappa putative EC=2.5.1.18
78	cds.comp106751_c0_seq1|m.32734	3	3	no hit
78	cds.comp362_c0_seq1|m.164	2	2	Q2YFE3_DERPT | Glutathione transferase delta-like Dp7018E11
78	cds.comp99324_c0_seq1|m.26033	2	2	A1KXH2_DERFA | Der f 1 allergen
78	cds.comp79187_c0_seq1|m.18650	2	2	E9IA80_SOLIN | Fructose-bisphosphate aldolase EC=4.1.2.13 Flags: Fragment
78	Der f 8.0101	2	2	Der f 8.0101
78	cds.comp19576_c0_seq1|m.4639	2	2	Q3Y596_MACRS | Superoxide dismutase EC=1.15.1.1
78	cds.comp108218_c0_seq1|m.33973	2	2	G3MMG5_9ACAR | Proteasome subunit alpha type EC=3.4.25.1
79	cds.comp83879_c0_seq1|m.19673	10	10	R4G3J7_RHOPR | Putative gamma interferon inducible lysosomal thiol reductase gilt
79	Der f 2.0108	8	8	Der f 2.0108
79	cds.comp50993_c0_seq1|m.12321	3	3	J3JYL1_9CUCU | Superoxide dismutase [Cu-Zn] EC=1.15.1.1
79	Der f 22.0101	3	3	Der f 22.0101

Spot number	Accession	#Peptides	#Unique	Description
79	cds.comp99320_c0_seq1|m.26025	2	2	A1KXH2_DERFA | Der f 1 allergen
79	cds.comp106751_c0_seq1|m.32734	2	2	no hit
79	cds.comp114973_c0_seq1|m.38346	2	2	Der f 36
80	Der f 2.0108	9	9	Der f 2.0108
80	cds.comp80154_c0_seq1|m.18914	5	5	Der f 35
80	cds.comp103521_c0_seq1|m.29903	4	4	Q66RP5_TYRPU | Fatty acid-biding protein
80	Der f 22.0101	4	4	Der f 22.0101
80	cds.comp599_c0_seq1|m.238	2	2	B7Q9T4_IXOSC | Peptidyl-prolyl cis-trans isomerase
80	cds.comp99324_c0_seq1|m.26033	2	2	A1KXH2_DERFA | Der f 1 allergen
80	cds.comp151211_c0_seq1|m.51394	2	2	no hit
80	cds.comp86748_c0_seq1|m.20317	2	2	G6CVS9_DANPL | Legumaturain
80	cds.comp106751_c0_seq1|m.32734	2	2	no hit
80	cds.comp114969_c0_seq1|m.38342	2	2	Der f 36
81	Der f 2.0108	8	8	Der f 2.0108
81	cds.comp80154_c0_seq1|m.18914	8	8	Der f 35
81	cds.comp103521_c0_seq1|m.29903	5	5	Q66RP5_TYRPU | Fatty acid-biding protein
81	Der f 22.0101	4	4	Der f 22.0101
81	cds.comp9078_c0_seq1|m.2192	2	2	E9HLI4_DAPPU | Putative uncharacterized protein
81	cds.comp99324_c0_seq1|m.26033	2	2	A1KXH2_DERFA | Der f 1 allergen

Spot number	Accession	#Peptides	#Unique	Description
81	cds.comp151211_c0_seq1|m.51394	2	2	no hit
81	cds.comp86748_c0_seq1|m.20317	2	2	G6CVS9_DANPL	Legumaturain
82	cds.comp80154_c0_seq1|m.18914	8	8	Der f 35
82	Der f 2.0108	6	6	Der f 2.0108
82	cds.comp103521_c0_seq1|m.29903	4	4	Q66RP5_TYRPU	Fatty acid-biding protein
82	Der f 22.0101	4	4	Der f 22.0101
82	cds.comp99324_c0_seq1|m.26033	2	2	A1KXH2_DERFA	Der f 1 allergen
83	Der f 2.0108	12	12	Der f 2.0108
83	cds.comp54969_c0_seq1|m.13319	9	9	B7Q1I8_IXOSC	Rho GDP dissociation inhibitor putative Flags: Fragment
83	Der f 22.0101	5	5	Der f 22.0101
83	cds.comp110765_c0_seq1|m.35562	4	4	F4YSY7_9DIPT	Putative peroxiredoxin
83	cds.comp114969_c0_seq1|m.38342	4	4	Der f 36
83	cds.comp107643_c0_seq1|m.33453	2	2	D3TS01_GLOMM	Nucleoside diphosphate kinase
83	cds.comp99077_c0_seq1|m.25746	2	2	Q8MWR6_DERPT	14.5 kDa bacteriolytic enzyme
83	cds.comp80154_c0_seq1|m.18914	2	2	Der f 35
83	Der f 1.0104	2	2	Der f 1.0104
84	Der f 2.0108	10	10	Der f 2.0108
84	cds.comp110765_c0_seq1|m.35562	6	6	F4YSY7_9DIPT	Putative peroxiredoxin
84	Der f 22.0101	4	4	Der f 22.0101

Spot number	Accession	#Peptides	#Unique	Description
84	Der f 1.0104	4	4	Der f 1.0104
84	cds.comp114969_c0_seq1|m.38342	4	4	Der f 36
84	cds.comp103307_c0_seq1|m.29701	3	3	B7P417_IXOSC | Peritrophic membrane chitin binding protein putative
84	cds.comp80154_c0_seq1|m.18914	2	2	Der f 35
84	cds.comp83879_c0_seq1|m.19673	2	2	R4G3J7_RHOPR | Putative gamma interferon inducible lysosomal thiol reductase gilt
84	cds.comp102658_c0_seq1|m.28914	2	2	B4JZU2_DROGR | Bt
85	Der f 2.0108	11	11	Der f 2.0108
85	Der f 22.0101	4	4	Der f 22.0101
85	Der f 1.0104	3	3	Der f 1.0104
86	Der f 2.0108	15	2	Der f 2.0108
86	Der f 2.0107	14	0	Der f 2.0107
86	Der f 2.0102	13	0	Der f 2.0102
86	Der f 2.0109	12	0	Der f 2.0109
86	cds.comp114973_c0_seq1|m.38346	5	5	Der f 36
86	Der f 22.0101	3	3	Der f 22.0101
87	Der f 2.0108	6	6	Der f 2.0108
87	Der f 22.0101	5	5	Der f 22.0101
87	cds.comp102528_c0_seq1|m.28816	5	5	Der f 34
87	cds.comp151211_c0_seq1|m.51394	4	4	no hit

Spot number	Accession	#Peptides	#Unique	Description
87	Der f 1.0104	3	3	Der f 1.0104
87	Der f 14.0101	3	3	Der f 14.0101
87	cds.comp96036_c0_seq1|m.22609	2	2	E3UKG7_ERISI | Ubiquitin b SubName: Full=Ubiquitin/ribosomal L40 fusion protein
87	cds.comp128016_c0_seq1|m.44537	2	2	B7P3P1_IXOSC | Cathepsin B endopeptidase putative EC=3.4.22.1
88	cds.comp107298_c0_seq1|m.33085	4	4	CYTL_TACTR | L-cystatin Flags: Precursor
88	Der f 22.0101	4	4	Der f 22.0101
88	Der f 2.0107	2	2	Der f 2.0107
88	cds.comp113929_c0_seq1|m.37815	2	2	B4L991_DROMO | GI16804
89	Der f 22.0101	7	7	Der f 22.0101
89	Der f 2.0108	5	1	Der f 2.0108
89	Der f 2.0109	5	1	Der f 2.0109
89	cds.comp107299_c0_seq1|m.33086	2	2	CYTL_TACTR | L-cystatin Flags: Precursor
89	cds.comp102527_c0_seq1|m.28815	2	2	Der f 35
90	cds.comp151211_c0_seq1|m.51394	6	6	no hit
90	cds.comp848_c0_seq1|m.291	4	4	Q9GUA9_AMBAM | Macrophage migration inhibitory factor
90	Der f 22.0101	3	3	Der f 22.0101
90	cds.comp128013_c0_seq1|m.44526	3	3	B7P3P0_IXOSC | Cathepsin B endopeptidase putative EC=3.4.22.1
90	cds.comp128016_c0_seq1|m.44537	3	3	B7P3P1_IXOSC | Cathepsin B endopeptidase putative EC=3.4.22.1
91	cds.comp99077_c0_seq1|m.25746	2	2	Q8MWR6_DERPT | 14.5 kDa bacteriolytic enzyme

Spot number	Accession	#Peptides	#Unique	Description
91	cds.comp25488_c0_seq1|m.6081	2	2	no hit
92	cds.comp99077_c0_seq1|m.25746	2	2	Q8MWR6_DERPT | 14.5 kDa bacteriolytic enzyme
93	cds.comp105191_c0_seq1|m.31572	13	13	L7M0B5_9ACAR | Putative beta-glucocerebrosidase
93	Der f 4.0101	7	7	Der f 4.0101
93	cds.comp41295_c0_seq1|m.9906	6	6	B7QC45_IXOSC | Acetylcholinesterase putative EC=3.1.1.7 Flags: Fragment
93	cds.comp97082_c0_seq1|m.23801	5	5	B7PNW7_IXOSC | Beta-glucocerebrosidase putative EC=3.2.1.45
93	cds.comp13305_c0_seq1|m.3171	3	3	B3TFG6_9ACAR | Esterase TCE1
93	cds.comp32305_c0_seq1|m.7703	3	3	E2ABS8_CAMFO | Lysosomal alpha-mannosidase
93	cds.comp113240_c0_seq1|m.37386	2	2	Q8ISH5_ARAVE | Chitinase
94	cds.comp105191_c0_seq1|m.31572	12	12	L7M0B5_9ACAR | Putative beta-glucocerebrosidase
94	cds.comp119114_c0_seq1|m.40635	12	12	L7M2B8_9ACAR | Putative n-acylaminoacyl-peptide hydrolase
94	Der f 4.0101	10	10	Der f 4.0101
94	cds.comp41295_c0_seq1|m.9906	8	8	B7QC45_IXOSC | Acetylcholinesterase putative EC=3.1.1.7 Flags: Fragment
94	cds.comp97082_c0_seq1|m.23801	5	5	B7PNW7_IXOSC | Beta-glucocerebrosidase putative EC=3.2.1.45
94	cds.comp144500_c0_seq1|m.49975	5	5	E0W0P0_PEDHC | Plasma alpha-L-fucosidase putative EC=3.2.1.51
94	cds.comp113240_c0_seq1|m.37386	4	4	Q8ISH5_ARAVE | Chitinase
94	cds.comp13305_c0_seq1|m.3171	3	3	B3TFG6_9ACAR | Esterase TCE1
94	cds.comp108775_c0_seq1|m.34394	3	3	D6WCN1_TRICA | Beta-galactosidase EC=3.2.1.23
94	cds.comp103559_c0_seq1|m.29931	3	3	L7M0C6_9ACAR | Putative beta-glucocerebrosidase

Spot number	Accession	#Peptides	#Unique	Description
94	cds.comp144163_c0_seq1|m.49905	2	2	Q7PYX7_ANOGA	Dihydrolipoyl dehydrogenase EC=1.8.1.4 Flags: Fragment
94	Der f 1.0104	2	2	Der f 1.0104
