# Supplementary material for: A combined transcriptome and proteome analysis extends the allergome of house dust mite Dermatophagoides species
Source: PLoS One. 2017 Oct 5;12(10):e0185830. doi: 10.1371/journal.pone.0185830 (PMC5628879; doi:10.1371/journal.pone.0185830)
Supplement: S5 Table — A protein extract from whole D. pteronyssinus culture was submitted to two-dimensional gel electrophoresis. After staining with Sypro Ruby, gel plugs from 99 protein spots (S2 Fig) were recovered, trypsin digested then analyzed by LC-MS/MS. Numbers refer to the IgE-reactive (red cell) and non IgE-reactive (green cell) spots analyzed. Protein identification was performed using the species-specific transcriptome derived protein database, supplemented with IUIS-registered allergen sequences, as reference dataset. Only entries identified by a minimum of 2 peptides sequenced were taken into account. Proteins are reported by the entry name with the total numbers of supporting sequenced peptides (#peptides) and of uniquely mapping peptides (#unique) as well as, when available, the result of the annotation by blast analysis. (PDF) [file pone.0185830.s008.pdf]

Spot number	Accession	#Peptides	#Unique	Description
1	cds.comp4291_c0_seq1|m.6618	12	2	E2A599_CAMFO | Lysosomal alpha-glucosidase
1	cds.comp11083_c0_seq1|m.19764	8	8	L7MGV3_9ACAR | Putative glycosyl hydrolase family 38 Flags: Fragment
1	cds.comp4303_c0_seq1|m.6631	6	2	E2A599_CAMFO | Lysosomal alpha-glucosidase
1	cds.comp26721_c0_seq1|m.51414	5	5	G6D1V5_DANPL | Putative lysosomal alpha-mannosidase
1	cds.comp61015_c0_seq1|m.113575	5	5	G6CKF6_DANPL | Putative prolyl endopeptidase isoform 1
1	cds.comp61010_c0_seq1|m.113566	4	4	B7PDF5_IXOSC | Prolyl endopeptidase putative EC=3.4.21.26
1	cds.comp112467_c0_seq1|m.190255	4	4	L7M4Z6_9ACAR | Putative beta-mannosidase
1	cds.comp114923_c0_seq1|m.194060	2	2	Q9Y197_DERPT | Alpha-amylase Flags: Fragment
1	cds.comp86986_c0_seq1|m.155830	2	2	no hit
2	cds.comp4299_c0_seq1|m.6626	14	0	E2A599_CAMFO | Lysosomal alpha-glucosidase
2	cds.comp4291_c0_seq1|m.6618	11	0	E2A599_CAMFO | Lysosomal alpha-glucosidase
2	cds.comp4287_c0_seq1|m.6614	8	0	F4WH36_ACREC | Lysosomal alpha-glucosidase
2	cds.comp4292_c0_seq1|m.6619	8	0	E2A599_CAMFO | Lysosomal alpha-glucosidase
2	cds.comp4316_c0_seq1|m.6655	7	0	F4WH36_ACREC | Lysosomal alpha-glucosidase
2	cds.comp4313_c0_seq1|m.6652	7	0	F4WH36_ACREC | Lysosomal alpha-glucosidase
2	cds.comp11083_c0_seq1|m.19764	6	6	L7MGV3_9ACAR | Putative glycosyl hydrolase family 38 Flags: Fragment
2	cds.comp4303_c0_seq1|m.6631	5	5	E2A599_CAMFO | Lysosomal alpha-glucosidase
2	cds.comp26721_c0_seq1|m.51414	5	5	G6D1V5_DANPL | Putative lysosomal alpha-mannosidase
2	cds.comp4286_c0_seq1|m.6613	4	1	E2BEF1_HARSA | Lysosomal alpha-glucosidase Flags: Fragment

Spot number	Accession	#Peptides	#Unique	Description
2	cds.comp61010_c0_seq1|m.113566	3	3	B7PDF5_IXOSC | Prolyl endopeptidase putative EC=3.4.21.26
2	cds.comp4307_c0_seq1|m.6644	2	1	K7IPK3_NASVI | Uncharacterized protein
2	cds.comp61015_c0_seq1|m.113575	2	2	G6CKF6_DANPL | Putative prolyl endopeptidase isoform 1
3	cds.comp4299_c0_seq1|m.6626	13	0	E2A599_CAMFO | Lysosomal alpha-glucosidase
3	cds.comp26723_c0_seq1|m.51416	8	8	Q5TS83_ANOGA | AGAP008584-PA
3	cds.comp4292_c0_seq1|m.6619	8	0	E2A599_CAMFO | Lysosomal alpha-glucosidase
3	cds.comp61024_c0_seq1|m.113597	7	7	G6CKF6_DANPL | Putative prolyl endopeptidase isoform 1
3	cds.comp61025_c0_seq1|m.113599	7	7	G6CKF6_DANPL | Putative prolyl endopeptidase isoform 1
3	cds.comp61011_c0_seq1|m.113567	7	7	G6CKF6_DANPL | Putative prolyl endopeptidase isoform 1
3	cds.comp61027_c0_seq1|m.113603	7	7	G6CKF6_DANPL | Putative prolyl endopeptidase isoform 1
3	cds.comp61023_c0_seq1|m.113595	7	7	G6CKF6_DANPL | Putative prolyl endopeptidase isoform 1
3	cds.comp61016_c0_seq1|m.113577	4	4	B7PDF5_IXOSC | Prolyl endopeptidase putative EC=3.4.21.26
3	cds.comp115052_c0_seq1|m.194257	3	3	E2BBG1_HARSA | Filamin-C
3	cds.comp4307_c0_seq1|m.6644	2	1	K7IPK3_NASVI | Uncharacterized protein
3	cds.comp86986_c0_seq1|m.155830	2	2	no hit
3	cds.comp114923_c0_seq1|m.194060	2	2	Q9Y197_DERPT | Alpha-amylase Flags: Fragment
3	cds.comp4305_c0_seq1|m.6636	2	0	F4WH36_ACREC | Lysosomal alpha-glucosidase
3	cds.comp61018_c0_seq1|m.113586	2	2	E0VY04_PEDHC | Prolyl endopeptidase putative EC=3.4.21.26
4	cds.comp4299_c0_seq1|m.6626	8	0	E2A599_CAMFO | Lysosomal alpha-glucosidase

Spot number	Accession	#Peptides	#Unique	Description
4	cds.comp4291_c0_seq1|m.6618	8	0	E2A599_CAMFO | Lysosomal alpha-glucosidase
4	cds.comp4287_c0_seq1|m.6614	6	0	F4WH36_ACREC | Lysosomal alpha-glucosidase
4	cds.comp4303_c0_seq1|m.6631	6	1	E2A599_CAMFO | Lysosomal alpha-glucosidase
4	cds.comp4313_c0_seq1|m.6652	5	0	F4WH36_ACREC | Lysosomal alpha-glucosidase
4	cds.comp11083_c0_seq1|m.19764	5	5	L7MGV3_9ACAR | Putative glycosyl hydrolase family 38 Flags: Fragment
4	cds.comp4301_c0_seq1|m.6629	5	0	L7M201_9ACAR | Putative glucosidase ii catalytic alpha subunit
4	cds.comp26721_c0_seq1|m.51414	5	5	G6D1V5_DANPL | Putative lysosomal alpha-mannosidase
4	cds.comp4292_c0_seq1|m.6619	5	0	E2A599_CAMFO | Lysosomal alpha-glucosidase
4	cds.comp4316_c0_seq1|m.6655	4	0	F4WH36_ACREC | Lysosomal alpha-glucosidase
4	cds.comp61015_c0_seq1|m.113575	4	4	G6CKF6_DANPL | Putative prolyl endopeptidase isoform 1
4	cds.comp61010_c0_seq1|m.113566	4	4	B7PDF5_IXOSC | Prolyl endopeptidase putative EC=3.4.21.26
4	cds.comp4286_c0_seq1|m.6613	3	1	E2BEF1_HARSA | Lysosomal alpha-glucosidase Flags: Fragment
4	cds.comp112467_c0_seq1|m.190255	3	3	L7M4Z6_9ACAR | Putative beta-mannosidase
4	cds.comp86986_c0_seq1|m.155830	3	3	no hit
4	cds.comp115052_c0_seq1|m.194257	3	3	E2BBG1_HARSA | Filamin-C
4	cds.comp114923_c0_seq1|m.194060	3	3	Q9Y197_DERPT | Alpha-amylase Flags: Fragment
4	cds.comp4307_c0_seq1|m.6644	2	1	K7IPK3_NASVI | Uncharacterized protein
4	cds.comp4305_c0_seq1|m.6636	2	0	F4WH36_ACREC | Lysosomal alpha-glucosidase
5	Der p 15.0101	25	2	Q4JK69_DERPT Group 15 allergen protein short isoform OS=Dermatophagoides pteronyssinus PE=2 SV=1

Spot number	Accession	#Peptides	#Unique	Description
5	cds.comp57250_c0_seq1|m.106674	24	1	Q4JK69_DERPT | Group 15 allergen protein short isoform
5	cds.comp44312_c0_seq1|m.83626	13	13	B4MDC5_DROVI | GJ16197
5	cds.comp113853_c0_seq1|m.192441	10	10	no hit
5	cds.comp4299_c0_seq1|m.6626	9	1	E2A599_CAMFO | Lysosomal alpha-glucosidase
5	cds.comp113522_c0_seq1|m.191885	8	8	B0WIN7_CULQU | Peritrophic membrane chitin binding protein
5	cds.comp117832_c0_seq1|m.197937	8	8	B4LCX5_DROVI | GJ12921
5	cds.comp101940_c0_seq1|m.176964	8	8	L7M6G1_9ACAR | Putative dipeptidylpeptidase 3 strongylocentrotus purpuratus : similar to dipeptidylpeptidase 3
5	cds.comp4291_c0_seq1|m.6618	7	0	E2A599_CAMFO | Lysosomal alpha-glucosidase
5	cds.comp4303_c0_seq1|m.6631	6	6	E2A599_CAMFO | Lysosomal alpha-glucosidase
5	cds.comp4293_c0_seq1|m.6621	6	0	E2A599_CAMFO | Lysosomal alpha-glucosidase
5	cds.comp109962_c0_seq1|m.187492	5	5	B4PPZ1_DROYA | GE26259
5	cds.comp86986_c0_seq1|m.155830	5	5	no hit
5	cds.comp11083_c0_seq1|m.19764	4	4	L7MGV3_9ACAR | Putative glycosyl hydrolase family 38 Flags: Fragment
5	cds.comp114213_c0_seq1|m.193079	3	3	Q9U6R7_DERFA | 98kDa HDM allergen SubName: Full=Group 15 allergen Der f 15
5	cds.comp136643_c0_seq1|m.214661	3	3	E2C2J9_HARSA | Pancreatic triacylglycerol lipase
5	cds.comp75194_c0_seq1|m.137402	3	3	L7LXZ6_9ACAR | Beta-hexosaminidase EC=3.2.1.52
5	cds.comp26721_c0_seq1|m.51414	3	3	G6D1V5_DANPL | Putative lysosomal alpha-mannosidase
5	cds.comp86161_c0_seq1|m.154648	3	2	D6WGZ1_TRICA | Cathepsin B
5	cds.comp93718_c0_seq1|m.166217	3	3	ACES_ANOGA | Acetylcholinesterase Short=AChE EC=3.1.1.7 Flags: Precursor

Spot number	Accession	#Peptides	#Unique	Description
5	cds.comp118497_c0_seq1|m.198756	3	3	L7M225_9ACAR | Putative lysosomal pro-x carboxypeptidase-like protein
5	cds.comp86983_c0_seq1|m.155817	2	2	no hit
5	cds.comp4307_c0_seq1|m.6644	2	1	K7IPK3_NASVI | Uncharacterized protein
5	Der p 1.0107	2	2	Der p 1.0107
5	cds.comp86168_c0_seq1|m.154662	2	1	R4FNL2_RHOPR | Putative cathepsin b-like proteinase
5	cds.comp94280_c0_seq1|m.166903	2	2	ALL2_DERPT | Mite group 2 allergen Der p 2 AltName: Full=Allergen Der p II AltName: Full=DPX AltName: Allergen=Der p 2 Flags: Precursor
5	cds.comp71144_c0_seq1|m.130311	2	2	Q4JK71_DERPT | Group 18 allergen protein
6	cds.comp57250_c0_seq1|m.106674	24	24	Q4JK69_DERPT | Group 15 allergen protein short isoform
6	cds.comp44312_c0_seq1|m.83626	14	14	B4MDC5_DROVI | GJ16197
6	cds.comp4299_c0_seq1|m.6626	11	2	E2A599_CAMFO | Lysosomal alpha-glucosidase
6	cds.comp113522_c0_seq1|m.191885	10	10	B0WIN7_CULQU | Peritrophic membrane chitin binding protein
6	cds.comp117832_c0_seq1|m.197937	10	10	B4LCX5_DROVI | GJ12921
6	cds.comp4291_c0_seq1|m.6618	8	0	E2A599_CAMFO | Lysosomal alpha-glucosidase
6	cds.comp101940_c0_seq1|m.176964	5	5	L7M6G1_9ACAR | Putative dipeptidylpeptidase 3 strongylocentrotus purpuratus : similar to dipeptidylpeptidase 3
6	cds.comp109962_c0_seq1|m.187492	4	4	B4PPZ1_DROYA | GE26259
6	cds.comp86168_c0_seq1|m.154662	3	2	R4FNL2_RHOPR | Putative cathepsin b-like proteinase
6	cds.comp136643_c0_seq1|m.214661	3	3	E2C2J9_HARSA | Pancreatic triacylglycerol lipase
6	cds.comp114213_c0_seq1|m.193079	3	3	Q9U6R7_DERFA | 98kDa HDM allergen SubName: Full=Group 15 allergen Der f 15
6	cds.comp11083_c0_seq1|m.19764	3	3	L7MGV3_9ACAR | Putative glycosyl hydrolase family 38 Flags: Fragment

Spot number	Accession	#Peptides	#Unique	Description
6	Der p 2.0105	3	3	Der p 2.0105
6	cds.comp75194_c0_seq1|m.137402	2	2	L7LXZ6_9ACAR | Beta-hexosaminidase EC=3.2.1.52
6	cds.comp93716_c0_seq1|m.166215	2	2	R4ZGQ0_9ACAR | Acetylcholinesterase
6	cds.comp86172_c0_seq1|m.154673	2	1	R4FNL2_RHOPR | Putative cathepsin b-like proteinase
6	cds.comp4307_c0_seq1|m.6644	2	1	K7IPK3_NASVI | Uncharacterized protein
6	cds.comp141173_c0_seq1|m.216962	2	2	no hit
6	cds.comp61010_c0_seq1|m.113566	2	2	B7PDF5_IXOSC | Prolyl endopeptidase putative EC=3.4.21.26
7	cds.comp57250_c0_seq1|m.106674	11	11	Q4JK69_DERPT | Group 15 allergen protein short isoform
7	cds.comp44312_c0_seq1|m.83626	8	8	B4MDC5_DROVI | GJ16197
7	cds.comp4299_c0_seq1|m.6626	5	1	E2A599_CAMFO | Lysosomal alpha-glucosidase
7	cds.comp4291_c0_seq1|m.6618	5	0	E2A599_CAMFO | Lysosomal alpha-glucosidase
7	cds.comp117832_c0_seq1|m.197937	4	4	B4LCX5_DROVI | GJ12921
7	cds.comp4293_c0_seq1|m.6621	2	0	E2A599_CAMFO | Lysosomal alpha-glucosidase
7	cds.comp11083_c0_seq1|m.19764	2	2	L7MGV3_9ACAR | Putative glycosyl hydrolase family 38 Flags: Fragment
7	cds.comp113522_c0_seq1|m.191885	2	2	B0WIN7_CULQU | Peritrophic membrane chitin binding protein
7	cds.comp114213_c0_seq1|m.193079	2	2	Q9U6R7_DERFA | 98kDa HDM allergen SubName: Full=Group 15 allergen Der f 15
7	cds.comp86986_c0_seq1|m.155830	2	2	no hit
8	cds.comp57250_c0_seq1|m.106674	18	18	Q4JK69_DERPT | Group 15 allergen protein short isoform
8	cds.comp117832_c0_seq1|m.197937	14	14	B4LCX5_DROVI | GJ12921

Spot number	Accession	#Peptides	#Unique	Description
8	cds.comp44312_c0_seq1|m.83626	12	12	B4MDC5_DROVI | GJ16197
8	cds.comp4299_c0_seq1|m.6626	7	1	E2A599_CAMFO | Lysosomal alpha-glucosidase
8	cds.comp4291_c0_seq1|m.6618	7	1	E2A599_CAMFO | Lysosomal alpha-glucosidase
8	cds.comp4303_c0_seq1|m.6631	6	6	E2A599_CAMFO | Lysosomal alpha-glucosidase
8	cds.comp114213_c0_seq1|m.193079	4	4	Q9U6R7_DERFA | 98kDa HDM allergen SubName: Full=Group 15 allergen Der f 15
8	cds.comp4293_c0_seq1|m.6621	4	0	E2A599_CAMFO | Lysosomal alpha-glucosidase
8	cds.comp61010_c0_seq1|m.113566	4	4	B7PDF5_IXOSC | Prolyl endopeptidase putative EC=3.4.21.26
8	cds.comp86986_c0_seq1|m.155830	3	3	no hit
8	cds.comp113522_c0_seq1|m.191885	3	3	B0WIN7_CULQU | Peritrophic membrane chitin binding protein
8	cds.comp61015_c0_seq1|m.113575	3	3	G6CKF6_DANPL | Putative prolyl endopeptidase isoform 1
8	cds.comp11083_c0_seq1|m.19764	2	2	L7MGV3_9ACAR | Putative glycosyl hydrolase family 38 Flags: Fragment
8	cds.comp4307_c0_seq1|m.6644	2	1	K7IPK3_NASVI | Uncharacterized protein
8	cds.comp26721_c0_seq1|m.51414	2	2	G6D1V5_DANPL | Putative lysosomal alpha-mannosidase
8	cds.comp75194_c0_seq1|m.137402	2	2	L7LXZ6_9ACAR | Beta-hexosaminidase EC=3.2.1.52
8	cds.comp86983_c0_seq1|m.155817	2	2	comp86983_c0_seq1|g.155817 ORF comp86983_c0_seq1|g.155817 comp86983_c0_seq1|m.155817 type:5prime_partial len:87 (-) comp86983_c0_seq1:2532-2792(-)NoHit
8	cds.comp113849_c0_seq1|m.192438	2	2	no hit
8	cds.comp9912_c0_seq1|m.17778	2	2	Q0KKA6_HAELO | Leucine aminopeptidase
8	cds.comp61009_c0_seq1|m.113564	2	2	B7PDF5_IXOSC | Prolyl endopeptidase putative EC=3.4.21.26
9	cds.comp113522_c0_seq1|m.191885	27	27	B0WIN7_CULQU | Peritrophic membrane chitin binding protein

Spot number	Accession	#Peptides	#Unique	Description
9	cds.comp136643_c0_seq1|m.214661	19	19	E2C2J9_HARSA | Pancreatic triacylglycerol lipase
9	cds.comp57250_c0_seq1|m.106674	18	17	Q4JK69_DERPT | Group 15 allergen protein short isoform
9	cds.comp109962_c0_seq1|m.187492	13	13	B4PPZ1_DROYA | GE26259
9	cds.comp4299_c0_seq1|m.6626	7	2	E2A599_CAMFO | Lysosomal alpha-glucosidase
9	cds.comp4291_c0_seq1|m.6618	5	0	E2A599_CAMFO | Lysosomal alpha-glucosidase
9	cds.comp101940_c0_seq1|m.176964	4	4	L7M6G1_9ACAR | Putative dipeptidylpeptidase 3 strongylocentrotus purpuratus : similar to dipeptidylpeptidase 3
9	cds.comp86986_c0_seq1|m.155830	4	4	no hit
9	cds.comp4303_c0_seq1|m.6631	3	3	E2A599_CAMFO | Lysosomal alpha-glucosidase
9	cds.comp114213_c0_seq1|m.193079	3	3	Q9U6R7_DERFA | 98kDa HDM allergen SubName: Full=Group 15 allergen Der f 15
9	cds.comp4293_c0_seq1|m.6621	3	0	E2A599_CAMFO | Lysosomal alpha-glucosidase
9	cds.comp86983_c0_seq1|m.155817	2	2	no hit
9	cds.comp86172_c0_seq1|m.154673	2	2	R4FNL2_RHOPR | Putative cathepsin b-like proteinase
10	cds.comp113522_c0_seq1|m.191885	30	30	B0WIN7_CULQU | Peritrophic membrane chitin binding protein
10	cds.comp109962_c0_seq1|m.187492	19	19	B4PPZ1_DROYA | GE26259
10	cds.comp136643_c0_seq1|m.214661	18	18	E2C2J9_HARSA | Pancreatic triacylglycerol lipase
10	cds.comp57250_c0_seq1|m.106674	17	17	Q4JK69_DERPT | Group 15 allergen protein short isoform
10	cds.comp101938_c0_seq1|m.176963	9	9	L7M6G1_9ACAR | Putative dipeptidylpeptidase 3 strongylocentrotus purpuratus : similar to dipeptidylpeptidase 3
10	cds.comp4299_c0_seq1|m.6626	7	1	E2A599_CAMFO | Lysosomal alpha-glucosidase
10	cds.comp4291_c0_seq1|m.6618	6	0	E2A599_CAMFO | Lysosomal alpha-glucosidase

Spot number	Accession	#Peptides	#Unique	Description
10	cds.comp86986_c0_seq1|m.155830	5	5	no hit
10	cds.comp4303_c0_seq1|m.6631	4	4	E2A599_CAMFO | Lysosomal alpha-glucosidase
10	cds.comp4292_c0_seq1|m.6619	4	0	E2A599_CAMFO | Lysosomal alpha-glucosidase
10	cds.comp114213_c0_seq1|m.193079	3	3	Q9U6R7_DERFA | 98kDa HDM allergen SubName: Full=Group 15 allergen Der f 15
10	cds.comp86983_c0_seq1|m.155817	2	2	no hit
10	cds.comp86172_c0_seq1|m.154673	2	2	R4FNL2_RHOPR | Putative cathepsin b-like proteinase
10	cds.comp70036_c0_seq1|m.128549	2	2	B4Q0A3_DROYA | GE15791
10	cds.comp44312_c0_seq1|m.83626	2	2	B4MDC5_DROVI | GJ16197
11	cds.comp113522_c0_seq1|m.191885	20	20	B0WIN7_CULQU | Peritrophic membrane chitin binding protein
11	cds.comp57250_c0_seq1|m.106674	13	13	Q4JK69_DERPT | Group 15 allergen protein short isoform
11	cds.comp136643_c0_seq1|m.214661	12	12	E2C2J9_HARSA | Pancreatic triacylglycerol lipase
11	cds.comp109962_c0_seq1|m.187492	8	8	B4PPZ1_DROYA | GE26259
11	cds.comp101938_c0_seq1|m.176963	8	8	L7M6G1_9ACAR | Putative dipeptidylpeptidase 3 strongylocentrotus purpuratus : similar to dipeptidylpeptidase 3
11	cds.comp4299_c0_seq1|m.6626	4	1	E2A599_CAMFO | Lysosomal alpha-glucosidase
11	cds.comp4291_c0_seq1|m.6618	3	0	E2A599_CAMFO | Lysosomal alpha-glucosidase
11	cds.comp86986_c0_seq1|m.155830	3	3	no hit
11	cds.comp114213_c0_seq1|m.193079	2	2	Q9U6R7_DERFA | 98kDa HDM allergen SubName: Full=Group 15 allergen Der f 15
11	cds.comp86983_c0_seq1|m.155817	2	2	no hit
12				no identification

Spot number	Accession	#Peptides	#Unique	Description
13	cds.comp101938_c0_seq1|m.176963	29	29	L7M6G1_9ACAR | Putative dipeptidylpeptidase 3 strongylocentrotus purpuratus : similar to dipeptidylpeptidase 3
13	cds.comp136643_c0_seq1|m.214661	10	10	E2C2J9_HARSA | Pancreatic triacylglycerol lipase
13	cds.comp113522_c0_seq1|m.191885	6	6	B0WIN7_CULQU | Peritrophic membrane chitin binding protein
13	cds.comp53420_c0_seq1|m.100211	5	5	B4PC03_DROYA | GE11380
13	cds.comp15546_c0_seq1|m.28868	4	4	E9G305_DAPPU | Putative leukotriene A4 hydrolase EC=3.3.2.6
13	cds.comp110406_c0_seq1|m.187904	4	4	E2A0U6_CAMFO | N-acetylmuramoyl-L-alanine amidase
13	cds.comp86986_c0_seq1|m.155830	4	4	no hit
13	cds.comp86161_c0_seq1|m.154648	3	2	D6WGZ1_TRICA | Cathepsin B
13	cds.comp93718_c0_seq1|m.166217	3	3	ACES_ANOGA | Acetylcholinesterase Short=AChE EC=3.1.1.7 Flags: Precursor
13	cds.comp4299_c0_seq1|m.6626	2	1	E2A599_CAMFO | Lysosomal alpha-glucosidase
13	Der p 1.0107	2	2	Der p 1.0107
13	cds.comp75194_c0_seq1|m.137402	2	2	L7LXZ6_9ACAR | Beta-hexosaminidase EC=3.2.1.52
13	cds.comp57248_c0_seq1|m.106667	2	2	Q4JK70_DERPT | Group 15 allergen protein
13	cds.comp86983_c0_seq1|m.155817	2	2	no hit
13	cds.comp141173_c0_seq1|m.216962	2	2	no hit
13	cds.comp11139_c0_seq1|m.19850	2	2	B7P512_IXOSC | Acetylcholinesterase putative EC=3.1.1.7
13	cds.comp67063_c0_seq1|m.123525	2	2	B7PAR6_IXOSC | Heat shock protein putative EC=1.3.1.74
13	cds.comp61603_c0_seq1|m.114501	2	2	G6D480_DANPL | Putative ecdysteroid-inducible angiotensin-converting enzyme
13	cds.comp109962_c0_seq1|m.187492	2	2	B4PPZ1_DROYA | GE26259

Spot number	Accession	#Peptides	#Unique	Description
13	cds.comp86168_c0_seq1|m.154662	2	1	R4FNL2_RHOPR | Putative cathepsin b-like proteinase
13	cds.comp112921_c0_seq1|m.190980	2	2	B7PB45_IXOSC | Transferrin receptor putative EC=3.4.11.10
13	cds.comp117252_c0_seq1|m.197148	2	2	ALL2_PSOOV | Mite group 2 allergen Pso o 2 AltName: Full=Allergen Pso o A AltName: Allergen=Pso o 2 Flags: Precursor
14	cds.comp101938_c0_seq1|m.176963	40	40	L7M6G1_9ACAR | Putative dipeptidylpeptidase 3 strongylocentrotus purpuratus : similar to dipeptidylpeptidase 3
14	cds.comp136643_c0_seq1|m.214661	12	12	E2C2J9_HARSA | Pancreatic triacylglycerol lipase
14	cds.comp113522_c0_seq1|m.191885	7	7	B0WIN7_CULQU | Peritrophic membrane chitin binding protein
14	cds.comp4299_c0_seq1|m.6626	6	1	E2A599_CAMFO | Lysosomal alpha-glucosidase
14	cds.comp15546_c0_seq1|m.28868	6	6	E9G305_DAPPU | Putative leukotriene A4 hydrolase EC=3.3.2.6
14	cds.comp4291_c0_seq1|m.6618	5	0	E2A599_CAMFO | Lysosomal alpha-glucosidase
14	cds.comp86986_c0_seq1|m.155830	5	5	no hit
14	cds.comp110406_c0_seq1|m.187904	4	4	E2A0U6_CAMFO | N-acetylmuramoyl-L-alanine amidase
14	cds.comp141173_c0_seq1|m.216962	4	4	no hit
14	cds.comp4293_c0_seq1|m.6621	4	0	E2A599_CAMFO | Lysosomal alpha-glucosidase
14	cds.comp61603_c0_seq1|m.114501	3	3	G6D480_DANPL | Putative ecdysteroid-inducible angiotensin-converting enzyme
14	cds.comp75194_c0_seq1|m.137402	3	3	L7LXZ6_9ACAR | Beta-hexosaminidase EC=3.2.1.52
14	cds.comp86161_c0_seq1|m.154648	3	2	D6WGZ1_TRICA | Cathepsin B
14	cds.comp11139_c0_seq1|m.19850	3	3	B7P512_IXOSC | Acetylcholinesterase putative EC=3.1.1.7
14	cds.comp109962_c0_seq1|m.187492	3	3	B4PPZ1_DROYA | GE26259
14	cds.comp57248_c0_seq1|m.106667	3	3	Q4JK70_DERPT | Group 15 allergen protein

Spot number	Accession	#Peptides	#Unique	Description
14	Der p 6.0101	3	3	Der p 6.0101
14	Der p 1.0107	2	2	Der p 1.0107
14	cds.comp86168_c0_seq1|m.154662	2	1	R4FNL2_RHOPR | Putative cathepsin b-like proteinase
14	cds.comp86983_c0_seq1|m.155817	2	2	no hit
14	cds.comp112921_c0_seq1|m.190980	2	2	B7PB45_IXOSC | Transferrin receptor putative EC=3.4.11.10
14	cds.comp71144_c0_seq1|m.130311	2	2	Q4JK71_DERPT | Group 18 allergen protein
14	cds.comp141429_c0_seq1|m.217087	2	2	Q155V8_DERFA | Der f 6 Flags: Fragment
14	cds.comp117832_c0_seq1|m.197937	2	2	B4LCX5_DROVI | GJ12921
14	cds.comp117252_c0_seq1|m.197148	2	2	ALL2_PSOOV | Mite group 2 allergen Pso o 2 AltName: Full=Allergen Pso o A AltName: Allergen=Pso o 2 Flags: Precursor
14	cds.comp44313_c0_seq1|m.83628	2	2	no hit
14	cds.comp54249_c0_seq1|m.101582	2	2	no hit
15	cds.comp101938_c0_seq1|m.176963	34	34	L7M6G1_9ACAR | Putative dipeptidylpeptidase 3 strongylocentrotus purpuratus : similar to dipeptidylpeptidase 3
15	cds.comp136643_c0_seq1|m.214661	8	8	E2C2J9_HARSA | Pancreatic triacylglycerol lipase
15	cds.comp113522_c0_seq1|m.191885	8	8	B0WIN7_CULQU | Peritrophic membrane chitin binding protein
15	cds.comp15546_c0_seq1|m.28868	7	7	E9G305_DAPPU | Putative leukotriene A4 hydrolase EC=3.3.2.6
15	cds.comp86986_c0_seq1|m.155830	6	6	no hit
15	cds.comp109962_c0_seq1|m.187492	5	5	B4PPZ1_DROYA | GE26259
15	cds.comp4291_c0_seq1|m.6618	3	0	E2A599_CAMFO | Lysosomal alpha-glucosidase
15	cds.comp61603_c0_seq1|m.114501	3	3	G6D480_DANPL | Putative ecdysteroid-inducible angiotensin-converting enzyme

Spot number	Accession	#Peptides	#Unique	Description
15	cds.comp110406_c0_seq1|m.187904	3	3	E2A0U6_CAMFO | N-acetylmuramoyl-L-alanine amidase
15	cds.comp141173_c0_seq1|m.216962	3	3	no hit
15	Der p 1.0107	2	2	Der p 1.0107
15	cds.comp4299_c0_seq1|m.6626	2	0	E2A599_CAMFO | Lysosomal alpha-glucosidase
15	cds.comp75194_c0_seq1|m.137402	2	2	L7LXZ6_9ACAR | Beta-hexosaminidase EC=3.2.1.52
15	cds.comp4303_c0_seq1|m.6631	2	2	E2A599_CAMFO | Lysosomal alpha-glucosidase
15	cds.comp86161_c0_seq1|m.154648	2	1	D6WGZ1_TRICA | Cathepsin B
16	cds.comp101938_c0_seq1|m.176963	38	38	L7M6G1_9ACAR | Putative dipeptidylpeptidase 3 strongylocentrotus purpuratus : similar to dipeptidylpeptidase 3
16	cds.comp136643_c0_seq1|m.214661	9	9	E2C2J9_HARSA | Pancreatic triacylglycerol lipase
16	cds.comp4299_c0_seq1|m.6626	6	1	E2A599_CAMFO | Lysosomal alpha-glucosidase
16	cds.comp113522_c0_seq1|m.191885	6	6	B0WIN7_CULQU | Peritrophic membrane chitin binding protein
16	cds.comp61602_c0_seq1|m.114500	6	6	G6D480_DANPL | Putative ecdysteroid-inducible angiotensin-converting enzyme
16	cds.comp15546_c0_seq1|m.28868	5	5	E9G305_DAPPU | Putative leukotriene A4 hydrolase EC=3.3.2.6
16	cds.comp86986_c0_seq1|m.155830	5	5	no hit
16	cds.comp4291_c0_seq1|m.6618	4	0	E2A599_CAMFO | Lysosomal alpha-glucosidase
16	cds.comp75194_c0_seq1|m.137402	4	4	L7LXZ6_9ACAR | Beta-hexosaminidase EC=3.2.1.52
16	cds.comp11139_c0_seq1|m.19850	4	4	B7P512_IXOSC | Acetylcholinesterase putative EC=3.1.1.7
16	cds.comp4293_c0_seq1|m.6621	4	0	E2A599_CAMFO | Lysosomal alpha-glucosidase
16	cds.comp110406_c0_seq1|m.187904	3	3	E2A0U6_CAMFO | N-acetylmuramoyl-L-alanine amidase

Spot number	Accession	#Peptides	#Unique	Description
16	cds.comp141173_c0_seq1|m.216962	3	3	no hit
16	cds.comp94280_c0_seq1|m.166903	3	3	ALL2_DERPT | Mite group 2 allergen Der p 2 AltName: Full=Allergen Der p II AltName: Full=DPX AltName: Allergen=Der p 2 Flags: Precursor
16	cds.comp26721_c0_seq1|m.51414	3	3	G6D1V5_DANPL | Putative lysosomal alpha-mannosidase
16	cds.comp44313_c0_seq1|m.83628	3	3	no hit
16	Der p 1.0107	2	2	Der p 1.0107
16	cds.comp86983_c0_seq1|m.155817	2	2	no hit
16	cds.comp4303_c0_seq1|m.6631	2	2	E2A599_CAMFO | Lysosomal alpha-glucosidase
16	cds.comp71144_c0_seq1|m.130311	2	2	Q4JK71_DERPT | Group 18 allergen protein
16	cds.comp57248_c0_seq1|m.106667	2	2	Q4JK70_DERPT | Group 15 allergen protein
16	cds.comp100616_c0_seq1|m.175216	2	2	F4WLV0_ACREC | DNA-directed RNA polymerase EC=2.7.7.6 Flags: Fragment
16	cds.comp54251_c0_seq1|m.101586	2	2	no hit
17	cds.comp9909_c0_seq1|m.17773	12	12	Q0KKA6_HAELO | Leucine aminopeptidase
17	cds.comp45515_c0_seq1|m.85694	10	10	B4KNA8_DROMO | GI20802
17	cds.comp71144_c0_seq1|m.130311	9	9	Q4JK71_DERPT | Group 18 allergen protein
17	cds.comp113522_c0_seq1|m.191885	8	8	B0WIN7_CULQU | Peritrophic membrane chitin binding protein
17	cds.comp112630_c0_seq1|m.190474	7	7	L7M384_9ACAR | Putative biotinidase and vanin
17	cds.comp90671_c0_seq1|m.161623	6	6	E9FTN1_DAPPU | Beta-galactosidase EC=3.2.1.23
17	cds.comp132356_c0_seq1|m.211874	6	6	E2B4G2_HARSA | Plasma alpha-L-fucosidase
17	cds.comp86986_c0_seq1|m.155830	6	6	no hit

Spot number	Accession	#Peptides	#Unique	Description
17	cds.comp27063_c0_seq1|m.52228	5	5	no hit
17	cds.comp126379_c0_seq1|m.206831	5	5	L7M0C6_9ACAR | Putative beta-glucocerebrosidase
17	cds.comp56254_c0_seq1|m.105044	5	5	G6CSN9_DANPL | Hexokinase
17	cds.comp41866_c0_seq1|m.79355	5	5	no hit
17	cds.comp71718_c0_seq1|m.131367	4	4	C1BPZ3_9MAXI | Probable serine carboxypeptidase CPVL
17	cds.comp115055_c0_seq1|m.194263	4	4	E2BBG1_HARSA | Filamin-C
17	cds.comp138408_c0_seq1|m.215625	4	4	B7P9Z3_IXOSC | Phosphoribosylformylglycinamidine synthase putative EC=6.3.5.3
17	cds.comp101940_c0_seq1|m.176964	4	4	L7M6G1_9ACAR | Putative dipeptidylpeptidase 3 strongylocentrotus purpuratus : similar to dipeptidylpeptidase 3
17	cds.comp26721_c0_seq1|m.51414	4	4	G6D1V5_DANPL | Putative lysosomal alpha-mannosidase
17	cds.comp118497_c0_seq1|m.198756	4	4	L7M225_9ACAR | Putative lysosomal pro-x carboxypeptidase-like protein
17	cds.comp11933_c0_seq1|m.21531	4	3	B7PDZ6_IXOSC | Alpha-D-galactosidase putative EC=3.2.1.49 Flags: Fragment
17	cds.comp136643_c0_seq1|m.214661	4	4	E2C2J9_HARSA | Pancreatic triacylglycerol lipase
17	cds.comp92988_c0_seq1|m.165215	4	4	R4V1A4_COPFO | Enolase
17	cds.comp112733_c0_seq1|m.190676	4	4	B7P9Z3_IXOSC | Phosphoribosylformylglycinamidine synthase putative EC=6.3.5.3
17	cds.comp44313_c0_seq1|m.83628	4	4	no hit
17	cds.comp11139_c0_seq1|m.19850	4	4	B7P512_IXOSC | Acetylcholinesterase putative EC=3.1.1.7
17	cds.comp53420_c0_seq1|m.100211	4	4	B4PC03_DROYA | GE11380
17	cds.comp138947_c0_seq1|m.215905	3	3	E2BBG1_HARSA | Filamin-C
17	cds.comp115051_c0_seq1|m.194256	3	3	E2BBG1_HARSA | Filamin-C

Spot number	Accession	#Peptides	#Unique	Description
17	cds.comp86168_c0_seq1|m.154662	3	2	R4FNL2_RHOPR | Putative cathepsin b-like proteinase
17	cds.comp71098_c0_seq1|m.130257	3	2	B4YTT9_9ACAR | Heat shock protein 70-2
17	cds.comp56264_c0_seq1|m.105061	3	3	E7D1D8_LATHE | Hexokinase Flags: Fragment
17	cds.comp86161_c0_seq1|m.154648	3	2	D6WGZ1_TRICA | Cathepsin B
17	cds.comp11934_c0_seq1|m.21532	3	2	B7PDZ5_IXOSC | Alpha-D-galactosidase putative EC=3.2.1.49
17	cds.comp112464_c0_seq1|m.190252	3	3	L7M4Z6_9ACAR | Putative beta-mannosidase
17	Der p 1.0107	2	2	Der p 1.0107
17	cds.comp110928_c0_seq1|m.188689	2	2	A1KXH7_DERFA | Der f Alt a 10 allergen
17	cds.comp67072_c0_seq1|m.123542	2	1	M1KYK4_9HYME | Heat shock cognate 70
17	cds.comp86983_c0_seq1|m.155817	2	2	no hit
17	Der p 2.0105	2	2	Der p 2.0105
17	cds.comp117252_c0_seq1|m.197148	2	2	ALL2_PSOOV | Mite group 2 allergen Pso o 2 AltName: Full=Allergen Pso o A AltName: Allergen=Pso o 2 Flags: Precursor
17	cds.comp129554_c0_seq1|m.209679	2	2	B7PE53_IXOSC | Beta-hexosaminidase EC=3.2.1.52
17	cds.comp4291_c0_seq1|m.6618	2	2	E2A599_CAMFO | Lysosomal alpha-glucosidase
17	cds.comp112921_c0_seq1|m.190980	2	2	B7PB45_IXOSC | Transferrin receptor putative EC=3.4.11.10
17	Der p 6.0101	2	2	Der p 6.0101
18	cds.comp9909_c0_seq1|m.17773	12	12	Q0KKA6_HAELO | Leucine aminopeptidase
18	cds.comp45515_c0_seq1|m.85694	11	11	B4KNA8_DROMO | GI20802
18	cds.comp71144_c0_seq1|m.130311	8	8	Q4JK71_DERPT | Group 18 allergen protein

Spot number	Accession	#Peptides	#Unique	Description
18	cds.comp112630_c0_seq1|m.190474	8	8	L7M384_9ACAR | Putative biotinidase and vanin
18	cds.comp113522_c0_seq1|m.191885	7	7	B0WIN7_CULQU | Peritrophic membrane chitin binding protein
18	cds.comp92988_c0_seq1|m.165215	7	7	R4V1A4_COPFO | Enolase
18	cds.comp53420_c0_seq1|m.100211	7	7	B4PC03_DROYA | GE11380
18	cds.comp115055_c0_seq1|m.194263	6	6	E2BBG1_HARSA | Filamin-C
18	cds.comp132356_c0_seq1|m.211874	6	6	E2B4G2_HARSA | Plasma alpha-L-fucosidase
18	cds.comp11933_c0_seq1|m.21531	6	5	B7PDZ6_IXOSC | Alpha-D-galactosidase putative EC=3.2.1.49 Flags: Fragment
18	cds.comp86986_c0_seq1|m.155830	6	6	no hit
18	cds.comp138408_c0_seq1|m.215625	5	5	B7P9Z3_IXOSC | Phosphoribosylformylglycinamidine synthase putative EC=6.3.5.3
18	Der p 1.0107	4	4	Der p 1.0107
18	cds.comp71718_c0_seq1|m.131367	4	4	C1BPZ3_9MAXI | Probable serine carboxypeptidase CPVL
18	cds.comp126379_c0_seq1|m.206831	4	4	L7M0C6_9ACAR | Putative beta-glucocerebrosidase
18	cds.comp112733_c0_seq1|m.190676	4	4	B7P9Z3_IXOSC | Phosphoribosylformylglycinamidine synthase putative EC=6.3.5.3
18	cds.comp86161_c0_seq1|m.154648	4	3	D6WGZ1_TRICA | Cathepsin B
18	cds.comp26721_c0_seq1|m.51414	4	4	G6D1V5_DANPL | Putative lysosomal alpha-mannosidase
18	cds.comp27063_c0_seq1|m.52228	4	4	no hit
18	cds.comp11934_c0_seq1|m.21532	4	3	B7PDZ5_IXOSC | Alpha-D-galactosidase putative EC=3.2.1.49
18	cds.comp86168_c0_seq1|m.154662	3	2	R4FNL2_RHOPR | Putative cathepsin b-like proteinase
18	cds.comp138947_c0_seq1|m.215905	3	3	E2BBG1_HARSA | Filamin-C

Spot number	Accession	#Peptides	#Unique	Description
18	cds.comp11139_c0_seq1|m.19850	3	3	B7P512_IXOSC | Acetylcholinesterase putative EC=3.1.1.7
18	cds.comp56264_c0_seq1|m.105061	3	3	E7D1D8_LATHE | Hexokinase Flags: Fragment
18	cds.comp90671_c0_seq1|m.161623	3	3	E9FTN1_DAPPU | Beta-galactosidase EC=3.2.1.23
18	Der p 6.0101	3	3	Der p 6.0101
18	cds.comp44313_c0_seq1|m.83628	3	3	no hit
18	cds.comp41866_c0_seq1|m.79355	3	3	no hit
18	Der p 2.0105	3	3	Der p 2.0105
18	cds.comp86983_c0_seq1|m.155817	2	2	no hit
18	cds.comp117252_c0_seq1|m.197148	2	2	ALL2_PSOOV | Mite group 2 allergen Pso o 2 AltName: Full=Allergen Pso o A AltName: Allergen=Pso o 2 Flags: Precursor
18	cds.comp129554_c0_seq1|m.209679	2	2	B7PE53_IXOSC | Beta-hexosaminidase EC=3.2.1.52
18	cds.comp136643_c0_seq1|m.214661	2	2	E2C2J9_HARSA | Pancreatic triacylglycerol lipase
18	cds.comp56254_c0_seq1|m.105044	2	2	G6CSN9_DANPL | Hexokinase
18	cds.comp101940_c0_seq1|m.176964	2	2	L7M6G1_9ACAR | Putative dipeptidylpeptidase 3 strongylocentrotus purpuratus : similar to dipeptidylpeptidase 3
18	cds.comp72130_c0_seq1|m.132048	2	2	DERP3_DERPT | Mite allergen Der p 3 EC=3.4.21.- AltName: Full=Allergen Der p III AltName: Allergen=Der p 3 Flags: Precursor
18	cds.comp8012_c0_seq1|m.14281	2	2	PEPT1_EURMA | Peptidase 1 EC=3.4.22.65 AltName: Full=Allergen Eur m I AltName: Full=Mite group 1 allergen Eur m 1 AltName: Allergen=Eur m 1 Flags: Precursor
18	cds.comp112464_c0_seq1|m.190252	2	2	L7M4Z6_9ACAR | Putative beta-mannosidase
19	cds.comp112921_c0_seq1|m.190980	9	9	B7PB45_IXOSC | Transferrin receptor putative EC=3.4.11.10
19	cds.comp45515_c0_seq1|m.85694	9	9	B4KNA8_DROMO | GI20802
19	cds.comp113522_c0_seq1|m.191885	9	9	B0WIN7_CULQU | Peritrophic membrane chitin binding protein

Spot number	Accession	#Peptides	#Unique	Description
19	cds.comp26723_c0_seq1|m.51416	7	7	Q5TS83_ANOGA | AGAP008584-PA
19	cds.comp132356_c0_seq1|m.211874	7	7	E2B4G2_HARSA | Plasma alpha-L-fucosidase
19	cds.comp71144_c0_seq1|m.130311	6	6	Q4JK71_DERPT | Group 18 allergen protein
19	cds.comp11933_c0_seq1|m.21531	6	6	B7PDZ6_IXOSC | Alpha-D-galactosidase putative EC=3.2.1.49 Flags: Fragment
19	cds.comp9909_c0_seq1|m.17773	6	6	Q0KKA6_HAELO | Leucine aminopeptidase
19	cds.comp126379_c0_seq1|m.206831	5	5	L7M0C6_9ACAR | Putative beta-glucocerebrosidase
19	cds.comp136643_c0_seq1|m.214661	5	5	E2C2J9_HARSA | Pancreatic triacylglycerol lipase
19	cds.comp90671_c0_seq1|m.161623	5	5	E9FTN1_DAPPU | Beta-galactosidase EC=3.2.1.23
19	cds.comp11139_c0_seq1|m.19850	4	4	B7P512_IXOSC | Acetylcholinesterase putative EC=3.1.1.7
19	cds.comp86986_c0_seq1|m.155830	4	4	no hit
19	cds.comp112733_c0_seq1|m.190676	4	4	B7P9Z3_IXOSC | Phosphoribosylformylglycinamidine synthase putative EC=6.3.5.3
19	cds.comp138408_c0_seq1|m.215625	3	3	B7P9Z3_IXOSC | Phosphoribosylformylglycinamidine synthase putative EC=6.3.5.3
19	cds.comp115055_c0_seq1|m.194263	3	3	E2BBG1_HARSA | Filamin-C
19	cds.comp44313_c0_seq1|m.83628	3	3	no hit
19	cds.comp86168_c0_seq1|m.154662	3	2	R4FNL2_RHOPR | Putative cathepsin b-like proteinase
19	cds.comp11934_c0_seq1|m.21532	3	3	B7PDZ5_IXOSC | Alpha-D-galactosidase putative EC=3.2.1.49
19	cds.comp27063_c0_seq1|m.52228	3	3	no hit
19	cds.comp86161_c0_seq1|m.154648	3	2	D6WGZ1_TRICA | Cathepsin B
19	Der p 1.0107	2	2	Der p 1.0107

Spot number	Accession	#Peptides	#Unique	Description
19	cds.comp71718_c0_seq1|m.131367	2	2	C1BPZ3_9MAXI | Probable serine carboxypeptidase CPVL
19	cds.comp86983_c0_seq1|m.155817	2	2	no hit
19	cds.comp67063_c0_seq1|m.123525	2	2	B7PAR6_IXOSC | Heat shock protein putative EC=1.3.1.74
19	cds.comp11004_c0_seq1|m.19621	2	2	E0VRZ4_PEDHC | Predicted protein
19	cds.comp138947_c0_seq1|m.215905	2	2	E2BBG1_HARSA | Filamin-C
20	cds.comp45515_c0_seq1|m.85694	13	13	B4KNA8_DROMO | GI20802
20	cds.comp112921_c0_seq1|m.190980	10	10	B7PB45_IXOSC | Transferrin receptor putative EC=3.4.11.10
20	cds.comp132356_c0_seq1|m.211874	10	10	E2B4G2_HARSA | Plasma alpha-L-fucosidase
20	cds.comp126379_c0_seq1|m.206831	10	10	L7M0C6_9ACAR | Putative beta-glucocerebrosidase
20	cds.comp26723_c0_seq1|m.51416	10	10	Q5TS83_ANOGA | AGAP008584-PA
20	cds.comp9909_c0_seq1|m.17773	9	9	Q0KKA6_HAELO | Leucine aminopeptidase
20	cds.comp11933_c0_seq1|m.21531	5	5	B7PDZ6_IXOSC | Alpha-D-galactosidase putative EC=3.2.1.49 Flags: Fragment
20	cds.comp112733_c0_seq1|m.190676	5	5	B7P9Z3_IXOSC | Phosphoribosylformylglycinamidine synthase putative EC=6.3.5.3
20	cds.comp113522_c0_seq1|m.191885	5	5	B0WIN7_CULQU | Peritrophic membrane chitin binding protein
20	cds.comp86986_c0_seq1|m.155830	5	5	no hit
20	cds.comp11139_c0_seq1|m.19850	5	5	B7P512_IXOSC | Acetylcholinesterase putative EC=3.1.1.7
20	cds.comp86168_c0_seq1|m.154662	4	4	R4FNL2_RHOPR | Putative cathepsin b-like proteinase
20	cds.comp41866_c0_seq1|m.79355	4	4	no hit
20	cds.comp11004_c0_seq1|m.19621	3	3	E0VRZ4_PEDHC | Predicted protein

Spot number	Accession	#Peptides	#Unique	Description
20	cds.comp11934_c0_seq1|m.21532	3	3	B7PDZ5_IXOSC | Alpha-D-galactosidase putative EC=3.2.1.49
20	cds.comp44312_c0_seq1|m.83626	3	3	B4MDC5_DROVI | GJ16197
20	Der p 1.0107	2	2	Der p 1.0107
20	cds.comp138408_c0_seq1|m.215625	2	2	B7P9Z3_IXOSC | Phosphoribosylformylglycinamidine synthase putative EC=6.3.5.3
20	cds.comp129554_c0_seq1|m.209679	2	2	B7PE53_IXOSC | Beta-hexosaminidase EC=3.2.1.52
20	cds.comp86983_c0_seq1|m.155817	2	2	no hit
20	cds.comp136643_c0_seq1|m.214661	2	2	E2C2J9_HARSA | Pancreatic triacylglycerol lipase
20	cds.comp136645_c0_seq1|m.214664	2	2	E2C2J9_HARSA | Pancreatic triacylglycerol lipase
20	cds.comp112630_c0_seq1|m.190474	2	2	L7M384_9ACAR | Putative biotinidase and vanin
20	cds.comp129554_c0_seq1|m.209678	2	2	B7PE53_IXOSC | Beta-hexosaminidase EC=3.2.1.52
20	cds.comp92988_c0_seq1|m.165215	2	2	R4V1A4_COPFO | Enolase
20	cds.comp148443_c0_seq1|m.219638	2	2	E0W0P0_PEDHC | Plasma alpha-L-fucosidase putative EC=3.2.1.51
20	cds.comp53420_c0_seq1|m.100211	2	2	B4PC03_DROYA | GE11380
20	cds.comp94280_c0_seq1|m.166903	2	2	ALL2_DERPT | Mite group 2 allergen Der p 2 AltName: Full=Allergen Der p II AltName: Full=DPX AltName: Allergen=Der p 2 Flags: Precursor
20	cds.comp92389_c0_seq1|m.164353	2	2	D6WCM8_TRICA | Beta-galactosidase EC=3.2.1.23
20	cds.comp27085_c0_seq1|m.52277	2	2	no hit
21	cds.comp45515_c0_seq1|m.85694	11	11	B4KNA8_DROMO | GI20802
21	cds.comp126379_c0_seq1|m.206831	10	10	L7M0C6_9ACAR | Putative beta-glucocerebrosidase
21	cds.comp132356_c0_seq1|m.211874	9	9	E2B4G2_HARSA | Plasma alpha-L-fucosidase

Spot number	Accession	#Peptides	#Unique	Description
21	cds.comp112921_c0_seq1|m.190980	8	8	B7PB45_IXOSC | Transferrin receptor putative EC=3.4.11.10
21	cds.comp115055_c0_seq1|m.194263	8	8	E2BBG1_HARSA | Filamin-C
21	cds.comp26723_c0_seq1|m.51416	8	8	Q5TS83_ANOGA | AGAP008584-PA
21	cds.comp86986_c0_seq1|m.155830	4	4	no hit
21	cds.comp11933_c0_seq1|m.21531	4	4	B7PDZ6_IXOSC | Alpha-D-galactosidase putative EC=3.2.1.49 Flags: Fragment
21	Der p 1.0107	3	3	Der p 1.0107
21	cds.comp86164_c0_seq1|m.154651	3	3	B0W0V3_CULQU | Cathepsin L
21	cds.comp9909_c0_seq1|m.17773	3	3	Q0KKA6_HAELO | Leucine aminopeptidase
21	cds.comp138947_c0_seq1|m.215905	3	3	E2BBG1_HARSA | Filamin-C
21	cds.comp112846_c0_seq1|m.190822	2	2	L7M118_9ACAR | Putative purple acid phosphatase
21	cds.comp83774_c0_seq1|m.150923	2	2	L7MD56_9ACAR | Putative neural cell adhesion molecule l1 Flags: Fragment
21	cds.comp144454_c0_seq1|m.218370	2	2	B7PZG8_IXOSC | Aldehyde dehydrogenase putative EC=1.2.1.3
21	cds.comp11004_c0_seq1|m.19621	2	2	E0VRZ4_PEDHC | Predicted protein
21	cds.comp138408_c0_seq1|m.215625	2	2	B7P9Z3_IXOSC | Phosphoribosylformylglycinamidine synthase putative EC=6.3.5.3
21	cds.comp112733_c0_seq1|m.190676	2	2	B7P9Z3_IXOSC | Phosphoribosylformylglycinamidine synthase putative EC=6.3.5.3
21	cds.comp129554_c0_seq1|m.209679	2	2	B7PE53_IXOSC | Beta-hexosaminidase EC=3.2.1.52
21	cds.comp11139_c0_seq1|m.19850	2	2	B7P512_IXOSC | Acetylcholinesterase putative EC=3.1.1.7
21	cds.comp86983_c0_seq1|m.155817	2	2	no hit
21	cds.comp11934_c0_seq1|m.21532	2	2	B7PDZ5_IXOSC | Alpha-D-galactosidase putative EC=3.2.1.49

Spot number	Accession	#Peptides	#Unique	Description
21	cds.comp92389_c0_seq1|m.164353	2	2	D6WCM8_TRICA | Beta-galactosidase EC=3.2.1.23
21	cds.comp27085_c0_seq1|m.52277	2	2	no hit
21	cds.comp44313_c0_seq1|m.83628	2	2	no hit
21	cds.comp136643_c0_seq1|m.214661	2	2	E2C2J9_HARSA | Pancreatic triacylglycerol lipase
21	cds.comp58725_c0_seq1|m.109492	2	2	E9IA80_SOLIN | Fructose-bisphosphate aldolase EC=4.1.2.13 Flags: Fragment
22	cds.comp132356_c0_seq1|m.211874	13	13	E2B4G2_HARSA | Plasma alpha-L-fucosidase
22	cds.comp9909_c0_seq1|m.17773	12	12	Q0KKA6_HAELO | Leucine aminopeptidase
22	cds.comp126379_c0_seq1|m.206831	11	11	L7M0C6_9ACAR | Putative beta-glucocerebrosidase
22	cds.comp45515_c0_seq1|m.85694	10	10	B4KNA8_DROMO | GI20802
22	cds.comp112921_c0_seq1|m.190980	9	9	B7PB45_IXOSC | Transferrin receptor putative EC=3.4.11.10
22	cds.comp26723_c0_seq1|m.51416	7	7	Q5TS83_ANOGA | AGAP008584-PA
22	cds.comp11933_c0_seq1|m.21531	7	7	B7PDZ6_IXOSC | Alpha-D-galactosidase putative EC=3.2.1.49 Flags: Fragment
22	cds.comp112846_c0_seq1|m.190822	7	7	L7M118_9ACAR | Putative purple acid phosphatase
22	cds.comp11004_c0_seq1|m.19621	6	6	E0VRZ4_PEDHC | Predicted protein
22	cds.comp92988_c0_seq1|m.165215	6	6	R4V1A4_COPFO | Enolase
22	cds.comp134935_c0_seq1|m.213543	5	2	L7M118_9ACAR | Putative purple acid phosphatase
22	cds.comp86986_c0_seq1|m.155830	5	5	no hit
22	cds.comp144455_c0_seq1|m.218371	4	4	B7PZG8_IXOSC | Aldehyde dehydrogenase putative EC=1.2.1.3
22	cds.comp112733_c0_seq1|m.190676	4	4	B7P9Z3_IXOSC | Phosphoribosylformylglycinamidine synthase putative EC=6.3.5.3

Spot number	Accession	#Peptides	#Unique	Description
22	cds.comp134934_c0_seq1|m.213541	4	0	L7M118_9ACAR | Putative purple acid phosphatase
22	cds.comp134948_c0_seq1|m.213564	4	4	E0VLI0_PEDHC | Purple acid phosphatase putative
22	cds.comp11139_c0_seq1|m.19850	4	4	B7P512_IXOSC | Acetylcholinesterase putative EC=3.1.1.7
22	cds.comp11934_c0_seq1|m.21532	3	3	B7PDZ5_IXOSC | Alpha-D-galactosidase putative EC=3.2.1.49
22	cds.comp122816_c0_seq1|m.203611	3	3	C1IE32_9HEXA | Beta-1 3-D-glucanase EC=3.2.1.6 SubName: Full=Endo-beta-1 3-glucanase EC=3.2.1.39 Flags: Precursor
22	cds.comp92389_c0_seq1|m.164353	3	3	D6WCM8_TRICA | Beta-galactosidase EC=3.2.1.23
22	cds.comp44313_c0_seq1|m.83628	3	3	no hit
22	cds.comp129554_c0_seq1|m.209678	3	3	B7PE53_IXOSC | Beta-hexosaminidase EC=3.2.1.52
22	Der p 1.0107	2	2	Der p 1.0107
22	cds.comp129554_c0_seq1|m.209679	2	2	B7PE53_IXOSC | Beta-hexosaminidase EC=3.2.1.52
22	cds.comp86983_c0_seq1|m.155817	2	2	no hit
22	cds.comp115051_c0_seq1|m.194256	2	2	E2BBG1_HARSA | Filamin-C
22	cds.comp86164_c0_seq1|m.154651	2	2	B0W0V3_CULQU | Cathepsin L
22	cds.comp113522_c0_seq1|m.191885	2	2	B0WIN7_CULQU | Peritrophic membrane chitin binding protein
22	cds.comp58725_c0_seq1|m.109492	2	2	E9IA80_SOLIN | Fructose-bisphosphate aldolase EC=4.1.2.13 Flags: Fragment
22	cds.comp94280_c0_seq1|m.166903	2	2	ALL2_DERPT | Mite group 2 allergen Der p 2 AltName: Full=Allergen Der p II AltName: Full=DPX AltName: Allergen=Der p 2 Flags: Precursor
22	cds.comp134938_c0_seq1|m.213549	2	1	E0VL88_PEDHC | Acid phosphatase putative
22	cds.comp57967_c0_seq1|m.107892	2	2	MMSA_AEDAE | Probable methylmalonate-semialdehyde dehydrogenase [acylating] mitochondrial Short=MMSDH Short=Malonate-semialdehyde dehydrogenase [acylating] EC=1.2.1.18 EC=1.2.1.27 Flags: Precursor
22	cds.comp27085_c0_seq1|m.52277	2	2	no hit

Spot number	Accession	#Peptides	#Unique	Description
23	Der p 4.0101	23	23	Q9Y197_DERPT Alpha-amylase (Fragment) OS=Dermatophagoides pteronyssinus GN=group 4 allergen PE=2 SV=1
23	cds.comp75889_c0_seq1|m.138492	18	18	L7M6V9_9ACAR | Putative aldehyde dehydrogenase
23	cds.comp105891_c0_seq1|m.182028	3	3	G3MM22_9ACAR | Glucose-6-phosphate isomerase EC=5.3.1.9
23	cds.comp11083_c0_seq1|m.19764	2	2	L7MGV3_9ACAR | Putative glycosyl hydrolase family 38 Flags: Fragment
23	cds.comp105893_c0_seq1|m.182033	2	2	Q2F5I6_BOMMO | Glucose-6-phosphate isomerase EC=5.3.1.9
23	cds.comp86986_c0_seq1|m.155830	2	2	no hit
23	cds.comp128409_c0_seq1|m.208647	2	2	B7Q1V0_IXOSC | Acetylcholinesterase putative EC=3.1.1.7
23	cds.comp69948_c0_seq1|m.128434	2	2	L7MAC6_9ACAR | Putative glutamate/leucine/phenylalanine/valine dehydrogenase
23	cds.comp131981_c0_seq1|m.211670	2	2	C1BQY6_9MAXI | Arylsulfatase A
23	cds.comp128410_c0_seq1|m.208651	2	2	B7Q9J0_IXOSC | Acetylcholinesterase putative EC=3.1.1.7
24	Der p 4.0101	27	27	Q9Y197_DERPT Alpha-amylase (Fragment) OS=Dermatophagoides pteronyssinus GN=group 4 allergen PE=2 SV=1
24	cds.comp75889_c0_seq1|m.138492	19	19	L7M6V9_9ACAR | Putative aldehyde dehydrogenase
24	cds.comp105891_c0_seq1|m.182028	6	6	G3MM22_9ACAR | Glucose-6-phosphate isomerase EC=5.3.1.9
24	cds.comp105893_c0_seq1|m.182033	4	4	Q2F5I6_BOMMO | Glucose-6-phosphate isomerase EC=5.3.1.9
24	cds.comp128410_c0_seq1|m.208651	3	3	B7Q9J0_IXOSC | Acetylcholinesterase putative EC=3.1.1.7
24	cds.comp128409_c0_seq1|m.208647	2	2	B7Q1V0_IXOSC | Acetylcholinesterase putative EC=3.1.1.7
24	cds.comp4303_c0_seq1|m.6631	2	2	E2A599_CAMFO | Lysosomal alpha-glucosidase
24	cds.comp86986_c0_seq1|m.155830	2	2	no hit
24	cds.comp11085_c0_seq1|m.19768	2	2	F4W8Y5_ACREC | Lysosomal alpha-mannosidase

Spot number	Accession	#Peptides	#Unique	Description
24	cds.comp11083_c0_seq1|m.19764	2	2	L7MGV3_9ACAR | Putative glycosyl hydrolase family 38 Flags: Fragment
24	cds.comp69948_c0_seq1|m.128434	2	2	L7MAC6_9ACAR | Putative glutamate/leucine/phenylalanine/valine dehydrogenase
24	cds.comp92389_c0_seq1|m.164353	2	2	D6WCM8_TRICA | Beta-galactosidase EC=3.2.1.23
25	cds.comp114923_c0_seq1|m.194060	27	27	Q9Y197_DERPT | Alpha-amylase Flags: Fragment
25	cds.comp75889_c0_seq1|m.138492	25	25	L7M6V9_9ACAR | Putative aldehyde dehydrogenase
25	cds.comp105893_c0_seq1|m.182032	5	5	G6CUI0_DANPL | Glucose-6-phosphate isomerase EC=5.3.1.9
25	cds.comp86986_c0_seq1|m.155830	5	5	no hit
25	cds.comp128410_c0_seq1|m.208651	4	4	B7Q9J0_IXOSC | Acetylcholinesterase putative EC=3.1.1.7
25	cds.comp105893_c0_seq1|m.182033	4	4	Q2F5I6_BOMMO | Glucose-6-phosphate isomerase EC=5.3.1.9
25	cds.comp128409_c0_seq1|m.208647	3	3	B7Q1V0_IXOSC | Acetylcholinesterase putative EC=3.1.1.7
25	cds.comp11083_c0_seq1|m.19764	2	2	L7MGV3_9ACAR | Putative glycosyl hydrolase family 38 Flags: Fragment
25	cds.comp4303_c0_seq1|m.6631	2	2	E2A599_CAMFO | Lysosomal alpha-glucosidase
25	cds.comp109962_c0_seq1|m.187492	2	2	B4PPZ1_DROYA | GE26259
25	cds.comp72130_c0_seq1|m.132048	2	2	DERP3_DERPT | Mite allergen Der p 3 EC=3.4.21.- AltName: Full=Allergen Der p III AltName: Allergen=Der p 3 Flags: Precursor
26	cds.comp92988_c0_seq1|m.165215	19	19	R4V1A4_COPFO | Enolase
26	cds.comp9909_c0_seq1|m.17773	14	14	Q0KKA6_HAELO | Leucine aminopeptidase
26	cds.comp45515_c0_seq1|m.85694	7	7	B4KNA8_DROMO | GI20802
26	cds.comp122816_c0_seq1|m.203611	6	6	C1IE32_9HEXA | Beta-1 3-D-glucanase EC=3.2.1.6 SubName: Full=Endo-beta-1 3-glucanase EC=3.2.1.39 Flags: Precursor
26	cds.comp89229_c0_seq1|m.159476	6	6	L7M8R8_9ACAR | Adenosylhomocysteinase EC=3.3.1.1

Spot number	Accession	#Peptides	#Unique	Description
26	cds.comp126379_c0_seq1|m.206831	6	6	L7M0C6_9ACAR | Putative beta-glucocerebrosidase
26	cds.comp11933_c0_seq1|m.21531	6	6	B7PDZ6_IXOSC | Alpha-D-galactosidase putative EC=3.2.1.49 Flags: Fragment
26	cds.comp86986_c0_seq1|m.155830	5	5	no hit
26	cds.comp114923_c0_seq1|m.194060	5	5	Q9Y197_DERPT | Alpha-amylase Flags: Fragment
26	Der p 2.0105	4	4	Der p 2.0105
26	cds.comp9900_c0_seq1|m.17754	4	4	E0VXD2_PEDHC | Homogentisate 1 2-dioxygenase putative EC=1.13.11.5
26	cds.comp11934_c0_seq1|m.21532	3	3	B7PDZ5_IXOSC | Alpha-D-galactosidase putative EC=3.2.1.49
26	cds.comp110406_c0_seq1|m.187904	3	3	E2A0U6_CAMFO | N-acetylmuramoyl-L-alanine amidase
26	cds.comp4303_c0_seq1|m.6631	2	2	E2A599_CAMFO | Lysosomal alpha-glucosidase
26	cds.comp35248_c0_seq1|m.67971	2	2	D3TLY7_GLOMM | Putative serine protease
26	cds.comp86983_c0_seq1|m.155817	2	2	no hit
26	cds.comp134948_c0_seq1|m.213564	2	2	E0VLI0_PEDHC | Purple acid phosphatase putative
26	cds.comp85284_c0_seq1|m.153344	2	2	B7QMK0_IXOSC | Dipeptidyl peptidase IV putative EC=3.4.14.5 Flags: Fragment
26	cds.comp68560_c0_seq1|m.125791	2	2	no hit
26	cds.comp60484_c0_seq1|m.112775	2	2	L7MIP8_9ACAR | Putative beta-lactamase Flags: Fragment
26	cds.comp134934_c0_seq1|m.213541	2	0	L7M118_9ACAR | Putative purple acid phosphatase
26	cds.comp123134_c0_seq1|m.203950	2	2	L7M5B4_9ACAR | Putative 3-hydroxyacyl-coa dehydrogenase
26	cds.comp68560_c0_seq1|m.125793	2	2	no hit
26	cds.comp130991_c0_seq1|m.210835	2	2	L7MIP8_9ACAR | Putative beta-lactamase Flags: Fragment

Spot number	Accession	#Peptides	#Unique	Description
26	cds.comp134938_c0_seq1|m.213549	2	1	E0VL88_PEDHC | Acid phosphatase putative
27	cds.comp9909_c0_seq1|m.17773	25	25	Q0KKA6_HAELO | Leucine aminopeptidase
27	cds.comp92988_c0_seq1|m.165215	17	17	R4V1A4_COPFO | Enolase
27	cds.comp122816_c0_seq1|m.203611	6	6	C1IE32_9HEXA | Beta-1 3-D-glucanase EC=3.2.1.6 SubName: Full=Endo-beta-1 3-glucanase EC=3.2.1.39 Flags: Precursor
27	cds.comp86986_c0_seq1|m.155830	6	6	no hit
27	cds.comp9900_c0_seq1|m.17754	5	5	E0VXD2_PEDHC | Homogentisate 1 2-dioxygenase putative EC=1.13.11.5
27	cds.comp114923_c0_seq1|m.194060	5	5	Q9Y197_DERPT | Alpha-amylase Flags: Fragment
27	cds.comp45515_c0_seq1|m.85694	5	5	B4KNA8_DROMO | GI20802
27	cds.comp60483_c0_seq1|m.112772	3	3	L7MIP8_9ACAR | Putative beta-lactamase Flags: Fragment
27	Der p 2.0105	3	3	Der p 2.0105
27	cds.comp134935_c0_seq1|m.213543	3	1	L7M118_9ACAR | Putative purple acid phosphatase
27	cds.comp110406_c0_seq1|m.187904	3	3	E2A0U6_CAMFO | N-acetylmuramoyl-L-alanine amidase
27	cds.comp134934_c0_seq1|m.213541	3	0	L7M118_9ACAR | Putative purple acid phosphatase
27	cds.comp86983_c0_seq1|m.155817	2	2	no hit
27	cds.comp4303_c0_seq1|m.6631	2	2	E2A599_CAMFO | Lysosomal alpha-glucosidase
27	cds.comp85287_c0_seq1|m.153349	2	2	B7QMK0_IXOSC | Dipeptidyl peptidase IV putative EC=3.4.14.5 Flags: Fragment
27	cds.comp67063_c0_seq1|m.123525	2	2	B7PAR6_IXOSC | Heat shock protein putative EC=1.3.1.74
27	cds.comp103018_c0_seq1|m.178616	2	2	E2BUG0_HARSA | Titin
27	cds.comp112846_c0_seq1|m.190822	2	2	L7M118_9ACAR | Putative purple acid phosphatase

Spot number	Accession	#Peptides	#Unique	Description
27	cds.comp123134_c0_seq1|m.203950	2	2	L7M5B4_9ACAR | Putative 3-hydroxyacyl-coa dehydrogenase
27	cds.comp126380_c0_seq1|m.206833	2	2	L7M0C6_9ACAR | Putative beta-glucocerebrosidase
27	cds.comp130991_c0_seq1|m.210835	2	2	L7MIP8_9ACAR | Putative beta-lactamase Flags: Fragment
27	cds.comp59793_c0_seq1|m.111642	2	2	Q16M58_AEDAE | AAEL012418-PA
27	cds.comp68560_c0_seq1|m.125791	2	2	no hit
27	cds.comp134938_c0_seq1|m.213549	2	1	E0VL88_PEDHC | Acid phosphatase putative
28	cds.comp92988_c0_seq1|m.165215	27	27	R4V1A4_COPFO | Enolase
28	cds.comp9909_c0_seq1|m.17773	10	10	Q0KKA6_HAELO | Leucine aminopeptidase
28	cds.comp9900_c0_seq1|m.17754	7	7	E0VXD2_PEDHC | Homogentisate 1 2-dioxygenase putative EC=1.13.11.5
28	cds.comp114923_c0_seq1|m.194060	6	6	Q9Y197_DERPT | Alpha-amylase Flags: Fragment
28	cds.comp86986_c0_seq1|m.155830	5	5	no hit
28	cds.comp134948_c0_seq1|m.213564	3	3	E0VLI0_PEDHC | Purple acid phosphatase putative
28	cds.comp123134_c0_seq1|m.203950	3	3	L7M5B4_9ACAR | Putative 3-hydroxyacyl-coa dehydrogenase
28	cds.comp110406_c0_seq1|m.187904	3	3	E2A0U6_CAMFO | N-acetylmuramoyl-L-alanine amidase
28	cds.comp86983_c0_seq1|m.155817	2	2	no hit
28	cds.comp4303_c0_seq1|m.6631	2	2	E2A599_CAMFO | Lysosomal alpha-glucosidase
28	cds.comp58725_c0_seq1|m.109492	2	2	E9IA80_SOLIN | Fructose-bisphosphate aldolase EC=4.1.2.13 Flags: Fragment
28	cds.comp123115_c0_seq1|m.203925	2	2	L7M5B4_9ACAR | Putative 3-hydroxyacyl-coa dehydrogenase
28	cds.comp85287_c0_seq1|m.153349	2	2	B7QMK0_IXOSC | Dipeptidyl peptidase IV putative EC=3.4.14.5 Flags: Fragment

Spot number	Accession	#Peptides	#Unique	Description
28	Der p 2.0105	2	2	Der p 2.0105
28	cds.comp59793_c0_seq1|m.111642	2	2	Q16M58_AEDAE | AAEL012418-PA
28	cds.comp68560_c0_seq1|m.125793	2	2	no hit
28	cds.comp130991_c0_seq1|m.210835	2	2	L7MIP8_9ACAR | Putative beta-lactamase Flags: Fragment
28	cds.comp134938_c0_seq1|m.213549	2	1	E0VL88_PEDHC | Acid phosphatase putative
28	cds.comp68560_c0_seq1|m.125791	2	2	no hit
28	cds.comp27085_c0_seq1|m.52277	2	2	no hit
29	cds.comp85495_c0_seq1|m.153673	17	1	A1KXJ1_BLOTA | Blo t Mag29 allergen
29	cds.comp123877_c0_seq1|m.204692	16	16	E2BIZ1_HARSA | 4-hydroxyphenylpyruvate dioxygenase
29	cds.comp67049_c0_seq1|m.123493	15	8	F4WTV7_ACREC | Heat shock 70 kDa protein cognate 4
29	cds.comp71061_c0_seq1|m.130213	11	1	B4YTU0_9ACAR | Heat shock protein 70-3
29	cds.comp85485_c0_seq1|m.153656	10	0	E1ZZW6_CAMFO | Heat shock 70 kDa protein cognate 4
29	cds.comp112846_c0_seq1|m.190822	10	10	L7M118_9ACAR | Putative purple acid phosphatase
29	cds.comp71098_c0_seq1|m.130257	8	6	B4YTT9_9ACAR | Heat shock protein 70-2
29	cds.comp68560_c0_seq1|m.125791	8	8	no hit
29	cds.comp72134_c0_seq1|m.132054	8	1	DERP3_DERPT | Mite allergen Der p 3 EC=3.4.21.- AltName: Full=Allergen Der p III AltName: Allergen=Der p 3 Flags: Precursor
29	cds.comp72130_c0_seq1|m.132048	8	1	DERP3_DERPT | Mite allergen Der p 3 EC=3.4.21.- AltName: Full=Allergen Der p III AltName: Allergen=Der p 3 Flags: Precursor
29	cds.comp122816_c0_seq1|m.203611	6	6	C1IE32_9HEXA | Beta-1 3-D-glucanase EC=3.2.1.6 SubName: Full=Endo-beta-1 3-glucanase EC=3.2.1.39 Flags: Precursor
29	cds.comp86986_c0_seq1|m.155830	6	6	no hit

Spot number	Accession	#Peptides	#Unique	Description
29	cds.comp100562_c0_seq1|m.175130	5	5	B7Q8U6_IXOSC | Adenosine kinase putative EC=2.7.1.20
29	cds.comp92422_c0_seq1|m.164417	5	5	I1ZE47_DERPT | Heat shock protein cognate 5 Flags: Fragment
29	cds.comp77137_c0_seq1|m.140524	4	0	B7PAR6_IXOSC | Heat shock protein putative EC=1.3.1.74
29	Der p 2.0105	4	4	Der p 2.0105
29	cds.comp123134_c0_seq1|m.203950	4	4	L7M5B4_9ACAR | Putative 3-hydroxyacyl-coa dehydrogenase
29	cds.comp129401_c0_seq1|m.209549	4	4	B7PAQ0_IXOSC | Gamma-glutamyltransferase putative EC=2.3.2.2
29	cds.comp81915_c0_seq1|m.147992	3	3	G9C5G0_SCHGR | Aldo-keto reductase Flags: Fragment
29	cds.comp86983_c0_seq1|m.155817	2	2	no hit
29	cds.comp113914_c0_seq1|m.192570	2	2	B7PKP9_IXOSC | Glyceraldehyde-3-phosphate dehydrogenase EC=1.2.1.12
29	Der p 1.0107	2	2	Der p 1.0107
29	cds.comp68560_c0_seq1|m.125793	2	2	no hit
29	cds.comp108715_c0_seq1|m.185926	2	2	E2AX31_CAMFO | Nuclear pore membrane glycoprotein 210
29	cds.comp58725_c0_seq1|m.109492	2	2	E9IA80_SOLIN | Fructose-bisphosphate aldolase EC=4.1.2.13 Flags: Fragment
29	cds.comp27085_c0_seq1|m.52277	2	2	no hit
29	cds.comp110407_c0_seq1|m.187908	2	2	E9H6P1_DAPPU | Putative uncharacterized protein
29	cds.comp108598_c0_seq1|m.185810	2	2	L7LWN9_9ACAR | Putative arylsulfatase b
29	cds.comp134938_c0_seq1|m.213549	2	1	E0VL88_PEDHC | Acid phosphatase putative
29	cds.comp134934_c0_seq1|m.213541	2	0	L7M118_9ACAR | Putative purple acid phosphatase
29	cds.comp134935_c0_seq1|m.213543	2	1	L7M118_9ACAR | Putative purple acid phosphatase

Spot number	Accession	#Peptides	#Unique	Description
29	cds.comp59793_c0_seq1|m.111642	2	2	Q16M58_AEDAE | AAEL012418-PA
30	cds.comp85495_c0_seq1|m.153673	23	2	A1KXJ1_BLOTA | Blo t Mag29 allergen
30	cds.comp67049_c0_seq1|m.123493	15	10	F4WTV7_ACREC | Heat shock 70 kDa protein cognate 4
30	cds.comp85485_c0_seq1|m.153656	15	0	E1ZZW6_CAMFO | Heat shock 70 kDa protein cognate 4
30	cds.comp71061_c0_seq1|m.130213	11	1	B4YTU0_9ACAR | Heat shock protein 70-3
30	cds.comp92422_c0_seq1|m.164417	9	9	I1ZE47_DERPT | Heat shock protein cognate 5 Flags: Fragment
30	cds.comp122816_c0_seq1|m.203611	6	6	C1IE32_9HEXA | Beta-1 3-D-glucanase EC=3.2.1.6 SubName: Full=Endo-beta-1 3-glucanase EC=3.2.1.39 Flags: Precursor
30	cds.comp59793_c0_seq1|m.111642	6	6	Q16M58_AEDAE | AAEL012418-PA
30	cds.comp100562_c0_seq1|m.175130	6	6	B7Q8U6_IXOSC | Adenosine kinase putative EC=2.7.1.20
30	cds.comp123115_c0_seq1|m.203925	6	6	L7M5B4_9ACAR | Putative 3-hydroxyacyl-coa dehydrogenase
30	cds.comp86986_c0_seq1|m.155830	6	6	no hit
30	cds.comp58725_c0_seq1|m.109492	5	5	E9IA80_SOLIN | Fructose-bisphosphate aldolase EC=4.1.2.13 Flags: Fragment
30	cds.comp81915_c0_seq1|m.147992	5	5	G9C5G0_SCHGR | Aldo-keto reductase Flags: Fragment
30	cds.comp112990_c0_seq1|m.191090	5	5	L7M2B8_9ACAR | Putative n-acylaminoacyl-peptide hydrolase
30	cds.comp81913_c0_seq1|m.147990	4	4	R4WD77_9HEMI | Aldo-keto reductase
30	Der p 2.0105	3	3	Der p 2.0105
30	cds.comp114923_c0_seq1|m.194060	3	3	Q9Y197_DERPT | Alpha-amylase Flags: Fragment
30	cds.comp129401_c0_seq1|m.209549	3	3	B7PAQ0_IXOSC | Gamma-glutamyltransferase putative EC=2.3.2.2
30	cds.comp72130_c0_seq1|m.132048	3	3	DERP3_DERPT | Mite allergen Der p 3 EC=3.4.21.- AltName: Full=Allergen Der p III AltName: Allergen=Der p 3 Flags: Precursor

Spot number	Accession	#Peptides	#Unique	Description
30	cds.comp123876_c0_seq1|m.204690	3	3	H9HYH7_ATTCE | 4-hydroxyphenylpyruvate dioxygenase
30	cds.comp86983_c0_seq1|m.155817	2	2	no hit
30	cds.comp134935_c0_seq1|m.213543	2	2	L7M118_9ACAR | Putative purple acid phosphatase
30	cds.comp78049_c0_seq1|m.141912	2	2	Q9XXZ6_BOMMO | Polyubiquitin
30	cds.comp4303_c0_seq1|m.6631	2	2	E2A599_CAMFO | Lysosomal alpha-glucosidase
30	cds.comp110407_c0_seq1|m.187908	2	2	E9H6P1_DAPPU | Putative uncharacterized protein
30	cds.comp27085_c0_seq1|m.52277	2	2	no hit
30	cds.comp68560_c0_seq1|m.125793	2	2	no hit
30	cds.comp68560_c0_seq1|m.125791	2	2	no hit
30	cds.comp108598_c0_seq1|m.185810	2	2	L7LWN9_9ACAR | Putative arylsulfatase b
31	cds.comp85495_c0_seq1|m.153673	24	2	A1KXJ1_BLOTA | Blo t Mag29 allergen
31	cds.comp85485_c0_seq1|m.153656	16	0	E1ZZW6_CAMFO | Heat shock 70 kDa protein cognate 4
31	cds.comp67049_c0_seq1|m.123493	13	8	F4WTV7_ACREC | Heat shock 70 kDa protein cognate 4
31	cds.comp71061_c0_seq1|m.130213	11	1	B4YTU0_9ACAR | Heat shock protein 70-3
31	cds.comp92422_c0_seq1|m.164417	11	11	I1ZE47_DERPT | Heat shock protein cognate 5 Flags: Fragment
31	cds.comp100562_c0_seq1|m.175130	9	9	B7Q8U6_IXOSC | Adenosine kinase putative EC=2.7.1.20
31	cds.comp114923_c0_seq1|m.194060	7	7	Q9Y197_DERPT | Alpha-amylase Flags: Fragment
31	cds.comp81915_c0_seq1|m.147992	7	7	G9C5G0_SCHGR | Aldo-keto reductase Flags: Fragment
31	cds.comp112990_c0_seq1|m.191090	7	7	L7M2B8_9ACAR | Putative n-acylaminoacyl-peptide hydrolase

Spot number	Accession	#Peptides	#Unique	Description
31	cds.comp81913_c0_seq1|m.147990	7	7	R4WD77_9HEMI | Aldo-keto reductase
31	cds.comp59793_c0_seq1|m.111642	6	6	Q16M58_AEDAE | AAEL012418-PA
31	cds.comp123134_c0_seq1|m.203950	6	1	L7M5B4_9ACAR | Putative 3-hydroxyacyl-coa dehydrogenase
31	cds.comp86986_c0_seq1|m.155830	6	6	no hit
31	cds.comp123115_c0_seq1|m.203925	5	0	L7M5B4_9ACAR | Putative 3-hydroxyacyl-coa dehydrogenase
31	cds.comp131981_c0_seq1|m.211670	5	5	C1BQY6_9MAXI | Arylsulfatase A
31	cds.comp129401_c0_seq1|m.209549	3	3	B7PAQ0_IXOSC | Gamma-glutamyltransferase putative EC=2.3.2.2
31	cds.comp27063_c0_seq1|m.52228	3	3	no hit
31	cds.comp72133_c0_seq1|m.132053	3	3	DERP3_DERPT | Mite allergen Der p 3 EC=3.4.21.- AltName: Full=Allergen Der p III AltName: Allergen=Der p 3 Flags: Precursor
31	cds.comp86983_c0_seq1|m.155817	2	2	no hit
31	cds.comp4303_c0_seq1|m.6631	2	2	E2A599_CAMFO | Lysosomal alpha-glucosidase
31	cds.comp113914_c0_seq1|m.192570	2	2	B7PKP9_IXOSC | Glyceraldehyde-3-phosphate dehydrogenase EC=1.2.1.12
31	cds.comp110407_c0_seq1|m.187908	2	2	E9H6P1_DAPPU | Putative uncharacterized protein
31	cds.comp58725_c0_seq1|m.109492	2	2	E9IA80_SOLIN | Fructose-bisphosphate aldolase EC=4.1.2.13 Flags: Fragment
31	cds.comp61446_c0_seq1|m.114264	2	2	B7PAQ0_IXOSC | Gamma-glutamyltransferase putative EC=2.3.2.2
31	cds.comp108598_c0_seq1|m.185810	2	2	L7LWN9_9ACAR | Putative arylsulfatase b
32	cds.comp58725_c0_seq1|m.109492	12	12	E9IA80_SOLIN | Fructose-bisphosphate aldolase EC=4.1.2.13 Flags: Fragment
32	cds.comp110928_c0_seq1|m.188689	7	7	A1KXH7_DERFA | Der f Alt a 10 allergen
32	cds.comp86168_c0_seq1|m.154662	7	0	R4FNL2_RHOPR | Putative cathepsin b-like proteinase

Spot number	Accession	#Peptides	#Unique	Description
32	cds.comp112630_c0_seq1|m.190474	7	7	L7M384_9ACAR | Putative biotinidase and vanin
32	cds.comp86166_c0_seq1|m.154654	7	1	R4FNL2_RHOPR | Putative cathepsin b-like proteinase
32	cds.comp72130_c0_seq1|m.132048	7	1	DERP3_DERPT | Mite allergen Der p 3 EC=3.4.21.- AltName: Full=Allergen Der p III AltName: Allergen=Der p 3 Flags: Precursor
32	cds.comp44312_c0_seq1|m.83626	6	6	B4MDC5_DROVI | GJ16197
32	cds.comp72133_c0_seq1|m.132053	6	0	DERP3_DERPT | Mite allergen Der p 3 EC=3.4.21.- AltName: Full=Allergen Der p III AltName: Allergen=Der p 3 Flags: Precursor
32	cds.comp11103_c0_seq1|m.19801	5	5	B0X973_CULQU | Lysosomal alpha-mannosidase
32	cds.comp70828_c0_seq1|m.129947	4	4	Q6PPI6_HOMVI | Putative cytoplasmic actin A3a1
32	cds.comp71144_c0_seq1|m.130311	4	4	Q4JK71_DERPT | Group 18 allergen protein
32	Der p 2.0105	4	4	Der p 2.0105
32	cds.comp86986_c0_seq1|m.155830	4	4	no hit
32	cds.comp86161_c0_seq1|m.154648	4	3	D6WGZ1_TRICA | Cathepsin B
32	cds.comp90671_c0_seq1|m.161623	4	4	E9FTN1_DAPPU | Beta-galactosidase EC=3.2.1.23
32	cds.comp120606_c0_seq1|m.201109	3	3	Q17G61_AEDAE | AAEL003193-PA
32	cds.comp71098_c0_seq1|m.130257	3	3	B4YTT9_9ACAR | Heat shock protein 70-2
32	cds.comp136643_c0_seq1|m.214661	3	3	E2C2J9_HARSA | Pancreatic triacylglycerol lipase
32	cds.comp113914_c0_seq1|m.192570	3	3	B7PKP9_IXOSC | Glyceraldehyde-3-phosphate dehydrogenase EC=1.2.1.12
32	cds.comp47520_c0_seq1|m.89357	3	3	A9QW25_CARMA | Glycosyl-phosphatidylinositol-linked carbonic anhydrase EC=4.2.1.1
32	cds.comp27063_c0_seq1|m.52228	3	3	no hit
32	cds.comp81731_c0_seq1|m.147748	3	3	L7MB43_9ACAR | Putative glycerol-3-phosphate dehydrogenase/dihydroxyacetone 3-phosphate reductase

Spot number	Accession	#Peptides	#Unique	Description
32	Der p 1.0107	2	2	Der p 1.0107
32	cds.comp11139_c0_seq1|m.19850	2	2	B7P512_IXOSC | Acetylcholinesterase putative EC=3.1.1.7
32	cds.comp86983_c0_seq1|m.155817	2	2	no hit
32	cds.comp90809_c0_seq1|m.161776	2	2	L7M2J0_9ACAR | Putative aminopeptidase of the m17 family
32	cds.comp8071_c0_seq1|m.14356	2	1	PEPT1_EURMA | Peptidase 1 EC=3.4.22.65 AltName: Full=Allergen Eur m I AltName: Full=Mite group 1 allergen Eur m 1 AltName: Allergen=Eur m 1 Flags: Precursor
32	cds.comp105855_c0_seq1|m.181988	2	2	A1IHK5_HAELO | Serine carboxypeptidase
32	cds.comp8012_c0_seq1|m.14281	2	1	PEPT1_EURMA | Peptidase 1 EC=3.4.22.65 AltName: Full=Allergen Eur m I AltName: Full=Mite group 1 allergen Eur m 1 AltName: Allergen=Eur m 1 Flags: Precursor
32	cds.comp4557_c0_seq1|m.7090	2	2	no hit
32	Der p 6.0101	2	2	Der p 6.0101
32	cds.comp11933_c0_seq1|m.21531	2	2	B7PDZ6_IXOSC | Alpha-D-galactosidase putative EC=3.2.1.49 Flags: Fragment
32	cds.comp110406_c0_seq1|m.187904	2	2	E2A0U6_CAMFO | N-acetylmuramoyl-L-alanine amidase
32	cds.comp113522_c0_seq1|m.191885	2	2	B0WIN7_CULQU | Peritrophic membrane chitin binding protein
32	cds.comp92983_c0_seq1|m.165211	2	2	R4V1A4_COPFO | Enolase
33	cds.comp58725_c0_seq1|m.109492	20	20	E9IA80_SOLIN | Fructose-bisphosphate aldolase EC=4.1.2.13 Flags: Fragment
33	cds.comp86168_c0_seq1|m.154662	8	0	R4FNL2_RHOPR | Putative cathepsin b-like proteinase
33	cds.comp86166_c0_seq1|m.154654	8	1	R4FNL2_RHOPR | Putative cathepsin b-like proteinase
33	cds.comp110928_c0_seq1|m.188689	7	7	A1KXH7_DERFA | Der f Alt a 10 allergen
33	cds.comp71098_c0_seq1|m.130257	6	6	B4YTT9_9ACAR | Heat shock protein 70-2
33	cds.comp86986_c0_seq1|m.155830	6	6	no hit

Spot number	Accession	#Peptides	#Unique	Description
33	cds.comp11103_c0_seq1|m.19801	5	5	B0X973_CULQU | Lysosomal alpha-mannosidase
33	Der p 2.0112	5	5	Der p 2.0112
33	cds.comp90809_c0_seq1|m.161776	5	5	L7M2J0_9ACAR | Putative aminopeptidase of the m17 family
33	cds.comp112630_c0_seq1|m.190474	5	5	L7M384_9ACAR | Putative biotinidase and vanin
33	cds.comp120606_c0_seq1|m.201109	5	5	Q17G61_AEDAE | AAEL003193-PA
33	cds.comp72130_c0_seq1|m.132048	4	4	DERP3_DERPT | Mite allergen Der p 3 EC=3.4.21.- AltName: Full=Allergen Der p III AltName: Allergen=Der p 3 Flags: Precursor
33	cds.comp86161_c0_seq1|m.154648	4	3	D6WGZ1_TRICA | Cathepsin B
33	cds.comp90671_c0_seq1|m.161623	4	4	E9FTN1_DAPPU | Beta-galactosidase EC=3.2.1.23
33	Der p 6.0101	4	4	Der p 6.0101
33	Der p 1.0107	3	3	Der p 1.0107
33	cds.comp128133_c0_seq1|m.208437	3	3	C1IE32_9HEXA | Beta-1 3-D-glucanase EC=3.2.1.6 SubName: Full=Endo-beta-1 3-glucanase EC=3.2.1.39 Flags: Precursor
33	cds.comp44313_c0_seq1|m.83628	3	3	no hit
33	cds.comp122816_c0_seq1|m.203611	3	3	C1IE32_9HEXA | Beta-1 3-D-glucanase EC=3.2.1.6 SubName: Full=Endo-beta-1 3-glucanase EC=3.2.1.39 Flags: Precursor
33	cds.comp27085_c0_seq1|m.52277	3	3	no hit
33	cds.comp8012_c0_seq1|m.14281	2	2	PEPT1_EURMA | Peptidase 1 EC=3.4.22.65 AltName: Full=Allergen Eur m I AltName: Full=Mite group 1 allergen Eur m 1 AltName: Allergen=Eur m 1 Flags: Precursor
33	cds.comp8071_c0_seq1|m.14356	2	2	PEPT1_EURMA | Peptidase 1 EC=3.4.22.65 AltName: Full=Allergen Eur m I AltName: Full=Mite group 1 allergen Eur m 1 AltName: Allergen=Eur m 1 Flags: Precursor
33	cds.comp68825_c0_seq1|m.126306	2	2	B4J0B5_DROGR | GH15860
33	cds.comp86983_c0_seq1|m.155817	2	2	no hit
33	cds.comp136643_c0_seq1|m.214661	2	2	E2C2J9_HARSA | Pancreatic triacylglycerol lipase

Spot number	Accession	#Peptides	#Unique	Description
33	cds.comp70753_c0_seq1|m.129809	2	2	B2XY22_9ARAC | Actin Flags: Fragment
33	cds.comp11139_c0_seq1|m.19850	2	2	B7P512_IXOSC | Acetylcholinesterase putative EC=3.1.1.7
33	cds.comp81731_c0_seq1|m.147748	2	2	L7MB43_9ACAR | Putative glycerol-3-phosphate dehydrogenase/dihydroxyacetone 3-phosphate reductase
33	cds.comp11973_c0_seq1|m.21633	2	2	E2B637_HARSA | Alpha-N-acetylgalactosaminidase
33	cds.comp124461_c0_seq1|m.205142	2	2	A1YW13_DERFA | Der f 1 allergen
34	cds.comp58725_c0_seq1|m.109492	16	16	E9IA80_SOLIN | Fructose-bisphosphate aldolase EC=4.1.2.13 Flags: Fragment
34	cds.comp110928_c0_seq1|m.188689	8	7	A1KXH7_DERFA | Der f Alt a 10 allergen
34	cds.comp16912_c0_seq1|m.31604	8	8	D3TS39_GLOMM | Adenosine kinase
34	cds.comp86168_c0_seq1|m.154662	7	0	R4FNL2_RHOPR | Putative cathepsin b-like proteinase
34	cds.comp86166_c0_seq1|m.154654	7	1	R4FNL2_RHOPR | Putative cathepsin b-like proteinase
34	cds.comp90792_c0_seq1|m.161731	7	7	L7M2J0_9ACAR | Putative aminopeptidase of the m17 family
34	cds.comp72130_c0_seq1|m.132048	7	1	DERP3_DERPT | Mite allergen Der p 3 EC=3.4.21.- AltName: Full=Allergen Der p III AltName: Allergen=Der p 3 Flags: Precursor
34	cds.comp72133_c0_seq1|m.132053	6	0	DERP3_DERPT | Mite allergen Der p 3 EC=3.4.21.- AltName: Full=Allergen Der p III AltName: Allergen=Der p 3 Flags: Precursor
34	Der p 2.0106	5	5	Der p 2.0106
34	cds.comp90671_c0_seq1|m.161623	5	5	E9FTN1_DAPPU | Beta-galactosidase EC=3.2.1.23
34	cds.comp11103_c0_seq1|m.19801	4	4	B0X973_CULQU | Lysosomal alpha-mannosidase
34	cds.comp113914_c0_seq1|m.192570	4	4	B7PKP9_IXOSC | Glyceraldehyde-3-phosphate dehydrogenase EC=1.2.1.12
34	cds.comp86986_c0_seq1|m.155830	4	4	no hit
34	cds.comp86161_c0_seq1|m.154648	3	2	D6WGZ1_TRICA | Cathepsin B

Spot number	Accession	#Peptides	#Unique	Description
34	cds.comp71098_c0_seq1|m.130257	3	3	B4YTT9_9ACAR | Heat shock protein 70-2
34	cds.comp122816_c0_seq1|m.203611	3	3	C1IE32_9HEXA | Beta-1 3-D-glucanase EC=3.2.1.6 SubName: Full=Endo-beta-1 3-glucanase EC=3.2.1.39 Flags: Precursor
34	Der p 1.0107	2	2	Der p 1.0107
34	cds.comp136643_c0_seq1|m.214661	2	2	E2C2J9_HARSA | Pancreatic triacylglycerol lipase
34	cds.comp110406_c0_seq1|m.187904	2	2	E2A0U6_CAMFO | N-acetylmuramoyl-L-alanine amidase
34	cds.comp11139_c0_seq1|m.19850	2	2	B7P512_IXOSC | Acetylcholinesterase putative EC=3.1.1.7
34	cds.comp86983_c0_seq1|m.155817	2	2	no hit
34	cds.comp118488_c0_seq1|m.198747	2	2	L7M225_9ACAR | Putative lysosomal pro-x carboxypeptidase-like protein
34	cds.comp92988_c0_seq1|m.165215	2	2	R4V1A4_COPFO | Enolase
34	cds.comp70328_c0_seq1|m.128999	2	2	L7ME31_9ACAR | Putative laminin a Flags: Fragment
34	cds.comp138947_c0_seq1|m.215905	2	2	E2BBG1_HARSA | Filamin-C
35	cds.comp58725_c0_seq1|m.109492	21	21	E9IA80_SOLIN | Fructose-bisphosphate aldolase EC=4.1.2.13 Flags: Fragment
35	cds.comp120606_c0_seq1|m.201109	8	8	Q17G61_AEDAE | AAEL003193-PA
35	cds.comp86168_c0_seq1|m.154662	7	0	R4FNL2_RHOPR | Putative cathepsin b-like proteinase
35	cds.comp86166_c0_seq1|m.154654	7	1	R4FNL2_RHOPR | Putative cathepsin b-like proteinase
35	cds.comp16912_c0_seq1|m.31604	7	7	D3TS39_GLOMM | Adenosine kinase
35	cds.comp113914_c0_seq1|m.192570	6	6	B7PKP9_IXOSC | Glyceraldehyde-3-phosphate dehydrogenase EC=1.2.1.12
35	cds.comp72133_c0_seq1|m.132053	6	6	DERP3_DERPT | Mite allergen Der p 3 EC=3.4.21.- AltName: Full=Allergen Der p III AltName: Allergen=Der p 3 Flags: Precursor
35	Der p 2.0106	6	6	Der p 2.0106

Spot number	Accession	#Peptides	#Unique	Description
35	cds.comp86986_c0_seq1|m.155830	6	6	no hit
35	cds.comp11103_c0_seq1|m.19801	5	5	B0X973_CULQU | Lysosomal alpha-mannosidase
35	cds.comp110928_c0_seq1|m.188689	5	4	A1KXH7_DERFA | Der f Alt a 10 allergen
35	cds.comp13484_c0_seq1|m.24563	5	5	E0W157_PEDHC | GDP-L-fucose synthetase putative EC=1.1.1.271
35	cds.comp27085_c0_seq1|m.52277	5	5	no hit
35	Der p 1.0108	4	1	Der p 1.0108
35	Der p 1.0116	4	0	Der p 1.0116
35	cds.comp117252_c0_seq1|m.197148	4	4	ALL2_PSOOV | Mite group 2 allergen Pso o 2 AltName: Full=Allergen Pso o A AltName: Allergen=Pso o 2 Flags: Precursor
35	cds.comp86161_c0_seq1|m.154648	4	3	D6WGZ1_TRICA | Cathepsin B
35	cds.comp11933_c0_seq1|m.21531	4	4	B7PDZ6_IXOSC | Alpha-D-galactosidase putative EC=3.2.1.49 Flags: Fragment
35	cds.comp116086_c0_seq1|m.195553	3	1	PEPT1_DERPT | Peptidase 1 EC=3.4.22.65 AltName: Full=Allergen Der p I AltName: Full=Major mite fecal allergen Der p 1 AltName: Allergen=Der p 1 Flags: Precursor
35	cds.comp8012_c0_seq1|m.14281	3	2	PEPT1_EURMA | Peptidase 1 EC=3.4.22.65 AltName: Full=Allergen Eur m I AltName: Full=Mite group 1 allergen Eur m 1 AltName: Allergen=Eur m 1 Flags: Precursor
35	Der p 6.0101	3	3	Der p 6.0101
35	cds.comp122816_c0_seq1|m.203611	3	3	C1IE32_9HEXA | Beta-1 3-D-glucanase EC=3.2.1.6 SubName: Full=Endo-beta-1 3-glucanase EC=3.2.1.39 Flags: Precursor
35	cds.comp68825_c0_seq1|m.126306	3	3	B4J0B5_DROGR | GH15860
35	cds.comp44313_c0_seq1|m.83628	3	3	no hit
35	cds.comp71098_c0_seq1|m.130257	3	3	B4YTT9_9ACAR | Heat shock protein 70-2
35	cds.comp90671_c0_seq1|m.161623	3	3	E9FTN1_DAPPU | Beta-galactosidase EC=3.2.1.23
35	cds.comp11139_c0_seq1|m.19850	3	3	B7P512_IXOSC | Acetylcholinesterase putative EC=3.1.1.7

Spot number	Accession	#Peptides	#Unique	Description
35	cds.comp90809_c0_seq1|m.161776	2	2	L7M2J0_9ACAR | Putative aminopeptidase of the m17 family
35	cds.comp86983_c0_seq1|m.155817	2	2	no hit
35	cds.comp39630_c0_seq1|m.75534	2	2	D0PWZ6_BOMMO | UDP-glucosyltransferase protein 3
35	cds.comp124460_c0_seq1|m.205141	2	2	A1YW13_DERFA | Der f 1 allergen
35	cds.comp8010_c0_seq1|m.14279	2	1	PEPT1_EURMA | Peptidase 1 EC=3.4.22.65 AltName: Full=Allergen Eur m I AltName: Full=Mite group 1 allergen Eur m 1 AltName: Allergen=Eur m 1 Flags: Precursor
35	cds.comp107939_c0_seq1|m.184964	2	1	A1KXI6_BLOTA | Blo t aldehyde dehydrogenase allergen
36	cds.comp58728_c0_seq1|m.109496	26	26	G9C5D5_SCHGR | Fructose-bisphosphate aldolase EC=4.1.2.13
36	cds.comp113914_c0_seq1|m.192570	14	14	B7PKP9_IXOSC | Glyceraldehyde-3-phosphate dehydrogenase EC=1.2.1.12
36	cds.comp123134_c0_seq1|m.203950	9	1	L7M5B4_9ACAR | Putative 3-hydroxyacyl-coa dehydrogenase
36	cds.comp114923_c0_seq1|m.194060	6	6	Q9Y197_DERPT | Alpha-amylase Flags: Fragment
36	cds.comp86986_c0_seq1|m.155830	5	5	no hit
36	cds.comp72133_c0_seq1|m.132053	4	4	DERP3_DERPT | Mite allergen Der p 3 EC=3.4.21.- AltName: Full=Allergen Der p III AltName: Allergen=Der p 3 Flags: Precursor
36	cds.comp129401_c0_seq1|m.209549	3	3	B7PAQ0_IXOSC | Gamma-glutamyltransferase putative EC=2.3.2.2
36	cds.comp86983_c0_seq1|m.155817	2	2	no hit
36	cds.comp105480_c0_seq1|m.181427	2	2	L7M5T1_9ACAR | Putative actin regulatory protein
36	cds.comp60483_c0_seq1|m.112772	2	2	L7MIP8_9ACAR | Putative beta-lactamase Flags: Fragment
37	cds.comp72134_c0_seq1|m.132054	10	2	DERP3_DERPT | Mite allergen Der p 3 EC=3.4.21.- AltName: Full=Allergen Der p III AltName: Allergen=Der p 3 Flags: Precursor
37	cds.comp54535_c0_seq1|m.102040	9	9	A1KXH8_DERFA | Der f Gal d 2 allergen
37	cds.comp72130_c0_seq1|m.132048	9	1	DERP3_DERPT | Mite allergen Der p 3 EC=3.4.21.- AltName: Full=Allergen Der p III AltName: Allergen=Der p 3 Flags: Precursor

Spot number	Accession	#Peptides	#Unique	Description
37	cds.comp70346_c0_seq1|m.129037	9	9	B7PYU7_IXOSC | Calcium-binding protein putative Flags: Fragment
37	cds.comp141003_c0_seq1|m.216893	8	8	I4DIJ5_PAPXU | Similar to CG3108
37	cds.comp86168_c0_seq1|m.154662	7	6	R4FNL2_RHOPR | Putative cathepsin b-like proteinase
37	cds.comp86986_c0_seq1|m.155830	7	7	no hit
37	cds.comp11050_c0_seq1|m.19698	6	6	L7MGQ3_9ACAR | Putative maltase glucoamylase Flags: Fragment
37	cds.comp70828_c0_seq1|m.129947	5	5	Q6PPI6_HOMVI | Putative cytoplasmic actin A3a1
37	cds.comp70792_c0_seq1|m.129869	5	5	B2MVM3_SARSC | Actin
37	Der p 6.0101	5	5	Der p 6.0101
37	cds.comp70403_c0_seq1|m.129123	5	5	A1KXH8_DERFA | Der f Gal d 2 allergen
37	cds.comp105855_c0_seq1|m.181988	4	4	A1IHK5_HAELO | Serine carboxypeptidase
37	cds.comp86161_c0_seq1|m.154648	4	3	D6WGZ1_TRICA | Cathepsin B
37	cds.comp136643_c0_seq1|m.214661	4	4	E2C2J9_HARSA | Pancreatic triacylglycerol lipase
37	Der p 1.0107	3	3	Der p 1.0107
37	cds.comp8012_c0_seq1|m.14281	3	2	PEPT1_EURMA | Peptidase 1 EC=3.4.22.65 AltName: Full=Allergen Eur m I AltName: Full=Mite group 1 allergen Eur m 1 AltName: Allergen=Eur m 1 Flags: Precursor
37	cds.comp97466_c0_seq1|m.171059	3	3	no hit
37	cds.comp86983_c0_seq1|m.155817	3	3	no hit
37	cds.comp122816_c0_seq1|m.203611	3	3	C1IE32_9HEXA | Beta-1 3-D-glucanase EC=3.2.1.6 SubName: Full=Endo-beta-1 3-glucanase EC=3.2.1.39 Flags: Precursor
37	cds.comp54254_c0_seq1|m.101590	2	2	no hit
37	cds.comp47520_c0_seq1|m.89357	2	2	A9QW25_CARMA | Glycosyl-phosphatidylinositol-linked carbonic anhydrase EC=4.2.1.1

Spot number	Accession	#Peptides	#Unique	Description
37	cds.comp110399_c0_seq1|m.187884	2	2	E2A0U6_CAMFO | N-acetylmuramoyl-L-alanine amidase
37	cds.comp133360_c0_seq1|m.212547	2	2	no hit
37	cds.comp94280_c0_seq1|m.166903	2	2	ALL2_DERPT | Mite group 2 allergen Der p 2 AltName: Full=Allergen Der p II AltName: Full=DPX AltName: Allergen=Der p 2 Flags: Precursor
38	cds.comp70346_c0_seq1|m.129037	14	14	B7PYU7_IXOSC | Calcium-binding protein putative Flags: Fragment
38	cds.comp72134_c0_seq1|m.132054	12	2	DERP3_DERPT | Mite allergen Der p 3 EC=3.4.21.- AltName: Full=Allergen Der p III AltName: Allergen=Der p 3 Flags: Precursor
38	cds.comp72130_c0_seq1|m.132048	11	1	DERP3_DERPT | Mite allergen Der p 3 EC=3.4.21.- AltName: Full=Allergen Der p III AltName: Allergen=Der p 3 Flags: Precursor
38	cds.comp11050_c0_seq1|m.19698	9	9	L7MGQ3_9ACAR | Putative maltase glucoamylase Flags: Fragment
38	cds.comp70828_c0_seq1|m.129947	8	7	Q6PPI6_HOMVI | Putative cytoplasmic actin A3a1
38	cds.comp86168_c0_seq1|m.154662	8	7	R4FNL2_RHOPR | Putative cathepsin b-like proteinase
38	cds.comp54535_c0_seq1|m.102040	8	8	A1KXH8_DERFA | Der f Gal d 2 allergen
38	cds.comp141003_c0_seq1|m.216893	8	1	I4DIJ5_PAPXU | Similar to CG3108
38	cds.comp86986_c0_seq1|m.155830	8	8	no hit
38	cds.comp8012_c0_seq1|m.14281	7	2	PEPT1_EURMA | Peptidase 1 EC=3.4.22.65 AltName: Full=Allergen Eur m I AltName: Full=Mite group 1 allergen Eur m 1 AltName: Allergen=Eur m 1 Flags: Precursor
38	cds.comp141002_c0_seq1|m.216892	7	0	F4X0L8_ACREC | Carboxypeptidase B
38	Der p 6.0101	7	7	Der p 6.0101
38	cds.comp70758_c0_seq1|m.129814	6	5	B2MVM3_SARSC | Actin
38	cds.comp8071_c0_seq1|m.14356	5	3	PEPT1_EURMA | Peptidase 1 EC=3.4.22.65 AltName: Full=Allergen Eur m I AltName: Full=Mite group 1 allergen Eur m 1 AltName: Allergen=Eur m 1 Flags: Precursor
38	Der p 1.0115	4	4	Der p 1.0115
38	cds.comp8057_c0_seq1|m.14337	4	0	PEPT1_EURMA | Peptidase 1 EC=3.4.22.65 AltName: Full=Allergen Eur m I AltName: Full=Mite group 1 allergen Eur m 1 AltName: Allergen=Eur m 1 Flags: Precursor

Spot number	Accession	#Peptides	#Unique	Description
38	cds.comp86162_c0_seq1|m.154650	4	3	D6WGZ1_TRICA | Cathepsin B
38	cds.comp105855_c0_seq1|m.181988	4	4	A1IHK5_HAELO | Serine carboxypeptidase
38	cds.comp122816_c0_seq1|m.203611	4	4	C1IE32_9HEXA | Beta-1 3-D-glucanase EC=3.2.1.6 SubName: Full=Endo-beta-1 3-glucanase EC=3.2.1.39 Flags: Precursor
38	cds.comp8010_c0_seq1|m.14279	4	2	PEPT1_EURMA | Peptidase 1 EC=3.4.22.65 AltName: Full=Allergen Eur m I AltName: Full=Mite group 1 allergen Eur m 1 AltName: Allergen=Eur m 1 Flags: Precursor
38	cds.comp70396_c0_seq1|m.129112	3	3	A1KXH8_DERFA | Der f Gal d 2 allergen
38	cds.comp47520_c0_seq1|m.89357	3	3	A9QW25_CARMA | Glycosyl-phosphatidylinositol-linked carbonic anhydrase EC=4.2.1.1
38	cds.comp57248_c0_seq1|m.106667	3	3	Q4JK70_DERPT | Group 15 allergen protein
38	cds.comp11138_c0_seq1|m.19849	3	3	B3TFG6_9ACAR | Esterase TCE1
38	cds.comp8013_c0_seq1|m.14282	3	0	PEPT1_EURMA | Peptidase 1 EC=3.4.22.65 AltName: Full=Allergen Eur m I AltName: Full=Mite group 1 allergen Eur m 1 AltName: Allergen=Eur m 1 Flags: Precursor
38	cds.comp97466_c0_seq1|m.171059	2	2	no hit
38	cds.comp86983_c0_seq1|m.155817	2	2	no hit
38	cds.comp6189_c0_seq1|m.10276	2	2	B7PCE6_IXOSC | Ionotropic glutamate receptor putative
38	cds.comp133360_c0_seq1|m.212547	2	2	no hit
38	cds.comp42243_c0_seq1|m.80013	2	2	L7LYB7_9ACAR | Putative conserved secreted protein
38	cds.comp124460_c0_seq1|m.205141	2	2	A1YW13_DERFA | Der f 1 allergen
38	cds.comp92986_c0_seq1|m.165213	2	2	R4V1A4_COPFO | Enolase
38	cds.comp41866_c0_seq1|m.79355	2	2	no hit
38	cds.comp97134_c0_seq1|m.170573	2	2	B7PB29_IXOSC | Excitatory amino acid transporter putative
39	cds.comp86986_c0_seq1|m.155830	8	8	no hit

Spot number	Accession	#Peptides	#Unique	Description
39	cds.comp8012_c0_seq1|m.14281	7	2	PEPT1_EURMA | Peptidase 1 EC=3.4.22.65 AltName: Full=Allergen Eur m I AltName: Full=Mite group 1 allergen Eur m 1 AltName: Allergen=Eur m 1 Flags: Precursor
39	Der p 6.0101	7	7	Der p 6.0101
39	cds.comp11050_c0_seq1|m.19698	7	7	L7MGQ3_9ACAR | Putative maltase glucoamylase Flags: Fragment
39	cds.comp97466_c0_seq1|m.171059	6	6	no hit
39	cds.comp86161_c0_seq1|m.154648	4	3	D6WGZ1_TRICA | Cathepsin B
39	cds.comp8057_c0_seq1|m.14337	4	0	PEPT1_EURMA | Peptidase 1 EC=3.4.22.65 AltName: Full=Allergen Eur m I AltName: Full=Mite group 1 allergen Eur m 1 AltName: Allergen=Eur m 1 Flags: Precursor
39	cds.comp105855_c0_seq1|m.181988	4	4	A1IHK5_HAELO | Serine carboxypeptidase
39	cds.comp47520_c0_seq1|m.89357	4	4	A9QW25_CARMA | Glycosyl-phosphatidylinositol-linked carbonic anhydrase EC=4.2.1.1
39	cds.comp122816_c0_seq1|m.203611	4	4	C1IE32_9HEXA | Beta-1 3-D-glucanase EC=3.2.1.6 SubName: Full=Endo-beta-1 3-glucanase EC=3.2.1.39 Flags: Precursor
39	cds.comp8010_c0_seq1|m.14279	4	0	PEPT1_EURMA | Peptidase 1 EC=3.4.22.65 AltName: Full=Allergen Eur m I AltName: Full=Mite group 1 allergen Eur m 1 AltName: Allergen=Eur m 1 Flags: Precursor
39	cds.comp86983_c0_seq1|m.155817	3	3	no hit
39	cds.comp120606_c0_seq1|m.201109	3	3	Q17G61_AEDAE | AAEL003193-PA
39	cds.comp136643_c0_seq1|m.214661	3	3	E2C2J9_HARSA | Pancreatic triacylglycerol lipase
39	cds.comp11138_c0_seq1|m.19849	3	3	B3TFG6_9ACAR | Esterase TCE1
39	cds.comp57248_c0_seq1|m.106667	3	3	Q4JK70_DERPT | Group 15 allergen protein
39	cds.comp8013_c0_seq1|m.14282	3	0	PEPT1_EURMA | Peptidase 1 EC=3.4.22.65 AltName: Full=Allergen Eur m I AltName: Full=Mite group 1 allergen Eur m 1 AltName: Allergen=Eur m 1 Flags: Precursor
39	cds.comp27085_c0_seq1|m.52277	3	3	no hit
39	cds.comp117252_c0_seq1|m.197148	2	2	ALL2_PSOOV | Mite group 2 allergen Pso o 2 AltName: Full=Allergen Pso o A AltName: Allergen=Pso o 2 Flags: Precursor
39	cds.comp70398_c0_seq1|m.129114	2	2	A1KXH8_DERFA | Der f Gal d 2 allergen

Spot number	Accession	#Peptides	#Unique	Description
39	cds.comp110399_c0_seq1|m.187884	2	2	E2A0U6_CAMFO | N-acetylmuramoyl-L-alanine amidase
39	cds.comp133360_c0_seq1|m.212547	2	2	no hit
39	cds.comp42243_c0_seq1|m.80013	2	2	L7LYB7_9ACAR | Putative conserved secreted protein
39	cds.comp8016_c0_seq1|m.14286	2	0	PEPT1_EURMA | Peptidase 1 EC=3.4.22.65 AltName: Full=Allergen Eur m I AltName: Full=Mite group 1 allergen Eur m 1 AltName: Allergen=Eur m 1 Flags: Precursor
39	cds.comp124461_c0_seq1|m.205142	2	2	A1YW13_DERFA | Der f 1 allergen
39	cds.comp94280_c0_seq1|m.166903	2	2	ALL2_DERPT | Mite group 2 allergen Der p 2 AltName: Full=Allergen Der p II AltName: Full=DPX AltName: Allergen=Der p 2 Flags: Precursor
40	cds.comp72133_c0_seq1|m.132053	10	10	DERP3_DERPT | Mite allergen Der p 3 EC=3.4.21.- AltName: Full=Allergen Der p III AltName: Allergen=Der p 3 Flags: Precursor
40	cds.comp113914_c0_seq1|m.192570	8	8	B7PKP9_IXOSC | Glyceraldehyde-3-phosphate dehydrogenase EC=1.2.1.12
40	cds.comp122816_c0_seq1|m.203611	8	8	C1IE32_9HEXA | Beta-1 3-D-glucanase EC=3.2.1.6 SubName: Full=Endo-beta-1 3-glucanase EC=3.2.1.39 Flags: Precursor
40	cds.comp105482_c0_seq1|m.181430	7	7	L7M5T1_9ACAR | Putative actin regulatory protein
40	cds.comp67072_c0_seq1|m.123542	6	3	M1KYK4_9HYME | Heat shock cognate 70
40	cds.comp86986_c0_seq1|m.155830	6	6	no hit
40	cds.comp123134_c0_seq1|m.203950	5	1	L7M5B4_9ACAR | Putative 3-hydroxyacyl-coa dehydrogenase
40	Der p 2.0106	5	5	Der p 2.0106
40	cds.comp58725_c0_seq1|m.109492	5	5	E9IA80_SOLIN | Fructose-bisphosphate aldolase EC=4.1.2.13 Flags: Fragment
40	cds.comp68825_c0_seq1|m.126306	5	5	B4J0B5_DROGR | GH15860
40	cds.comp123115_c0_seq1|m.203925	4	0	L7M5B4_9ACAR | Putative 3-hydroxyacyl-coa dehydrogenase
40	cds.comp71060_c0_seq1|m.130212	4	1	B4YTU0_9ACAR | Heat shock protein 70-3
40	cds.comp59793_c0_seq1|m.111642	4	4	Q16M58_AEDAE | AAEL012418-PA

Spot number	Accession	#Peptides	#Unique	Description
40	cds.comp11139_c0_seq1|m.19850	3	3	B7P512_IXOSC | Acetylcholinesterase putative EC=3.1.1.7
40	cds.comp123876_c0_seq1|m.204690	3	3	H9HYH7_ATTCE | 4-hydroxyphenylpyruvate dioxygenase
40	cds.comp86164_c0_seq1|m.154651	3	3	B0W0V3_CULQU | Cathepsin L
40	cds.comp124461_c0_seq1|m.205142	3	3	A1YW13_DERFA | Der f 1 allergen
40	cds.comp72078_c0_seq1|m.131978	3	3	A7UI22_AMBAM | Lospin 7
40	cds.comp11083_c0_seq1|m.19764	3	3	L7MGV3_9ACAR | Putative glycosyl hydrolase family 38 Flags: Fragment
40	cds.comp86983_c0_seq1|m.155817	2	2	no hit
40	cds.comp100884_c0_seq1|m.175767	2	2	E9H9U9_DAPPU | Putative uncharacterized protein
40	cds.comp129401_c0_seq1|m.209549	2	2	B7PAQ0_IXOSC | Gamma-glutamyltransferase putative EC=2.3.2.2
40	cds.comp9912_c0_seq1|m.17778	2	2	Q0KKA6_HAELO | Leucine aminopeptidase
40	cds.comp114923_c0_seq1|m.194060	2	2	Q9Y197_DERPT | Alpha-amylase Flags: Fragment
41	cds.comp58725_c0_seq1|m.109492	20	20	E9IA80_SOLIN | Fructose-bisphosphate aldolase EC=4.1.2.13 Flags: Fragment
41	cds.comp113914_c0_seq1|m.192570	13	13	B7PKP9_IXOSC | Glyceraldehyde-3-phosphate dehydrogenase EC=1.2.1.12
41	cds.comp123134_c0_seq1|m.203950	6	1	L7M5B4_9ACAR | Putative 3-hydroxyacyl-coa dehydrogenase
41	cds.comp123115_c0_seq1|m.203925	5	0	L7M5B4_9ACAR | Putative 3-hydroxyacyl-coa dehydrogenase
41	cds.comp122816_c0_seq1|m.203611	5	5	C1IE32_9HEXA | Beta-1 3-D-glucanase EC=3.2.1.6 SubName: Full=Endo-beta-1 3-glucanase EC=3.2.1.39 Flags: Precursor
41	Der p 2.0113	4	4	Der p 2.0113
41	cds.comp59793_c0_seq1|m.111642	4	4	Q16M58_AEDAE | AAEL012418-PA
41	cds.comp72133_c0_seq1|m.132053	3	3	DERP3_DERPT | Mite allergen Der p 3 EC=3.4.21.- AltName: Full=Allergen Der p III AltName: Allergen=Der p 3 Flags: Precursor

Spot number	Accession	#Peptides	#Unique	Description
41	cds.comp86986_c0_seq1|m.155830	3	3	no hit
41	cds.comp105482_c0_seq1|m.181430	3	3	L7M5T1_9ACAR | Putative actin regulatory protein
41	cds.comp83774_c0_seq1|m.150923	3	3	L7MD56_9ACAR | Putative neural cell adhesion molecule l1 Flags: Fragment
41	cds.comp86164_c0_seq1|m.154651	2	2	B0W0V3_CULQU | Cathepsin L
41	cds.comp4546_c0_seq1|m.7079	2	2	R4WCI9_9HEMI | Unkown protein
41	cds.comp17603_c0_seq1|m.32810	2	2	G3MQ23_9ACAR | Transaldolase EC=2.2.1.2
41	cds.comp11007_c0_seq1|m.19626	2	2	F4W8Y5_ACREC | Lysosomal alpha-mannosidase
41	cds.comp129401_c0_seq1|m.209549	2	2	B7PAQ0_IXOSC | Gamma-glutamyltransferase putative EC=2.3.2.2
41	cds.comp86983_c0_seq1|m.155817	2	2	no hit
42	cds.comp113914_c0_seq1|m.192570	6	6	B7PKP9_IXOSC | Glyceraldehyde-3-phosphate dehydrogenase EC=1.2.1.12
42	cds.comp122816_c0_seq1|m.203611	6	6	C1IE32_9HEXA | Beta-1 3-D-glucanase EC=3.2.1.6 SubName: Full=Endo-beta-1 3-glucanase EC=3.2.1.39 Flags: Precursor
42	cds.comp72133_c0_seq1|m.132053	6	6	DERP3_DERPT | Mite allergen Der p 3 EC=3.4.21.- AltName: Full=Allergen Der p III AltName: Allergen=Der p 3 Flags: Precursor
42	cds.comp86986_c0_seq1|m.155830	5	5	no hit
42	cds.comp107939_c0_seq1|m.184964	5	5	A1KXI6_BLOTA | Blo t aldehyde dehydrogenase allergen
42	cds.comp123134_c0_seq1|m.203950	4	1	L7M5B4_9ACAR | Putative 3-hydroxyacyl-coa dehydrogenase
42	cds.comp123115_c0_seq1|m.203925	3	0	L7M5B4_9ACAR | Putative 3-hydroxyacyl-coa dehydrogenase
42	cds.comp86983_c0_seq1|m.155817	3	3	no hit
42	cds.comp59793_c0_seq1|m.111642	2	2	Q16M58_AEDAE | AAEL012418-PA
42	cds.comp58725_c0_seq1|m.109492	2	2	E9IA80_SOLIN | Fructose-bisphosphate aldolase EC=4.1.2.13 Flags: Fragment

Spot number	Accession	#Peptides	#Unique	Description
42	cds.comp105480_c0_seq1|m.181427	2	2	L7M5T1_9ACAR | Putative actin regulatory protein
43	cds.comp113914_c0_seq1|m.192570	11	11	B7PKP9_IXOSC | Glyceraldehyde-3-phosphate dehydrogenase EC=1.2.1.12
43	cds.comp123134_c0_seq1|m.203950	11	1	L7M5B4_9ACAR | Putative 3-hydroxyacyl-coa dehydrogenase
43	cds.comp123115_c0_seq1|m.203925	11	1	L7M5B4_9ACAR | Putative 3-hydroxyacyl-coa dehydrogenase
43	cds.comp72133_c0_seq1|m.132053	9	9	DERP3_DERPT | Mite allergen Der p 3 EC=3.4.21.- AltName: Full=Allergen Der p III AltName: Allergen=Der p 3 Flags: Precursor
43	cds.comp59793_c0_seq1|m.111642	8	8	Q16M58_AEDAE | AAEL012418-PA
43	cds.comp58725_c0_seq1|m.109492	7	7	E9IA80_SOLIN | Fructose-bisphosphate aldolase EC=4.1.2.13 Flags: Fragment
43	cds.comp107939_c0_seq1|m.184964	5	5	A1KXI6_BLOTA | Blo t aldehyde dehydrogenase allergen
43	cds.comp86986_c0_seq1|m.155830	5	5	no hit
43	cds.comp11083_c0_seq1|m.19764	4	4	L7MGV3_9ACAR | Putative glycosyl hydrolase family 38 Flags: Fragment
43	cds.comp123876_c0_seq1|m.204690	4	4	H9HYH7_ATTCE | 4-hydroxyphenylpyruvate dioxygenase
43	cds.comp72078_c0_seq1|m.131978	3	3	A7UI22_AMBAM | Lospin 7
43	cds.comp114923_c0_seq1|m.194060	3	3	Q9Y197_DERPT | Alpha-amylase Flags: Fragment
43	cds.comp86983_c0_seq1|m.155817	2	2	no hit
43	cds.comp4303_c0_seq1|m.6631	2	2	E2A599_CAMFO | Lysosomal alpha-glucosidase
43	cds.comp94280_c0_seq1|m.166903	2	2	ALL2_DERPT | Mite group 2 allergen Der p 2 AltName: Full=Allergen Der p II AltName: Full=DPX AltName: Allergen=Der p 2 Flags: Precursor
43	Der p 9.0102	2	2	Q8MWR4_DERPT Serine protease LM-1 (Fragment) OS=Dermatophagoides pteronyssinus PE=2 SV=2
43	cds.comp122816_c0_seq1|m.203611	2	2	C1IE32_9HEXA | Beta-1 3-D-glucanase EC=3.2.1.6 SubName: Full=Endo-beta-1 3-glucanase EC=3.2.1.39 Flags: Precursor
43	cds.comp53540_c0_seq1|m.100381	2	2	G3MH40_9ACAR | Malate dehydrogenase EC=1.1.1.37 Flags: Fragment

Spot number	Accession	#Peptides	#Unique	Description
43	cds.comp129401_c0_seq1|m.209549	2	2	B7PAQ0_IXOSC | Gamma-glutamyltransferase putative EC=2.3.2.2
43	cds.comp124461_c0_seq1|m.205142	2	2	A1YW13_DERFA | Der f 1 allergen
44	cds.comp113914_c0_seq1|m.192570	10	10	B7PKP9_IXOSC | Glyceraldehyde-3-phosphate dehydrogenase EC=1.2.1.12
44	cds.comp72133_c0_seq1|m.132053	7	7	DERP3_DERPT | Mite allergen Der p 3 EC=3.4.21.- AltName: Full=Allergen Der p III AltName: Allergen=Der p 3 Flags: Precursor
44	cds.comp123134_c0_seq1|m.203950	6	1	L7M5B4_9ACAR | Putative 3-hydroxyacyl-coa dehydrogenase
44	cds.comp86986_c0_seq1|m.155830	6	6	no hit
44	cds.comp71061_c0_seq1|m.130213	6	3	B4YTU0_9ACAR | Heat shock protein 70-3
44	cds.comp67072_c0_seq1|m.123542	6	3	M1KYK4_9HYME | Heat shock cognate 70
44	cds.comp59793_c0_seq1|m.111642	5	5	Q16M58_AEDAE | AAEL012418-PA
44	cds.comp123115_c0_seq1|m.203925	5	0	L7M5B4_9ACAR | Putative 3-hydroxyacyl-coa dehydrogenase
44	cds.comp123876_c0_seq1|m.204690	5	5	H9HYH7_ATTCE | 4-hydroxyphenylpyruvate dioxygenase
44	cds.comp114923_c0_seq1|m.194060	5	5	Q9Y197_DERPT | Alpha-amylase Flags: Fragment
44	Der p 2.0113	4	4	Der p 2.0113
44	cds.comp105480_c0_seq1|m.181427	3	3	L7M5T1_9ACAR | Putative actin regulatory protein
44	cds.comp11144_c0_seq1|m.19855	2	2	B7P512_IXOSC | Acetylcholinesterase putative EC=3.1.1.7
44	cds.comp11083_c0_seq1|m.19764	2	2	L7MGV3_9ACAR | Putative glycosyl hydrolase family 38 Flags: Fragment
44	cds.comp86983_c0_seq1|m.155817	2	2	no hit
44	cds.comp53540_c0_seq1|m.100381	2	2	G3MH40_9ACAR | Malate dehydrogenase EC=1.1.1.37 Flags: Fragment
45	cds.comp113914_c0_seq1|m.192570	14	14	B7PKP9_IXOSC | Glyceraldehyde-3-phosphate dehydrogenase EC=1.2.1.12

Spot number	Accession	#Peptides	#Unique	Description
45	cds.comp123134_c0_seq1|m.203950	12	1	L7M5B4_9ACAR | Putative 3-hydroxyacyl-coa dehydrogenase
45	cds.comp123115_c0_seq1|m.203925	12	1	L7M5B4_9ACAR | Putative 3-hydroxyacyl-coa dehydrogenase
45	cds.comp59793_c0_seq1|m.111642	9	9	Q16M58_AEDAE | AAEL012418-PA
45	cds.comp59796_c0_seq1|m.111647	9	9	Q16M58_AEDAE | AAEL012418-PA
45	Der p 2.0112	8	1	Der p 2.0112
45	Der p 2.0109	8	1	Der p 2.0109
45	cds.comp72133_c0_seq1|m.132053	7	7	DERP3_DERPT | Mite allergen Der p 3 EC=3.4.21.- AltName: Full=Allergen Der p III AltName: Allergen=Der p 3 Flags: Precursor
45	cds.comp86986_c0_seq1|m.155830	6	6	no hit
45	cds.comp58725_c0_seq1|m.109492	4	4	E9IA80_SOLIN | Fructose-bisphosphate aldolase EC=4.1.2.13 Flags: Fragment
45	cds.comp105480_c0_seq1|m.181427	4	4	L7M5T1_9ACAR | Putative actin regulatory protein
45	cds.comp61160_c0_seq1|m.113810	4	4	L7M520_9ACAR | Malic enzyme
45	cds.comp136840_c0_seq1|m.214779	4	4	L7UZA7_DERFA | Triosephosphate isomerase EC=5.3.1.1
45	cds.comp114923_c0_seq1|m.194060	4	4	Q9Y197_DERPT | Alpha-amylase Flags: Fragment
45	cds.comp11083_c0_seq1|m.19764	3	3	L7MGV3_9ACAR | Putative glycosyl hydrolase family 38 Flags: Fragment
45	cds.comp86983_c0_seq1|m.155817	2	2	no hit
45	cds.comp129401_c0_seq1|m.209549	2	2	B7PAQ0_IXOSC | Gamma-glutamyltransferase putative EC=2.3.2.2
45	Der p 9.0102	2	2	Q8MWR4_DERPT Serine protease LM-1 (Fragment) OS=Dermatophagoides pteronyssinus PE=2 SV=2
45	cds.comp4303_c0_seq1|m.6631	2	2	E2A599_CAMFO | Lysosomal alpha-glucosidase
46	cds.comp113914_c0_seq1|m.192570	14	14	B7PKP9_IXOSC | Glyceraldehyde-3-phosphate dehydrogenase EC=1.2.1.12

Spot number	Accession	#Peptides	#Unique	Description
46	cds.comp72134_c0_seq1|m.132054	14	14	DERP3_DERPT | Mite allergen Der p 3 EC=3.4.21.- AltName: Full=Allergen Der p III AltName: Allergen=Der p 3 Flags: Precursor
46	cds.comp105482_c0_seq1|m.181430	9	9	L7M5T1_9ACAR | Putative actin regulatory protein
46	cds.comp59793_c0_seq1|m.111642	7	7	Q16M58_AEDAE | AAEL012418-PA
46	cds.comp107939_c0_seq1|m.184964	7	7	A1KXI6_BLOTA | Blo t aldehyde dehydrogenase allergen
46	cds.comp123134_c0_seq1|m.203950	7	1	L7M5B4_9ACAR | Putative 3-hydroxyacyl-coa dehydrogenase
46	cds.comp58725_c0_seq1|m.109492	7	7	E9IA80_SOLIN | Fructose-bisphosphate aldolase EC=4.1.2.13 Flags: Fragment
46	cds.comp123115_c0_seq1|m.203925	6	0	L7M5B4_9ACAR | Putative 3-hydroxyacyl-coa dehydrogenase
46	cds.comp86986_c0_seq1|m.155830	6	6	no hit
46	cds.comp114923_c0_seq1|m.194060	5	5	Q9Y197_DERPT | Alpha-amylase Flags: Fragment
46	cds.comp11083_c0_seq1|m.19764	3	3	L7MGV3_9ACAR | Putative glycosyl hydrolase family 38 Flags: Fragment
46	cds.comp86983_c0_seq1|m.155817	3	3	no hit
46	cds.comp9912_c0_seq1|m.17778	3	3	Q0KKA6_HAELO | Leucine aminopeptidase
46	Der p 9.0102	2	2	Q8MWR4_DERPT Serine protease LM-1 (Fragment) OS=Dermatophagoides pteronyssinus PE=2 SV=2
46	cds.comp4303_c0_seq1|m.6631	2	2	E2A599_CAMFO | Lysosomal alpha-glucosidase
46	cds.comp32740_c0_seq1|m.62997	2	2	G1UIC8_9ARAC | Thioester-containing protein
46	cds.comp57928_c0_seq1|m.107765	2	2	Q7PF06_ANOGA | AGAP011050-PA
47	cds.comp113914_c0_seq1|m.192570	18	18	B7PKP9_IXOSC | Glyceraldehyde-3-phosphate dehydrogenase EC=1.2.1.12
47	cds.comp72134_c0_seq1|m.132054	13	13	DERP3_DERPT | Mite allergen Der p 3 EC=3.4.21.- AltName: Full=Allergen Der p III AltName: Allergen=Der p 3 Flags: Precursor
47	cds.comp123134_c0_seq1|m.203950	11	1	L7M5B4_9ACAR | Putative 3-hydroxyacyl-coa dehydrogenase

Spot number	Accession	#Peptides	#Unique	Description
47	cds.comp123115_c0_seq1|m.203925	11	1	L7M5B4_9ACAR | Putative 3-hydroxyacyl-coa dehydrogenase
47	cds.comp59793_c0_seq1|m.111642	8	8	Q16M58_AEDAE | AAEL012418-PA
47	cds.comp86986_c0_seq1|m.155830	7	7	no hit
47	cds.comp114923_c0_seq1|m.194060	6	6	Q9Y197_DERPT | Alpha-amylase Flags: Fragment
47	cds.comp11083_c0_seq1|m.19764	5	5	L7MGV3_9ACAR | Putative glycosyl hydrolase family 38 Flags: Fragment
47	cds.comp134821_c0_seq1|m.213456	4	4	Q7Z163_DERPT | Trypsin-like serine protease
47	cds.comp86983_c0_seq1|m.155817	3	3	no hit
47	cds.comp105480_c0_seq1|m.181427	3	3	L7M5T1_9ACAR | Putative actin regulatory protein
47	cds.comp129401_c0_seq1|m.209549	3	3	B7PAQ0_IXOSC | Gamma-glutamyltransferase putative EC=2.3.2.2
47	cds.comp11144_c0_seq1|m.19855	3	3	B7P512_IXOSC | Acetylcholinesterase putative EC=3.1.1.7
47	cds.comp4303_c0_seq1|m.6631	2	2	E2A599_CAMFO | Lysosomal alpha-glucosidase
47	cds.comp61160_c0_seq1|m.113810	2	2	L7M520_9ACAR | Malic enzyme
47	cds.comp90671_c0_seq1|m.161623	2	2	E9FTN1_DAPPU | Beta-galactosidase EC=3.2.1.23
48	cds.comp54535_c0_seq1|m.102040	14	14	A1KXH8_DERFA | Der f Gal d 2 allergen
48	cds.comp70403_c0_seq1|m.129123	14	14	A1KXH8_DERFA | Der f Gal d 2 allergen
48	cds.comp57248_c0_seq1|m.106667	12	12	Q4JK70_DERPT | Group 15 allergen protein
48	cds.comp8012_c0_seq1|m.14281	10	2	PEPT1_EURMA | Peptidase 1 EC=3.4.22.65 AltName: Full=Allergen Eur m I AltName: Full=Mite group 1 allergen Eur m 1 AltName: Allergen=Eur m 1 Flags: Precursor
48	cds.comp72133_c0_seq1|m.132053	9	9	DERP3_DERPT | Mite allergen Der p 3 EC=3.4.21.- AltName: Full=Allergen Der p III AltName: Allergen=Der p 3 Flags: Precursor
48	Der p 6.0101	8	8	Der p 6.0101

Spot number	Accession	#Peptides	#Unique	Description
48	cds.comp113914_c0_seq1|m.192571	8	8	B3NZN4_DROER | GG16982
48	cds.comp86168_c0_seq1|m.154662	7	6	R4FNL2_RHOPR | Putative cathepsin b-like proteinase
48	cds.comp8057_c0_seq1|m.14337	7	0	PEPT1_EURMA | Peptidase 1 EC=3.4.22.65 AltName: Full=Allergen Eur m I AltName: Full=Mite group 1 allergen Eur m 1 AltName: Allergen=Eur m 1 Flags: Precursor
48	cds.comp8071_c0_seq1|m.14356	6	3	PEPT1_EURMA | Peptidase 1 EC=3.4.22.65 AltName: Full=Allergen Eur m I AltName: Full=Mite group 1 allergen Eur m 1 AltName: Allergen=Eur m 1 Flags: Precursor
48	cds.comp136643_c0_seq1|m.214661	6	6	E2C2J9_HARSA | Pancreatic triacylglycerol lipase
48	cds.comp8014_c0_seq1|m.14284	6	1	PEPT1_EURMA | Peptidase 1 EC=3.4.22.65 AltName: Full=Allergen Eur m I AltName: Full=Mite group 1 allergen Eur m 1 AltName: Allergen=Eur m 1 Flags: Precursor
48	cds.comp8010_c0_seq1|m.14279	6	2	PEPT1_EURMA | Peptidase 1 EC=3.4.22.65 AltName: Full=Allergen Eur m I AltName: Full=Mite group 1 allergen Eur m 1 AltName: Allergen=Eur m 1 Flags: Precursor
48	Der p 1.0116	5	0	Der p 1.0116
48	Der p 1.0108	5	1	Der p 1.0108
48	cds.comp86161_c0_seq1|m.154648	5	4	D6WGZ1_TRICA | Cathepsin B
48	cds.comp70339_c0_seq1|m.129030	5	5	B7PYU7_IXOSC | Calcium-binding protein putative Flags: Fragment
48	cds.comp8013_c0_seq1|m.14282	5	0	PEPT1_EURMA | Peptidase 1 EC=3.4.22.65 AltName: Full=Allergen Eur m I AltName: Full=Mite group 1 allergen Eur m 1 AltName: Allergen=Eur m 1 Flags: Precursor
48	cds.comp116086_c0_seq1|m.195553	4	1	PEPT1_DERPT | Peptidase 1 EC=3.4.22.65 AltName: Full=Allergen Der p I AltName: Full=Major mite fecal allergen Der p 1 AltName: Allergen=Der p 1 Flags: Precursor
48	cds.comp105855_c0_seq1|m.181988	4	4	A1IHK5_HAELO | Serine carboxypeptidase
48	cds.comp128133_c0_seq1|m.208437	3	3	C1IE32_9HEXA | Beta-1 3-D-glucanase EC=3.2.1.6 SubName: Full=Endo-beta-1 3-glucanase EC=3.2.1.39 Flags: Precursor
48	cds.comp65229_c0_seq1|m.120785	3	3	B7P7Y4_IXOSC | Enoyl-CoA hydratase putative EC=2.3.1.48 EC=5.3.3.8 Flags: Fragment
48	cds.comp86986_c0_seq1|m.155830	2	2	no hit
48	cds.comp110399_c0_seq1|m.187884	2	2	E2A0U6_CAMFO | N-acetylmuramoyl-L-alanine amidase
48	cds.comp122816_c0_seq1|m.203611	2	2	C1IE32_9HEXA | Beta-1 3-D-glucanase EC=3.2.1.6 SubName: Full=Endo-beta-1 3-glucanase EC=3.2.1.39 Flags: Precursor

Spot number	Accession	#Peptides	#Unique	Description
48	cds.comp70438_c0_seq1|m.129154	2	2	Q9U6M8_RHIMP | Esterase SubName: Full=Pyrethroid-metabolizing carboxylesterase
48	cds.comp8016_c0_seq1|m.14286	2	2	PEPT1_EURMA | Peptidase 1 EC=3.4.22.65 AltName: Full=Allergen Eur m I AltName: Full=Mite group 1 allergen Eur m 1 AltName: Allergen=Eur m 1 Flags: Precursor
48	cds.comp124461_c0_seq1|m.205142	2	2	A1YW13_DERFA | Der f 1 allergen
48	cds.comp70420_c0_seq1|m.129137	2	2	no hit
48	cds.comp27085_c0_seq1|m.52277	2	2	no hit
49	cds.comp70403_c0_seq1|m.129123	11	11	A1KXH8_DERFA | Der f Gal d 2 allergen
49	cds.comp8012_c0_seq1|m.14281	9	2	PEPT1_EURMA | Peptidase 1 EC=3.4.22.65 AltName: Full=Allergen Eur m I AltName: Full=Mite group 1 allergen Eur m 1 AltName: Allergen=Eur m 1 Flags: Precursor
49	cds.comp57248_c0_seq1|m.106667	9	9	Q4JK70_DERPT | Group 15 allergen protein
49	Der p 6.0101	8	8	Der p 6.0101
49	cds.comp72133_c0_seq1|m.132053	8	8	DERP3_DERPT | Mite allergen Der p 3 EC=3.4.21.- AltName: Full=Allergen Der p III AltName: Allergen=Der p 3 Flags: Precursor
49	cds.comp54535_c0_seq1|m.102040	7	7	A1KXH8_DERFA | Der f Gal d 2 allergen
49	cds.comp8057_c0_seq1|m.14337	7	0	PEPT1_EURMA | Peptidase 1 EC=3.4.22.65 AltName: Full=Allergen Eur m I AltName: Full=Mite group 1 allergen Eur m 1 AltName: Allergen=Eur m 1 Flags: Precursor
49	cds.comp8071_c0_seq1|m.14356	6	3	PEPT1_EURMA | Peptidase 1 EC=3.4.22.65 AltName: Full=Allergen Eur m I AltName: Full=Mite group 1 allergen Eur m 1 AltName: Allergen=Eur m 1 Flags: Precursor
49	Der p 1.0116	6	0	Der p 1.0116
49	Der p 1.0106	6	1	Der p 1.0106
49	cds.comp113914_c0_seq1|m.192571	6	6	B3NZN4_DROER | GG16982
49	cds.comp86168_c0_seq1|m.154662	5	4	R4FNL2_RHOPR | Putative cathepsin b-like proteinase
49	cds.comp116086_c0_seq1|m.195553	5	1	PEPT1_DERPT | Peptidase 1 EC=3.4.22.65 AltName: Full=Allergen Der p I AltName: Full=Major mite fecal allergen Der p 1 AltName: Allergen=Der p 1 Flags: Precursor
49	cds.comp86161_c0_seq1|m.154648	5	4	D6WGZ1_TRICA | Cathepsin B

Spot number	Accession	#Peptides	#Unique	Description
49	cds.comp136643_c0_seq1|m.214661	5	5	E2C2J9_HARSA | Pancreatic triacylglycerol lipase
49	cds.comp8014_c0_seq1|m.14284	5	1	PEPT1_EURMA | Peptidase 1 EC=3.4.22.65 AltName: Full=Allergen Eur m I AltName: Full=Mite group 1 allergen Eur m 1 AltName: Allergen=Eur m 1 Flags: Precursor
49	cds.comp8010_c0_seq1|m.14279	5	2	PEPT1_EURMA | Peptidase 1 EC=3.4.22.65 AltName: Full=Allergen Eur m I AltName: Full=Mite group 1 allergen Eur m 1 AltName: Allergen=Eur m 1 Flags: Precursor
49	cds.comp128133_c0_seq1|m.208437	4	4	C1IE32_9HEXA | Beta-1 3-D-glucanase EC=3.2.1.6 SubName: Full=Endo-beta-1 3-glucanase EC=3.2.1.39 Flags: Precursor
49	cds.comp8013_c0_seq1|m.14282	4	0	PEPT1_EURMA | Peptidase 1 EC=3.4.22.65 AltName: Full=Allergen Eur m I AltName: Full=Mite group 1 allergen Eur m 1 AltName: Allergen=Eur m 1 Flags: Precursor
49	cds.comp105855_c0_seq1|m.181988	3	3	A1IHK5_HAELO | Serine carboxypeptidase
49	cds.comp114643_c0_seq1|m.193702	3	3	G3MQD4_9ACAR | Glutamine synthetase EC=6.3.1.2
49	cds.comp113512_c0_seq1|m.191862	2	2	B4PW26_DROYA | GE14560
49	cds.comp122816_c0_seq1|m.203611	2	2	C1IE32_9HEXA | Beta-1 3-D-glucanase EC=3.2.1.6 SubName: Full=Endo-beta-1 3-glucanase EC=3.2.1.39 Flags: Precursor
49	cds.comp70438_c0_seq1|m.129154	2	2	Q9U6M8_RHIMP | Esterase SubName: Full=Pyrethroid-metabolizing carboxylesterase
49	cds.comp27085_c0_seq1|m.52277	2	2	no hit
49	cds.comp137015_c0_seq1|m.214858	2	2	PEPT1_EURMA | Peptidase 1 EC=3.4.22.65 AltName: Full=Allergen Eur m I AltName: Full=Mite group 1 allergen Eur m 1 AltName: Allergen=Eur m 1 Flags: Precursor
49	cds.comp141003_c0_seq1|m.216893	2	2	I4DIJ5_PAPXU | Similar to CG3108
50	Der p 6.0101	8	8	Der p 6.0101
50	cds.comp8012_c0_seq1|m.14281	7	2	PEPT1_EURMA | Peptidase 1 EC=3.4.22.65 AltName: Full=Allergen Eur m I AltName: Full=Mite group 1 allergen Eur m 1 AltName: Allergen=Eur m 1 Flags: Precursor
50	cds.comp86168_c0_seq1|m.154662	6	5	R4FNL2_RHOPR | Putative cathepsin b-like proteinase
50	cds.comp8007_c0_seq1|m.14274	5	2	PEPT1_EURMA | Peptidase 1 EC=3.4.22.65 AltName: Full=Allergen Eur m I AltName: Full=Mite group 1 allergen Eur m 1 AltName: Allergen=Eur m 1 Flags: Precursor
50	cds.comp8057_c0_seq1|m.14337	5	0	PEPT1_EURMA | Peptidase 1 EC=3.4.22.65 AltName: Full=Allergen Eur m I AltName: Full=Mite group 1 allergen Eur m 1 AltName: Allergen=Eur m 1 Flags: Precursor
50	cds.comp72133_c0_seq1|m.132053	5	5	DERP3_DERPT | Mite allergen Der p 3 EC=3.4.21.- AltName: Full=Allergen Der p III AltName: Allergen=Der p 3 Flags: Precursor

Spot number	Accession	#Peptides	#Unique	Description
50	cds.comp8052_c0_seq1|m.14330	4	2	PEPT1_EURMA | Peptidase 1 EC=3.4.22.65 AltName: Full=Allergen Eur m I AltName: Full=Mite group 1 allergen Eur m 1 AltName: Allergen=Eur m 1 Flags: Precursor
50	Der p 1.0108	4	1	Der p 1.0108
50	Der p 1.0107	4	1	Der p 1.0107
50	cds.comp8071_c0_seq1|m.14356	4	2	PEPT1_EURMA | Peptidase 1 EC=3.4.22.65 AltName: Full=Allergen Eur m I AltName: Full=Mite group 1 allergen Eur m 1 AltName: Allergen=Eur m 1 Flags: Precursor
50	cds.comp86161_c0_seq1|m.154648	4	3	D6WGZ1_TRICA | Cathepsin B
50	cds.comp58860_c0_seq1|m.109626	4	4	A0SHR2_AMBVA | Protein disulfide isomerase EC=5.3.4.1
50	Der p 1.0122	3	0	Der p 1.0122
50	cds.comp116082_c0_seq1|m.195545	3	1	PEPT1_DERPT | Peptidase 1 EC=3.4.22.65 AltName: Full=Allergen Der p I AltName: Full=Major mite fecal allergen Der p 1 AltName: Allergen=Der p 1 Flags: Precursor
50	cds.comp8013_c0_seq1|m.14282	3	0	PEPT1_EURMA | Peptidase 1 EC=3.4.22.65 AltName: Full=Allergen Eur m I AltName: Full=Mite group 1 allergen Eur m 1 AltName: Allergen=Eur m 1 Flags: Precursor
50	cds.comp8014_c0_seq1|m.14284	3	0	PEPT1_EURMA | Peptidase 1 EC=3.4.22.65 AltName: Full=Allergen Eur m I AltName: Full=Mite group 1 allergen Eur m 1 AltName: Allergen=Eur m 1 Flags: Precursor
50	cds.comp105855_c0_seq1|m.181988	3	3	A1IHK5_HAELO | Serine carboxypeptidase
50	cds.comp8010_c0_seq1|m.14279	3	1	PEPT1_EURMA | Peptidase 1 EC=3.4.22.65 AltName: Full=Allergen Eur m I AltName: Full=Mite group 1 allergen Eur m 1 AltName: Allergen=Eur m 1 Flags: Precursor
50	cds.comp110406_c0_seq1|m.187904	3	3	E2A0U6_CAMFO | N-acetylmuramoyl-L-alanine amidase
50	cds.comp128133_c0_seq1|m.208437	2	2	C1IE32_9HEXA | Beta-1 3-D-glucanase EC=3.2.1.6 SubName: Full=Endo-beta-1 3-glucanase EC=3.2.1.39 Flags: Precursor
50	cds.comp122816_c0_seq1|m.203611	2	2	C1IE32_9HEXA | Beta-1 3-D-glucanase EC=3.2.1.6 SubName: Full=Endo-beta-1 3-glucanase EC=3.2.1.39 Flags: Precursor
50	Der p 1.0103	2	1	Der p 1.0103
50	cds.comp8015_c0_seq1|m.14285	2	0	PEPT1_EURMA | Peptidase 1 EC=3.4.22.65 AltName: Full=Allergen Eur m I AltName: Full=Mite group 1 allergen Eur m 1 AltName: Allergen=Eur m 1 Flags: Precursor
50	cds.comp102914_c0_seq1|m.178489	2	2	Q8ISH5_ARAVE | Chitinase
50	cds.comp136643_c0_seq1|m.214661	2	2	E2C2J9_HARSA | Pancreatic triacylglycerol lipase

Spot number	Accession	#Peptides	#Unique	Description
50	cds.comp27085_c0_seq1|m.52277	2	2	no hit
51	cds.comp128133_c0_seq1|m.208437	8	8	C1IE32_9HEXA | Beta-1 3-D-glucanase EC=3.2.1.6 SubName: Full=Endo-beta-1 3-glucanase EC=3.2.1.39 Flags: Precursor
51	Der p 6.0101	8	8	Der p 6.0101
51	Der p 1.0106	7	3	Der p 1.0106
51	cds.comp8012_c0_seq1|m.14281	7	2	PEPT1_EURMA | Peptidase 1 EC=3.4.22.65 AltName: Full=Allergen Eur m I AltName: Full=Mite group 1 allergen Eur m 1 AltName: Allergen=Eur m 1 Flags: Precursor
51	cds.comp136643_c0_seq1|m.214661	7	7	E2C2J9_HARSA | Pancreatic triacylglycerol lipase
51	cds.comp116086_c0_seq1|m.195553	5	1	PEPT1_DERPT | Peptidase 1 EC=3.4.22.65 AltName: Full=Allergen Der p I AltName: Full=Major mite fecal allergen Der p 1 AltName: Allergen=Der p 1 Flags: Precursor
51	cds.comp86168_c0_seq1|m.154662	5	4	R4FNL2_RHOPR | Putative cathepsin b-like proteinase
51	cds.comp86161_c0_seq1|m.154648	4	3	D6WGZ1_TRICA | Cathepsin B
51	cds.comp8057_c0_seq1|m.14337	4	0	PEPT1_EURMA | Peptidase 1 EC=3.4.22.65 AltName: Full=Allergen Eur m I AltName: Full=Mite group 1 allergen Eur m 1 AltName: Allergen=Eur m 1 Flags: Precursor
51	cds.comp8014_c0_seq1|m.14284	4	1	PEPT1_EURMA | Peptidase 1 EC=3.4.22.65 AltName: Full=Allergen Eur m I AltName: Full=Mite group 1 allergen Eur m 1 AltName: Allergen=Eur m 1 Flags: Precursor
51	cds.comp8010_c0_seq1|m.14279	4	0	PEPT1_EURMA | Peptidase 1 EC=3.4.22.65 AltName: Full=Allergen Eur m I AltName: Full=Mite group 1 allergen Eur m 1 AltName: Allergen=Eur m 1 Flags: Precursor
51	cds.comp8071_c0_seq1|m.14356	3	1	PEPT1_EURMA | Peptidase 1 EC=3.4.22.65 AltName: Full=Allergen Eur m I AltName: Full=Mite group 1 allergen Eur m 1 AltName: Allergen=Eur m 1 Flags: Precursor
51	cds.comp11138_c0_seq1|m.19849	3	3	B3TFG6_9ACAR | Esterase TCE1
51	cds.comp94769_c0_seq1|m.167474	3	3	C1BS68_9MAXI | 3-oxoacyl-acyl-carrier-protein reductase
51	cds.comp123088_c0_seq1|m.203897	3	3	no hit
51	cds.comp136840_c0_seq1|m.214779	3	3	L7UZA7_DERFA | Triosephosphate isomerase EC=5.3.1.1
51	cds.comp8013_c0_seq1|m.14282	3	0	PEPT1_EURMA | Peptidase 1 EC=3.4.22.65 AltName: Full=Allergen Eur m I AltName: Full=Mite group 1 allergen Eur m 1 AltName: Allergen=Eur m 1 Flags: Precursor
51	cds.comp124461_c0_seq1|m.205142	2	2	A1YW13_DERFA | Der f 1 allergen

Spot number	Accession	#Peptides	#Unique	Description
51	cds.comp86985_c0_seq1|m.155823	2	2	no hit
51	cds.comp8016_c0_seq1|m.14286	2	0	PEPT1_EURMA | Peptidase 1 EC=3.4.22.65 AltName: Full=Allergen Eur m I AltName: Full=Mite group 1 allergen Eur m 1 AltName: Allergen=Eur m 1 Flags: Precursor
52	Der p 6.0101	16	16	Der p 6.0101
52	cds.comp86162_c0_seq1|m.154650	11	9	D6WGZ1_TRICA | Cathepsin B
52	cds.comp86168_c0_seq1|m.154662	7	0	R4FNL2_RHOPR | Putative cathepsin b-like proteinase
52	cds.comp86167_c0_seq1|m.154658	7	2	R4FNL2_RHOPR | Putative cathepsin b-like proteinase
52	Der p 1.0108	6	1	Der p 1.0108
52	Der p 1.0115	6	0	Der p 1.0115
52	cds.comp8012_c0_seq1|m.14281	5	2	PEPT1_EURMA | Peptidase 1 EC=3.4.22.65 AltName: Full=Allergen Eur m I AltName: Full=Mite group 1 allergen Eur m 1 AltName: Allergen=Eur m 1 Flags: Precursor
52	cds.comp128133_c0_seq1|m.208437	5	5	C1IE32_9HEXA | Beta-1 3-D-glucanase EC=3.2.1.6 SubName: Full=Endo-beta-1 3-glucanase EC=3.2.1.39 Flags: Precursor
52	cds.comp116086_c0_seq1|m.195553	4	1	PEPT1_DERPT | Peptidase 1 EC=3.4.22.65 AltName: Full=Allergen Der p I AltName: Full=Major mite fecal allergen Der p 1 AltName: Allergen=Der p 1 Flags: Precursor
52	cds.comp72133_c0_seq1|m.132053	4	4	DERP3_DERPT | Mite allergen Der p 3 EC=3.4.21.- AltName: Full=Allergen Der p III AltName: Allergen=Der p 3 Flags: Precursor
52	cds.comp8071_c0_seq1|m.14356	3	1	PEPT1_EURMA | Peptidase 1 EC=3.4.22.65 AltName: Full=Allergen Eur m I AltName: Full=Mite group 1 allergen Eur m 1 AltName: Allergen=Eur m 1 Flags: Precursor
52	cds.comp102914_c0_seq1|m.178489	3	3	Q8ISH5_ARAVE | Chitinase
52	cds.comp122816_c0_seq1|m.203611	3	3	C1IE32_9HEXA | Beta-1 3-D-glucanase EC=3.2.1.6 SubName: Full=Endo-beta-1 3-glucanase EC=3.2.1.39 Flags: Precursor
52	cds.comp148823_c0_seq1|m.219720	3	3	no hit
52	cds.comp8057_c0_seq1|m.14337	2	0	PEPT1_EURMA | Peptidase 1 EC=3.4.22.65 AltName: Full=Allergen Eur m I AltName: Full=Mite group 1 allergen Eur m 1 AltName: Allergen=Eur m 1 Flags: Precursor
52	cds.comp8010_c0_seq1|m.14279	2	1	PEPT1_EURMA | Peptidase 1 EC=3.4.22.65 AltName: Full=Allergen Eur m I AltName: Full=Mite group 1 allergen Eur m 1 AltName: Allergen=Eur m 1 Flags: Precursor
52	cds.comp8014_c0_seq1|m.14284	2	1	PEPT1_EURMA | Peptidase 1 EC=3.4.22.65 AltName: Full=Allergen Eur m I AltName: Full=Mite group 1 allergen Eur m 1 AltName: Allergen=Eur m 1 Flags: Precursor

Spot number	Accession	#Peptides	#Unique	Description
53	Der p 6.0101	11	11	Der p 6.0101
53	cds.comp86162_c0_seq1|m.154650	10	8	D6WGZ1_TRICA | Cathepsin B
53	cds.comp86168_c0_seq1|m.154662	8	0	R4FNL2_RHOPR | Putative cathepsin b-like proteinase
53	cds.comp8012_c0_seq1|m.14281	7	5	PEPT1_EURMA | Peptidase 1 EC=3.4.22.65 AltName: Full=Allergen Eur m I AltName: Full=Mite group 1 allergen Eur m 1 AltName: Allergen=Eur m 1 Flags: Precursor
53	cds.comp86166_c0_seq1|m.154654	7	1	R4FNL2_RHOPR | Putative cathepsin b-like proteinase
53	cds.comp72133_c0_seq1|m.132053	7	7	DERP3_DERPT | Mite allergen Der p 3 EC=3.4.21.- AltName: Full=Allergen Der p III AltName: Allergen=Der p 3 Flags: Precursor
53	Der p 1.0108	5	1	Der p 1.0108
53	Der p 1.0116	5	0	Der p 1.0116
53	cds.comp8010_c0_seq1|m.14279	5	3	PEPT1_EURMA | Peptidase 1 EC=3.4.22.65 AltName: Full=Allergen Eur m I AltName: Full=Mite group 1 allergen Eur m 1 AltName: Allergen=Eur m 1 Flags: Precursor
53	cds.comp148823_c0_seq1|m.219720	5	5	no hit
53	cds.comp122816_c0_seq1|m.203611	5	5	C1IE32_9HEXA | Beta-1 3-D-glucanase EC=3.2.1.6 SubName: Full=Endo-beta-1 3-glucanase EC=3.2.1.39 Flags: Precursor
53	cds.comp116086_c0_seq1|m.195553	4	1	PEPT1_DERPT | Peptidase 1 EC=3.4.22.65 AltName: Full=Allergen Der p I AltName: Full=Major mite fecal allergen Der p 1 AltName: Allergen=Der p 1 Flags: Precursor
53	cds.comp128133_c0_seq1|m.208437	2	2	C1IE32_9HEXA | Beta-1 3-D-glucanase EC=3.2.1.6 SubName: Full=Endo-beta-1 3-glucanase EC=3.2.1.39 Flags: Precursor
53	cds.comp102914_c0_seq1|m.178489	2	2	Q8ISH5_ARAVE | Chitinase
53	cds.comp72078_c0_seq1|m.131978	2	2	A7UI22_AMBAM | Lospin 7
53	cds.comp29245_c0_seq1|m.56272	2	2	R4WEH7_9HEMI | Ornithine decarboxylase Flags: Fragment
53	cds.comp124461_c0_seq1|m.205142	2	2	A1YW13_DERFA | Der f 1 allergen
54	cds.comp72134_c0_seq1|m.132054	18	5	DERP3_DERPT | Mite allergen Der p 3 EC=3.4.21.- AltName: Full=Allergen Der p III AltName: Allergen=Der p 3 Flags: Precursor
54	cds.comp72130_c0_seq1|m.132048	14	1	DERP3_DERPT | Mite allergen Der p 3 EC=3.4.21.- AltName: Full=Allergen Der p III AltName: Allergen=Der p 3 Flags: Precursor

Spot number	Accession	#Peptides	#Unique	Description
54	cds.comp86166_c0_seq1|m.154654	12	2	R4FNL2_RHOPR | Putative cathepsin b-like proteinase
54	cds.comp86168_c0_seq1|m.154662	11	0	R4FNL2_RHOPR | Putative cathepsin b-like proteinase
54	Der p 6.0101	9	9	Der p 6.0101
54	cds.comp8012_c0_seq1|m.14281	8	5	PEPT1_EURMA | Peptidase 1 EC=3.4.22.65 AltName: Full=Allergen Eur m I AltName: Full=Mite group 1 allergen Eur m 1 AltName: Allergen=Eur m 1 Flags: Precursor
54	cds.comp122816_c0_seq1|m.203611	7	7	C1IE32_9HEXA | Beta-1 3-D-glucanase EC=3.2.1.6 SubName: Full=Endo-beta-1 3-glucanase EC=3.2.1.39 Flags: Precursor
54	cds.comp118488_c0_seq1|m.198747	6	6	L7M225_9ACAR | Putative lysosomal pro-x carboxypeptidase-like protein
54	cds.comp86161_c0_seq1|m.154648	5	4	D6WGZ1_TRICA | Cathepsin B
54	cds.comp8010_c0_seq1|m.14279	5	2	PEPT1_EURMA | Peptidase 1 EC=3.4.22.65 AltName: Full=Allergen Eur m I AltName: Full=Mite group 1 allergen Eur m 1 AltName: Allergen=Eur m 1 Flags: Precursor
54	Der p 1.0107	4	4	Der p 1.0107
54	cds.comp30440_c0_seq1|m.58399	4	4	F0JA41_AMBVA | Na+/K+ ATPase beta subunit
54	cds.comp29245_c0_seq1|m.56272	4	4	R4WEH7_9HEMI | Ornithine decarboxylase Flags: Fragment
54	cds.comp92988_c0_seq1|m.165215	4	4	R4V1A4_COPFO | Enolase
54	cds.comp47520_c0_seq1|m.89357	4	4	A9QW25_CARMA | Glycosyl-phosphatidylinositol-linked carbonic anhydrase EC=4.2.1.1
54	cds.comp72078_c0_seq1|m.131978	4	4	A7UI22_AMBAM | Lospin 7
54	cds.comp124463_c0_seq1|m.205144	4	4	A1YW13_DERFA | Der f 1 allergen
54	cds.comp57928_c0_seq1|m.107765	3	3	Q7PF06_ANOGA | AGAP011050-PA
54	cds.comp110399_c0_seq1|m.187884	2	2	E2A0U6_CAMFO | N-acetylmuramoyl-L-alanine amidase
54	cds.comp27085_c0_seq1|m.52277	2	2	no hit
55	cds.comp72133_c0_seq1|m.132053	14	3	DERP3_DERPT | Mite allergen Der p 3 EC=3.4.21.- AltName: Full=Allergen Der p III AltName: Allergen=Der p 3 Flags: Precursor

Spot number	Accession	#Peptides	#Unique	Description
55	cds.comp72130_c0_seq1|m.132048	12	1	DERP3_DERPT | Mite allergen Der p 3 EC=3.4.21.- AltName: Full=Allergen Der p III AltName: Allergen=Der p 3 Flags: Precursor
55	cds.comp86166_c0_seq1|m.154654	9	2	R4FNL2_RHOPR | Putative cathepsin b-like proteinase
55	cds.comp86168_c0_seq1|m.154662	9	0	R4FNL2_RHOPR | Putative cathepsin b-like proteinase
55	cds.comp8012_c0_seq1|m.14281	8	6	PEPT1_EURMA | Peptidase 1 EC=3.4.22.65 AltName: Full=Allergen Eur m I AltName: Full=Mite group 1 allergen Eur m 1 AltName: Allergen=Eur m 1 Flags: Precursor
55	cds.comp122816_c0_seq1|m.203611	7	7	C1IE32_9HEXA | Beta-1 3-D-glucanase EC=3.2.1.6 SubName: Full=Endo-beta-1 3-glucanase EC=3.2.1.39 Flags: Precursor
55	cds.comp86161_c0_seq1|m.154648	7	5	D6WGZ1_TRICA | Cathepsin B
55	cds.comp30440_c0_seq1|m.58399	5	5	F0JA41_AMBVA | Na+/K+ ATPase beta subunit
55	Der p 6.0101	5	5	Der p 6.0101
55	Der p 1.0107	4	4	Der p 1.0107
55	cds.comp29245_c0_seq1|m.56272	4	4	R4WEH7_9HEMI | Ornithine decarboxylase Flags: Fragment
55	cds.comp72078_c0_seq1|m.131978	4	4	A7UI22_AMBAM | Lospin 7
55	cds.comp8071_c0_seq1|m.14356	3	1	PEPT1_EURMA | Peptidase 1 EC=3.4.22.65 AltName: Full=Allergen Eur m I AltName: Full=Mite group 1 allergen Eur m 1 AltName: Allergen=Eur m 1 Flags: Precursor
55	cds.comp148823_c0_seq1|m.219720	3	3	no hit
55	cds.comp27085_c0_seq1|m.52277	2	2	no hit
55	cds.comp124461_c0_seq1|m.205142	2	2	A1YW13_DERFA | Der f 1 allergen
56	cds.comp72134_c0_seq1|m.132054	16	4	DERP3_DERPT | Mite allergen Der p 3 EC=3.4.21.- AltName: Full=Allergen Der p III AltName: Allergen=Der p 3 Flags: Precursor
56	cds.comp72130_c0_seq1|m.132048	13	1	DERP3_DERPT | Mite allergen Der p 3 EC=3.4.21.- AltName: Full=Allergen Der p III AltName: Allergen=Der p 3 Flags: Precursor
56	cds.comp86166_c0_seq1|m.154654	12	2	R4FNL2_RHOPR | Putative cathepsin b-like proteinase
56	cds.comp86168_c0_seq1|m.154662	11	0	R4FNL2_RHOPR | Putative cathepsin b-like proteinase

Spot number	Accession	#Peptides	#Unique	Description
56	cds.comp122816_c0_seq1|m.203611	8	8	C1IE32_9HEXA | Beta-1 3-D-glucanase EC=3.2.1.6 SubName: Full=Endo-beta-1 3-glucanase EC=3.2.1.39 Flags: Precursor
56	Der p 6.0101	7	7	Der p 6.0101
56	cds.comp8012_c0_seq1|m.14281	4	4	PEPT1_EURMA | Peptidase 1 EC=3.4.22.65 AltName: Full=Allergen Eur m I AltName: Full=Mite group 1 allergen Eur m 1 AltName: Allergen=Eur m 1 Flags: Precursor
56	cds.comp57928_c0_seq1|m.107765	4	4	Q7PF06_ANOGA | AGAP011050-PA
56	Der p 1.0107	3	3	Der p 1.0107
56	cds.comp86161_c0_seq1|m.154648	3	2	D6WGZ1_TRICA | Cathepsin B
56	cds.comp124461_c0_seq1|m.205142	3	3	A1YW13_DERFA | Der f 1 allergen
56	cds.comp72078_c0_seq1|m.131978	2	2	A7UI22_AMBAM | Lospin 7
57	cds.comp72134_c0_seq1|m.132054	24	9	DERP3_DERPT | Mite allergen Der p 3 EC=3.4.21.- AltName: Full=Allergen Der p III AltName: Allergen=Der p 3 Flags: Precursor
57	cds.comp72130_c0_seq1|m.132048	17	2	DERP3_DERPT | Mite allergen Der p 3 EC=3.4.21.- AltName: Full=Allergen Der p III AltName: Allergen=Der p 3 Flags: Precursor
57	cds.comp86166_c0_seq1|m.154654	9	2	R4FNL2_RHOPR | Putative cathepsin b-like proteinase
57	cds.comp122816_c0_seq1|m.203611	9	9	C1IE32_9HEXA | Beta-1 3-D-glucanase EC=3.2.1.6 SubName: Full=Endo-beta-1 3-glucanase EC=3.2.1.39 Flags: Precursor
57	cds.comp86168_c0_seq1|m.154662	8	0	R4FNL2_RHOPR | Putative cathepsin b-like proteinase
57	cds.comp86161_c0_seq1|m.154648	4	3	D6WGZ1_TRICA | Cathepsin B
57	cds.comp124461_c0_seq1|m.205142	4	4	A1YW13_DERFA | Der f 1 allergen
57	cds.comp30440_c0_seq1|m.58399	3	3	F0JA41_AMBVA | Na+/K+ ATPase beta subunit
57	cds.comp86986_c0_seq1|m.155830	3	3	no hit
57	Der p 6.0101	3	3	Der p 6.0101
57	Der p 1.0107	2	2	Der p 1.0107

Spot number	Accession	#Peptides	#Unique	Description
57	cds.comp118488_c0_seq1|m.198747	2	2	L7M225_9ACAR | Putative lysosomal pro-x carboxypeptidase-like protein
57	cds.comp90809_c0_seq1|m.161776	2	2	L7M2J0_9ACAR | Putative aminopeptidase of the m17 family
57	cds.comp57928_c0_seq1|m.107765	2	2	Q7PF06_ANOGA | AGAP011050-PA
58	cds.comp72134_c0_seq1|m.132054	19	5	DERP3_DERPT | Mite allergen Der p 3 EC=3.4.21.- AltName: Full=Allergen Der p III AltName: Allergen=Der p 3 Flags: Precursor
58	cds.comp72130_c0_seq1|m.132048	15	1	DERP3_DERPT | Mite allergen Der p 3 EC=3.4.21.- AltName: Full=Allergen Der p III AltName: Allergen=Der p 3 Flags: Precursor
58	cds.comp86166_c0_seq1|m.154654	11	2	R4FNL2_RHOPR | Putative cathepsin b-like proteinase
58	cds.comp86168_c0_seq1|m.154662	10	0	R4FNL2_RHOPR | Putative cathepsin b-like proteinase
58	cds.comp122816_c0_seq1|m.203611	10	10	C1IE32_9HEXA | Beta-1 3-D-glucanase EC=3.2.1.6 SubName: Full=Endo-beta-1 3-glucanase EC=3.2.1.39 Flags: Precursor
58	cds.comp57928_c0_seq1|m.107765	6	6	Q7PF06_ANOGA | AGAP011050-PA
58	cds.comp8012_c0_seq1|m.14281	4	3	PEPT1_EURMA | Peptidase 1 EC=3.4.22.65 AltName: Full=Allergen Eur m I AltName: Full=Mite group 1 allergen Eur m 1 AltName: Allergen=Eur m 1 Flags: Precursor
58	cds.comp86161_c0_seq1|m.154648	4	3	D6WGZ1_TRICA | Cathepsin B
58	cds.comp124461_c0_seq1|m.205142	4	4	A1YW13_DERFA | Der f 1 allergen
58	Der p 6.0101	3	3	Der p 6.0101
58	cds.comp72078_c0_seq1|m.131978	3	3	A7UI22_AMBAM | Lospin 7
58	Der p 1.0107	2	2	Der p 1.0107
58	cds.comp8071_c0_seq1|m.14356	2	1	PEPT1_EURMA | Peptidase 1 EC=3.4.22.65 AltName: Full=Allergen Eur m I AltName: Full=Mite group 1 allergen Eur m 1 AltName: Allergen=Eur m 1 Flags: Precursor
59	cds.comp72134_c0_seq1|m.132054	22	7	DERP3_DERPT | Mite allergen Der p 3 EC=3.4.21.- AltName: Full=Allergen Der p III AltName: Allergen=Der p 3 Flags: Precursor
59	cds.comp72130_c0_seq1|m.132048	16	1	DERP3_DERPT | Mite allergen Der p 3 EC=3.4.21.- AltName: Full=Allergen Der p III AltName: Allergen=Der p 3 Flags: Precursor
59	cds.comp122816_c0_seq1|m.203611	13	13	C1IE32_9HEXA | Beta-1 3-D-glucanase EC=3.2.1.6 SubName: Full=Endo-beta-1 3-glucanase EC=3.2.1.39 Flags: Precursor

Spot number	Accession	#Peptides	#Unique	Description
59	cds.comp86166_c0_seq1|m.154654	12	2	R4FNL2_RHOPR | Putative cathepsin b-like proteinase
59	cds.comp86168_c0_seq1|m.154662	11	0	R4FNL2_RHOPR | Putative cathepsin b-like proteinase
59	Der p 6.0101	6	6	Der p 6.0101
59	cds.comp72078_c0_seq1|m.131978	5	5	A7UI22_AMBAM | Lospin 7
59	cds.comp86161_c0_seq1|m.154648	4	3	D6WGZ1_TRICA | Cathepsin B
59	cds.comp57928_c0_seq1|m.107765	4	4	Q7PF06_ANOGA | AGAP011050-PA
59	Der p 1.0107	3	3	Der p 1.0107
59	Der p 9.0102	3	3	Q8MWR4_DERPT Serine protease LM-1 (Fragment) OS=Dermatophagoides pteronyssinus PE=2 SV=2
59	cds.comp124461_c0_seq1|m.205142	3	3	A1YW13_DERFA | Der f 1 allergen
59	cds.comp114190_c0_seq1|m.193025	3	3	B7Q0D4_IXOSC | Fumarylacetoacetase putative EC=3.7.1.2
59	cds.comp4546_c0_seq1|m.7079	2	2	R4WCI9_9HEMI | Unkown protein
59	cds.comp123119_c0_seq1|m.203926	2	2	L7MJ22_9ACAR | Putative 3-hydroxyacyl-coa dehydrogenase Flags: Fragment
59	cds.comp29245_c0_seq1|m.56272	2	2	R4WEH7_9HEMI | Ornithine decarboxylase Flags: Fragment
59	Der p 2.0105	2	2	Der p 2.0105
59	cds.comp27085_c0_seq1|m.52277	2	2	no hit
59	cds.comp148823_c0_seq1|m.219720	2	2	no hit
60	cds.comp72134_c0_seq1|m.132054	27	10	DERP3_DERPT | Mite allergen Der p 3 EC=3.4.21.- AltName: Full=Allergen Der p III AltName: Allergen=Der p 3 Flags: Precursor
60	cds.comp72130_c0_seq1|m.132048	19	2	DERP3_DERPT | Mite allergen Der p 3 EC=3.4.21.- AltName: Full=Allergen Der p III AltName: Allergen=Der p 3 Flags: Precursor
60	cds.comp122816_c0_seq1|m.203611	8	8	C1IE32_9HEXA | Beta-1 3-D-glucanase EC=3.2.1.6 SubName: Full=Endo-beta-1 3-glucanase EC=3.2.1.39 Flags: Precursor

Spot number	Accession	#Peptides	#Unique	Description
60	cds.comp86166_c0_seq1|m.154654	7	2	R4FNL2_RHOPR | Putative cathepsin b-like proteinase
60	cds.comp86168_c0_seq1|m.154662	6	0	R4FNL2_RHOPR | Putative cathepsin b-like proteinase
60	cds.comp124461_c0_seq1|m.205142	3	3	A1YW13_DERFA | Der f 1 allergen
60	cds.comp86161_c0_seq1|m.154648	3	2	D6WGZ1_TRICA | Cathepsin B
60	cds.comp57928_c0_seq1|m.107765	2	2	Q7PF06_ANOGA | AGAP011050-PA
61	cds.comp72134_c0_seq1|m.132054	21	6	DERP3_DERPT | Mite allergen Der p 3 EC=3.4.21.- AltName: Full=Allergen Der p III AltName: Allergen=Der p 3 Flags: Precursor
61	cds.comp72130_c0_seq1|m.132048	16	1	DERP3_DERPT | Mite allergen Der p 3 EC=3.4.21.- AltName: Full=Allergen Der p III AltName: Allergen=Der p 3 Flags: Precursor
61	cds.comp122816_c0_seq1|m.203611	9	9	C1IE32_9HEXA | Beta-1 3-D-glucanase EC=3.2.1.6 SubName: Full=Endo-beta-1 3-glucanase EC=3.2.1.39 Flags: Precursor
61	cds.comp86168_c0_seq1|m.154662	8	7	R4FNL2_RHOPR | Putative cathepsin b-like proteinase
61	cds.comp123134_c0_seq1|m.203950	7	7	L7M5B4_9ACAR | Putative 3-hydroxyacyl-coa dehydrogenase
61	Der p 2.0112	6	6	Der p 2.0112
61	cds.comp57928_c0_seq1|m.107765	6	6	Q7PF06_ANOGA | AGAP011050-PA
61	cds.comp59793_c0_seq1|m.111642	5	5	Q16M58_AEDAE | AAEL012418-PA
61	cds.comp72078_c0_seq1|m.131978	5	5	A7UI22_AMBAM | Lospin 7
61	cds.comp86986_c0_seq1|m.155830	5	5	no hit
61	cds.comp123876_c0_seq1|m.204690	5	5	H9HYH7_ATTCE | 4-hydroxyphenylpyruvate dioxygenase
61	cds.comp53540_c0_seq1|m.100381	4	4	G3MH40_9ACAR | Malate dehydrogenase EC=1.1.1.37 Flags: Fragment
61	cds.comp58725_c0_seq1|m.109492	4	4	E9IA80_SOLIN | Fructose-bisphosphate aldolase EC=4.1.2.13 Flags: Fragment
61	cds.comp118488_c0_seq1|m.198747	4	4	L7M225_9ACAR | Putative lysosomal pro-x carboxypeptidase-like protein

Spot number	Accession	#Peptides	#Unique	Description
61	cds.comp124461_c0_seq1|m.205142	3	3	A1YW13_DERFA | Der f 1 allergen
61	cds.comp134821_c0_seq1|m.213456	3	3	Q7Z163_DERPT | Trypsin-like serine protease
61	cds.comp11144_c0_seq1|m.19855	3	3	B7P512_IXOSC | Acetylcholinesterase putative EC=3.1.1.7
61	cds.comp27085_c0_seq1|m.52277	3	3	no hit
61	cds.comp4546_c0_seq1|m.7079	2	2	R4WCI9_9HEMI | Unkown protein
61	cds.comp4303_c0_seq1|m.6631	2	2	E2A599_CAMFO | Lysosomal alpha-glucosidase
61	cds.comp100884_c0_seq1|m.175767	2	2	E9H9U9_DAPPU | Putative uncharacterized protein
61	cds.comp114923_c0_seq1|m.194060	2	2	Q9Y197_DERPT | Alpha-amylase Flags: Fragment
61	cds.comp86161_c0_seq1|m.154648	2	1	D6WGZ1_TRICA | Cathepsin B
61	cds.comp100562_c0_seq1|m.175130	2	2	B7Q8U6_IXOSC | Adenosine kinase putative EC=2.7.1.20
61	cds.comp32740_c0_seq1|m.62997	2	2	G1UIC8_9ARAC | Thioester-containing protein
61	cds.comp117832_c0_seq1|m.197937	2	2	B4LCX5_DROVI | GJ12921
61	cds.comp60484_c0_seq1|m.112775	2	2	L7MIP8_9ACAR | Putative beta-lactamase Flags: Fragment
62	cds.comp72134_c0_seq1|m.132054	25	10	DERP3_DERPT | Mite allergen Der p 3 EC=3.4.21.- AltName: Full=Allergen Der p III AltName: Allergen=Der p 3 Flags: Precursor
62	cds.comp72130_c0_seq1|m.132048	16	1	DERP3_DERPT | Mite allergen Der p 3 EC=3.4.21.- AltName: Full=Allergen Der p III AltName: Allergen=Der p 3 Flags: Precursor
62	cds.comp107939_c0_seq1|m.184964	10	10	A1KXI6_BLOTA | Blo t aldehyde dehydrogenase allergen
62	cds.comp123134_c0_seq1|m.203950	8	8	L7M5B4_9ACAR | Putative 3-hydroxyacyl-coa dehydrogenase
62	cds.comp57928_c0_seq1|m.107765	7	7	Q7PF06_ANOGA | AGAP011050-PA
62	cds.comp123876_c0_seq1|m.204690	7	7	H9HYH7_ATTCE | 4-hydroxyphenylpyruvate dioxygenase

Spot number	Accession	#Peptides	#Unique	Description
62	cds.comp122816_c0_seq1|m.203611	6	6	C1IE32_9HEXA | Beta-1 3-D-glucanase EC=3.2.1.6 SubName: Full=Endo-beta-1 3-glucanase EC=3.2.1.39 Flags: Precursor
62	cds.comp53540_c0_seq1|m.100381	6	6	G3MH40_9ACAR | Malate dehydrogenase EC=1.1.1.37 Flags: Fragment
62	cds.comp86168_c0_seq1|m.154662	4	3	R4FNL2_RHOPR | Putative cathepsin b-like proteinase
62	cds.comp59793_c0_seq1|m.111642	4	4	Q16M58_AEDAE | AAEL012418-PA
62	cds.comp86986_c0_seq1|m.155830	4	4	no hit
62	cds.comp124461_c0_seq1|m.205142	4	4	A1YW13_DERFA | Der f 1 allergen
62	cds.comp134821_c0_seq1|m.213456	3	3	Q7Z163_DERPT | Trypsin-like serine protease
62	cds.comp32740_c0_seq1|m.62997	3	3	G1UIC8_9ARAC | Thioester-containing protein
62	cds.comp4303_c0_seq1|m.6631	2	2	E2A599_CAMFO | Lysosomal alpha-glucosidase
62	cds.comp86172_c0_seq1|m.154673	2	1	R4FNL2_RHOPR | Putative cathepsin b-like proteinase
62	cds.comp114923_c0_seq1|m.194060	2	2	Q9Y197_DERPT | Alpha-amylase Flags: Fragment
63	cds.comp72134_c0_seq1|m.132054	22	1	DERP3_DERPT | Mite allergen Der p 3 EC=3.4.21.- AltName: Full=Allergen Der p III AltName: Allergen=Der p 3 Flags: Precursor
63	Der p 3.0101	22	1	P39675_DERP3_DERPT Mite allergen Der p 3 OS=Dermatophagoides pteronyssinus GN=DERP3 PE=1 SV=1
63	cds.comp72130_c0_seq1|m.132048	15	1	DERP3_DERPT | Mite allergen Der p 3 EC=3.4.21.- AltName: Full=Allergen Der p III AltName: Allergen=Der p 3 Flags: Precursor
63	cds.comp134821_c0_seq1|m.213456	7	7	Q7Z163_DERPT | Trypsin-like serine protease
63	cds.comp123115_c0_seq1|m.203925	7	7	L7M5B4_9ACAR | Putative 3-hydroxyacyl-coa dehydrogenase
63	cds.comp59796_c0_seq1|m.111647	5	5	Q16M58_AEDAE | AAEL012418-PA
63	cds.comp100562_c0_seq1|m.175130	5	5	B7Q8U6_IXOSC | Adenosine kinase putative EC=2.7.1.20
63	cds.comp57928_c0_seq1|m.107765	4	4	Q7PF06_ANOGA | AGAP011050-PA

Spot number	Accession	#Peptides	#Unique	Description
63	Der p 2.0104	4	4	Der p 2.0104
63	cds.comp86986_c0_seq1|m.155830	3	3	no hit
63	cds.comp122816_c0_seq1|m.203611	3	3	C1IE32_9HEXA | Beta-1 3-D-glucanase EC=3.2.1.6 SubName: Full=Endo-beta-1 3-glucanase EC=3.2.1.39 Flags: Precursor
63	cds.comp58725_c0_seq1|m.109492	2	2	E9IA80_SOLIN | Fructose-bisphosphate aldolase EC=4.1.2.13 Flags: Fragment
63	cds.comp114923_c0_seq1|m.194060	2	2	Q9Y197_DERPT | Alpha-amylase Flags: Fragment
64	Der p 3.0101	28	12	P39675_DERP3_DERPT Mite allergen Der p 3 OS=Dermatophagoides pteronyssinus GN=DERP3 PE=1 SV=1
64	cds.comp72130_c0_seq1|m.132048	17	1	DERP3_DERPT | Mite allergen Der p 3 EC=3.4.21.- AltName: Full=Allergen Der p III AltName: Allergen=Der p 3 Flags: Precursor
64	cds.comp134821_c0_seq1|m.213456	7	7	Q7Z163_DERPT | Trypsin-like serine protease
64	cds.comp86986_c0_seq1|m.155830	7	7	no hit
64	cds.comp58725_c0_seq1|m.109492	4	4	E9IA80_SOLIN | Fructose-bisphosphate aldolase EC=4.1.2.13 Flags: Fragment
64	cds.comp100562_c0_seq1|m.175130	4	4	B7Q8U6_IXOSC | Adenosine kinase putative EC=2.7.1.20
64	Der p 1.0107	3	3	Der p 1.0107
64	cds.comp77087_c0_seq1|m.140422	3	3	E2BH78_HARSA | Phosphatidylinositol-specific phospholipase C X domain-containing protein 1
64	cds.comp4303_c0_seq1|m.6631	2	2	E2A599_CAMFO | Lysosomal alpha-glucosidase
64	cds.comp86983_c0_seq1|m.155817	2	2	no hit
64	cds.comp53540_c0_seq1|m.100381	2	2	G3MH40_9ACAR | Malate dehydrogenase EC=1.1.1.37 Flags: Fragment
64	cds.comp4654_c0_seq1|m.7303	2	2	no hit
64	cds.comp61446_c0_seq1|m.114264	2	2	B7PAQ0_IXOSC | Gamma-glutamyltransferase putative EC=2.3.2.2
64	cds.comp90671_c0_seq1|m.161623	2	2	E9FTN1_DAPPU | Beta-galactosidase EC=3.2.1.23

Spot number	Accession	#Peptides	#Unique	Description
64	cds.comp57928_c0_seq1|m.107765	2	2	Q7PF06_ANOGA | AGAP011050-PA
64	cds.comp75373_c0_seq1|m.137683	2	2	D3PI43_9MAXI | Platelet-activating factor acetylhydrolase IB subunit gamma
65	Der p 3.0101	28	12	P39675_DERP3_DERPT Mite allergen Der p 3 OS=Dermatophagoides pteronyssinus GN=DERP3 PE=1 SV=1
65	cds.comp72130_c0_seq1|m.132048	17	1	DERP3_DERPT | Mite allergen Der p 3 EC=3.4.21.- AltName: Full=Allergen Der p III AltName: Allergen=Der p 3 Flags: Precursor
65	cds.comp134821_c0_seq1|m.213456	8	8	Q7Z163_DERPT | Trypsin-like serine protease
65	cds.comp86986_c0_seq1|m.155830	6	6	no hit
65	cds.comp18135_c0_seq1|m.33902	6	6	K7J833_NASVI | Malate dehydrogenase EC=1.1.1.37
65	cds.comp100562_c0_seq1|m.175130	4	4	B7Q8U6_IXOSC | Adenosine kinase putative EC=2.7.1.20
65	cds.comp57928_c0_seq1|m.107765	3	3	Q7PF06_ANOGA | AGAP011050-PA
65	cds.comp86983_c0_seq1|m.155817	2	2	no hit
65	cds.comp4303_c0_seq1|m.6631	2	2	E2A599_CAMFO | Lysosomal alpha-glucosidase
65	cds.comp4654_c0_seq1|m.7303	2	2	no hit
65	cds.comp61446_c0_seq1|m.114264	2	2	B7PAQ0_IXOSC | Gamma-glutamyltransferase putative EC=2.3.2.2
65	cds.comp90671_c0_seq1|m.161623	2	2	E9FTN1_DAPPU | Beta-galactosidase EC=3.2.1.23
65	cds.comp77087_c0_seq1|m.140422	2	2	E2BH78_HARSA | Phosphatidylinositol-specific phospholipase C X domain-containing protein 1
65	cds.comp95432_c0_seq1|m.168277	2	2	L7M7J0_9ACAR | Putative ribonuclease t2 family
65	cds.comp92389_c0_seq1|m.164353	2	2	D6WCM8_TRICA | Beta-galactosidase EC=3.2.1.23
66	Der p 3.0101	24	9	P39675_DERP3_DERPT Mite allergen Der p 3 OS=Dermatophagoides pteronyssinus GN=DERP3 PE=1 SV=1
66	cds.comp72130_c0_seq1|m.132048	16	1	DERP3_DERPT | Mite allergen Der p 3 EC=3.4.21.- AltName: Full=Allergen Der p III AltName: Allergen=Der p 3 Flags: Precursor

Spot number	Accession	#Peptides	#Unique	Description
66	cds.comp18135_c0_seq1|m.33902	6	6	K7J833_NASVI | Malate dehydrogenase EC=1.1.1.37
66	cds.comp134821_c0_seq1|m.213456	6	6	Q7Z163_DERPT | Trypsin-like serine protease
66	cds.comp86986_c0_seq1|m.155830	6	6	no hit
66	cds.comp86983_c0_seq1|m.155817	2	2	no hit
66	cds.comp4303_c0_seq1|m.6631	2	2	E2A599_CAMFO | Lysosomal alpha-glucosidase
66	cds.comp95432_c0_seq1|m.168277	2	2	L7M7J0_9ACAR | Putative ribonuclease t2 family
67	Der p 3.0101	30	15	P39675_DERP3_DERPT Mite allergen Der p 3 OS=Dermatophagoides pteronyssinus GN=DERP3 PE=1 SV=1
67	cds.comp72130_c0_seq1|m.132048	16	1	DERP3_DERPT | Mite allergen Der p 3 EC=3.4.21.- AltName: Full=Allergen Der p III AltName: Allergen=Der p 3 Flags: Precursor
67	cds.comp18135_c0_seq1|m.33902	10	10	K7J833_NASVI | Malate dehydrogenase EC=1.1.1.37
67	cds.comp134821_c0_seq1|m.213456	8	8	Q7Z163_DERPT | Trypsin-like serine protease
67	cds.comp86986_c0_seq1|m.155830	6	6	no hit
67	cds.comp95432_c0_seq1|m.168277	3	3	L7M7J0_9ACAR | Putative ribonuclease t2 family
67	cds.comp4303_c0_seq1|m.6631	2	2	E2A599_CAMFO | Lysosomal alpha-glucosidase
67	cds.comp4654_c0_seq1|m.7303	2	2	no hit
67	cds.comp104639_c0_seq1|m.180559	2	2	B7PLR3_IXOSC | Filamin-C putative EC=3.1.3.48
68	Der p 1.0116	10	0	Der p 1.0116
68	Der p 1.0108	9	0	Der p 1.0108
68	cds.comp116086_c0_seq1|m.195553	9	1	PEPT1_DERPT | Peptidase 1 EC=3.4.22.65 AltName: Full=Allergen Der p I AltName: Full=Major mite fecal allergen Der p 1 AltName: Allergen=Der p 1 Flags: Precursor
68	Der p 1.0103	8	1	Der p 1.0103

Spot number	Accession	#Peptides	#Unique	Description
68	cds.comp136840_c0_seq1|m.214779	5	5	L7UZA7_DERFA | Triosephosphate isomerase EC=5.3.1.1
68	cds.comp86168_c0_seq1|m.154662	4	3	R4FNL2_RHOPR | Putative cathepsin b-like proteinase
68	cds.comp86161_c0_seq1|m.154648	4	3	D6WGZ1_TRICA | Cathepsin B
68	cds.comp136643_c0_seq1|m.214661	4	4	E2C2J9_HARSA | Pancreatic triacylglycerol lipase
68	cds.comp128133_c0_seq1|m.208437	3	3	C1IE32_9HEXA | Beta-1 3-D-glucanase EC=3.2.1.6 SubName: Full=Endo-beta-1 3-glucanase EC=3.2.1.39 Flags: Precursor
68	Der p 6.0101	3	3	Der p 6.0101
68	cds.comp8014_c0_seq1|m.14284	2	2	PEPT1_EURMA | Peptidase 1 EC=3.4.22.65 AltName: Full=Allergen Eur m I AltName: Full=Mite group 1 allergen Eur m 1 AltName: Allergen=Eur m 1 Flags: Precursor
68	cds.comp86985_c0_seq1|m.155823	2	2	no hit
68	cds.comp124461_c0_seq1|m.205142	2	2	A1YW13_DERFA | Der f 1 allergen
69	Der p 1.0115	11	0	Der p 1.0115
69	Der p 1.0121	11	0	Der p 1.0121
69	cds.comp116086_c0_seq1|m.195553	9	2	PEPT1_DERPT | Peptidase 1 EC=3.4.22.65 AltName: Full=Allergen Der p I AltName: Full=Major mite fecal allergen Der p 1 AltName: Allergen=Der p 1 Flags: Precursor
69	Der p 1.0103	9	1	Der p 1.0103
69	cds.comp86168_c0_seq1|m.154662	5	4	R4FNL2_RHOPR | Putative cathepsin b-like proteinase
69	cds.comp136840_c0_seq1|m.214779	5	5	L7UZA7_DERFA | Triosephosphate isomerase EC=5.3.1.1
69	Der p 6.0101	4	4	Der p 6.0101
69	cds.comp8063_c0_seq1|m.14348	3	3	A1KXH2_DERFA | Der f 1 allergen
69	cds.comp86161_c0_seq1|m.154648	3	2	D6WGZ1_TRICA | Cathepsin B
69	cds.comp62314_c0_seq1|m.115830	2	2	B7Q0R0_IXOSC | Phosphoglycerate mutase putative EC=5.4.2.1

Spot number	Accession	#Peptides	#Unique	Description
69	cds.comp58725_c0_seq1|m.109492	2	2	E9IA80_SOLIN | Fructose-bisphosphate aldolase EC=4.1.2.13 Flags: Fragment
70	Der p 1.0105	11	0	Der p 1.0105
70	Der p 1.0121	11	2	Der p 1.0121
70	cds.comp116086_c0_seq1|m.195553	9	2	PEPT1_DERPT | Peptidase 1 EC=3.4.22.65 AltName: Full=Allergen Der p I AltName: Full=Major mite fecal allergen Der p 1 AltName: Allergen=Der p 1 Flags: Precursor
70	cds.comp136840_c0_seq1|m.214779	5	5	L7UZA7_DERFA | Triosephosphate isomerase EC=5.3.1.1
70	cds.comp86168_c0_seq1|m.154662	4	3	R4FNL2_RHOPR | Putative cathepsin b-like proteinase
70	cds.comp117252_c0_seq1|m.197148	4	4	ALL2_PSOOV | Mite group 2 allergen Pso o 2 AltName: Full=Allergen Pso o A AltName: Allergen=Pso o 2 Flags: Precursor
70	cds.comp86161_c0_seq1|m.154648	4	3	D6WGZ1_TRICA | Cathepsin B
70	Der p 6.0101	3	3	Der p 6.0101
70	cds.comp58728_c0_seq1|m.109496	2	2	G9C5D5_SCHGR | Fructose-bisphosphate aldolase EC=4.1.2.13
71	cds.comp136841_c0_seq1|m.214780	10	10	L7UZA7_DERFA | Triosephosphate isomerase EC=5.3.1.1
71	Der p 1.0116	9	0	Der p 1.0116
71	Der p 1.0121	8	1	Der p 1.0121
71	cds.comp116084_c0_seq1|m.195549	8	2	PEPT1_DERPT | Peptidase 1 EC=3.4.22.65 AltName: Full=Allergen Der p I AltName: Full=Major mite fecal allergen Der p 1 AltName: Allergen=Der p 1 Flags: Precursor
71	cds.comp86168_c0_seq1|m.154662	4	3	R4FNL2_RHOPR | Putative cathepsin b-like proteinase
71	cds.comp112995_c0_seq1|m.191100	4	4	Q28XY6_DROPS | GA19673 isoform A SubName: Full=GA19673 isoform B
71	cds.comp86162_c0_seq1|m.154650	3	2	D6WGZ1_TRICA | Cathepsin B
71	cds.comp94280_c0_seq1|m.166903	2	2	ALL2_DERPT | Mite group 2 allergen Der p 2 AltName: Full=Allergen Der p II AltName: Full=DPX AltName: Allergen=Der p 2 Flags: Precursor
71	cds.comp122816_c0_seq1|m.203611	2	2	C1IE32_9HEXA | Beta-1 3-D-glucanase EC=3.2.1.6 SubName: Full=Endo-beta-1 3-glucanase EC=3.2.1.39 Flags: Precursor

Spot number	Accession	#Peptides	#Unique	Description
71	cds.comp11138_c0_seq1|m.19849	2	2	B3TFG6_9ACAR | Esterase TCE1
71	cds.comp124461_c0_seq1|m.205142	2	2	A1YW13_DERFA | Der f 1 allergen
72	Der p 1.0121	16	2	Der p 1.0121
72	Der p 1.0105	16	0	Der p 1.0105
72	cds.comp116086_c0_seq1|m.195553	13	2	PEPT1_DERPT | Peptidase 1 EC=3.4.22.65 AltName: Full=Allergen Der p I AltName: Full=Major mite fecal allergen Der p 1 AltName: Allergen=Der p 1 Flags: Precursor
72	cds.comp136840_c0_seq1|m.214779	9	9	L7UZA7_DERFA | Triosephosphate isomerase EC=5.3.1.1
72	cds.comp86164_c0_seq1|m.154651	3	3	B0W0V3_CULQU | Cathepsin L
72	cds.comp74118_c0_seq1|m.135887	2	2	Q2YFF0_SARSC | Glutathione transferase mu class Yv5004H11
72	cds.comp94280_c0_seq1|m.166903	2	2	ALL2_DERPT | Mite group 2 allergen Der p 2 AltName: Full=Allergen Der p II AltName: Full=DPX AltName: Allergen=Der p 2 Flags: Precursor
72	Der p 6.0101	2	2	Der p 6.0101
72	cds.comp124461_c0_seq1|m.205142	2	2	A1YW13_DERFA | Der f 1 allergen
73	cds.comp123878_c0_seq1|m.204693	11	11	K7J3E2_NASVI | 4-hydroxyphenylpyruvate dioxygenase
73	Der p 1.0108	8	1	Der p 1.0108
73	Der p 1.0118	8	0	Der p 1.0118
73	cds.comp136840_c0_seq1|m.214779	8	8	L7UZA7_DERFA | Triosephosphate isomerase EC=5.3.1.1
73	cds.comp116086_c0_seq1|m.195553	7	2	PEPT1_DERPT | Peptidase 1 EC=3.4.22.65 AltName: Full=Allergen Der p I AltName: Full=Major mite fecal allergen Der p 1 AltName: Allergen=Der p 1 Flags: Precursor
73	Der p 2.0104	7	7	Der p 2.0104
73	cds.comp58725_c0_seq1|m.109492	6	6	E9IA80_SOLIN | Fructose-bisphosphate aldolase EC=4.1.2.13 Flags: Fragment
73	cds.comp35806_c0_seq1|m.69175	6	6	D2SNU8_HELVI | Short-chain dehydrogenase

Spot number	Accession	#Peptides	#Unique	Description
73	cds.comp122816_c0_seq1|m.203611	4	4	C1IE32_9HEXA | Beta-1 3-D-glucanase EC=3.2.1.6 SubName: Full=Endo-beta-1 3-glucanase EC=3.2.1.39 Flags: Precursor
73	cds.comp114225_c0_seq1|m.193096	4	4	Q5TQ02_ANOGA | AGAP011457-PA Flags: Fragment
73	cds.comp88963_c0_seq1|m.159079	3	3	L7M287_9ACAR | Glutathione peroxidase
73	cds.comp8010_c0_seq1|m.14279	3	3	PEPT1_EURMA | Peptidase 1 EC=3.4.22.65 AltName: Full=Allergen Eur m I AltName: Full=Mite group 1 allergen Eur m 1 AltName: Allergen=Eur m 1 Flags: Precursor
73	Der p 8.0101	2	2	P46419_GSTM1_DERPT Glutathione S-transferase OS=Dermatophagoides pteronyssinus PE=1 SV=1
73	cds.comp86985_c0_seq1|m.155823	2	2	no hit
73	cds.comp114625_c0_seq1|m.193683	2	2	A1KXH6_DERFA | Der f 8 allergen Flags: Fragment
73	cds.comp72078_c0_seq1|m.131978	2	2	A7UI22_AMBAM | Lospin 7
73	cds.comp116578_c0_seq1|m.196362	2	2	Q3Y596_MACRS | Superoxide dismutase EC=1.15.1.1
73	cds.comp117252_c0_seq1|m.197148	2	2	ALL2_PSOOV | Mite group 2 allergen Pso o 2 AltName: Full=Allergen Pso o A AltName: Allergen=Pso o 2 Flags: Precursor
73	cds.comp75373_c0_seq1|m.137683	2	2	D3PI43_9MAXI | Platelet-activating factor acetylhydrolase IB subunit gamma
73	cds.comp61446_c0_seq1|m.114264	2	2	B7PAQ0_IXOSC | Gamma-glutamyltransferase putative EC=2.3.2.2
73	cds.comp112919_c0_seq1|m.190975	2	2	no hit
73	cds.comp57928_c0_seq1|m.107765	2	2	Q7PF06_ANOGA | AGAP011050-PA
73	cds.comp72130_c0_seq1|m.132048	2	2	DERP3_DERPT | Mite allergen Der p 3 EC=3.4.21.- AltName: Full=Allergen Der p III AltName: Allergen=Der p 3 Flags: Precursor
73	cds.comp124461_c0_seq1|m.205142	2	2	A1YW13_DERFA | Der f 1 allergen
73	cds.comp87578_c0_seq1|m.156729	2	2	B7QC64_IXOSC | Glutathione S-transferase kappa putative EC=2.5.1.18
74	cds.comp136840_c0_seq1|m.214779	19	19	L7UZA7_DERFA | Triosephosphate isomerase EC=5.3.1.1
74	Der p 2.0112	8	1	Der p 2.0112

Spot number	Accession	#Peptides	#Unique	Description
74	Der p 2.0109	8	1	Der p 2.0109
74	Der p 1.0118	6	3	Der p 1.0118
74	cds.comp116086_c0_seq1|m.195553	5	2	PEPT1_DERPT | Peptidase 1 EC=3.4.22.65 AltName: Full=Allergen Der p I AltName: Full=Major mite fecal allergen Der p 1 AltName: Allergen=Der p 1 Flags: Precursor
74	cds.comp58725_c0_seq1|m.109492	5	5	E9IA80_SOLIN | Fructose-bisphosphate aldolase EC=4.1.2.13 Flags: Fragment
74	cds.comp47794_c0_seq1|m.89789	5	5	L7M2A4_9ACAR | Putative alpha actinin
74	cds.comp55438_c0_seq1|m.103707	5	5	B7PEY0_IXOSC | AP-2 complex subunit alpha-1 putative
74	cds.comp86986_c0_seq1|m.155830	4	4	no hit
74	Der p 9.0101	3	3	Q7Z163_DERPT Trypsin-like serine protease OS=Dermatophagoides pteronyssinus PE=2 SV=1
74	cds.comp120093_c0_seq1|m.200515	3	3	B7QGR2_IXOSC | Apical endosomal glycoprotein putative Flags: Fragment
74	cds.comp117504_c0_seq1|m.197521	3	3	F4WNF6_ACREC | Proteasome subunit alpha type EC=3.4.25.1 Flags: Fragment
74	cds.comp72078_c0_seq1|m.131978	3	3	A7UI22_AMBAM | Lospin 7
74	cds.comp8010_c0_seq1|m.14279	3	3	PEPT1_EURMA | Peptidase 1 EC=3.4.22.65 AltName: Full=Allergen Eur m I AltName: Full=Mite group 1 allergen Eur m 1 AltName: Allergen=Eur m 1 Flags: Precursor
74	cds.comp8016_c0_seq1|m.14286	3	3	PEPT1_EURMA | Peptidase 1 EC=3.4.22.65 AltName: Full=Allergen Eur m I AltName: Full=Mite group 1 allergen Eur m 1 AltName: Allergen=Eur m 1 Flags: Precursor
74	cds.comp77087_c0_seq1|m.140422	3	3	E2BH78_HARSA | Phosphatidylinositol-specific phospholipase C X domain-containing protein 1
74	cds.comp95432_c0_seq1|m.168277	2	2	L7M7J0_9ACAR | Putative ribonuclease t2 family
74	cds.comp95433_c0_seq1|m.168278	2	2	L7M7J0_9ACAR | Putative ribonuclease t2 family
74	cds.comp112995_c0_seq1|m.191100	2	2	Q28XY6_DROPS | GA19673 isoform A SubName: Full=GA19673 isoform B
74	cds.comp90293_c0_seq1|m.161025	2	2	B7PSJ2_IXOSC | Proteasome subunit alpha type EC=3.4.25.1
74	cds.comp116393_c0_seq1|m.196074	2	2	L7M9E3_9ACAR | Putative beta adaptin

Spot number	Accession	#Peptides	#Unique	Description
74	cds.comp61446_c0_seq1|m.114264	2	2	B7PAQ0_IXOSC | Gamma-glutamyltransferase putative EC=2.3.2.2
74	cds.comp72133_c0_seq1|m.132053	2	2	DERP3_DERPT | Mite allergen Der p 3 EC=3.4.21.- AltName: Full=Allergen Der p III AltName: Allergen=Der p 3 Flags: Precursor
74	cds.comp75373_c0_seq1|m.137683	2	2	D3PI43_9MAXI | Platelet-activating factor acetylhydrolase IB subunit gamma
74	cds.comp8009_c0_seq1|m.14277	2	2	PEPT1_EURMA | Peptidase 1 EC=3.4.22.65 AltName: Full=Allergen Eur m I AltName: Full=Mite group 1 allergen Eur m 1 AltName: Allergen=Eur m 1 Flags: Precursor
74	cds.comp114923_c0_seq1|m.194060	2	2	Q9Y197_DERPT | Alpha-amylase Flags: Fragment
75	cds.comp58725_c0_seq1|m.109492	15	15	E9IA80_SOLIN | Fructose-bisphosphate aldolase EC=4.1.2.13 Flags: Fragment
75	Der p 2.0112	8	8	Der p 2.0112
75	cds.comp87576_c0_seq1|m.156724	7	7	B7QC64_IXOSC | Glutathione S-transferase kappa putative EC=2.5.1.18
75	cds.comp35806_c0_seq1|m.69175	7	7	D2SNU8_HELVI | Short-chain dehydrogenase
75	cds.comp114625_c0_seq1|m.193683	7	6	A1KXH6_DERFA | Der f 8 allergen Flags: Fragment
75	Der p 1.0122	6	3	Der p 1.0122
75	cds.comp120091_c0_seq1|m.200514	6	6	B7QGR2_IXOSC | Apical endosomal glycoprotein putative Flags: Fragment
75	cds.comp116393_c0_seq1|m.196074	6	6	L7M9E3_9ACAR | Putative beta adaptin
75	cds.comp75040_c0_seq1|m.137217	5	5	E0VP09_PEDHC | Protein-L-isoaspartate O-methyltransferase EC=2.1.1.77
75	cds.comp61446_c0_seq1|m.114264	5	5	B7PAQ0_IXOSC | Gamma-glutamyltransferase putative EC=2.3.2.2
75	cds.comp116086_c0_seq1|m.195553	4	1	PEPT1_DERPT | Peptidase 1 EC=3.4.22.65 AltName: Full=Allergen Der p I AltName: Full=Major mite fecal allergen Der p 1 AltName: Allergen=Der p 1 Flags: Precursor
75	cds.comp88453_c0_seq1|m.158224	4	4	Q09JF9_ARGMO | Mitochondrial associated endoribonuclease MAR1-isochorismatase superfamily
75	cds.comp112920_c0_seq1|m.190979	4	4	no hit
75	Der p 8.0101	3	2	P46419_GSTM1_DERPT Glutathione S-transferase OS=Dermatophagoides pteronyssinus PE=1 SV=1

Spot number	Accession	#Peptides	#Unique	Description
75	cds.comp75369_c0_seq1|m.137678	3	2	E0W141_PEDHC | Platelet-activating factor acetylhydrolase ib putative
75	cds.comp116578_c0_seq1|m.196362	3	3	Q3Y596_MACRS | Superoxide dismutase EC=1.15.1.1
75	cds.comp72078_c0_seq1|m.131978	3	3	A7UI22_AMBAM | Lospin 7
75	cds.comp70238_c0_seq1|m.128857	3	3	L7M422_9ACAR | Putative pyridoxamine-phosphate oxidase
75	cds.comp15338_c0_seq1|m.28443	3	2	Q2YFE4_DERPT | Glutathione transferase mu class Dp7002H05
75	cds.comp72133_c0_seq1|m.132053	3	3	DERP3_DERPT | Mite allergen Der p 3 EC=3.4.21.- AltName: Full=Allergen Der p III AltName: Allergen=Der p 3 Flags: Precursor
75	cds.comp5428_c0_seq1|m.9113	3	3	E2AIC3_CAMFO | Pyridoxine-5'-phosphate oxidase
75	cds.comp136839_c0_seq1|m.214778	2	2	L7UZA7_DERFA | Triosephosphate isomerase EC=5.3.1.1
75	cds.comp88963_c0_seq1|m.159079	2	2	L7M287_9ACAR | Glutathione peroxidase
75	cds.comp75372_c0_seq1|m.137682	2	1	B4MGD8_DROVI | GJ18477
75	cds.comp92386_c0_seq1|m.164348	2	2	D6WCN0_TRICA | Beta-galactosidase EC=3.2.1.23
75	cds.comp9610_c0_seq1|m.17297	2	2	E0VCV6_PEDHC | Carboxypeptidase M putative EC=3.4.17.22
75	cds.comp114923_c0_seq1|m.194060	2	2	Q9Y197_DERPT | Alpha-amylase Flags: Fragment
75	cds.comp95575_c0_seq1|m.168518	2	2	G3MMG5_9ACAR | Proteasome subunit alpha type EC=3.4.25.1
76	cds.comp114625_c0_seq1|m.193683	9	7	A1KXH6_DERFA | Der f 8 allergen Flags: Fragment
76	Der p 8.0101	7	5	P46419_GSTM1_DERPT Glutathione S-transferase OS=Dermatophagoides pteronyssinus PE=1 SV=1
76	cds.comp95432_c0_seq1|m.168277	7	7	L7M7J0_9ACAR | Putative ribonuclease t2 family
76	cds.comp15338_c0_seq1|m.28443	7	5	Q2YFE4_DERPT | Glutathione transferase mu class Dp7002H05
76	cds.comp35506_c0_seq1|m.68564	6	6	G6DQU9_DANPL | Proteasome subunit beta type EC=3.4.25.1

Spot number	Accession	#Peptides	#Unique	Description
76	cds.comp15338_c0_seq1|m.28442	6	6	Q2YFE4_DERPT | Glutathione transferase mu class Dp7002H05
76	cds.comp53420_c0_seq1|m.100211	4	4	B4PC03_DROYA | GE11380
76	Der p 1.0114	3	2	Der p 1.0114
76	cds.comp83765_c0_seq1|m.150899	3	3	E9G1A4_DAPPU | Putative uncharacterized protein
76	cds.comp116086_c0_seq1|m.195553	2	1	PEPT1_DERPT | Peptidase 1 EC=3.4.22.65 AltName: Full=Allergen Der p I AltName: Full=Major mite fecal allergen Der p 1 AltName: Allergen=Der p 1 Flags: Precursor
76	cds.comp72133_c0_seq1|m.132053	2	2	DERP3_DERPT | Mite allergen Der p 3 EC=3.4.21.- AltName: Full=Allergen Der p III AltName: Allergen=Der p 3 Flags: Precursor
76	cds.comp95575_c0_seq1|m.168518	2	2	G3MMG5_9ACAR | Proteasome subunit alpha type EC=3.4.25.1
76	cds.comp58725_c0_seq1|m.109492	2	2	E9IA80_SOLIN | Fructose-bisphosphate aldolase EC=4.1.2.13 Flags: Fragment
76	cds.comp61446_c0_seq1|m.114264	2	2	B7PAQ0_IXOSC | Gamma-glutamyltransferase putative EC=2.3.2.2
76	cds.comp122683_c0_seq1|m.203466	2	2	R4WDM8_9HEMI | Short-chain dehydrogenase
77	cds.comp15338_c0_seq1|m.28443	7	5	Q2YFE4_DERPT | Glutathione transferase mu class Dp7002H05
77	cds.comp114625_c0_seq1|m.193683	7	5	A1KXH6_DERFA | Der f 8 allergen Flags: Fragment
77	cds.comp15338_c0_seq1|m.28442	6	6	Q2YFE4_DERPT | Glutathione transferase mu class Dp7002H05
77	cds.comp95432_c0_seq1|m.168277	6	6	L7M7J0_9ACAR | Putative ribonuclease t2 family
77	cds.comp35506_c0_seq1|m.68564	5	5	G6DQU9_DANPL | Proteasome subunit beta type EC=3.4.25.1
77	cds.comp53420_c0_seq1|m.100211	4	4	B4PC03_DROYA | GE11380
77	cds.comp122683_c0_seq1|m.203466	3	3	R4WDM8_9HEMI | Short-chain dehydrogenase
77	Der p 8.0101	3	1	P46419_GSTM1_DERPT Glutathione S-transferase OS=Dermatophagoides pteronyssinus PE=1 SV=1
78	cds.comp134821_c0_seq1|m.213456	16	16	Q7Z163_DERPT | Trypsin-like serine protease

Spot number	Accession	#Peptides	#Unique	Description
78	cds.comp95432_c0_seq1|m.168277	4	4	L7M7J0_9ACAR | Putative ribonuclease t2 family
79	cds.comp117245_c0_seq1|m.197139	3	3	ALL2_PSOOV | Mite group 2 allergen Pso o 2 AltName: Full=Allergen Pso o A AltName: Allergen=Pso o 2 Flags: Precursor
79	cds.comp8071_c0_seq1|m.14356	2	2	PEPT1_EURMA | Peptidase 1 EC=3.4.22.65 AltName: Full=Allergen Eur m I AltName: Full=Mite group 1 allergen Eur m 1 AltName: Allergen=Eur m 1 Flags: Precursor
79	cds.comp129013_c0_seq1|m.209205	2	2	B4JYH8_DROGR | GH14296
80	Der p 2.0112	10	10	Der p 2.0112
80	cds.comp117252_c0_seq1|m.197148	5	5	ALL2_PSOOV | Mite group 2 allergen Pso o 2 AltName: Full=Allergen Pso o A AltName: Allergen=Pso o 2 Flags: Precursor
80	cds.comp142309_c0_seq1|m.217543	5	5	I7E449_ANTYA | Trypsin
80	Der p 1.0106	3	2	Der p 1.0106
80	cds.comp129013_c0_seq1|m.209205	3	3	B4JYH8_DROGR | GH14296
80	cds.comp107339_c0_seq1|m.184045	3	3	L7LUC2_9ACAR | Adenylyl cyclase-associated protein
80	cds.comp116086_c0_seq1|m.195553	2	1	PEPT1_DERPT | Peptidase 1 EC=3.4.22.65 AltName: Full=Allergen Der p I AltName: Full=Major mite fecal allergen Der p 1 AltName: Allergen=Der p 1 Flags: Precursor
80	cds.comp39349_c0_seq1|m.74988	2	2	Q09JE3_ARGMO | Superoxide dismutase [Cu-Zn] EC=1.15.1.1
80	cds.comp11404_c0_seq1|m.20428	2	2	A5X5X4_DERFA | Allergen SubName: Full=Group 22 allergen Der f 22
80	cds.comp2827_c0_seq1|m.3911	2	2	L7UZ91_DERFA | Ferritin
80	cds.comp92386_c0_seq1|m.164348	2	2	D6WCN0_TRICA | Beta-galactosidase EC=3.2.1.23
81	Der p 2.0112	8	1	Der p 2.0112
81	Der p 2.0109	8	1	Der p 2.0109
81	cds.comp117252_c0_seq1|m.197148	5	5	ALL2_PSOOV | Mite group 2 allergen Pso o 2 AltName: Full=Allergen Pso o A AltName: Allergen=Pso o 2 Flags: Precursor
81	cds.comp113514_c0_seq1|m.191868	4	4	B4PW26_DROYA | GE14560

Spot number	Accession	#Peptides	#Unique	Description
81	cds.comp29245_c0_seq1|m.56272	4	4	R4WEH7_9HEMI | Ornithine decarboxylase Flags: Fragment
81	Der p 1.0120	3	2	Der p 1.0120
81	cds.comp67992_c0_seq1|m.124878	3	3	Q66RP5_TYRPU | Fatty acid-biding protein
81	cds.comp116084_c0_seq1|m.195549	2	1	PEPT1_DERPT | Peptidase 1 EC=3.4.22.65 AltName: Full=Allergen Der p I AltName: Full=Major mite fecal allergen Der p 1 AltName: Allergen=Der p 1 Flags: Precursor
81	cds.comp70421_c0_seq1|m.129141	2	2	no hit
81	cds.comp120093_c0_seq1|m.200515	2	2	B7QGR2_IXOSC | Apical endosomal glycoprotein putative Flags: Fragment
81	cds.comp46771_c0_seq1|m.88350	2	2	B7Q8W6_IXOSC | Alkyl hydroperoxide reductase thiol specific antioxidant putative EC=1.11.1.15
82	Der p 2.0112	10	1	Der p 2.0112
82	Der p 2.0109	10	1	Der p 2.0109
82	cds.comp117252_c0_seq1|m.197148	8	8	ALL2_PSOOV | Mite group 2 allergen Pso o 2 AltName: Full=Allergen Pso o A AltName: Allergen=Pso o 2 Flags: Precursor
82	cds.comp29245_c0_seq1|m.56272	5	5	R4WEH7_9HEMI | Ornithine decarboxylase Flags: Fragment
82	Der p 1.0106	3	3	Der p 1.0106
82	cds.comp46788_c0_seq1|m.88368	3	3	B7Q8W6_IXOSC | Alkyl hydroperoxide reductase thiol specific antioxidant putative EC=1.11.1.15
82	cds.comp105854_c0_seq1|m.181986	2	2	A1IHK5_HAELO | Serine carboxypeptidase
82	cds.comp78049_c0_seq1|m.141912	2	2	Q9XXZ6_BOMMO | Polyubiquitin
83	cds.comp117245_c0_seq1|m.197139	5	5	ALL2_PSOOV | Mite group 2 allergen Pso o 2 AltName: Full=Allergen Pso o A AltName: Allergen=Pso o 2 Flags: Precursor
84	Der p 2.0109	12	0	Der p 2.0109
84	Der p 2.0112	12	0	Der p 2.0112
84	Der p 2.0107	12	0	Der p 2.0107

Spot number	Accession	#Peptides	#Unique	Description
84	Der p 2.0101	12	0	Der p 2.0101
84	Der p 2.0104	12	0	Der p 2.0104
84	Der p 2.0105	12	0	Der p 2.0105
84	Der p 2.0113	11	0	Der p 2.0113
84	Der p 2.0114	9	0	Der p 2.0114
84	cds.comp105854_c0_seq1|m.181986	6	6	A1IHK5_HAELO | Serine carboxypeptidase
84	cds.comp117252_c0_seq1|m.197148	4	4	ALL2_PSOOV | Mite group 2 allergen Pso o 2 AltName: Full=Allergen Pso o A AltName: Allergen=Pso o 2 Flags: Precursor
84	cds.comp11404_c0_seq1|m.20428	4	4	A5X5X4_DERFA | Allergen SubName: Full=Group 22 allergen Der f 22
84	cds.comp23278_c0_seq1|m.44326	3	3	Q8MWR6_DERPT | 14.5 kDa bacteriolytic enzyme
84	Der p 1.0107	2	2	Der p 1.0107
85	Der p 2.0113	4	4	Der p 2.0113
86	Der p 2.0109	12	0	Der p 2.0109
86	Der p 2.0107	12	0	Der p 2.0107
86	Der p 2.0112	12	0	Der p 2.0112
86	Der p 2.0101	12	0	Der p 2.0101
86	Der p 2.0104	12	0	Der p 2.0104
86	Der p 2.0105	12	0	Der p 2.0105
86	Der p 2.0113	11	0	Der p 2.0113
86	Der p 2.0114	9	0	Der p 2.0114

Spot number	Accession	#Peptides	#Unique	Description
86	cds.comp11404_c0_seq1|m.20428	5	5	A5X5X4_DERFA | Allergen SubName: Full=Group 22 allergen Der f 22
86	cds.comp112919_c0_seq1|m.190975	5	5	comp112919_c0_seq1|g.190975 ORF comp112919_c0_seq1|g.190975 comp112919_c0_seq1|m.190975 type:complete len:106 (-) comp112919_c0_seq1:1574-1891(-)NoHit
86	cds.comp23278_c0_seq1|m.44326	2	2	Q8MWR6_DERPT | 14.5 kDa bacteriolytic enzyme
87	Der p 2.0107	14	0	Der p 2.0107
87	Der p 2.0101	14	0	Der p 2.0101
87	Der p 2.0105	14	0	Der p 2.0105
87	Der p 2.0102	14	0	Der p 2.0102
87	Der p 2.0109	14	0	Der p 2.0109
87	Der p 2.0112	14	0	Der p 2.0112
87	Der p 2.0103	14	0	Der p 2.0103
87	Der p 2.0104	14	0	Der p 2.0104
87	Der p 2.0111	14	0	Der p 2.0111
87	Der p 2.0113	13	0	Der p 2.0113
87	Der p 2.0114	10	0	Der p 2.0114
87	cds.comp112917_c0_seq1|m.190971	4	4	no hit
87	cds.comp11404_c0_seq1|m.20428	3	3	A5X5X4_DERFA | Allergen SubName: Full=Group 22 allergen Der f 22
87	cds.comp23278_c0_seq1|m.44326	2	2	Q8MWR6_DERPT | 14.5 kDa bacteriolytic enzyme
88	Der p 2.0109	13	0	Der p 2.0109
88	Der p 2.0102	13	0	Der p 2.0102

Spot number	Accession	#Peptides	#Unique	Description
88	Der p 2.0104	13	0	Der p 2.0104
88	Der p 2.0111	13	0	Der p 2.0111
88	Der p 2.0112	13	0	Der p 2.0112
88	Der p 2.0113	13	0	Der p 2.0113
88	Der p 2.0114	10	0	Der p 2.0114
88	cds.comp112917_c0_seq1|m.190971	4	4	no hit
88	cds.comp11404_c0_seq1|m.20428	3	3	A5X5X4_DERFA | Allergen SubName: Full=Group 22 allergen Der f 22
88	cds.comp23278_c0_seq1|m.44326	2	2	Q8MWR6_DERPT | 14.5 kDa bacteriolytic enzyme
88	cds.comp86172_c0_seq1|m.154673	2	2	R4FNL2_RHOPR | Putative cathepsin b-like proteinase
89	Der p 2.0107	5	1	Der p 2.0107
89	cds.comp94280_c0_seq1|m.166903	5	1	ALL2_DERPT | Mite group 2 allergen Der p 2 AltName: Full=Allergen Der p II AltName: Full=DPX AltName: Allergen=Der p 2 Flags: Precursor
89	cds.comp23278_c0_seq1|m.44326	4	4	Q8MWR6_DERPT | 14.5 kDa bacteriolytic enzyme
89	cds.comp72385_c0_seq1|m.132552	2	2	Q9GUA9_AMBAM | Macrophage migration inhibitory factor
90	Der p 2.0109	8	1	Der p 2.0109
90	Der p 2.0112	8	0	Der p 2.0112
90	Der p 2.0113	7	0	Der p 2.0113
90	cds.comp112920_c0_seq1|m.190979	7	7	no hit
90	cds.comp11404_c0_seq1|m.20428	6	6	A5X5X4_DERFA | Allergen SubName: Full=Group 22 allergen Der f 22
90	Der p 2.0114	5	0	Der p 2.0114

Spot number	Accession	#Peptides	#Unique	Description
90	cds.comp103957_c0_seq1|m.179732	3	3	A1KXG2_DERFA | Peptidyl-prolyl cis-trans isomerase EC=5.2.1.8
90	cds.comp86172_c0_seq1|m.154673	2	2	R4FNL2_RHOPR | Putative cathepsin b-like proteinase
90	cds.comp101224_c0_seq1|m.176142	2	2	D3TS01_GLOMM | Nucleoside diphosphate kinase
90	cds.comp23278_c0_seq1|m.44326	2	2	Q8MWR6_DERPT | 14.5 kDa bacteriolytic enzyme
90	cds.comp86985_c0_seq1|m.155823	2	2	no hit
90	cds.comp115292_c0_seq1|m.194597	2	2	C9W1L3_RHISA | Hypothetical conserved secreted protein
91	Der p 2.0109	7	1	Der p 2.0109
91	Der p 2.0112	7	1	Der p 2.0112
91	cds.comp112920_c0_seq1|m.190979	6	6	no hit
91	cds.comp103957_c0_seq1|m.179732	2	2	A1KXG2_DERFA | Peptidyl-prolyl cis-trans isomerase EC=5.2.1.8
91	cds.comp86172_c0_seq1|m.154673	2	2	R4FNL2_RHOPR | Putative cathepsin b-like proteinase
91	cds.comp115292_c0_seq1|m.194597	2	2	C9W1L3_RHISA | Hypothetical conserved secreted protein
91	cds.comp101224_c0_seq1|m.176142	2	2	D3TS01_GLOMM | Nucleoside diphosphate kinase
91	cds.comp23278_c0_seq1|m.44326	2	2	Q8MWR6_DERPT | 14.5 kDa bacteriolytic enzyme
91	cds.comp61455_c0_seq1|m.114274	2	2	B7PAQ0_IXOSC | Gamma-glutamyltransferase putative EC=2.3.2.2
92	Der p 2.0112	6	1	Der p 2.0112
92	Der p 2.0109	6	1	Der p 2.0109
92	cds.comp23278_c0_seq1|m.44326	4	4	Q8MWR6_DERPT | 14.5 kDa bacteriolytic enzyme
92	cds.comp72385_c0_seq1|m.132552	4	4	Q9GUA9_AMBAM | Macrophage migration inhibitory factor

Spot number	Accession	#Peptides	#Unique	Description
92	Der p 9.0102	3	3	Q8MWR4_DERPT Serine protease LM-1 (Fragment) OS=Dermatophagoides pteronyssinus PE=2 SV=2
93				no identification
94	cds.comp23278_c0_seq1|m.44326	4	4	Q8MWR6_DERPT | 14.5 kDa bacteriolytic enzyme
94	cds.comp94280_c0_seq1|m.166903	2	2	ALL2_DERPT | Mite group 2 allergen Der p 2 AltName: Full=Allergen Der p II AltName: Full=DPX AltName: Allergen=Der p 2 Flags: Precursor
95	cds.comp23278_c0_seq1|m.44326	2	2	Q8MWR6_DERPT | 14.5 kDa bacteriolytic enzyme
96	cds.comp23278_c0_seq1|m.44326	3	3	Q8MWR6_DERPT | 14.5 kDa bacteriolytic enzyme
97	cds.comp122816_c0_seq1|m.203611	9	9	C1IE32_9HEXA | Beta-1 3-D-glucanase EC=3.2.1.6 SubName: Full=Endo-beta-1 3-glucanase EC=3.2.1.39 Flags: Precursor
97	Der p 3.0101	8	1	P39675_DERP3_DERPT Mite allergen Der p 3 OS=Dermatophagoides pteronyssinus GN=DERP3 PE=1 SV=1
97	cds.comp72130_c0_seq1|m.132048	8	1	DERP3_DERPT | Mite allergen Der p 3 EC=3.4.21.- AltName: Full=Allergen Der p III AltName: Allergen=Der p 3 Flags: Precursor
97	cds.comp72078_c0_seq1|m.131978	6	6	A7UI22_AMBAM | Lospin 7
97	Der p 1.0120	3	2	Der p 1.0120
97	Der p 6.0101	3	3	Der p 6.0101
97	Der p 9.0102	3	3	Q8MWR4_DERPT Serine protease LM-1 (Fragment) OS=Dermatophagoides pteronyssinus PE=2 SV=2
97	cds.comp116084_c0_seq1|m.195549	2	1	PEPT1_DERPT | Peptidase 1 EC=3.4.22.65 AltName: Full=Allergen Der p I AltName: Full=Major mite fecal allergen Der p 1 AltName: Allergen=Der p 1 Flags: Precursor
97	cds.comp4546_c0_seq1|m.7079	2	2	R4WCI9_9HEMI | Unkown protein
97	cds.comp124461_c0_seq1|m.205142	2	2	A1YW13_DERFA | Der f 1 allergen
97	cds.comp114198_c0_seq1|m.193051	2	2	B7Q0D4_IXOSC | Fumarylacetoacetase putative EC=3.7.1.2
97	cds.comp86162_c0_seq1|m.154650	2	2	D6WGZ1_TRICA | Cathepsin B
98	cds.comp122816_c0_seq1|m.203611	4	4	C1IE32_9HEXA | Beta-1 3-D-glucanase EC=3.2.1.6 SubName: Full=Endo-beta-1 3-glucanase EC=3.2.1.39 Flags: Precursor

Spot number	Accession	#Peptides	#Unique	Description
99	cds.comp122816_c0_seq1|m.203611	14	14	C1IE32_9HEXA | Beta-1 3-D-glucanase EC=3.2.1.6 SubName: Full=Endo-beta-1 3-glucanase EC=3.2.1.39 Flags: Precursor
99	cds.comp72130_c0_seq1|m.132048	6	0	DERP3_DERPT | Mite allergen Der p 3 EC=3.4.21.- AltName: Full=Allergen Der p III AltName: Allergen=Der p 3 Flags: Precursor
99	cds.comp72131_c0_seq1|m.132050	6	0	DERP3_DERPT | Mite allergen Der p 3 EC=3.4.21.- AltName: Full=Allergen Der p III AltName: Allergen=Der p 3 Flags: Precursor
